# Sulfonyl Anthranilic Acid Analogues Display Pan-Serotype Anti-Dengue Activity by Downregulating the Expression of Ribosomal Proteins Encoded by 5′-Terminal Oligopyrimidine Motif-Containing mRNA

**DOI:** 10.1021/acs.jmedchem.5c03414

**Published:** 2026-03-10

**Authors:** Chin Piaw Gwee, Tommaso Felicetti, Kitti Wing Ki Chan, Min Jie Alvin Tan, Muhammad Danial Bin Mohd Mazlan, Ciro Milite, Giacomo Pepe, Chiara Sarnari, Xiao Dan Ng, Wint Wint Phoo, Jasmine Hwee Yee Tan, Marcus G. Mah, Satoru Watanabe, Jing Xiu Huang, Serena Massari, Oriana Tabarrini, Stefano Sabatini, Pietro Campiglia, Gianluca Sbardella, Gavin J. D. Smith, Sylvie Alonso, Alfred Xuyang Sun, Radoslaw M. Sobota, Subhash G. Vasudevan, Giuseppe Manfroni

**Affiliations:** † Program in Emerging Infectious Diseases, 121579 Duke-NUS Medical School, 8 College Road, 169857 Singapore, Singapore; ‡ Infectious Diseases Translational Research Programme, Department of Microbiology & Immunology, Yong Loo Lin School of Medicine, 117545 Singapore, Singapore; § Dipartimento di Scienze Farmaceutiche, 9309 Università degli Studi di Perugia, Via Del Liceo, 1-06123 Perugia, Italy; ∥ Dipartimento di Farmacia, Università degli Studi di Salerno, Via Giovanni Paolo II, 132, 84084 Fisciano, Italy; ⊥ Immunology programme, Life Sciences Institute, 37580 National University of Singapore, 117456 Singapore, Singapore; # Program in Neuroscience and Behavioral Disorders, Duke-NUS Medical School, 8 College Road, 169857 Singapore, Singapore; ∇ Department of Anesthesiology, State Key Laboratory of Oncology in South China, Guangdong Provincial Clinical Research Center for Cancer, Sun Yat-sen University Cancer Center, Guangzhou 510060, Guangdong, People’s Republic of China; ○ Communicable Diseases Agency, 307684 Singapore, Singapore; ◆ Institute of Molecular and Cell Biology, Agency for Science (IMCB), Technology and Research (A*STAR), 138673 Singapore, Singapore; ¶ Institute for Biomedicine and Glycomics, Griffith University, Southport, Queensland 4222, Australia

## Abstract

Dengue virus (DENV) remains a major global health concern without effective treatments. Previously, we identified sulfonyl anthranilic acid (SAA) derivatives (compounds **1** and **2**) as potent pan-DENV inhibitors, likely targeting a primate-specific factor. Here, mass spectrometry-based target deconvolution revealed that SAA compounds downregulate ribosomal protein expression, some of which are essential for DENV replication, as confirmed by siRNA-knockdown studies. This novel mechanism aligns with the broad-spectrum antiviral activity of compounds **1** and **2**. Moreover, compound **1** was also effective against the Zika virus in a human brain organoid model. The subsequent medicinal chemistry optimization process resulted in the identification of compound **7**, which demonstrated an EC_50_ value of 50 nM against DENV-2, promising broad-spectrum potential and favorable *in vitro* ADME properties. Further studies indicated that these compounds modulate the 5**′**-terminal oligopyrimidine (5**′**-TOP) motif in ribosomal mRNAs. These findings open a new avenue for antiviral development by targeting a previously unexplored host pathway.

## Graphical abstract

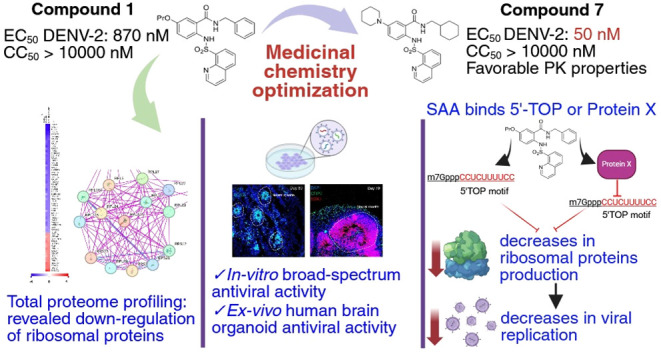

## Introduction

Dengue is a fast-spreading mosquito-borne disease caused by the four distinct dengue virus (DENV) serotypes DENV-1–4.^1^ DENV is a member of the genus *Flavivirus*, family *Flaviviridae*, alongside other arboviruses such as Zika virus (ZIKV), West Nile virus (WNV), and Yellow Fever virus (YFV).^2^ DENV has a global distribution, with Southeast Asia, the Americas, and the Western Pacific regions being particularly impacted and over 100 countries experiencing endemic DENV transmission. Dengue has emerged as a substantial public health concern, as evidenced by the European Centre for Disease Prevention and Control reporting of 3.6 million dengue cases and over 1900 dengue-related fatalities from the onset of 2025 to July of the same year across 94 countries.^3^ Additionally, the World Health Organization (WHO) has cautioned that approximately 4 billion individuals are susceptible to dengue infection, with an estimated average annual incidence of 390 million cases.^4^ The majority of dengue infections are either asymptomatic or manifest solely as mild symptoms, including fever, headaches, and joint pain. However, approximately 1–5% of cases progress to severe dengue, manifesting as either classical dengue fever (DF) or the life-threatening dengue hemorrhagic fever (DHS)/dengue shock syndrome (DSS).^5^ DENV genome is a single-stranded positive-sense RNA approximately 10.7 kb in length, with a type I cap structure at the 5′ end but lacking the 3′ end poly-A tail. The genome encodes a single open reading frame, which gives rise to three structural proteins, capsid (C), prM (premembrane), and envelope (E), and seven nonstructural (NS) proteins (NS1, NS2A, NS2B, NS3, NS4A, NS4B, and NS5). The open reading frame is flanked by 5′ and 3′ untranslated regions (UTRs), which form highly conserved secondary structures that are essential for efficient RNA replication and translation.^2,6^

Despite intense research efforts, to date, there are no currently effective antiviral drugs available against DENV serotypes,^7,8^ with some direct-acting antiviral compounds (e.g., mosnodenvir/JNJ-64281802) that have recently failed to progress to clinical use.^9–11^ In the area of prophylaxis, the promising licensed vaccine Qdenga^12^ offers partial protection, with notably reduced efficacy against the DENV-3 serotype. Vaccine administration in individuals without prior DENV exposure carries a risk of antibody-dependent enhancement (ADE), which has been associated with an increased rate of hospitalization and the development of severe forms of dengue.^13^

A further strategy involves the identification of host-targeting agents that have the potential to circumvent the development of viral resistance^14,15^ while simultaneously conferring a broad-spectrum activity. This latter feature is of particular utility in the context of flaviviruses, which are known to circulate simultaneously in endemic regions and are frequently associated with coinfections.^16,17^ In recent years, the advent of multiomics approaches has led to the identification of numerous host factors (i.e., cyclophilin,^18–20^ heat shock proteins,^21,22^ α-glucosidase,^23,24^ kinases,^25,26^ ribosomal proteins,^27–29^ and signal peptide peptidase^30^) that have been shown to be essential for the replication of various viruses. In the context of DENV, research in this field has led to the identification of host-targeting agents, with some (i.e., celgosivir, lovastatin, ivermectin,^31^ and resomelagon) even reaching clinical trials. However, the first three agents failed to significantly reduce viral load or fever burden in dengue patients.^32,33^ Studies on the efficacy of resomelagon are still in the preliminary phase.^34^

Over the years, we have been involved in the discovery of new molecules as anti-DENV agents.^35–38^ More recently, we identified a novel class of sulfonyl anthranilic acid (SAA) derivatives as potent pan-serotype DENV inhibitors.^39^ The most potent compounds of the series, **1** and **2** (Figure 1), display EC_50_ values ranging from 0.54 to 1.36 μM and selectivity indexes (SIs) higher than 100. Importantly, the antiviral effects of compounds **1** and **2** were independent of DENV NS3 and NS5 inhibition, and their effects were primate-specific, suggesting the involvement of host factors in their mechanism of action. In accordance with these observations, no resistant viral mutants were produced after 30 serial passages in the presence of SAA. Despite the considerable potential of this novel class of presumed host-targeting agents, the absence of a known molecular target has slowed the medicinal chemistry optimization process. This limitation precluded the application of a target-based drug discovery strategy and prevented the employment of a biochemical or enzymatic assay that would otherwise facilitate the rapid delineation of a precise Structure–Activity Relationship (SAR).

**Figure 1:**
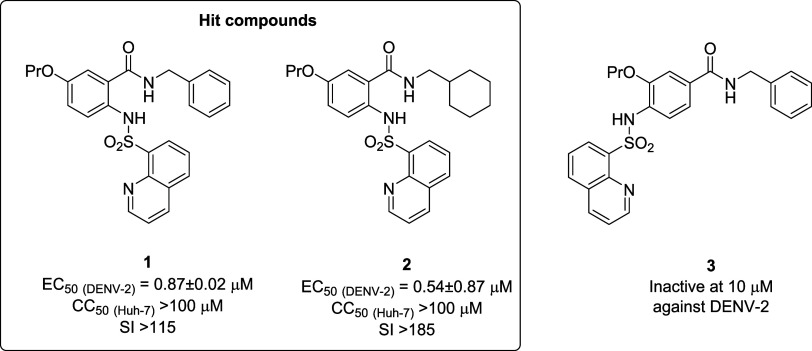
Chemical structures of the SAA derivatives reported in the previous work: hit compounds **1** and **2**, and the inactive derivative **3**.^39^

Accordingly, in this study, we first conducted an in-depth investigation of the mechanism of action of the SAA class, employing compounds **1** and **2** as the most potent representatives of the series and compound **3** (Figure 1) as an inactive analogue. Our findings revealed an innovative mechanism of action that is likely responsible for the broad-spectrum antiviral activity of compounds **1** and **2** against various RNA viruses, as well as the observed activity of compound **1** in an *ex vivo* human brain organoid of ZIKV infection. Subsequently, a ligand-based strategy was employed to refine the SAR of the SAA series, with compounds **1** and **2** serving as starting points. This endeavor culminated in the design and synthesis of new SAA derivatives **4–37** and the identification of compound **7** as a nanomolar pan-serotype DENV inhibitor. Further mechanistic studies revealed a previously unexplored antiviral pathway in which selective downregulation of 5′**-**TOP motif-mediated ribosomal protein expressions impairs virus replication without affecting global protein translation.

## Results and Discussion

### Target Deconvolution Using Proteomic Approaches

We previously showed that the SAA inhibitors exhibited a primate-specific antiviral activity and a high barrier to resistance development,^39^ suggesting that cellular host factors may play a key role in their antiviral mechanism of action. Their low-micromolar potency against different DENV serotypes (Table 1) coupled with a no/low toxicity profile *in vitro* prompted us to investigate the cellular binding targets of SAA compounds.

**Table 1:** Antiviral Activity against RNA Viruses of Compounds **1** and **2^a^**

			EC_50_ (μM) against different viruses^b^		CC_50_ (μM) on different cell lines^c^	
	cell line	virus	1	2	1	2
flaviviruses	Huh-7	DENV-1	1.36 ± 0.47^d^	0.84 ± 0.1^d^	>100	>100
		DENV-2	0.87 ± 0.02^d^	0.54 ± 2.8^d^		
		DENV-3	0.94 ± 0.02^d^	0.80 ± 0.05^d^		
		DENV-4	0.95 ± 0.12^d^	0.85 ± 0.02^d^		
		ZIKV	0.54 ± 0.07	0.39 ± 0.04		
	Vero	YF17D	1.08 ± 0.25	1.24 ± 0.17	>100	>100
		JEV SA-14–2–2	0.83 ± 0.33	0.34 ± 0.24		
		WNV_Kunjin_	2.22 ± 0.48	1.01 ± 0.31		
alphaviruses		CHIKV-ROSS	0.90 ± 0.06	0.29 ± 0.16		
		CHIKV-EAS	0.29 ± 0.04	0.12 ± 0.03		
enterovirus	SH-SY5Y	EV-A71	0.16 ± 0.03	0.07 ± 0.04	∼50	∼50
coronavirus	Caco2	229E	0.16 ± 0.06	0.44 ± 0.24	ND	ND

a Data represent mean ± SD from two independent experiments. Abbreviations: ND-not determined.

b EC_50_ is the effective concentration that inhibits 50% of virus infection

c CC_50_ is the cytotoxic concentration that reduces 50% of cell viability

d Data previously published on ref 39.

To this end, we first performed a global proteome profiling using Liquid Chromatography–Mass Spectrometry (LC-MS) to investigate the impact of compound **1** on the host cellular proteome. A comprehensive analysis of protein abundance between mock-treated and compound **1**-treated samples, both in the absence and presence of infection, was performed to capture both compound-specific and infection-associated proteomic landscapes. Comparative heat map analysis of the total proteome revealed clear alterations in the protein expression following compound **1** treatment. Specifically, 62 proteins were differentially expressed, with 46 proteins downregulated and 16 proteins upregulated, each showing at least a 1.5-fold difference in Log2 (ratio) compared to noninfected DMSO-treated cell control (Figure 2A).

**Figure 2:**
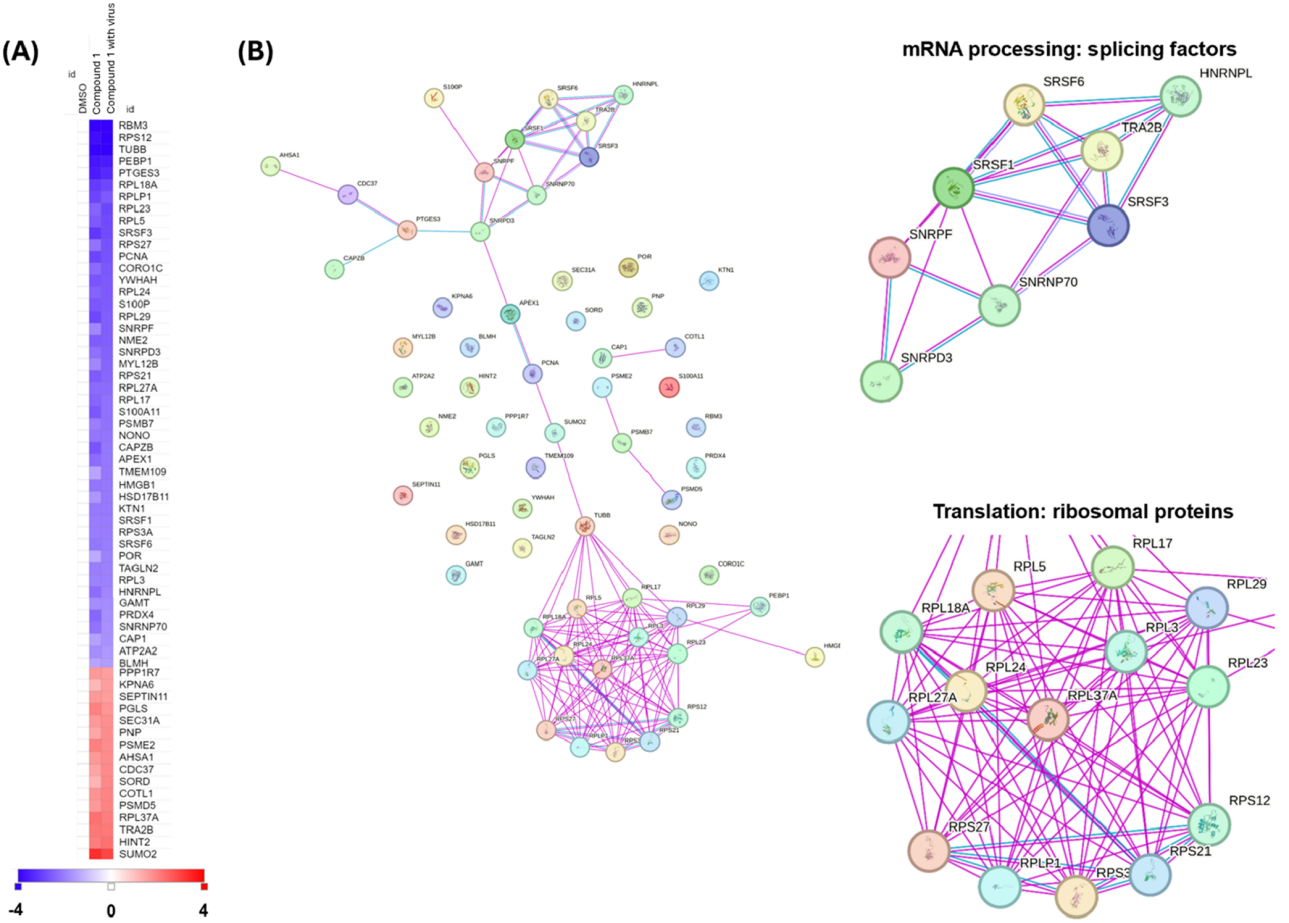
Total proteome analysis of compound **1**-treated cells using Liquid Chromatography–Mass Spectrometry (LC-MS). Huh-7 cells were infected with DENV-2 at MOI 5 for 1 h, and compound **1** was added at a concentration of 10 μM for 24 h. The cells were lysed, and protein abundance was quantified by LC-MS. (A) Heat map analysis of proteins showing −1.5 ≥ log_2_ (fold change of treated vs DMSO mock treated) ≥ 1.5 following compound **1** treatment. Proteins encoded in blue are those downregulated (log2 fold change ≤ −1.5) after compound **1** treatments, while proteins encoded in red are those upregulated (log2 fold change ≥ 1.5) following treatment. (B) STRING protein–protein interaction analysis of differentially expressed proteins. Red line represents experimentally validated protein–protein interactions, and the blue line indicates interactions derived from curated databases. Magnified views highlight two clusters of proteins regulated by compound **1**. Data shown are from a single experiment.

To further dissect the biological relevance of these changes, we performed protein–protein interaction and pathway enrichment analysis using STRING.^40^ Interestingly, the majority of differentially expressed proteins clustered into two major functional categories: proteins involved in mRNA processing, particularly splicing factors, and those associated with the translational machinery, primarily ribosomal proteins (Figure 2B). Ribosomal proteins accounted for the largest subset of downregulated proteins, implying a widespread suppression of ribosomal biogenesis and protein synthesis capacity upon compound **1** treatment. Such a concerted downregulation in ribosomal protein expression points toward a potential mechanism whereby compound **1** modulates host translation to restrict viral replication. In parallel, alterations in the expression of splicing factor indicate that compound **1** may also perturb host mRNA processing pathways, some of which are known to be exploited by viruses during infection.^41–43^ Together, these findings highlight that compound **1** likely exerts its antiviral activity through concerted regulation of RNA metabolism and translation. Although the global proteomic analysis provided valuable insights into pathways modulated by compound **1**, the data set was generated from a single exploratory experiment and is therefore limited by biological replication and statistical power. Accordingly, the proteomics data were used primarily for hypothesis generation rather than definitive quantitative conclusions. Key ribosomal proteins identified in the proteome analysis were subsequently validated using independent functional assays, including siRNA-mediated knockdown studies, assessment of global protein synthesis by puromycin labeling, and targeted reporter assays, as described in subsequent sections.

### Effect of SAA Inhibitors on Global Cellular Translation

Given the prominence of ribosomal proteins in the proteomic data set, we next examined whether SAA compounds exert a functional impact on global translation. To this end, we performed a puromycin labeling assay, which allows direct measurement of nascent protein synthesis. As shown in Figure 3, the treatment of SAA compounds including compounds **1–3** did not result in any detectable reduction in overall protein synthesis when compared with virus-infected and DMSO-treated controls. In contrast, treatment with cycloheximide, a known protein translation inhibitor included as a positive control, significantly reduced global protein synthesis. These findings indicate that despite the downregulation of ribosomal proteins, SAA compounds do not impair global translation under the tested conditions. This suggests that their antiviral activity may involve more selective modulation of ribosome-associated pathways or other host processes exploited during viral replication.

**Figure 3:**
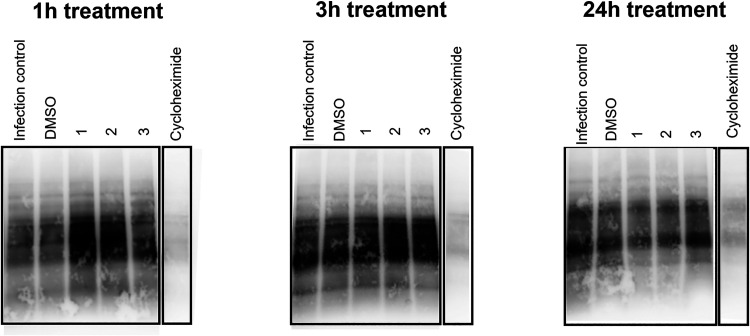
Investigation of the impact of SAA inhibitors on global cellular translation. Huh-7 cells were infected with DENV-2 at MOI 1, followed by SAA compounds treatment at a concentration of 10 μM for different durations (1, 3, and 24 h) starting 1 h postinfection. At the indicated time point, the cells were pulsed with puromycin for 40 min to label newly translated proteins. The cells were then lysed, and 10 μg of proteins from each sample was analyzed by Western blotting using antipuromycin antibodies. Cycloheximide (a known protein translation inhibitor) treatment at 10 μM was included as a positive control.

### siRNA-Mediated Knockdown of Ribosomal Genes

Viruses rely extensively on cellular host factors, including host translation machinery and ribosome-associated factors, for their efficient replication. Previous studies have shown that DENV NS1 interacts with multiple ribosomal proteins, and depletion of RPL18 significantly inhibited virus replication.^28^ In addition, the RPLP1/RPLP2 heterodimer has been identified as essential for flavivirus replication by mitigating the ribosome pausing on the DENV genome, hence promoting translation initiation and protein stability.^27,44^ Similar reliance on ribosomal proteins has also been reported in other RNA viruses such as enterovirus A71 where the 3D protein (Enteroviral RdRp) has been shown to interact with ribosomal proteins RPS6, and knockdown of RPS6 inhibits the virus replication.^45^

Since global translation is not affected by SAA inhibitors, we next investigated the functional importance of ribosomal proteins during DENV replication using siRNA-mediated knockdown experiments. Selection of siRNA targeted genes was guided by the magnitude of protein expression changes (≥log2 fold-change) identified in total proteome analysis, as well as the ribosomal proteins that were differentially expressed during DENV infection (Figure S1). RPLP1, previously identified as an essential factor for DENV replication,^27,44^ was included as a positive control. Consistent with earlier reports, RPLP1 knockdown resulted in a pronounced inhibitory effect on viral replication, with more than 80% reduction in intracellular RNA (Figure 4). Similarly, depletion of RPS3a and RPS27 also led to comparable inhibitory effect, with approximately 80% reduction of viral RNA. In contrast, knockdown of RPL29 had no significant impact on DENV replication, whereas knockdown of RPL24, RPS12 and RPL18a exhibited moderate impact on DENV replication, resulting in approximately 70%, 65% and 45% reduction in DENV intracellular RNA, respectively. These findings indicate that only a subset of ribosomal proteins is essential for efficient DENV replication. Not all ribosomal proteins are strictly indispensable for ribosome biogenesis or translation. Several ribosomal proteins possess extra-ribosomal functions, such as regulating immune signaling and apoptosis.^46–48^ Accordingly, knockdown of these genes such as RPLP1,^27^ RPL18,^28^ RPL19,^29^ and RPS25 ^49^ has minimal or no impact on overall protein synthesis, despite significantly impairing viral replication. Consistent with these observations, the ribosomal proteins regulated by compound **1** are unlikely to be essential for global protein translation but instead contribute to extra-ribosomal functions exploited by viruses. Although compound **1** did not completely abolish production of ribosomal proteins (Figure S2), the coordinated partial reduction of multiple ribosomal proteins may collectively weaken the capacity of the host cell to support viral replication. Taken together, these results support a model in which SAA inhibitors do not impair global translation but instead selectively perturbing ribosome-associated functions that are critical for DENV replication.

**Figure 4:**
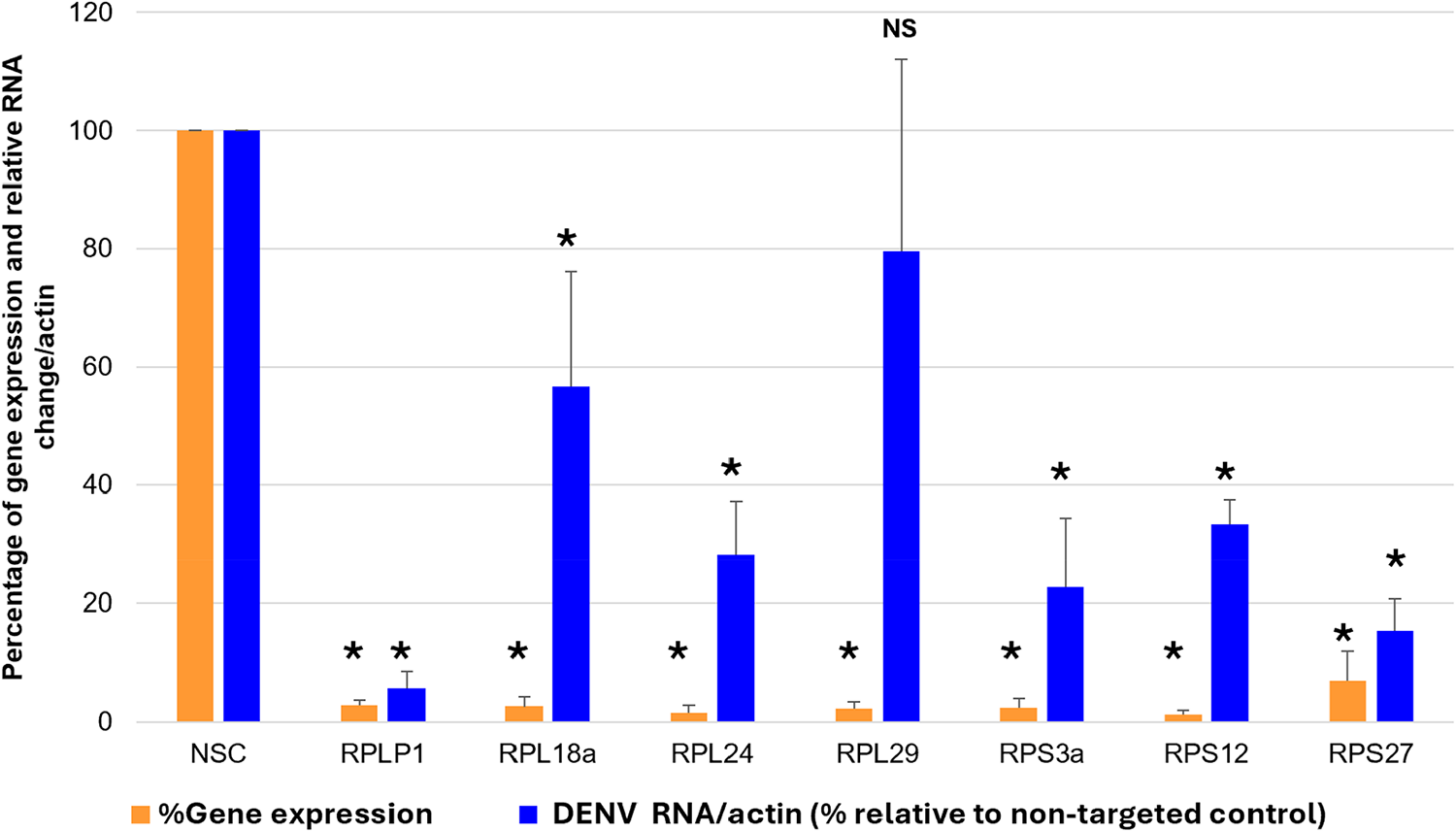
siRNA-mediated knockdown of ribosomal proteins identified from the total proteome analysis. Cells were transfected with either a nonsilencing control siRNA (NSC) or the siRNA targeting different genes selected from total proteome analysis. The cells were then infected with DENV-2 at an MOI value of 1 at 48 h post-transfection. Samples were harvested 24 h postinfection and quantified by qRT-PCR. Blue bar: gene expression levels of the targeted gene after siRNA-knockdown in Huh-7 cells. Orange bar: DENV intracellular RNA with respect to nontargeted control after siRNA-knockdown of the respective target gene. Data presented are the mean ± SD from two independent experiments. Statistical analysis was conducted using Student’s unpaired *t* test and considered significant if *p* < 0.05 (indicated by *) compared to nonsilencing control. NS: no significant difference.

### Evaluation of the Potential Broad-Spectrum Antiviral Activity of SAA Derivatives

In light of our findings that host cellular targets are involved in the mechanism of action of SAA derivatives and recognizing the novelty of selectively modulating ribosomal protein expression to inhibit DENV replication, we next explored whether this mode of action could be extended to achieve broad-spectrum antiviral activity. To investigate this possibility, we tested compounds **1** and **2** against a panel of selected flaviviruses, including Zika virus (ZIKV), Japanese encephalitis virus (JEV), Yellow Fever 17D virus (YF17D), West Nile Kunjin virus (WNV_Kunjin_), and flavivirus-unrelated RNA viruses, such as the alphaviruses Chikungunya ROSS (CHIKV-ROSS) and East African (CHIKV-EAS) strains, enterovirus EV-A71, and coronavirus 229E (CoV 229E) (Table 1 and Figure 5).

**Figure 5:**
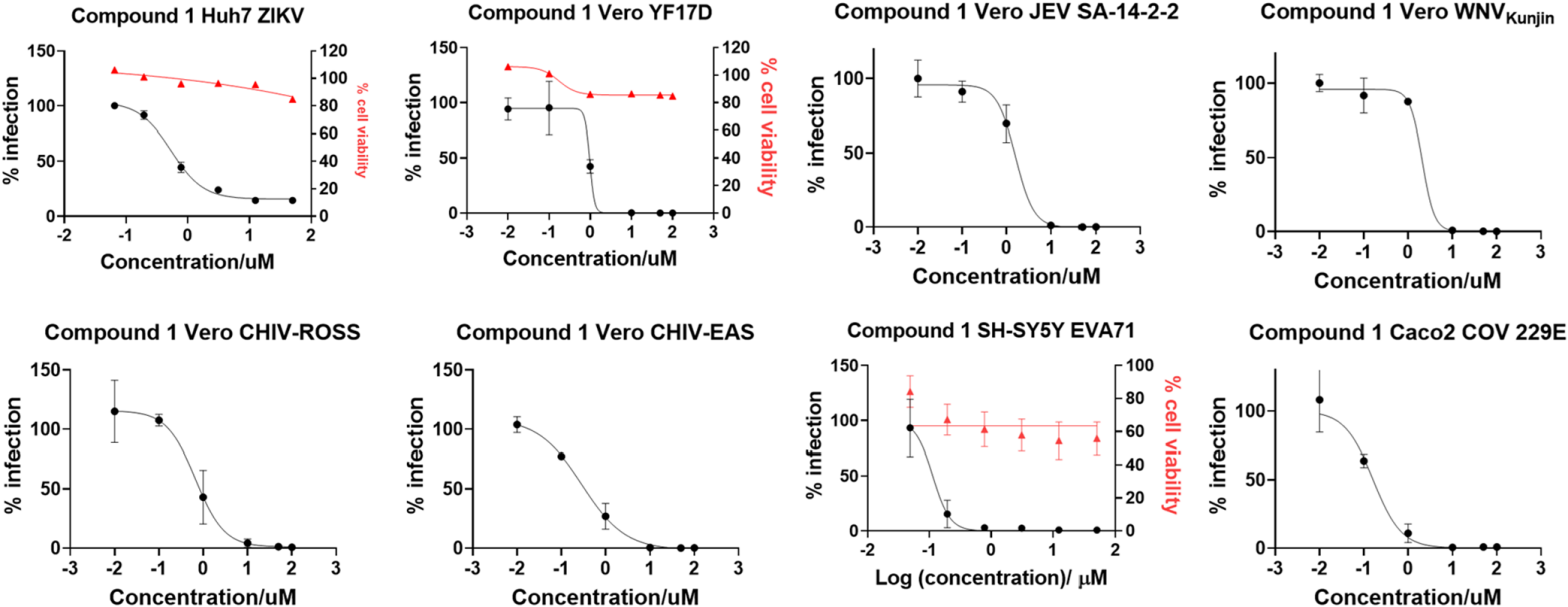
Dose–response viral inhibition of compound **1** against other RNA viruses. Antiviral efficacy of compound **1** against ZIKV (Genbank accession number KJ776791), YF17D (Genbank accession number X03700), JEV SA-14–2–2 (Genbank accession number AF315119), WNV_Kunjin_ (Genbank accession number KX394398.1), CHIKV-ROSS (Genbank accession number AF490259.3), CHIKV-EAS, Enterovirus EV71 (Genbank accession number AF316321), and Coronavirus 229E (CoV 229E). The efficacy of the compound **1** are presented as black curves while cytotoxicity is shown in red curves.

As shown in Table 1 and in Figure 5, our data shows that the antiviral efficacy of compounds **1** and **2** against other flaviviruses and RNA viruses was consistent with that observed against DENV,^39^ with both compounds showing sub or low-micromolar EC_50_ values against the tested viruses. In addition, the results demonstrated that the CC_50_ values for both compounds **1** and **2** were greater than or approximately equal to the highest drug concentration tested, thereby confirming that these compounds do not induce significant cellular toxicity despite targeting a host factor. Taken together, the potent antiviral efficacy combined with the favorable toxicity profile strongly suggests that both **1** and **2** have significant potential not only as pan-anti-DENV compounds but also as broad-spectrum antiviral agents against RNA viruses.

### Efficacy of SAA Inhibitors against ZIKV in *Ex Vivo* Human Brain Organoid

We next sought to ascertain whether the modulation of ribosomal protein expression by SAA compounds translates into antiviral activity in a more complex and physiologically relevant environment. Given the intricate nature of the mechanism of action underlying the antiviral activity of SAA analogues and the evidence suggesting a primate-specific mode of action, it prompted us to look beyond our established mouse model of DENV infection^50^ to more physiological human organoid models. However, organoid models suitable for DENV infection remain limited, representing only about 6% of all organoid systems developed for flaviviruses, particularly those based on liver cells.^51^ Notably, a liver organoid model of DENV infection was only recently reported for the evaluation of antiviral compounds.^52^ Considering that ZIKV is among the flaviviruses most phylogenetically related to DENV, we therefore established a neurotrophic ZIKV infection model to further evaluate the antiviral potential of SAA compounds.^53,54^

Human embryonic stem cells were differentiated into dorsal forebrain organoid following the established protocol,^53,54^ and the organoid development was tracked using specific markers at various differentiation stages by Western blotting and immunofluorescence staining (Figure S3A–C). We then assessed the ZIKV infectivity in the cortical organoids at different differentiation time points (day 20: neural proliferation stage; day 56: early maturation stage; day 90: late maturation stage)^55^ and showed that both neural proliferation and early maturation stage cortical organoids are highly permissive to ZIKV infection (Figure S3D). Based on these findings, antiviral evaluations were performed using day 60 early matured organoids, as they reflect the fetal brain at approximately 8–9 weeks postconception^56^ and are highly susceptible to ZIKV-induced neuropathology.^57^

Treatment of ZIKV-infected cortical organoids with compound **1** at a concentration of 5 μM resulted in an approximately 70% reduction in ZIKV infection at 48 h postinfection. The adenosine nucleoside inhibitor NITD008 ^58^ was included as a positive control and exhibited comparable efficacy, achieving approximately 80 to 90% reduction in ZIKV infection in the same time frame (Figure 6A). In contrast, the inactive SAA analogue compound **3** showed no antiviral activity in the human brain organoid model (Figure 6A). Interestingly, both compound **1** and NITD008 treatments exhibited lower antiviral efficacy at 24 h postinfection. The reduced potency at the early time point may be attributed to slow diffusion or limited compound penetration into the 3D organoid structure. Unlike 2D monolayer cultures, 3D organoids recapitulate key aspects of tissue architecture, including extracellular matrices, polarity, and multiple cell layers, which can impede compound access to the interior cells. Consequently, effective compound exposure within the organoid core may lag behind peripheral exposure by several hours to days.^59^ This interpretation is consistent with findings reported by Masmoudi and colleagues, where the EC_50_ of antivirals against enterovirus was 10-fold higher in organoid models than in rhabdomyosarcoma cells, indicating a higher barrier for antiviral activity in organoid systems.^60^ We also evaluated the cytotoxicity of compound **1**, compound **3**, and the positive control NITD008 in human brain organoids following 2-day and 4-day treatments at a concentration of 10 μM. As shown in Figure 6B, none of the tested compounds exhibited detectable cytotoxicity at the measured time points, indicating that the antiviral efficacy of compound **1** observed in human brain organoid models is not attributable to compound-induced cytotoxicity at the assessed time points.

**Figure 6:**
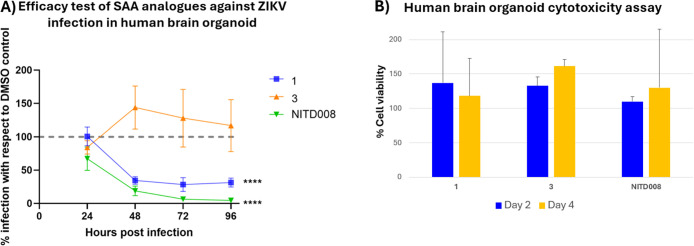
Evaluation of antiviral activity and cytotoxicity of SAA inhibitors in human brain organoid. (A) Antiviral efficacy of compounds **1**, **3** and NITD008 in human brain organoid. Day 60 cortical organoids were infected with ZIKV H/PF/2013 at MOI 1 for 2 h followed by treatment with 5 μM of compound **1**, inactive compound **3** as the negative control and NITD008 as positive control until 96 h postinfection. The kinetics of virus infection at the indicated time points following treatment was determined by qRT-PCR and presented as a percentage of infection relative to the mock DMSO-treated control. (B) Cytotoxicity test of compounds **1**, **3**, and NITD008 in human brain organoid. Dorsal forebrain organoids were treated with the indicated compound at a concentration of 10 μM for 2 and 4 days. Cell viability was assessed by quantifying ATP levels using CellTiter-Glo. Data represent mean ± SD from two organoids.

Nevertheless, our findings demonstrated that human brain organoids can serve as a suitable proof-of-concept model to evaluate antiviral effects of small molecules in a physiologically relevant setting. Importantly, compound **1** retained robust anti-ZIKV activity in this model, suggesting that its unique mechanism of action involving a ribosome-associated pathway is preserved in a complex multicellular model. These findings support the translational potential of SAA inhibitors as antiviral candidates against Flaviviruses and underscore the value of organoid systems for preclinical evaluation of antivirals.

### Design of New SAA Derivatives

The investigation of the mechanism of action of SAA derivatives resulted in the discovery of a complex cellular pathway involving the modulation of ribosomal protein expression, leading to a broad-spectrum antiviral activity observed for these compounds. In light of the encouraging but not yet optimal antiviral activity of initial hits **1** and **2**, and the absence of a definitive identification of the compounds’ exact molecular target, a ligand-based medicinal chemistry strategy was initiated to enhance the potency of the SAA derivatives. Preliminary SAR insights concerning SAA derivatives were obtained from previous work, such as (i) the importance of a lipophilic substituent of the amide, whose disubstitution led to a significant decrease in anti-DENV activity; (ii) the essential role of the quinoline moiety as a sulfonamide substituent, and in particular also of the quinolinic nitrogen; and (iii) the importance of the ether bridge linking the aromatic core to a small lipophilic chain at the C-5 position. However, there was a paucity of detailed information regarding the optimal substituents at the C-5 position, the potential for leveraging H-bonds through the amide substituent, the significance of the acidic proton of the sulfonamide, and the most suitable sulfonamide substituent. Consequently, starting from compounds **1** and **2**, we first designed a series of derivatives (**4**–**16** – Table 1 for chemical structures) bearing different substituents at the C-5 position. Subsequently, leveraging the optimal C-5 substituent, we expanded our design to encompass the incorporation of diverse amide and sulfonamide substituents at the C-1 position, in respect of the phenyl and methylcyclohexyl groups present in **1** and **2**, as well as the methylation of the monosubstituted sulfonamide and its replacement by an amide bond (derivatives **17**–**37** – Tables 2 and 3 for chemical structures).

**Table 2:**
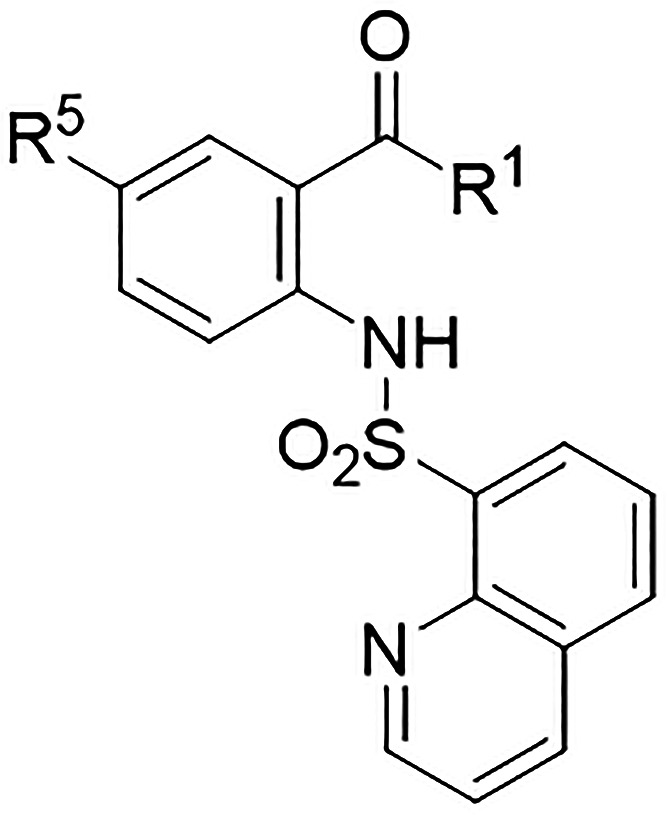

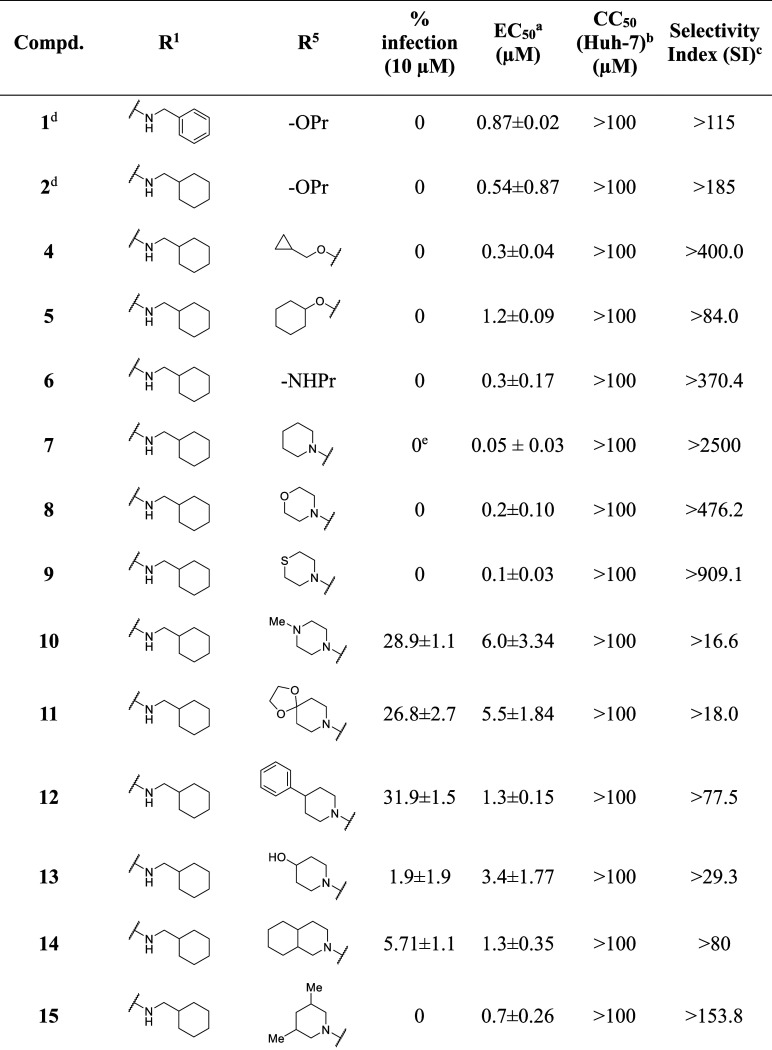

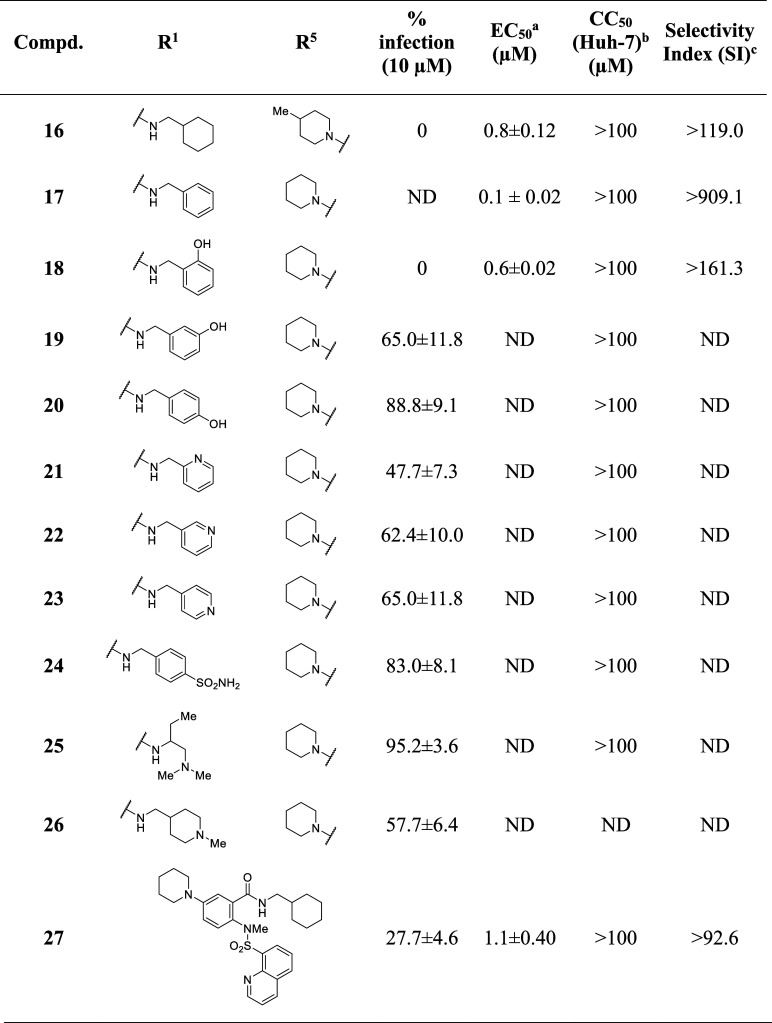
Anti-DENV-2 and Cytotoxicity Evaluation in Huh-7 Cells of Compounds **4–27^f^**

a EC_50_ is the effective concentration that inhibits 50% of virus infection.

b CC_50_ is the cytotoxic concentration that reduces 50% of cell viability.

c SI is the selectivity index determined by CC_50_/EC_50_.

d Starting hit compounds previously published in ref 39.

e The % infection for 10 μM treatment of compound 7 is quoted from the dose–response inhibition data point.

f Data represent mean ± SD from two independent experiments. Abbreviations: ND-Not determined.

**Table 3:**
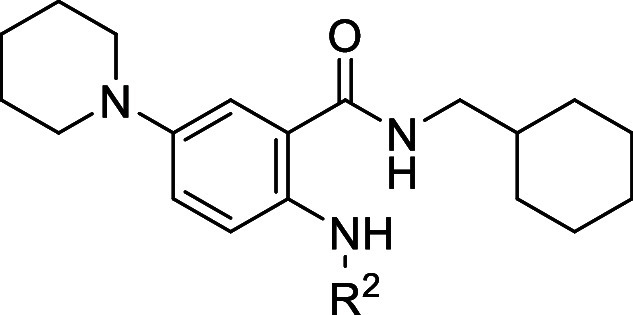

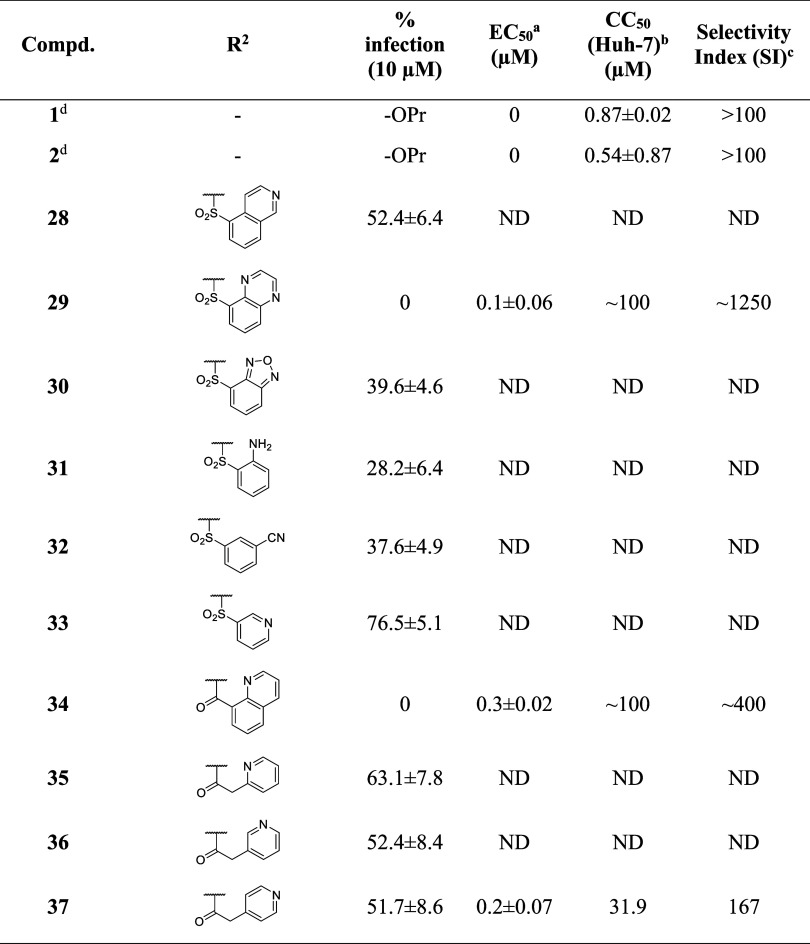
Anti-DENV-2 Evaluation in Huh-7 Cells of Compounds **28–37^e^**

a EC_50_ is the effective concentration that inhibits 50% of virus infection.

b CC_50_ is the cytotoxic concentration that reduces 50% of cell viability.

c SI is the selectivity index determined by CC_50_/EC.

d Starting hit compounds previously published in ref 39.

e Cytotoxicity evaluation on Huh-7 cells of compounds **29**, **34**, and **37**. Data represent mean ± SD from two independent experiments. Abbreviations: ND-Not determined.

### Chemistry

The synthesis of compounds **4–16** and **28–37** is shown in Scheme 1 Ether derivatives **39** and **40** were obtained from commercial phenol **38**, which was reacted with (bromomethyl)­cyclopropane or cyclohexanol tosylate in dry DMF, with K_2_CO_3_ acting as a base, at 70 °C. Subsequent basic hydrolysis in a mixture of aqueous 2 M NaOH and MeOH, after acidic workup, produced the corresponding carboxylic acids, **41** and **42**. These acids, along with the commercially available fluoro derivative, **43**, were first chlorinated with oxalyl chloride in dry CH_2_Cl_2_, catalyzed by a drop of dry DMF. Then, they were immediately reacted with (cyclohexylmethyl)­amine in dry toluene, in the presence of Et_3_N, at room temperature, producing the respective amides, **44**–**46**. The fluorine derivative **46** underwent nucleophilic aromatic substitution with various amines in excess under neat conditions at 70–110 °C or with K_2_CO_3_ in dry DMF at 90 °C to give the corresponding amino derivatives (**47**–**57**). All of the nitro derivatives (**44**, **45**, and **47**–**57**) were then reduced under H_2_ flow and Raney/Ni catalyst at room temperature to produce the C-2 amino derivatives (**58**–**70**). All of the amino derivatives (**58**–**70**) were then reacted with 8-quinolinesulfonyl chloride in dry pyridine at room temperature to give SAA derivatives **4**–**16**. Additionally, amino derivative **61** was reacted with various sulfonyl chlorides in dry pyridine, yielding compounds **28**–**30**, **32**, **33**, and **71**. Subsequently, the nitro group of derivative **71** was catalytically reduced, as already reported above, to afford derivative **31**. In parallel, starting from derivative **61**, an amidation reaction with four different acids, using TBTU as the activating agent and DIPEA as the base in dry DMSO, afforded compounds **34**–**37**.

**Scheme 1:**
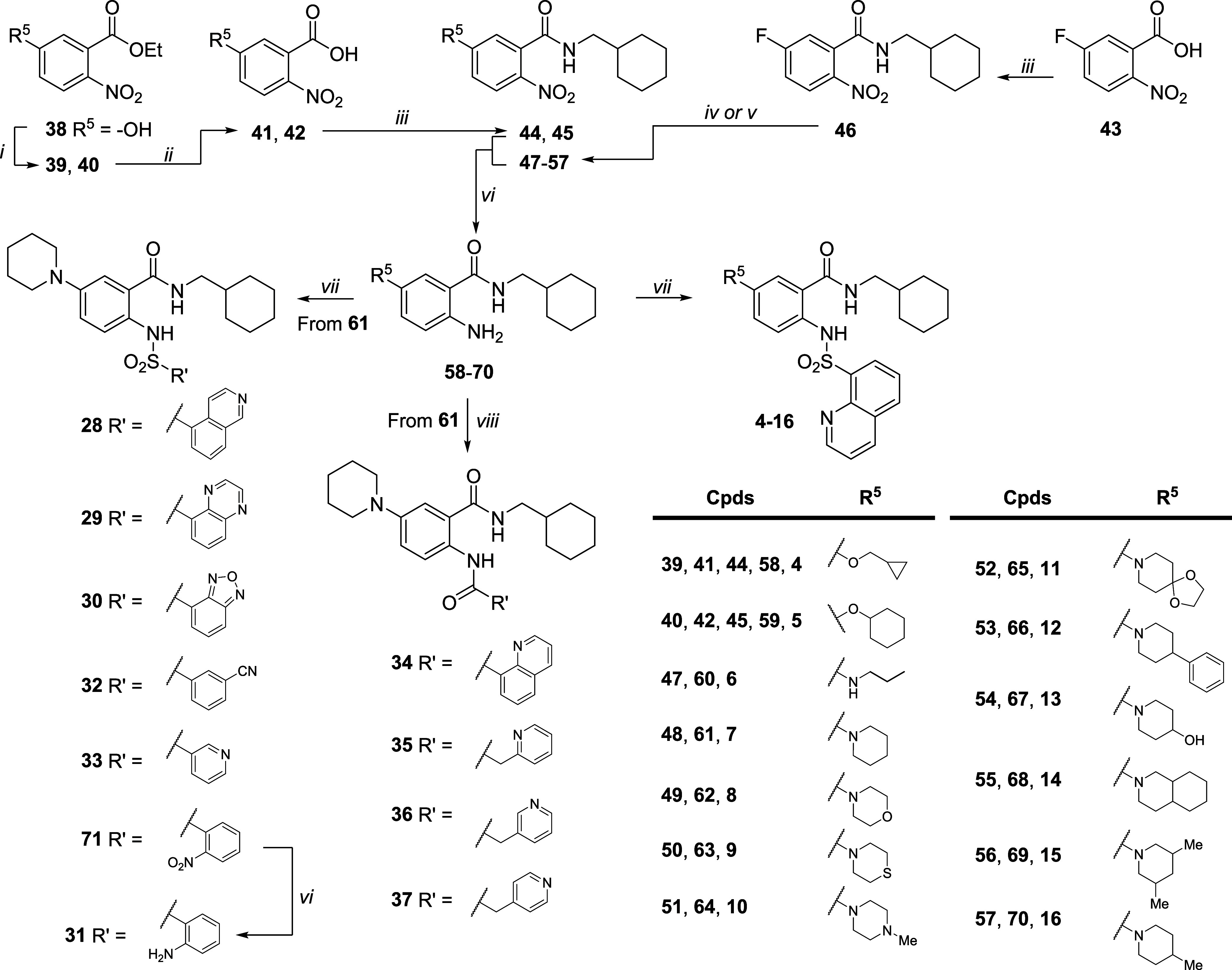
Synthesis of Compounds **4–16** and **28–37** [Fn s1fn1]

a
**Reagents and conditions**: (i) (bromomethyl)­cyclopropane or cyclohexanol tosylate, K_2_CO_3_, dry DMF, 70 °C, 70–86%; (ii) aq. 2 M NaOH, MeOH, 60 °C, 69–82%; (iii) (a) oxalyl chloride, dry DMF, dry CH_2_Cl_2_, rt, (b) (cyclohexylmethyl)­amine, Et_3_N, dry toluene, rt, 78–98%; (iv) appropriate nucleophilic amine, 70–110 °C, 75–98%; (v) appropriate nucleophilic amine, K_2_CO_3_, dry DMF, 90 °C, 78–99%; (vi) Raney/Ni, H_2_, EtOAc or EtOH or MeOH, rt, 43–98%; (vii) properly substituted sulfonyl chloride, dry pyridine, rt, 12–79%; (viii) appropriate carboxylic acid, TBTU, DIPEA, dry DMSO, rt, 30–51%.

The synthesis of compounds **17**–**27** is shown in Scheme 2. The esterification of commercially available acid **43** with EtI in dry DMF, in the presence of K_2_CO_3_, yielded the ethyl ester derivative **72**. Similar to the procedure reported in Scheme 1, the nucleophilic aromatic substitution reaction of **72** with piperidine under neat conditions at 110 °C afforded compound **73**. Then, Raney/Ni-catalyzed reduction of the nitro group of **73** under hydrogen flow in EtOH afforded the amino derivative **74**, which reacted with quinoline-8-sulfonyl chloride in dry pyridine to give the sulfonamide analogue **75**. Mild basic hydrolysis of the ethyl ester of **75** with 1 M LiOH and dioxane afforded the respective acid **76**, which was activated with TBTU in dry DMF in the presence of DIPEA and reacted with differently substituted amines to give compounds **17**–**26**. In parallel, the sulfonamide of derivative **75** was alkylated with MeI in dry DMF, in the presence of K_2_CO_3_, to afford the disubstituted sulfonamide derivative **77**. Basic hydrolysis of the ethyl ester of **77** with 1 M LiOH and dioxane at 70 °C afforded the corresponding acid, **78**, which was amidated with (cyclohexylmethyl)­amine under the previous reaction conditions to give compound **27**.

**Scheme 2:**
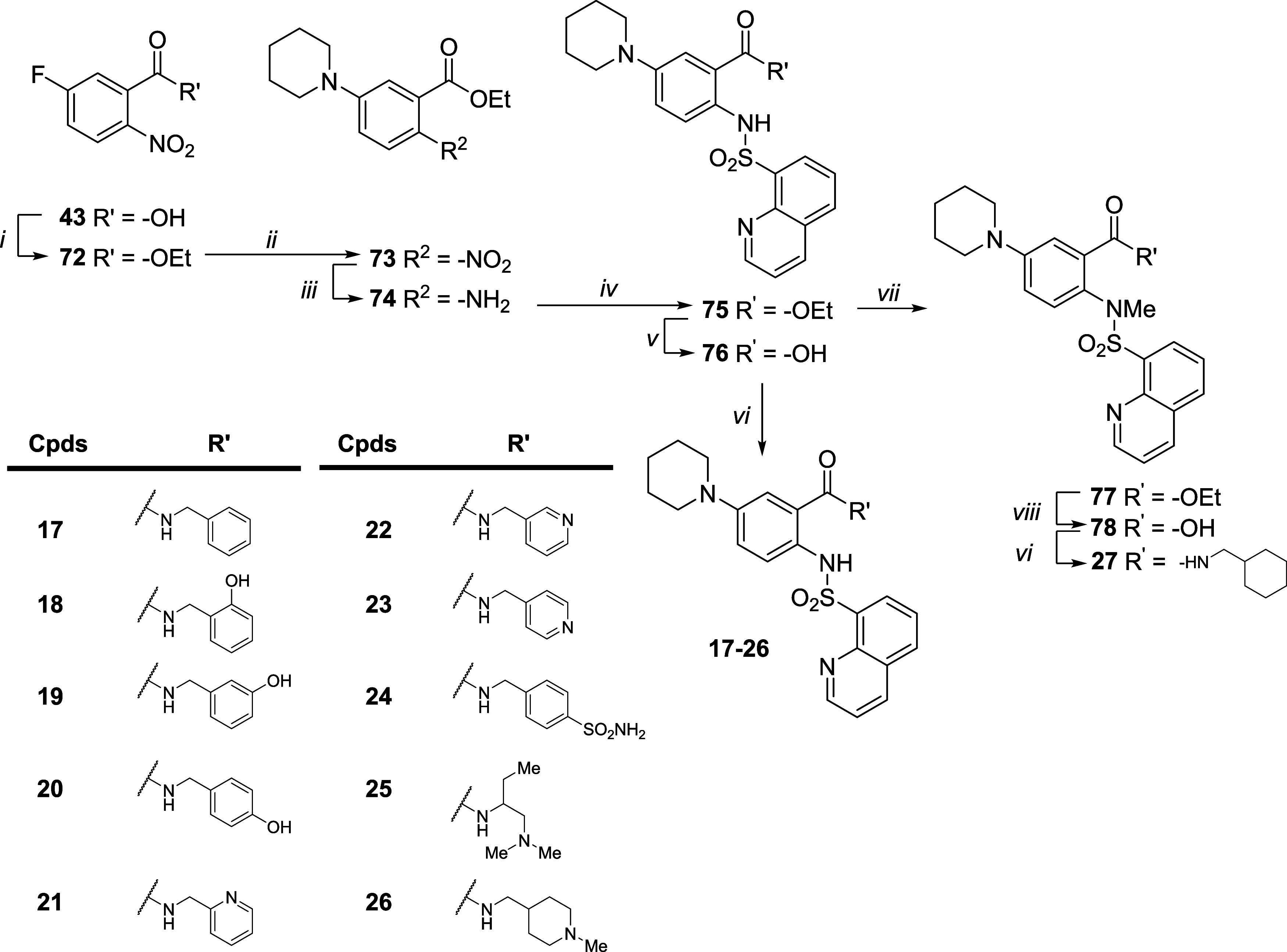
Synthesis of Compounds **17–27** [Fn s2fn1]

a
**Reagents and conditions**: (i) EtI, K_2_CO_3_, dry DMF, rt, 52%; (ii) piperidine, 110 °C, 82%; (iii) Raney/Ni, H_2_, EtOAc, 96%; (iv) 8-quinolinesulfonyl chloride, dry pyridine, rt, 79%; (v) aq. 1 M LiOH, 1,4-dioxane, rt, 83%; (vi) properly substituted amine, TBTU, DIPEA, dry DMF, rt, 11–62%; (vii) MeI, K_2_CO_3_, dry DMF, rt, 52%; (viii) aq. 1 M LiOH, 1,4-dioxane, 70 °C, 53%.

### Anti-DENV-2 Activity of SAA Analogues **4**–**37**

In a preliminary screening, the synthesized compounds were evaluated for their ability to inhibit DENV-2 replication at 10 μM in Huh-7 cells. Subsequently, for compounds that demonstrated at least 70% inhibition of replication, dose-dependent inhibition experiments were conducted to ascertain EC_50_ values. Concurrently, cytotoxicity evaluation was measured by determining CC_50_ values in Huh-7 cells (Tables 2 and 3).

Initially, in order to investigate the size of the small lipophilic ether substituent at the C-5 position, we decided to replace the propyl group of compound **2** with a methylcyclopropyl moiety (compound **4**) and a cyclohexyl ring (compound **5**). It was observed that both compounds did not display cellular toxicity at the highest concentrations that were tested, and they exhibited a significant anti-DENV-2 activity. However, modest variations in potency were observed with respect to compound **2**.^39^ Thus, we decided to investigate the significance of the ether bridge at the C-5 position. To do so, we maintained the propyl group constant and replaced the oxygen with an ″NH″ isosteric group (compound **6**). A marginal enhancement in potency was observed, but this chemical modification showed that the ether bridge is not essential to maintain anti-DENV-2 activity. Furthermore, the presence of a nitrogen atom could be used to introduce different substituents at the C-5 position. A narrow series of heterocycles was indeed selected to be introduced at the C-5 position of the SAA core, leading to the derivatives **7**–**16**. The exploration of different heterocycles resulted in significant changes in the potency of SAA analogues. This provided valuable SAR information, mainly indicating that terminal polar portions of the C-5 substituents are less tolerated than lipophilic ones (Figure 7). Worthy of note, the piperidine analogue **7** showed an impressive EC_50_ value of 50 nM and a SI > 2000, resulting in a more than 1-log potency improvement over the starting hit **2**. Further confirmation that the replacement of the propoxy group with piperidine led to a substantial increase in anti-DENV-2 activity was provided by **17**, a close analogue of **1**. At this point, taking advantage of the significant improvement in anti-DENV-2 potency when the piperidine was placed at C-5 position of the SAA core, we decided to keep it constant and designed some analogues bearing different C-1 amide substituents.

**Figure 7:**
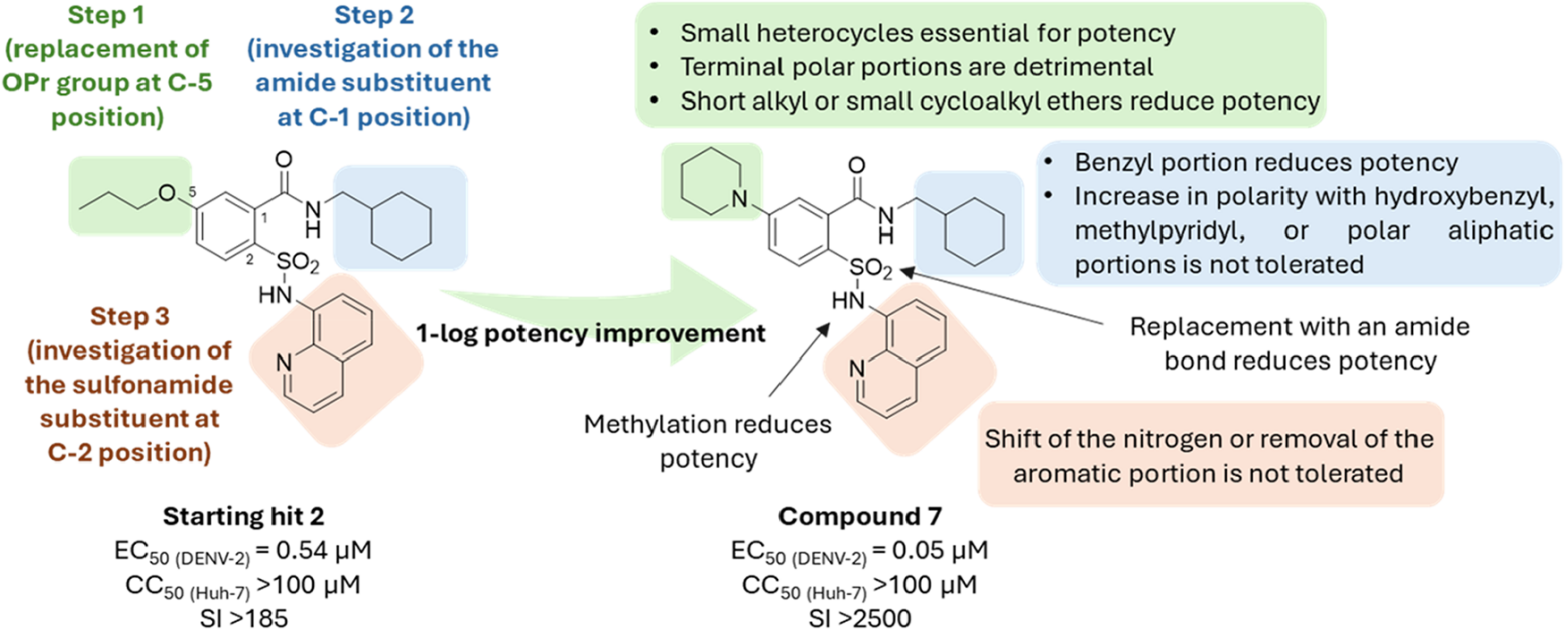
Steps of the design process and SAR highlights.

In this case, the direct modification of the benzyl ring of compound **1** by introducing some hydroxyl groups on the benzyl (**18**–**20**) or replacing the phenyl portion with pyridines (**21**–**23**) was evaluated to explore the possibility of establishing H-bond interaction at this level and also to increase the polarity of the molecule in order to improve water solubility. A substantial decline in the anti-DENV-2 activity was observed for all derivatives, with the exception of compound **18**, which possessed an *ortho* hydroxyl group and exhibited a submicromolar EC_50_ value. These observations suggest that polar groups are not well tolerated at these positions. This finding was further substantiated by the sulfonamide derivative **24**, as well as **25** and **26**, which possessed different polar aliphatic amide substituents.

Subsequently, we investigated the significance of the acidic proton of the C-2 sulfonamide group and devised a direct methylation of the sulfonamide of **7**, culminating in the disubstituted sulfonamide derivative **27**. The anti-DENV-2 activity was retained, indicating that the acidic proton is not essential for target recognition, though it does play a role in potency (EC_50_ from 50 to 1,100 nM).

Consequently, the focus was shifted to the exploration of the role of the quinoline moiety, which had previously been identified as an essential group for the anti-DENV activity (Figure 7). Initially, the investigation focused on determining whether the quinolinic nitrogen could be shifted. To this end, quinoline was substituted with isoquinoline **28**, and the anti-DENV-2 activity was nearly eliminated (48% inhibition of infection at 10 μM). Conversely, the introduction of a second nitrogen atom on the quinoline core of **7**, resulting in the quinazoline derivative **29**, led to a lesser decrease in potency (100 nM vs 50 nM). This reduced activity suggested that a nitrogen atom at this position is not optimal. The replacement of the quinoline of **7** with a benzo­[*c*]­[1,2,5]­oxadiazole **30** also nearly abolished the anti-DENV-2 activity, indicating that an increase in polarity at this level is not tolerated. Given the high lipophilicity of SAA derivatives, an attempt was made to reduce the aromatic portions by designing analogues **31** and **32**, which are closely related to **7**. In these derivatives, the pyridine ring of the quinoline is removed and replaced with an amino group in the *ortho* position (**31**) or a nitrile group in the meta position (**32**), thereby mimicking the quinolinic nitrogen. A marked decline in anti-DENV-2 activity was observed for both derivatives **31** and **32**, underscoring the critical role of both the quinolinic nitrogen, as an H-bond acceptor, and the entire aromatic portion of the quinoline. This conclusion was further substantiated by the pyridine analogue **33**, which, at a concentration of 10 μM, exhibited only a modest inhibition of DENV-2 replication. It was also hypothesized that the pyridine nitrogen of **33** was unlikely to reach the same position as the quinolinic nitrogen of **7**. Therefore, an attempt was made to obtain the SAA derivative with a CH_2_ bridge linking the pyridine to the sulfonamide. Nevertheless, our synthetic endeavors were unsuccessful due to the instability of the sulfonyl chloride necessary for sulfonamide formation. Subsequently, an effort was made to replicate the same approach, but by replacing the sulfonamide with an amide linkage. First, to verify whether an amide could replace the sulfonamide with a similar activity, compound **34** was designed as a close amide analogue of **7**. It was observed that, while **34** demonstrated a reduced potency compared to **7**, it maintained an EC_50_ value of 300 nM. Accordingly, we initiated the design of pyridine-methyl derivatives of **34** with the objective of reducing the aromatic portion, increasing flexibility, and enabling the pyridine nitrogen to reach a position analogous to that of the quinolinic nitrogen. The resulting pyridine analogues **35**–**37** exhibited suboptimal results. Specifically, **35** and **36** exhibited a substantial decline in anti-DENV-2 activity, while compound **37**, which maintained a robust DENV-2 inhibitory effect, demonstrated an increase in cellular toxicity (CC_50_ of 31.9 μM). The available data indicated that sulfonamide could be substituted for an amide bond without a substantial loss in potency. In parallel, the data also suggested that the aromatic portion of the quinoline may be essential for target recognition, thus imparting potency and selectivity.

### Anti-pan-DENV and Broad-Spectrum Antiviral Activity Evaluation of Compound **7**

The strong anti-DENV-2 efficacy of compound **7** prompted us to evaluate its pan-serotype anti-DENV activity in Huh-7 cells. As shown in Figure 8, compound **7** exhibited improved potency against all DENV serotypes compared to compound **1** and compound **2** (Table 1).^39^ The inactive compound **3** was included as a negative control (Figure S4). Furthermore, when tested against two other RNA viruses, in particular the flavivirus ZIKV and the enterovirus EV71, compound **7** displayed nanomolar EC_50_ values, confirming its potent efficacy against different RNA viruses and the potential for broad-spectrum antiviral activity.

**Figure 8:**
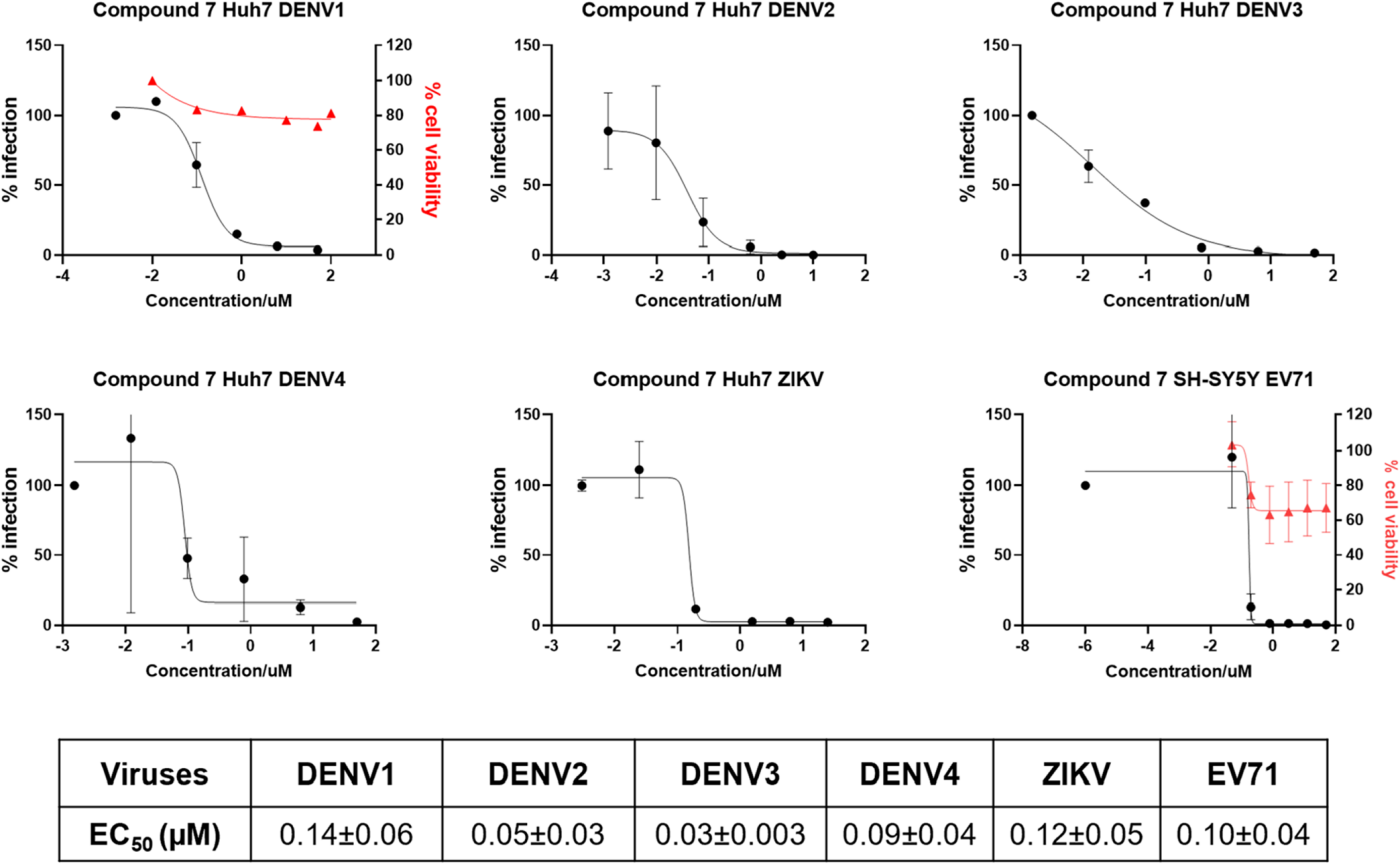
Dose–response viral inhibition of 7 against four dengue serotypes and other RNA viruses. Inhibitory effect of compound **7** against DENV-1–4, ZIKV in Huh-7 cells, and EV71 in SH-SY5Y cells. The EC_50_ values of compound **7** against the tested viruses are indicated in the inset table. Data for DENV-2 represent the mean ± SD from four independent experiments. Data for EV71 represent mean ± SD from two independent experiments. Data for DENV-1, DENV-3, DENV-4, and ZIKV represent the mean ± SEM from technical duplicates in a single experiment.

### Effect of SAA Derivatives on 5′-TOP-Driven Translation

During the hit optimization process of SAA derivatives, several attempts were made to shed more light on the pathway underlying the mechanism of action of these analogues. In the course of these experiments, we made the puzzling observation that the treatment with SAA inhibitors did not affect the transient ectopic expression of CMV promoter-driven NS5 protein while showing significant reduction in the EF1α promoter-driven NS5 expression level upon treatment (Figure 9A). Subsequent sequence analysis of the two promoter regions revealed the presence of 5′ Terminal Oligopyrimidine (5′-TOP) motif within the EF1α-promoter, suggesting that this element could represent a potential target of SAA inhibitors.

**Figure 9:**
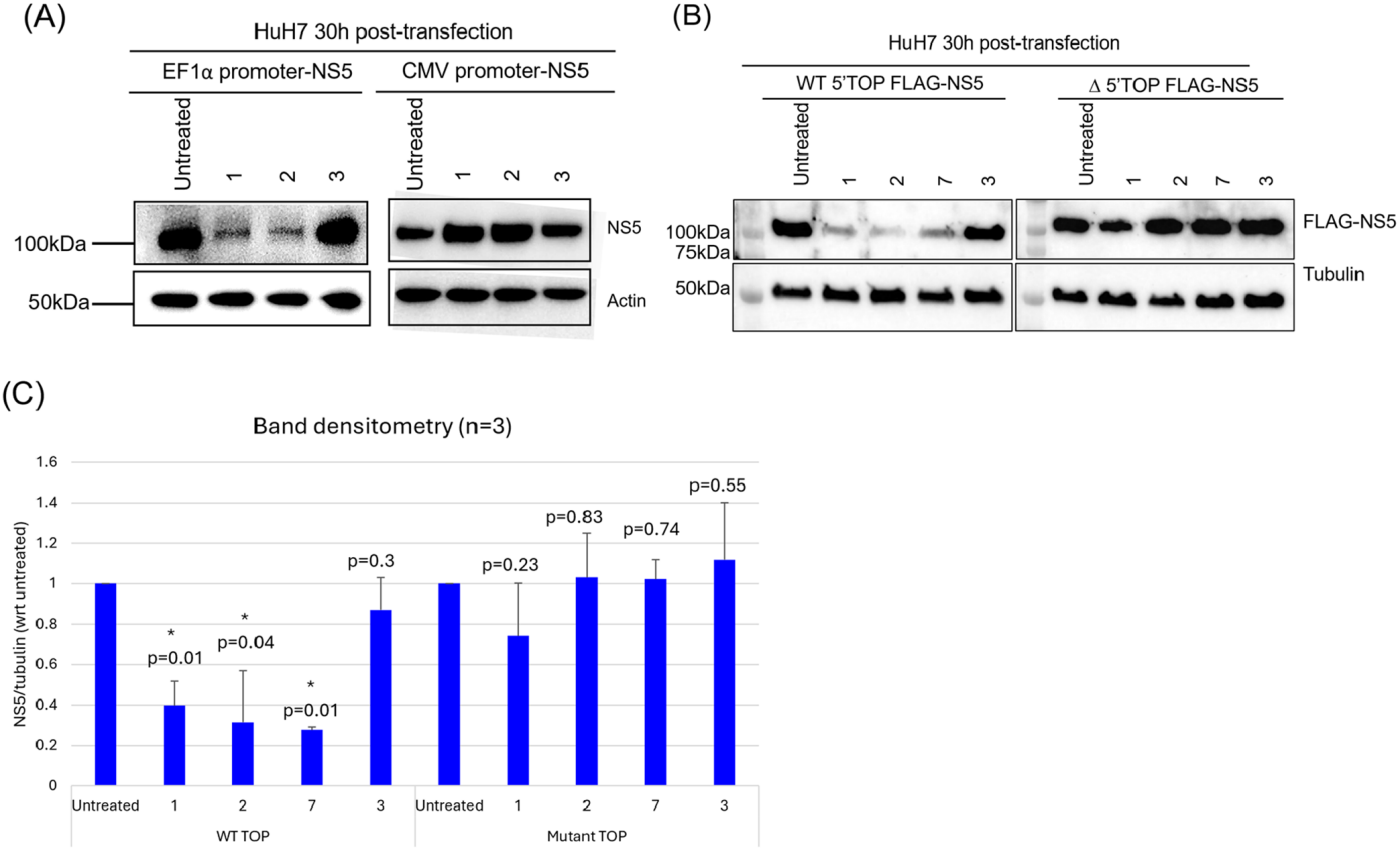
Transfection reporter assay for target deconvolution. (A) Impact of SAA compounds on CMV-and EF1α-promoter-driven NS5. Huh-7 cells were transfected with EF1α- promoter NS5 and CMV promoter NS5 plasmids. Six h post-transfection, the cells were treated with compounds **1**–**3** at 10 μM and further incubated for 24 h. Cells were lysed, and 10 μg of the total lysate was analyzed by Western blot using in-house 5M1 IgG.^69^ Actin was included as the loading control. (B) Effect of SAA inhibitors on plasmid carrying C > G mutation in EF1α promoter. Huh-7 cells were transfected with wild-type 5′-TOP-NS5 and mutant 5′-TOP-NS5 plasmids. At 6-h post-transfection, the cells were treated with compounds **1**, **2**, **7**, and **3** at a concentration of 10 μM. The samples were harvested at 24 h post-treatment and subjected to Western blot analysis. Tubulin was included as the loading control. (C) Band densitometry of wild-type 5′-TOP NS5 and mutant 5′-TOP NS5 expression following treatment with SAA inhibitors, as shown in (B). Data represent the mean ± SD from three independent experiment. A p-value less than 0.05 was considered significant (*, *p* < 0.05).

The 5′-TOP motif is a conserved sequence element found in all the mRNAs encoding translation-related proteins, including ribosomal proteins and elongation factors (such as EF1α), and serves as the key regulatory element for the translation of these TOP-motif-containing mRNAs.^61,62^ Accordingly, the perturbation of the expression of specific ribosomal proteins observed at the cellular level in response to treatment with SAA derivatives could be mediated by their potential modulation of mRNAs containing the 5′-TOP motif. Of note, the first pyrimidine within the 5′-TOP motif is critical for translation repression of TOP mRNAs, a process regulated through the binding of LARP1 protein to the 5′-TOP motif.^63^ A single nucleotide substitution of the first cytidine (5′-C) to guanine (5′-G) within the 5′-TOP motif significantly weakens this interaction.^64^

To validate the impact of SAA derivatives on the 5′-TOP motif, we introduced the same substitution (5′-C to 5′-G) into our EF1α-promoter-driven NS5 construct (Figure 9B,C). Our results demonstrated that SAA compounds **1**, **2**, and **7** significantly reduced the ectopic Flag-NS5 protein expression in EF1α promoter containing the wild-type 5′-TOP motif but had no effect on the Flag-NS5 expression from the mutant 5′-TOP motif driven EF1α promoter. A similar finding was observed when compounds were treated in the cells transfected with host cleavage and polyadenylation specific factor 3 (CPSF3) plasmid driven by EF1α-promoter (Figure S5), implying that the effect of the compound is not viral protein specific. In contrast, the inactive compound **3** had no inhibitory effect against all the constructs. Together, these findings suggest that SAA compounds specifically target 5′-TOP motifs within the EF1α-promoter, thereby affecting protein expression. This observation aligns with our global proteomic analysis, in which compound **1** treatment resulted in downregulation of ribosomal proteins, whose mRNAs contain 5′-TOP motifs.

5′-TOP mRNA translation is commonly regulated through the mTOR-LARP1 signaling pathway.^65^ However, although we showed that SAA compounds selectively modulate 5′-TOP-dependent translation, they did not reduce the expression of downstream substrates including p-mTOR, p-70s6k, and p-eIF4g in both noninfected and infected cells, unlike the effects observed in rapamycin-treated samples (Figure S6). These findings indicate that the activity of SAA is unlikely to involve direct inhibition of mTOR signaling. Instead, SAA compounds may regulate 5′-TOP mRNA translation through alternative mechanisms that remain to be defined. Several proteins have been reported to bind and regulate the 5′-TOP motifs including Cellular Nucleic Acid Binding Protein (CNBP), T-Cell Intracellular Antigen (TIA1) and TIA-Related Protein (TIAR) and ARE/poly­(U)-Binding/Degradation Factor 1­(AUF1), and may also represent potential targets of SAA.^61^

Collectively, these data suggest that SAA derivatives **1**, **2**, and **7** selectively modulate translation of 5′-TOP mRNAs, likely through direct or indirect interactions with the 5′-TOP motifs. In a direct binding scenario, SAA compounds may bind directly to the 5′-TOP motif, potentially interfering with the recruitment of translation initiation factors such as eIF4E and thereby disrupting 5′-TOP mRNA translation. Alternatively, SAA compounds may act indirectly by interacting with an unidentified regulatory protein that regulates the 5′-TOP motif. Mechanistically, the mode of action of SAA compounds is distinct from that of classical translation inhibitors (e.g., cycloheximide and plitidepsin) and other host-directed antivirals (e.g., silvestrol and elisabatin),^66–68^ which directly interfere with translation elongation, leading to global suppression of protein synthesis and are associated with cellular toxicity. In contrast, SAA compounds do not impair global translation but selectively modulate the expression of a subset of ribosomal proteins through the 5′TOP-driven translational pathway. By partially and selectively perturbing ribosome-associated functions essential for viral replication, SAA compounds represent a mechanistically distinct host-directed antiviral strategy with the potential to preserve essential host translational capacity.

### Preliminary *In Vitro* ADME Properties Evaluation of Compounds **1** and **7**

Given the encouraging antiviral activity observed for compound **7**, as well as its novel mechanism of action, we were interested in determining whether it also possessed favorable *in vitro* ADME properties. Therefore, a preliminary *in vitro* ADME properties assessment was performed, involving the evaluation of passive membrane permeability, plasma stability, and human liver metabolic stability. The starting hit compound **1** was included for comparison as a representative of the first series of SAA analogues, as well as because its structure is significantly different from that of optimized compound **7** (Table 4).

**Table 4:** Preliminary *In Vitro* ADME Profile of Compounds **1** and **7**

compd.	kinetic solubility (μM)^a^	*P*_app_ (10^‑6^ cm/s)^b^	plasma stability^c^		human liver microsomal stability	
			1 h	2 h	*In vitro* *t*_1/2_ [min]^d^	CL_int_ in vivo [mL min^–1^ kg^1–^]^e^
**1**	3.4	u.d.l.^f^	102.1% ± 2.9%	104.1% ± 5.1%	16.6	67.5
**7**	10.6	2.93 ± 0.34	98.6% ± 0.2%	96.9% ± 1.5%	59.8	18.8

a Determined in phosphate-buffered saline (PBS) (pH 7.4) with 1% DMSO at 25 °C after 24 h.

b Compounds were tested at 250 μM. Values for propranolol, furosemide and caffeine are consistent with literature data.

c (%) 100 – [concentration at time points min/concentration at 0 min] × 100.

d 0.693/*k*, where *k* is the slope of linear regression of the percentage parent compound remaining against time.

e CL_int *in vitro*_ × (mg microsome g 1 liver) × [liver mass (g)/body mass (kg)].

f Under detection limit.

The determination of kinetic solubility was conducted by employing the shake-flask method in a solution of PBS_7.4_ (1% DMSO). Compound **1** exhibited minimal solubility (3.4 μM), while compound **7** demonstrated a 3-fold improvement, surpassing the critical 10 μM benchmark.

Membrane permeability was evaluated using the well-established Parallel Artificial Membrane Permeability Assay (PAMPA), with propranolol (*P*_app_ of 14.10 × 10^–6^), caffeine (*P*_app_ of 2.49 × 10^–6^), and furosemide (under detection limit) serving as the positive, medium, and negative controls, respectively.^70–72^ The apparent permeability coefficient (*P*_app_) was calculated using Faller’s modification of the Sugano equation.^73^ Notably, compound **7** demonstrated an acceptable apparent permeability value (*P*_app_) of 2.93 × 10^–6^ cm/s. While this value was not yet optimal, it was significantly better than that of compound **1**, which proved to be poor in terms of permeability. Subsequently, the human plasma stability of compounds **1** and **7** was also evaluated. Notably, both compounds were recovered unchanged after incubation for up to 120 min, indicating excellent stability under plasma conditions.

Finally, the liver metabolic stability of compounds **1** and **7** was evaluated in human liver microsomes (HLMs). Microsomal incubations were performed in the presence of NADPH to monitor the disappearance of parent compounds and the formation of metabolites over time (Figure S7). As shown in Figure S7A, compound **1** was metabolized more rapidly than compound **7**, with a shorter half-life (*t*_1/2_ = 16.6 min) and higher intrinsic clearance (CL_int_ = 67.5 mL·min^–1^·kg^–1^), consistent with its lower metabolic stability. In contrast, compound **7** exhibited a slower metabolic turnover (*t*_1/2_ = 59.8 min; CL_int_ = 18.8 mL·min^–1^·kg^–1^) under the same experimental conditions. The negative control, obtained from parallel incubations without NADPH, confirmed that the tested compounds undergo NADPH-dependent oxidative pathways. Mass spectrometry analysis of metabolites generated during HLM incubation of compound **1** (Figure S7B) revealed that the main metabolite (**M7–1**, Figure S8G) was produced through mono-oxidation of the quinoline moiety. Several minor metabolites were also detected, including dioxidation products involving both the quinoline and benzyl groups (**M2–1**, Figure S8B), dealkylation of the aliphatic side chain (**M1–1**, Figure S8A; **M3–1**, Figure S8C), and oxidative desaturation (**M6–1**, Figure S8F; **M8–1**, Figure S8H). LC-MS/MS analysis revealed that the major metabolite of compound **7** (Figure S7C) was an oxidative product (**M8–7**), exhibiting a protonated molecular ion [M + H]^+^ at *m*/*z* 523.2365, consistent with a + 16 Da mass shift relative to the parent compound. MS/MS fragmentation spectra indicated mono-oxidation of the piperidine moiety (Figure S9H). Additional mono-oxidized products were observed (Figure S7C). LC-MS/MS analysis of metabolites **M3-**, **M4-**, and **M5–7** suggested oxidation on the cyclohexane moiety (Figure S9C–E). Minor dioxidated metabolites (**M1-M2–7**, Figure S9A,B) resulted from oxidation of the piperidine and cyclohexane groups, while **M6–7** (Figure S9F) showed oxidation on the piperidine moiety and an additional oxidation on the quinoline ring. In summary, the metabolic stability assessment showed that compound **7** was more stable than the starting hit **1** in HLM. This was evident from the significant increase in *t*_1/2_, which for compound **7** approached almost an hour. To note, metabolite analysis revealed key information that will inform future optimizations. Indeed, targeted modifications could be made to the piperidine of compound **7**, such as introducing difluorination to limit metabolic oxidation. The presence of fluoro substituents should be compatible with the observed SAR, which indicates that small heterocycles lacking polar terminal moieties are well tolerated at this position. In terms of increasing polarity and solubility, compound **7** represents a step forward from the starting hit compound **1**, though it is still suboptimal. The SAR clearly shows that the C-5 group and the amide substituent at C-1 cannot be exploited to introduce polar groups. However, there is room to further explore the sulfonamide substituent at C-2. The fact that replacing the sulfonamide bond with an amide bond is tolerated could be exploited to introduce a wider range of modifications to the quinoline nucleus more easily, with the aim of increasing polarity on this portion of the molecule.

## Conclusions

In this paper, we report on our efforts in the identification of the mode of action and the medicinal chemistry optimization of SAA derivatives. First, target deconvolution was performed using compound **1**, which revealed modulation of ribosomal protein expression as the antiviral mechanism. Several of these proteins were validated by siRNA knockdown and shown to contribute to DENV replication. It is noteworthy that this antiviral mechanism conferred broad-spectrum activity, with compounds **1** and **2** that retained submicromolar potency against multiple flaviviruses (ZIKV, YFV, JEV, WNV) and other RNA virus families (Alphavirus, Enterovirus, Coronavirus). Furthermore, compound **1** demonstrated its efficacy in a brain organoid model infected with ZIKV, resulting in a ∼70% reduction in ZIKV infection, comparable to the direct-acting antiviral NITD008. This finding suggests that SAA derivatives retained antiviral activity in a more complex and physiologically relevant environment. Encouraged by these results, we pursued a medicinal chemistry optimization strategy despite the lack of a defined molecular target that would permit the implementation of a target-based approach. These efforts culminated in the design and synthesis of 34 novel SAA derivatives, which were subsequently tested against DENV-2. The outcomes of the study served two main purposes: (i) to expand the SAR around this novel antiviral scaffold, significantly enriching the available data set, and (ii) to enable the identification of compound **7**, which exhibited a substantial enhancement in anti-DENV-2 activity. Compound **7** demonstrated an EC_50_ value of 50 nM and a SI > 2000, signifying a more than 10-fold improvement in potency compared to the starting hits **1** and **2**. Importantly, compound **7** exhibited pan-serotype anti-DENV activity and demonstrated broad-spectrum potential, maintaining nanomolar activity against both ZIKV and enterovirus.

Subsequent refinement of the mechanism of action indicated that SAA derivatives may directly or indirectly modulate the 5′-TOP motif present on mRNAs encoding proteins essential for translation. This partial and selective modulation of ribosomal protein expression through 5′-TOP-driven translation control may preferentially impair RNA or DNA viral replication, which often relies on specific ribosomal proteins for its survival. By selectively modulating host translational machinery rather than globally inhibiting protein synthesis, this approach may therefore enable broad-spectrum antiviral activity while preserving essential host functions and maintaining a high barrier to the emergence of viral resistance. Nevertheless, further biophysical investigations will be required to delineate the precise interaction between SAA derivatives and 5′-TOP-containing mRNAs.

In summary, the present study identified an antiviral compound with low-nanomolar activity that functions through a novel host-targeting mechanism of action. Moreover, the newly developed SAA derivative **7** demonstrated favorable preliminary *in vitro* ADME properties, exhibiting acceptable permeability along with good liver microsome and plasma stability. Therefore, the potency observed against diverse RNA viruses provides a strong foundation for future development of host-targeting antivirals with broad-spectrum potential. Importantly, the demonstration that these compounds can be optimized for potency while maintaining low host toxicity highlighted the viability of this pathway for the development of next-generation antiviral agents.

## Experimental Section

### Chemistry

Unless stated otherwise, all starting materials were obtained from commercial suppliers. Reagents and solvents were purchased from commercial suppliers and utilized in their original received state. The organic solutions were dried over anhydrous Na_2_SO_4_ and concentrated with an IKA RV 8 V rotary evaporator equipped with a vacuum pump (IKA VACSTAR digital) under reduced pressure. All reactions were routinely checked by TLC on silica gel 60_F254_ (Merck) and visualized by using UV and iodine. The purifications were conducted via flash column chromatography separations employing Merck silica gel 60 (mesh 230–400). The yields refer to purified products and are not optimized. ^1^H NMR and ^13^C NMR spectra were recorded at 400 and 100 MHz, respectively, with a Bruker Advance DRX-400 instrument. The chemical shifts (δ) are reported in ppm relative to TMS and calibrated using residual undeuterated solvent as an internal reference. The coupling constants (*J*) are reported in Hz. The spectra were acquired at 298 K, and the subsequent data processing was performed utilizing Bruker TopSpin 4.5.0 software. The resultant spectral data were found to be consistent with the assigned structures. The purity (>95%) was revealed at 254 nm through HPLC analysis using a Jasco LC-4000 instrument equipped with a UV–visible Diode Array Jasco MD-4015 and C18 Column (Gemini, Phenomenex), 3 μm, 2 mm × 100 mm. The HPLC methods used were method A, method B, or the method specified for each compound. Method A involves a gradient consisting of acetonitrile (ACN) and water containing 0.1% formic acid, with a linear increase of ACN from 20% to 100% over 10 min, followed by 100% ACN; flow: 0.5 mL/min. Method B involves a gradient consisting of ACN and water containing 0.1% formic acid, with a linear increase of ACN from 10% to 100% over 15 min, followed by 100% ACN; flow: 0.4 mL/min. The resulting chromatograms were then analyzed using the ChromNAV 2.0 Chromatography Data System software. The retention time of each peak is expressed in minutes. The detection of HRMS was based on electrospray ionization (ESI) in positive or negative polarity, as indicated for each compound, using a QTOF Ion Mobility Agilent 6560 equipped with U­(H)­PLC 1290 Infinity II.

#### Ethyl 5-(Cyclopropylmethoxy)-2-nitrobenzoate (**39**)

Under N_2_ atmosphere, to a solution of compound **38** (1.50 g, 7.00 mmol) in dry DMF (8 mL), (bromomethyl)­cyclopropane (1.38 mL, 14.00 mmol) and K_2_CO_3_ (2.94 g, 21.00 mmol) were added. After stirring at 70 °C for 3 h, the reaction mixture was poured into ice/water and extracted with EtOAc (×3). The combined organic layers were washed with brine, dried over Na_2_SO_4_, and evaporated under vacuum to give a yellow oil in 86% yield (1.60 g). ^1^H NMR (400 MHz, CDCl_3_): δ = 8.03 (d, *J* = 9.0 Hz, 1H, H-3), 7.03 (d, *J* = 2.6 Hz, 1H, H-6), 7.00 (dd, *J* = 2.8 and 9.0 Hz, 1H, H-4), 4.40 (q, *J* = 7.2 Hz, 2H, O*CH*_2_CH_3_), 3.91 (d, *J* = 7.0 Hz, 2H, OCH_2_), 1.36 (t, *J* = 7.2 Hz, 3H, OCH_2_ *CH*_3_), 1.33–1.23 (m, 1H, cyclopropyl-CH), 0.72–0.66 (m, 2H, cyclopropyl-CH_2_), 0.40–0.35 (m, 2H, cyclopropyl-CH_2_).

#### Ethyl 5-(Cyclohexyloxy)-2-nitrobenzoate (**40**)

Following the same procedure as for **39** and replacing the (bromomethyl)­cyclopropane with cyclohexanol tosylate, compound **40** was obtained, after stirring at 70 °C for 14 h, as a yellow oil in 70% yield (0.92 g). ^1^H NMR (400 MHz, CDCl_3_): δ = 8.02 (d, *J* = 9.0 Hz, 1H, H-3), 7.01 (d, *J* = 2.7 Hz, 1H, H-6), 6.98 (dd, *J* = 2.7 and 9.0 Hz, 1H, H-4), 4.43–4.36 (m, 3H, cyclohexyl-OCH and O*CH*_2_CH_3_), 2.02–1.94 (m, 2H, cyclohexyl-CH_2_), 1.85–1.77 (m, 2H, cyclohexyl-CH_2_), 1.63–1.56 (m, 4H, cyclohexyl-CH_2_ × 2), 1.46–1.39 (m, 2H, cyclohexyl-CH_2_), 1.37 (t, *J* = 7.2 Hz, 3H, OCH_2_ *CH*_3_).

#### 5-(Cyclopropylmethoxy)-2-nitrobenzoic Acid (**41**)

To a solution of derivative **39** (1.60 g, 5.98 mmol) in MeOH (9.20 mL), aq. 2 M NaOH (9.20 mL, 18.40 mmol) was added. After stirring at 60 °C for 2 h, the reaction mixture was poured into ice/water and acidified with 2 M HCl (pH = 3) to give a precipitate that was filtered under vacuum to obtain compound **41** as a solid in 82% yield (1.30 g). ^1^H NMR (400 MHz, CDCl_3_): δ = 9.10 (bs, 1H, CO_2_H), 8.02 (d, *J* = 9.1 Hz, 1H, H-3), 7.18 (d, *J* = 2.7 Hz, 1H, H-6), 7.07 (dd, *J* = 2.7 and 9.1 Hz, 1H, H-4), 3.94 (d, *J* = 7.0 Hz, 2H, OCH_2_), 1.36–1.26 (m, 1H, cyclopropyl-CH), 0.74–0.67 (m, 2H, cyclopropyl-CH_2_), 0.43–0.37 (m, 2H, cyclopropyl-CH_2_).

#### 5-(Cyclohexyloxy)-2-nitrobenzoic Acid (**42**)

Following the same procedure as for **41**, starting from derivative **40** (0.92 g, 3.14 mmol), after stirring at 60 °C for 12 h, compound **42** was obtained, after extraction with EtOAc (x3), as a pink solid in 69% yield (0.58 g). ^1^H NMR (400 MHz, CDCl_3_): δ = 7.95 (d, *J* = 9.1 Hz, 1H, H-3), 7.08 (d, *J* = 2.7 Hz, 1H, H-6), 6.96 (dd, *J* = 2.7 and 9.4 Hz, 1H, H-4), 4.38–4.31 (m, 1H, cyclohexyl-OCH), 1.97–1.88 (m, 2H, cyclohexyl-CH_2_), 1.80–1.71 (m, 2H, cyclohexyl-CH_2_), 1.58–1.47 (m, 3H, cyclohexyl-CH_2_ and CH_2_ × 1/2), 1.41–1.20 (m, 3H, cyclohexyl-CH_2_ and CH_2_ × 1/2).

### General Procedure for Amidation Reaction (Method A)

Under N_2_ atmosphere, to a solution of the appropriate benzoic acid derivative (1.00 equiv) in dry CH_2_Cl_2_ (5 mL per mmol), oxalyl chloride (1.20–1.60 equiv) and two drops of dry DMF were added. After stirring at rt for 2–5 h, the solvents were evaporated under vacuum to give the corresponding acyl chloride as a yellow oil that was dissolved in dry toluene (10 mL per mmol) and added dropwise to a solution of (cyclohexylmethyl)­amine (1.30 equiv) and Et_3_N (1.50 equiv) in dry toluene (5 mL per mmol). After stirring at rt for 1–14 h, the reaction mixture was poured into ice/water. The mixture was either filtered (if a precipitate formed) or extracted with EtOAc (×3), and the combined organic layers were washed with brine, dried over Na_2_SO_4_, and evaporated to dryness. The corresponding benzamide derivative was used in the next reaction without further purification.

#### *N*-(Cyclohexylmethyl)-5-(cyclopropylmethoxy)-2-nitrobenzamide (**44**)

Following the general procedure (method A), starting from derivative **41** and using 1.20 equiv of oxalyl chloride, after stirring for 3 h, the corresponding acyl chloride was obtained. Then, after stirring for 2 h, compound **44** was obtained after filtration as a yellow solid in 98% yield (1.64 g). ^1^H NMR (400 MHz, CDCl_3_): δ = 8.11 (d, *J* = 9.1 Hz, 1H, H-3), 6.97 (dd, *J* = 2.8 and 9.1 Hz, 1H, H-4), 6.92 (d, *J* = 2.7 Hz, 1H, H-6), 5.74 (bs, 1H, CONH), 3.90 (d, *J* = 7.0 Hz, 2H, OCH_2_), 3.31 (t, *J* = 6.4 Hz, 2H, NCH_2_), 1.84–1.58 (m, 6H, cyclohexyl-CH_2_ × 3), 1.34–1.12 (m, 4H, cyclohexyl-CH_2_ × 2), 1.06–0.94 (m, 2H, cyclopropyl-CH and cyclohexyl-CH), 0.72–0.66 (m, 2H, cyclopropyl-CH_2_), 0.40–0.35 (m, 2H, cyclopropyl-CH_2_).

#### *N*-(Cyclohexylmethyl)-5-(cyclohexyloxy)-2-nitrobenzamide (**45**)

Following the general procedure (method A), starting from derivative **42** and using 1.20 equiv of oxalyl chloride, after stirring for 3 h, the corresponding acyl chloride was obtained. Then, after stirring for 14 h, compound **45** was obtained after filtration as a yellow solid in 78% yield (0.53 g). ^1^H NMR (400 MHz, CDCl_3_): δ = 8.03 (d, *J* = 9.1 Hz, 1H, H-3), 6.86 (dd, *J* = 2.6 and 9.2 Hz, 1H, H-4), 6.82 (d, *J* = 2.4 Hz, 1H, H-6), 5.68 (bs, 1H, CONH), 4.35–4.27 (m, 1H, cyclohexyl-OCH), 3.24 (t, *J* = 6.4 Hz, 2H, NCH_2_), 1.95–1.86 (m, 2H, cyclohexyl-CH_2_), 1.78–1.43 (m, 10H, cyclohexyl-CH_2_ × 5), 1.38–1.05 (m, 7H, cyclohexyl-CH_2_ × 3 and CH), 0.99–0.87 (m, 2H, cyclohexyl-CH_2_).

#### *N*-(Cyclohexylmethyl)-5-fluoro-2-nitrobenzamide (**46**)

Following the general procedure (method A), starting from compound **43** and using 1.60 equiv of oxalyl chloride, after stirring for 5 h, the corresponding acyl chloride was obtained. Then, after stirring for 1 h, compound **46** was obtained after extraction as a yellow solid in 78% yield (1.17 g). ^1^H NMR (400 MHz, CDCl_3_): δ = 8.14 (dd, *J* = 4.7 and 9.0 Hz, 1H, Ar–H), 7.26–7.23 (m, 1H, Ar–H), 7.22–7.18 (m, 1H, Ar–H), 5.83 (bs, 1H, CONH), 3.31 (t, *J* = 6.5 Hz, 2H, NCH_2_), 1.84–1.72 (m, 4H, cyclohexyl-CH_2_ × 2), 1.72–1.53 (m, 2H, cyclohexyl-CH_2_), 1.35–1.12 (m, 3H, cyclohexyl-CH_2_ and CH), 1.07–0.95 (m, 2H, cyclohexyl-CH_2_).

### General Procedure for Aromatic Nucleophilic Substitution Reaction (Method B)

To the appropriate fluoro derivative (1.00 equiv), an appropriate nucleophilic amine (4.00–12.00 equiv) was added. After stirring at 70–110 °C for 1–2 h, the reaction mixture was poured into ice/water. The mixture was either filtered (if a precipitate formed) or extracted with EtOAc (x3), and the combined organic layers were washed with brine, dried over Na_2_SO_4_ and evaporated to dryness. The corresponding substituted derivative was used in the next step without further purification.

#### *N*-(Cyclohexylmethyl)-2-nitro-5-(propylamino)­benzamide (**47**)

Following the general procedure (method B), starting from derivative **46** and using 4.00 equiv of propylamine, after stirring at 70 °C for 2 h, compound **47** was obtained after extraction as a yellow solid in 90% yield (1.18 g). ^1^H NMR (400 MHz, DMSO-*d*_6_): δ = 8.36 (t, *J* = 5.8 Hz, 1H, CONH), 8.00 (d, *J* = 9.2 Hz, 1H, H-3), 7.36 (t, *J* = 5.4 Hz, 1H, *NH*CH_2_CH_2_CH_3_), 6.68 (dd, *J* = 2.6 and 9.2 Hz, 1H, H-4), 6.52 (d, *J* = 2.3 Hz, 1H, H-6), 3.17 (q, *J* = 6.8 Hz, 2H, NH*CH*_2_CH_2_CH_3_), 3.08 (t, *J* = 6.4 Hz, 2H, NCH_2_), 1.82–1.67 (m, 5H, cyclohexyl-CH_2_ × 2 and CH_2_ × 1/2), 1.65–1.60 (m, 2H, NHCH_2_ *CH*_2_CH_3_), 1.58–1.52 (m, 1H, cyclohexyl-CH), 1.32–1.14 (m, 3H, cyclohexyl-CH_2_ and CH_2_ × 1/2), 1.03–0.91 (m, 5H, cyclohexyl-CH_2_ and NHCH_2_CH_2_ *CH*_3_).

#### *N*-(Cyclohexylmethyl)-2-nitro-5-piperidin-1-ylbenzamide (**48**)

Following the general procedure (method B), starting from derivative **46** and using 5.00 equiv of piperidine, after stirring at 110 °C for 2 h, compound **48** was obtained after filtration as a yellow solid in 98% yield (8.10 g). ^1^H NMR (400 MHz, CDCl_3_): δ = 8.05 (d, *J* = 9.4 Hz, 1H, H-3), 6.77 (dd, *J* = 2.8 and 9.4 Hz, 1H, H-4), 6.71 (d, *J* = 2.8 Hz, 1H, H-6), 5.72 (bs, 1H, CONH), 3.47–3.39 (m, 4H, piperidine-NCH_2_ × 2), 3.30 (t, *J* = 6.4 Hz, 2H, NCH_2_), 1.83–1.71 (m, 4H, piperidine-CH_2_ × 2), 1.70–1.64 (m, 6H, piperidine-CH_2_ and cyclohexyl-CH_2_ × 2), 1.64–1.58 (m, 2H, cyclohexyl-CH and CH_2_ × 1/2), 1.34–1.12 (m, 3H, cyclohexyl-CH_2_ and CH_2_ × 1/2), 1.06–0.94 (m, 2H, cyclohexyl-CH_2_).

#### *N*-(Cyclohexylmethyl)-5-morpholin-4-yl-2-nitrobenzamide (**49**)

Following the general procedure (method B), starting from derivative **46** and using 12.00 equiv of morpholine, after stirring at 110 °C for 1 h, compound **49** was obtained after filtration as a yellow solid in 91% yield (0.90 g). ^1^H NMR (400 MHz, CDCl_3_): δ = 8.02 (d, *J* = 9.3 Hz, 1H, H-3), 6.75 (dd, *J* = 2.8 and 9.3 Hz, 1H, H-4), 6.69 (d, *J* = 2.8 Hz, 1H, H-6), 5.67 (bs, 1H, CONH), 3.78 (t, *J* = 4.8 Hz, 4H, morpholine-OCH_2_ × 2), 3.31 (t, *J* = 5.0 Hz, 4H, morpholine-NCH_2_ × 2), 3.23 (t, *J* = 6.3 Hz, 2H, NCH_2_), 1.74–1.49 (m, 6H, cyclohexyl-CH_2_ × 3), 1.25–1.08 (m, 3H, cyclohexyl-CH_2_ and CH), 0.97–0.91 (m, 2H, cyclohexyl-CH_2_).

### General Procedure for Aromatic Nucleophilic Substitution Reaction (Method C)

Under N_2_ atmosphere, to a solution of derivative **46** (1.00 equiv) in dry DMF (5 mL per mmol), an appropriate nucleophilic amine (2.00 equiv) and K_2_CO_3_ (3.00–4.00 equiv) were added. After stirring at 90 °C for 1–3 h, the reaction mixture was poured into ice/water. The mixture was either filtered (if a precipitate formed) or extracted with EtOAc (x3), and the combined organic layers were washed with brine, dried over Na2SO4 and evaporated to dryness. The corresponding substituted derivative was used in the next step without further purification.

#### *N*-(Cyclohexylmethyl)-2-nitro-5-thiomorpholin-4-ylbenzamide (**50**)

Following the general procedure (method C), using thiomorpholine and 4.00 equiv of K_2_CO_3_, after stirring for 3 h, compound **50** was obtained after filtration as a solid in 92% yield (0.99 g). ^1^H NMR (400 MHz, DMSO-*d*_6_): δ = 8.37 (t, *J* = 5.6 Hz, 1H, CONH), 7.97 (d, *J* = 9.4 Hz, 1H, H-3), 7.01 (dd, *J* = 2.7 and 9.4 Hz, 1H, H-4), 6.79 (d, *J* = 2.7 Hz, 1H, H-6), 3.87 (t, *J* = 4.7 Hz, 4H, thiomorpholine-NCH_2_ × 2), 3.04 (t, *J* = 6.3 Hz, 2H, NCH_2_), 2.65 (t, *J* = 4.8 Hz, 4H, thiomorpholine-SCH_2_ × 2), 1.78–1.54 (m, 5H, cyclohexyl-CH_2_ × 2 and CH_2_ × 1/2), 1.53–1.50 (m, 1H, cyclohexyl-CH), 1.26–1.13 (m, 3H, cyclohexyl-CH_2_ × 2 and CH_2_ × 1/2), 0.93–0.87 (m, 2H, cyclohexyl-CH_2_).

#### *N*-(Cyclohexylmethyl)-5-(4-methylpiperazin-1-yl)-2-nitrobenzamide (**51**)

Following the general procedure (method B), starting from derivative **46** and using 12.00 equiv of *N*-methylpiperazine, after stirring at 110 °C for 1 h, compound **51** was obtained after filtration as a yellow solid in 75% yield (0.77 g). ^1^H NMR (400 MHz, CDCl_3_): δ = 8.00 (d, *J* = 9.4 Hz, 1H, H-3), 6.74 (dd, *J* = 2.8 and 9.4 Hz, 1H, H-4), 6.58 (d, *J* = 2.8 Hz, 1H, H-6), 5.65 (bs, 1H, CONH), 3.37 (t, *J* = 5.0 Hz, 4H, piperazine-NCH_2_ × 2), 3.23 (t, *J* = 6.3 Hz, 2H, NCH_2_), 2.47 (t, *J* = 5.1 Hz, 4H, piperazine-NCH_2_ × 2), 2.28 (s, 3H, piperazine-NCH_3_), 1.77–1.60 (m, 5H, cyclohexyl-CH_2_ × 2 and CH_2_ × 1/2), 1.55–1.50 (m, 1H, cyclohexyl-CH), 1.26–1.01 (m, 3H, cyclohexyl-CH_2_ × 2 and CH_2_ × 1/2), 0.98–0.86 (m, 2H, cyclohexyl-CH_2_).

#### *N*-(Cyclohexylmethyl)-5-(1,4-dioxa-8-azaspiro­[4.5]­dec-8-yl)-2-nitrobenzamide (**52**)

Following the general procedure (method C), using 1,4-dioxa-8-azaspiro[4.5]­decane and 3.00 equiv of K_2_CO_3_, after stirring for 3 h, compound **52** was obtained after filtration as a yellow solid in 79% yield (0.11 g). ^1^H NMR (400 MHz, CDCl_3_): δ = 8.00 (d, *J* = 9.4 Hz, 1H, H-3), 6.74 (dd, *J* = 2.9 and 9.4 Hz, 1H, H-4), 6.68 (d, *J* = 2.8 Hz, 1H, H-6), 5.64 (bs, 1H, CONH), 3.93 (s, 4H, “dioxolane”-OCH_2_ × 2), 3.50 (t, *J* = 5.7 Hz, 4H, “piperidine”-NCH_2_ × 2), 3.23 (t, *J* = 6.5 Hz, 2H, NCH_2_), 1.73 (t, *J* = 5.9 Hz, 4H, “piperidine”-CH_2_ × 2), 1.70–1.58 (m, 6H, cyclohexyl-CH_2_ × 2 and CH_2_ × 1/2 and CH), 1.28–1.04 (m, 3H, cyclohexyl-CH_2_ × 2 and CH_2_ × 1/2), 0.98–0.86 (m, 2H, cyclohexyl-CH_2_).

#### *N*-(Cyclohexylmethyl)-2-nitro-5-(4-phenylpiperidin-1-yl)­benzamide (**53**)

Following the general procedure (method C), using 4-phenylpiperidine and 3.00 equiv of K_2_CO_3_, after stirring for 1 h, compound **53** was obtained after filtration as a yellow solid in 81% yield (0.12 g). ^1^H NMR (400 MHz, DMSO-*d*_6_) δ = 8.42 (t, *J* = 5.7 Hz, 1H, CONH), 8.03 (d, *J* = 9.4 Hz, 1H, H-3), 7.39–7.24 (m, 5H, Ar–H), 7.12 (dd, *J* = 2.7 and 9.4 Hz, 1H, H-4), 6.89 (d, *J* = 2.7 Hz, 1H, H-6), 4.25–4.22 (m, 2H, NCH_2_), 3.17–3.10 (m, 4H, piperidine-NCH_2_ × 2), 3.09–2.88 (m, 1H, piperidine-CH), 2.15–1.67 (m, 9H, piperidine-CH_2_ × 2 and cyclohexyl-CH_2_ × 2 and CH_2_ × 1/2), 1.63–1.59 (m, 1H, cyclohexyl-CH), 1.48–1.30 (m, 3H, cyclohexyl-CH_2_ and CH_2_ × 1/2), 1.02–0.90 (m, 2H, cyclohexyl-CH_2_).

#### *N*-(Cyclohexylmethyl)-5-(4-hydroxypiperidin-1-yl)-2-nitrobenzamide (**54**)

Following the general procedure (method C), using 4-hydroxypiperidine and 3.00 equiv of K_2_CO_3_, after stirring for 4 h, compound **54** was obtained after extraction as a yellow solid in 78% yield (0.10 g). ^1^H NMR (400 MHz, DMSO-*d*_6_) δ = 8.34 (t, *J* = 5.8 Hz, 1H, CONH), 7.95 (d, *J* = 9.4 Hz, 1H, H-3), 7.00 (dd, *J* = 2.8 and 9.5 Hz, 1H, H-4), 6.76 (d, *J* = 2.8 Hz, 1H, H-6), 4.77 (d, *J* = 4.1 Hz, 1H, OH), 3.86–3.72 (m, 3H, piperidine-NCH_2_ and CH), 3.25–3.15 (m, 2H, piperidine-NCH_2_), 3.04 (t, *J* = 6.3 Hz, 2H, NCH_2_), 1.86–1.60 (m, 7H, piperidine-CH_2_ × 2 and cyclohexyl-CH_2_ and CH_2_ × 1/2), 1.58–1.48 (m, 1H, cyclohexyl-CH), 1.45–1.34 (m, 2H, cyclohexyl-CH_2_), 1.27–1.08 (m, 3H, cyclohexyl-CH_2_ and CH_2_ × 1/2), 0.98–0.85 (m, 2H, cyclohexyl-CH_2_).

#### *N*-(Cyclohexylmethyl)-2-nitro-5-(octahydroisoquinolin-2­(1*H*)-yl)­benzamide (**55**)

Following the general procedure (method C), using perhydroisoquinoline and 4.00 equiv of K_2_CO_3_, after stirring for 1 h, compound **55** was obtained after filtration as a solid in 99% yield (0.99 g). ^1^H NMR (400 MHz, DMSO-*d*_6_) δ = 8.34 (t, *J* = 5.7 Hz, 1H, CONH), 7.94 (d, *J* = 9.4 Hz, 1H, H-3), 7.00 (dd, *J* = 2.8 and 9.5 Hz, 1H, H-4), 6.76 (d, *J* = 2.7 Hz, 1H, H-6), 4.10–4.07 (m, 1H, perhydroisoquinoline-NCH_2_ × 1/2), 3.93–3.90 (m, 1H, perhydroisoquinoline-NCH_2_ × 1/2), 3.04 (t, *J* = 6.3 Hz, 2H, NCH_2_), 2.99–2.89 (m, 1H, perhydroisoquinoline-NCH_2_ × 1/2), 2.64–2.56 (m, 1H, perhydroisoquinoline-NCH_2_ × 1/2), 1.80–1.58 (m, 10H, aliphatic-H), 1.56–1.48 (m, 1H, cyclohexyl-CH), 1.35–1.05 (m, 8H, aliphatic-H), 1.04–0.84 (m, 4H, aliphatic-H).

#### *N*-(Cyclohexylmethyl)-5-(3,5-dimethylpiperidin-1-yl)-2-nitrobenzamide (**56**)

Following the general procedure (method C), using 3,5-dimethylpiperidine and 4.00 equiv of K_2_CO_3_, after stirring for 1 h, compound **56** was obtained after filtration as a solid in 94% yield (0.63 g). ^1^H NMR (400 MHz, CDCl_3_) δ = 7.98 (d, *J* = 9.4 Hz, 1H, H-3), 6.70 (dd, *J* = 2.7 and 9.5 Hz, 1H, H-4), 6.64 (d, *J* = 2.7 Hz, 1H, H-6), 5.60 (bs, 1H, CONH), 3.83–3.71 (m, 2H, piperidine-NCH_2_), 3.24 (t, *J* = 6.4 Hz, 2H, NCH_2_), 2.44–2.35 (m, 2H, piperidine-NCH_2_), 2.02–1.90 (m, 1H, aliphatic-H), 1.85–1.55 (m, 8H, aliphatic-H), 1.30–1.10 (m, 4H, aliphatic-H), 0.90–0.75 (m, 7H, piperidine-CH_3_ × 2 and aliphatic-H), 0.74–0.70 (m, 1H, aliphatic-H).

#### *N*-(Cyclohexylmethyl)-5-(4-methylpiperidin-1-yl)-2-nitrobenzamide (**57**)

Following the general procedure (method C), using 4-methylpiperidine and 4.00 equiv of K_2_CO_3_, after stirring for 1 h, compound **57** was obtained after filtration as a solid in 92% yield (0.83 g). ^1^H NMR (400 MHz, CDCl_3_) δ = 7.98 (d, *J* = 9.4 Hz, 1H, H-3), 6.71 (dd, *J* = 2.9 and 9.4 Hz, 1H, H-4), 6.65 (d, *J* = 2.8 Hz, 1H, H-6), 5.60 (bs, 1H, CONH), 3.86–3.83 (m, 2H, piperidine-NCH_2_), 3.23 (t, *J* = 6.4 Hz, 2H, NCH_2_), 2.93–2.86 (m, 2H, piperidine-NCH_2_), 1.78–1.54 (m, 9H, aliphatic-H), 1.26–1.04 (m, 5H, aliphatic-H), 0.98–0.87 (m, 5H, piperidine-CH_3_ and aliphatic-H).

### General Procedure for the Reduction Reaction (Method D)

To a solution of the appropriate nitro derivative (1.00 equiv) in the solvent indicated for each compound (10 mL per mmol), Raney-Ni was added, and the reaction mixture was hydrogenated for 1–48 h at rt. The mixture was filtered over Celite and the solvent evaporated under reduced pressure to give a crude product that was either purified, as described below for each compound, or used as such for the next reaction.

#### 2-Amino-*N*-(cyclohexylmethyl)-5-(cyclopropylmethoxy)­benzamide (**58**)

Following the general procedure (method D), starting from intermediate **44** dissolved in EtOAc, after stirring for 6 h, compound **58** was obtained as a yellow solid in 91% yield (1.19 g). ^1^H NMR (400 MHz, CDCl_3_) δ = 6.91 (d, *J* = 2.8 Hz, 1H, H-6), 6.86 (dd, *J* = 2.8 and 8.8 Hz, 1H, H-4), 6.65 (d, *J* = 8.7 Hz, 1H, H-3), 6.13 (bs, 1H, CONH), 5.04 (bs, 2H, NH_2_), 3.74 (d, *J* = 6.9 Hz, 2H, OCH_2_), 3.25 (t, *J* = 6.4 Hz, 2H, NCH_2_), 1.83–1.63 (m, 5H, cyclohexyl-CH_2_ × 2 and cyclopropyl-CH), 1.62–1.49 (m, 1H, cyclohexyl-CH), 1.33–1.11 (m, 4H, cyclohexyl-CH_2_ × 2), 1.05–0.93 (m, 2H, cyclohexyl-CH_2_), 0.66–0.60 (m, 2H, cyclopropyl-CH_2_), 0.39–0.30 (m, 2H, cyclopropyl-CH_2_).

#### 2-Amino-*N*-(cyclohexylmethyl)-5-(cyclohexyloxy)­benzamide (**59**)

Following the general procedure (method D), starting from intermediate **45** dissolved in EtOAc, after stirring for 1 h, compound **59** was obtained as a yellow oil in 97% yield (0.40 g). ^1^H NMR (400 MHz, CDCl_3_) δ = 6.91 (d, *J* = 2.7 Hz, 1H, H-6), 6.87 (dd, *J* = 2.7 and 8.7 Hz, 1H, H-4), 6.47 (d, *J* = 8.7 Hz, 1H, H-3), 6.12 (bs, 1H, CONH), 5.09 (bs, 2H, NH_2_), 4.08–4.01 (m, 1H, cyclohexyl-OCH), 3.25 (t, *J* = 6.4 Hz, 2H, NCH_2_), 2.01–1.90 (m, 2H, cyclohexyl-CH_2_), 1.84–1.64 (m, 8H, cyclohexyl-CH_2_ × 4), 1.61–1.52 (m, 2H, cyclohexyl-CH_2_ × 1/2 and CH), 1.51–1.40 (m, 2H, cyclohexyl-CH_2_), 1.37–1.11 (m, 5H, cyclohexyl-CH_2_ × 2 and CH_2_ × 1/2), 1.05–0.91 (m, 2H, cyclohexyl-CH_2_).

#### 2-Amino-*N*-(cyclohexylmethyl)-5-(propylamino)­benzamide (**60**)

Following the general procedure (method D), starting from intermediate **47** dissolved in EtOH, after stirring for 4 h, compound **60** was obtained as a black solid in 98% yield (1.02 g). ^1^H NMR (400 MHz, DMSO-*d*_6_) δ = 8.09 (t, *J* = 5.6 Hz, 1H, CONH), 6.66 (d, *J* = 2.3 Hz, 1H, H-6), 6.56 (dd, *J* = 2.4 and 8.6 Hz, 1H, H-4), 6.52 (d, *J* = 8.6 Hz, 1H, H-3), 5.40 (s, 2H, NH_2_), 4.70 (bs, 1H, *NH*CH_2_CH_2_CH_3_), 3.04 (t, *J* = 6.4 Hz, 2H, NCH_2_), 2.95–2.87 (m, 2H, NH*CH*_2_CH_2_CH_3_), 1.75–1.65 (m, 4H, cyclohexyl-CH_2_ × 2), 1.65–1.58 (m, 1H, cyclohexyl-CH), 1.57–1.46 (m, 3H, NHCH_2_ *CH*_2_CH_3_ and cyclohexyl-CH_2_ × 1/2), 1.27–1.09 (m, 3H, cyclohexyl-CH_2_ and CH_2_ × 1/2), 0.99–0.84 (m, 5H, NHCH_2_CH_2_ *CH*_3_ and cyclohexyl-CH_2_).

#### 2-Amino-*N*-(cyclohexylmethyl)-5-piperidin-1-ylbenzamide (**61**)

Following the general procedure (method D), starting from intermediate **48** dissolved in MeOH, after stirring for 4 h, compound **61** was obtained as a green solid in 44% yield (0.27 g). ^1^H NMR (400 MHz, DMSO-*d*_6_) δ = 8.24 (t, *J* = 5.6 Hz, 1H, CONH), 7.07 (d, *J* = 2.6 Hz, 1H, H-6), 6.93 (dd, *J* = 2.8 and 8.8 Hz, 1H, H-4), 6.66 (d, *J* = 8.8 Hz, 1H, H-3), 5.91 (s, 2H, NH_2_), 3.10 (t, *J* = 6.4 Hz, 2H, NCH_2_), 2.97 (t, *J* = 5.2 Hz, 4H, piperidine-NCH_2_ × 2), 1.80–1.71 (m, 4H, piperidine-CH_2_ x 2), 1.70–1.64 (m, 6H, piperidine-CH_2_ and cyclohexyl-CH_2_ × 2), 1.63–1.49 (m, 3H, cyclohexyl-CH_2_ and CH), 1.32–1.13 (m, 2H, cyclohexyl-CH_2_), 1.04–0.90 (m, 2H, cyclohexyl-CH_2_).

#### 2-Amino-*N*-(cyclohexylmethyl)-5-morpholin-4-ylbenzamide (**62**)

Following the general procedure (method D), starting from intermediate **49** dissolved in EtOAc, after stirring for 2 h, compound **62** was obtained as a yellow solid in 48% yield (0.35 g). ^1^H NMR (400 MHz, DMSO-*d*_6_) δ = 8.25 (t, *J* = 5.6 Hz, 1H, CONH), 7.08 (d, *J* = 2.6 Hz, 1H, H-6), 6.95 (dd, *J* = 2.6 and 8.8 Hz, 1H, H-4), 6.69 (d, *J* = 8.8 Hz, 1H, H-3), 5.96 (s, 2H, NH_2_), 3.78 (t, *J* = 4.5 Hz, 4H, morpholine-OCH_2_ × 2), 3.11 (t, *J* = 6.3 Hz, 2H, NCH_2_), 2.99 (t, *J* = 4.7 Hz, 4H, morpholine-NCH_2_ × 2), 1.82–1.64 (m, 5H, cyclohexyl-CH_2_ × 2 and CH_2_ × 1/2), 1.62–1.52 (m, 1H, cyclohexyl-CH), 1.32–1.14 (m, 3H, cyclohexyl-CH_2_ and CH_2_ × 1/2), 1.04–0.89 (m, 2H, cyclohexyl-CH_2_).

#### 2-Amino-*N*-(cyclohexylmethyl)-5-thiomorpholin-4-ylbenzamide (**63**)

Following the general procedure (method D), starting from intermediate **50** dissolved in EtOAc, after stirring for 1 h, compound **63** was obtained as a solid in 72% yield (0.63 g). ^1^H NMR (400 MHz, DMSO-*d*_6_) δ = 8.25 (t, *J* = 5.4 Hz, 1H, CONH), 7.10 (d, *J* = 1.9 Hz, 1H, H-6), 6.94 (dd, *J* = 2.2 and 8.7 Hz, 1H, H-4), 6.68 (d, *J* = 8.7 Hz, 1H, H-3), 6.00 (s, 2H, NH_2_), 3.27 (t, *J* = 4.5 Hz, 4H, thiomorpholine-NCH_2_ × 2), 3.10 (t, *J* = 5.8 Hz, 2H, NCH_2_), 2.77 (t, *J* = 4.7 Hz, 4H, thiomorpholine-SCH_2_ × 2), 1.77–1.69 (m, 5H, cyclohexyl-CH_2_ × 2 and CH_2_ × 1/2), 1.59–1.56 (m, 1H, cyclohexyl-CH), 1.26–1.21 (m, 3H, cyclohexyl-CH_2_ and CH_2_ × 1/2), 1.01–0.95 (m, 2H, cyclohexyl-CH_2_).

#### 2-Amino-*N*-(cyclohexylmethyl)-5-(4-methylpiperazin-1-yl)­benzamide (**64**)

Following the general procedure (method D), starting from intermediate **51** dissolved in EtOAc, after stirring for 2 h, compound **64** was obtained as a yellow solid in 90% yield (0.58 g). ^1^H NMR (400 MHz, CDCl_3_) δ = 6.87–6.85 (m, 2H, Ar–H), 6.60–6.57 (m, 1H, Ar–H), 6.18 (bs, 1H, CONH), 4.06 (bs, 2H, NH_2_), 3.18 (t, *J* = 6.4 Hz, 2H, NCH_2_), 3.02 (t, *J* = 4.8 Hz, 4H, piperazine-NCH_2_ × 2), 2.57 (t, *J* = 4.9 Hz, 4H, piperazine-NCH_2_ × 2), 2.32 (s, 3H, piperazine-NCH_3_), 1.75–1.56 (m, 5H, cyclohexyl-CH_2_ × 2 and CH_2_ × 1/2), 1.54–1.44 (m, 1H, cyclohexyl-CH), 1.25–1.04 (m, 3H, cyclohexyl-CH_2_ and CH_2_ × 1/2), 0.98–0.84 (m, 2H, cyclohexyl-CH_2_).

#### 2-Amino-*N*-(cyclohexylmethyl)-5-(1,4-dioxa-8-azaspiro­[4.5]­dec-8-yl)­benzamide (**65**)

Following the general procedure (method D), starting from intermediate **52** dissolved in EtOAc, after stirring for 2 h, compound **65** was obtained as a brown oil in 71% yield (0.06 g). ^1^H NMR (400 MHz, CDCl_3_) δ = 6.96–6.91 (m, 2H, Ar–H), 6.59–6.57 (m, 1H, Ar–H), 6.08 (bs, 1H, CONH), 5.02 (bs, 2H, NH_2_), 3.93 (s, 4H, “dioxolane”-OCH_2_ × 2), 3.18 (t, *J* = 6.4 Hz, 2H, NCH_2_), 3.07 (bs, 4H, “piperidine”-NCH_2_ × 2), 1.83 (t, *J* = 5.4 Hz, 4H, “piperidine”-CH_2_ × 2), 1.74–1.56 (m, 5H, cyclohexyl-CH_2_ × 2 and CH_2_ × 1/2), 1.55–1.45 (m, 1H, cyclohexyl-CH), 1.24–1.06 (m, 3H, cyclohexyl-CH_2_ and CH_2_ × 1/2), 0.98–0.84 (m, 2H, cyclohexyl-CH_2_).

#### 2-Amino-*N*-(cyclohexylmethyl)-5-(4-phenylpiperidin-1-yl)­benzamide (**66**)

Following the general procedure (method D), starting from intermediate **53** dissolved in EtOAc, after stirring for 3 h, compound **66** was obtained as a light brown solid in 57% yield (0.48 g). ^1^H NMR (400 MHz, DMSO-*d*_6_) δ = 8.27 (t, *J* = 5.7 Hz, 1H, CONH), 7.40–7.33 (m, 4H, Ar–H), 7.28–7.23 (m, 1H, Ar–H), 7.13 (d, *J* = 2.7 Hz, 1H, H-6), 6.99 (dd, *J* = 2.6 and 8.8 Hz, 1H, H-4), 6.68 (d, *J* = 8.8 Hz, 1H, H-3), 5.95 (s, 2H, NH_2_), 3.63–3.56 (m, 2H, piperidine-NCH_2_), 3.11 (t, *J* = 6.3 Hz, 2H, NCH_2_), 2.74–2.60 (m, 2H, piperidine-NCH_2_), 1.96–1.82 (m, 4H, piperidine-CH_2_ × 2), 1.80–1.70 (m, 5H, cyclohexyl-CH_2_ × 2 and CH_2_ × 1/2), 1.68–1.64 (m, 1H, piperidine-CH), 1.62–1.54 (m, 1H, cyclohexyl-CH), 1.31–1.12 (m, 3H, cyclohexyl-CH_2_ and CH_2_ × 1/2), 1.04–0.90 (m, 2H, cyclohexyl-CH_2_).

#### 2-Amino-*N*-(cyclohexylmethyl)-5-(4-hydroxypiperidin-1-yl)­benzamide (**67**)

Following the general procedure (method D), starting from intermediate **54** dissolved in MeOH, after stirring for 1 h, compound **67** was obtained as a black solid in 70% yield (0.45 g). ^1^H NMR (400 MHz, CDCl_3_): δ = 6.94–6.76 (m, 2H, H-6 and H-4), 6.58 (d, *J* = 9.0 Hz, 1H, H-3), 6.11 (bs, 1H, CONH), 4.98 (bs, 2H, NH_2_), 3.81–3.72 (m, 1H, piperidine-CH), 3.29–3.21 (m, 2H, piperidine-NCH_2_), 3.18 (t, *J* = 6.4 Hz, 2H, NCH_2_), 2.77–2.69 (m, 2H, piperidine-NCH_2_), 2.02–1.90 (m, 2H, aliphatic-H), 1.76–1.58 (m, 7H, OH and aliphatic-H), 1.54–1.44 (m, 1H, cyclohexyl-CH), 1.26–1.06 (m, 4H, aliphatic-H), 0.98–0.86 (m, 2H, cyclohexyl-CH_2_).

#### 2-Amino-*N*-(cyclohexylmethyl)-5-(octahydroisoquinolin-2­(1*H*)-yl)­benzamide (**68**)

Following the general procedure (method D), starting from intermediate **55** dissolved in EtOAc, after stirring for 1 h, compound **68** was obtained as a solid in 86% yield (0.76 g). ^1^H NMR (400 MHz, DMSO-*d*_6_): δ = 8.23 (t, *J* = 5.5 Hz, 1H, CONH), 7.06 (d, *J* = 2.5 Hz, 1H, H-6), 6.93 (dd, *J* = 2.5 and 8.8 Hz, 1H, H-4), 6.65 (d, *J* = 8.8 Hz, 1H, H-3), 5.89 (s, 2H, NH_2_), 3.50–3.45 (m, 1H, perhydroisoquinoline-NCH_2_ × 1/2), 3.35–3.29 (m, 1H, perhydroisoquinoline-NCH_2_ × 1/2), 3.10 (t, *J* = 6.3 Hz, 2H, NCH_2_), 2.20–2.25 (m, 1H, perhydroisoquinoline-NCH_2_ × 1/2), 1.82–1.62 (m, 10H, aliphatic-H), 1.59–1.53 (m, 1H, cyclohexyl-CH), 1.40–1.15 (m, 8H, aliphatic-H), 1.10–0.88 (m, 5H, aliphatic-H).

#### 2-Amino-*N*-(cyclohexylmethyl)-5-(3,5-dimethylpiperidin-1-yl)­benzamide (**69**)

Following the general procedure (method D), starting from intermediate **56** dissolved in EtOAc, after stirring for 4 h, compound **69** was obtained after purification by flash column chromatography eluting with Cy/EtOAc (70:30) as a solid in 45% yield (0.25 g). ^1^H NMR (400 MHz, DMSO-*d*_6_): δ = 8.18 (t, *J* = 5.3 Hz, 1H, CONH), 6.95–7.00 (m, 1H, Ar–H), 6.89–6.87 (m, 1H, Ar–H), 6.60 (d, *J* = 8.7 Hz, 1H, H-3), 5.85 (s, 2H, NH_2_), 3.40–3.35 (m, 1H, aliphatic-H), 3.05 (t, *J* = 6.3 Hz, 2H, NCH_2_), 2.10–2.00 (m, 2H, aliphatic-H), 1.75–1.60 (m, 8H, aliphatic-H), 1.55–1.50 (m, 1H, cyclohexyl-CH), 1.20–1.10 (m, 4H, aliphatic-H), 0.90–0.85 (m, 8H, piperidine-CH_3_ × 2 and aliphatic-H), 0.70–0.60 (m, 1H, aliphatic-H).

#### 2-Amino-*N*-(cyclohexylmethyl)-5-(4-methylpiperidin-1-yl)­benzamide (**70**)

Following the general procedure (method D), starting from intermediate **57** dissolved in MeOH, after stirring for 1 h, compound **70** was obtained as a green solid in 74% yield (0.54 g). ^1^H NMR (400 MHz, DMSO-*d*_6_): δ = 8.23 (bs, 1H, CONH), 7.07 (d, *J* = 2.3 Hz, 1H, H-6), 6.93 (dd, *J* = 2.3 and 8.7 Hz, 1H, H-4), 6.64 (d, *J* = 8.8 Hz, 1H, H-3), 5.91 (s, 2H, NH_2_), 3.45–3.39 (m, 2H, piperidine-NCH_2_), 3.09 (t, *J* = 6.3 Hz, 2H, NCH_2_), 1.79–1.64 (m, 7H, aliphatic-H), 1.60–1.42 (m, 2H, piperidine-CH and cyclohexyl-CH), 1.35–1.14 (m, 7H, aliphatic-H), 1.02–0.88 (m, 5H, piperidine-CH_3_ and aliphatic-H).

### General Procedure for Nucleophilic Acyl Substitution Reaction (Method E)

Under N_2_ atmosphere, to a solution of an appropriate 2-amino derivative (1.00 equiv) in dry pyridine (4 mL per mmol), properly substituted sulfonyl chloride (1.50 equiv) was added portion wise, and the reaction mixture was stirred at rt for 1–48 h. After pouring into ice/water, the pH was adjusted up to 6 with 2 M HCl. The mixture was either filtered (if a precipitate formed) or extracted with EtOAc (×3), and the combined organic layers were washed with brine, dried over Na_2_SO_4_ and evaporated to dryness. The obtained crude product was purified as described below for each compound.

#### *N*-(cyclohexylmethyl)-5-(cyclopropylmethoxy)-2-[(quinolin-7-ylsulfonyl)­amino]­benzamide (**4**)

Following the general procedure (method E), starting from intermediate **58** and using 8-quinolinesulfonyl chloride, after stirring for 1 h, compound **4** was obtained after filtration and purification by crystallization by Cy/EtOAc as a yellow solid in 47% yield (0.23 g). ^1^H NMR (400 MHz, DMSO-*d*_6_): δ = 10.96 (s, 1H, NHSO_2_), 8.93 (dd, *J* = 1.6 and 4.2 Hz, 1H, Ar–H), 8.44 (dd, *J* = 1.6 and 8.4 Hz, 1H, Ar–H), 8.30 (dd, *J* = 1.2 and 7.3 Hz, 1H, Ar–H), 8.25–8.18 (m, 2H, Ar–H), 7.68–7.61 (m, 2H, Ar–H and CONH), 7.45 (d, *J* = 8.90 Hz, 1H, Ar–H), 6.91–6.84 (m, 2H, Ar–H), 3.65 (d, *J* = 7.02 Hz, 2H, OCH_2_), 2.90 (t, *J* = 6.3 Hz, 2H, NCH_2_), 1.68–1.52 (m, 5H, cyclohexyl-CH_2_ × 2 and CH_2_ × 1/2), 1.47–1.30 (m, 1H, cyclopropyl-CH), 1.21–1.01 (m, 4H, cyclohexyl-CH_2_ × 2 and CH), 0.90–0.75 (m, 2H, cyclohexyl-CH_2_), 0.49–0.43 (m, 2H, cyclopropyl-CH_2_), 0.21–0.15 (m, 2H, cyclopropyl-CH_2_). ^13^C NMR (100 MHz, DMSO-*d*_6_): δ = 167.48, 154.35, 151.84, 142.97, 137.30, 135.22, 134.80, 132.50, 130.82, 128.79, 126.02, 124.25, 123.02, 121.04, 117.71, 114.68, 72.70, 45.75, 37.63, 30.96, 26.49, 25.85, 10.46, 3.47. HPLC: Method A; *r*_t_ = 9.227 min. HRMS (ESI) calculated for C_27_H_31_N_3_O_4_S [M + H]^+^ 494.2108, found 494.2100.

#### *N*-(Cyclohexylmethyl)-5-(cyclohexyloxy)-2-[(quinolin-7-ylsulfonyl)­amino]­benzamide (**5**)

Following the general procedure (method E), starting from intermediate **59** and using 8-quinolinesulfonyl chloride, after stirring for 1 h, compound **5** was obtained after filtration and purification by crystallization by Cy/EtOAc as a white solid in 53% yield (0.30 g). ^1^H NMR (400 MHz, DMSO-*d*_6_): δ = 10.94 (s, 1H, NHSO_2_), 8.99 (dd, *J* = 1.6 and 4.2 Hz, 1H, Ar–H), 8.50 (dd, *J* = 1.4 and 8.4 Hz, 1H, Ar–H), 8.35 (dd, *J* = 1.1 and 7.3 Hz, 1H, Ar–H), 8.30–8.24 (m, 2H, Ar–H), 7.75–7.67 (m, 2H, Ar–H and CONH), 7.50 (d, *J* = 9.0 Hz, 1H, Ar–H), 6.98–6.90 (m, 2H, Ar–H), 4.24–4.15 (m, 1H, OCH), 2.94 (t, *J* = 6.3 Hz, 2H, NCH_2_), 1.86–1.74 (m, 2H, cyclohexyl-CH_2_), 1.73–1.56 (m, 7H, cyclohexyl-CH_2_ × 3 and CH_2_ × 1/2), 1.53–1.38 (m, 2H, cyclohexyl-CH and CH_2_ × 1/2), 1.37–1.26 (m, 4H, cyclohexyl-CH_2_ × 2), 1.25–1.08 (m, 4H, cyclohexyl-CH_2_ × 2), 0.93–0.80 (m, 2H, cyclohexyl-CH_2_). ^13^C NMR (100 MHz, CDCl_3_): δ 167.16, 154.72, 151.50, 143.42, 136.55, 136.20, 133.47, 131.50, 129.30, 128.70, 128.58, 125.47, 124.33, 122.26, 118.17, 115.75, 75.91, 46.10, 37.80, 31.68, 30.91, 26.35, 25.80, 25.47, 23.66. HPLC: Method A; *r*_t_ = 6.980 min. HRMS (ESI) calculated for C_29_H_35_N_3_O_4_S [M + H]^+^ 522.2421, found 522.2415.

#### *N*-(Cyclohexylmethyl)-5-(propylamino)-2-[(quinolin-7-ylsulfonyl)­amino]­benzamide (**6**)

Following the general procedure (method E), starting from intermediate **60** and using 8-quinolinesulfonyl chloride, after stirring for 1 h, compound **6** was obtained after filtration and purification by flash chromatography column eluting with CHCl_3_/MeOH (98:2) as a yellow solid in 12% yield (0.07 g). ^1^H NMR (400 MHz, CDCl_3_) *δ =* 9.13 (dd, *J* = 1.6 and 4.2 Hz, 1H, Ar–H), 9.08 (s, 1H, NHSO_2_), 8.32 (dd, *J* = 1.2 and 7.3 Hz, 1H, Ar–H), 8.28 (dd, *J* = 1.6 and 8.4 Hz, 1H, Ar–H), 8.04 (dd, *J* = 1.1 and 8.2 Hz, 1H, Ar–H), 7.61–7.55 (m, 2H, Ar–H), 6.74–6.70 (m, 2H, Ar–H), 6.67 (bs, 1H, CONH), 6.32 (dd, *J* = 2.8 and 8.8 Hz, 1H, Ar–H), 3.63 (bs, 1H, *NH*CH_2_CH_2_CH_3_), 3.11 (t, *J* = 6.4 Hz, 2H, NCH_2_), 2.97 (t, *J =* 7.0 Hz, 2H, NH*CH*_2_CH_2_CH_3_), 1.81–1.69 (m, 4H, cyclohexyl-CH_2_ × 2), 1.69–1.61 (m, 1H, cyclohexyl-CH_2_ × 1/2), 1.56–1.43 (m, 3H, NHCH_2_ *CH*_2_CH_3_ and cyclohexyl-CH), 1.31–1.11 (m, 3H, cyclohexyl-CH_2_ and CH_2_ × 1/2), 1.02–0.88 (m, 5H, cyclohexyl-CH_2_ and NHCH_2_CH_2_ *CH*_3_). ^13^C NMR (100 MHz, CDCl_3_): *δ =* 167.43, 151.51, 146.58, 143.37, 136.71, 136.23, 133.37, 131.98, 131.46, 128.73, 125.90, 125.59, 123.86, 122.28, 114.53, 112.38, 46.13, 45.65, 37.82, 30.94, 26.40, 25.84, 22.53, 11.52. HPLC: a gradient consisting of ACN and water containing 0.1% formic acid, with a linear increase of ACN from 20% to 100% over 15 min; flow: 0.4 mL/min.; *r*_t_ = 12.610 min. HRMS (ESI) calculated for C_26_H_32_N_4_O_3_S [M + H]^+^ 481.2268, found 481.2274.

#### *N*-(Cyclohexylmethyl)-5-piperidin-1-yl-2-[(quinolin-7-ylsulfonyl)­amino]­benzamide (**7**)

Following the general procedure (method E), starting from intermediate **61**, and using 8-quinolinesulfonyl chloride, after stirring for 4 h, compound **7** was obtained after extraction and purification by flash chromatography column eluting with CHCl_3_/MeOH (98:2) as a solid in 14% yield (0.06 g). ^1^H NMR (400 MHz, CDCl_3_): *δ =* 9.38 (s, 1H, NHSO_2_), 9.06 (dd, *J* = 1.7 and 4.2 Hz, 1H, Ar–H), 8.28 (dd, *J* = 1.4 and 7.3 Hz, 1H, Ar–H), 8.19 (dd, *J* = 1.7 and 8.4 Hz, 1H, Ar–H), 7.96 (dd, *J* = 1.3 and 8.2 Hz, 1H, Ar–H), 7.53–7.46 (m, 2H, Ar–H), 6.93–6.87 (m, 2H, Ar–H), 6.62 (dd, *J* = 2.9 and 8.9 Hz, 1H, Ar–H), 6.41 (bs, 1H, CONH), 3.05 (t, *J* = 6.4 Hz, 2H, NCH_2_), 2.96 (t, *J* = 5.2 Hz, 4H, piperidine-NCH_2_ × 2), 1.71–1.63 (m, 4H, piperidine-CH_2_ × 2), 1.60–1.52 (m, 5H, piperidine-CH_2_ and cyclohexyl-CH_2_ and CH), 1.48–1.35 (m, 3H, cyclohexyl-CH_2_ and CH_2_ × 1/2), 1.24–1.03 (m, 3H, cyclohexyl-CH_2_ and CH_2_ × 1/2), 0.95–0.82 (m, 2H, cyclohexyl-CH_2_). ^13^C NMR (100 MHz, CDCl_3_): *δ =* 166.67, 150.47, 148.49, 142.38, 135.60, 135.21, 132.39, 130.49, 128.94, 127.68, 125.41, 124.50, 123.40, 121.24, 117.71, 114.90, 49.34, 45.15, 36.77, 29.93, 25.36, 24.80, 24.64, 23.01. HPLC: Method A; *r*_t_ = 7.980 min. HRMS (ESI) calculated for C_28_H_34_N_4_O_3_S [M + H]^+^ 507.2425, found 507.2427.

#### *N*-(Cyclohexylmethyl)-5-morpholin-4-yl-2-[(quinolin-8-ylsulfonyl)­amino]­benzamide (**8**)

Following the general procedure (method E), starting from intermediate **62** and using 8-quinolinesulfonyl chloride, after stirring for 1 h, compound **8** was extracted after filtration and purification by crystallization by Cy/EtOAc as a white solid in 45% yield (0.22 g). ^1^H NMR (400 MHz, DMSO-*d*_6_): δ = 10.95 (s, 1H, NHSO_2_), 8.99 (dd, *J* = 1.6 and 4.1 Hz, 1H, Ar–H), 8.50 (dd, *J* = 1.5 and 8.4 Hz, 1H, Ar–H), 8.34 (dd, *J* = 1.0 and 7.2 Hz, 1H, Ar–H), 8.29–8.23 (m, 2H, Ar–H), 7.73–7.66 (m, 2H, Ar–H and CONH), 7.48 (d, *J* = 9.1 Hz, 1H, Ar–H), 6.94 (dd, *J* = 2.6 and 9.1 Hz, 1H, Ar–H), 6.88 (d, *J* = 2.7 Hz, 1H, Ar–H), 3.66 (t, *J* = 4.4 Hz, 4H, morpholine-OCH_2_ × 2), 3.01–2.92 (m, 6H, NCH_2_ and morpholine-NCH_2_ × 2), 1.74–1.56 (m, 5H, cyclohexyl-CH_2_ × 2 and CH_2_ × 1/2), 1.47–1.34 (m, 1H, cyclohexyl-CH), 1.26–1.08 (m, 3H, cyclohexyl-CH_2_ and CH_2_ × 1/2), 0.95–0.80 (m, 2H, cyclohexyl-CH_2_). ^13^C NMR (100 MHz, DMSO-*d*_6_): δ 167.97, 151.82, 146.90, 143.01, 137.29, 135.41, 134.73, 132.42, 129.72, 128.80, 126.02, 123.97, 123.01, 120.71, 118.79, 114.89, 66.41, 48.75, 45.81, 37.66, 31.01, 26.51, 25.86. HPLC: Method A; *r*_t_ = 9.990 min. HRMS (ESI) calculated for C_27_H_32_N_4_O_4_S [M + H]^+^ 509.2215, found 509.2215.

#### *N*-(Cyclohexylmethyl)-2-[(quinolin-8-ylsulfonyl)­amino]-5-thiomorpholin-4-ylbenzamide (**9**)

Following the general procedure (method E), starting from intermediate **63** and using 8-quinolinesulfonyl chloride, after stirring for 1 h, compound **9** was obtained after filtration and purification by flash chromatography column eluting with Acetone/MeOH (98:2) as a solid in 47% yield (0.29 g). ^1^H NMR (400 MHz, DMSO-*d*_6_): δ = 10.93 (s, 1H, NHSO_2_), 9.00 (dd, *J* = 1.6 and 4.2 Hz, 1H, Ar–H), 8.50 (dd, *J* = 1.5 and 8.4 Hz, 1H, Ar–H), 8.35 (dd, *J* = 1.1 and 7.3 Hz, 1H, Ar–H), 8.30–8.24 (m, 2H, Ar–H), 7.74–7.67 (m, 2H, Ar–H and CONH), 7.46 (d, *J* = 9.0 Hz, 1H, Ar–H), 6.93 (dd, *J* = 2.7 and 9.1 Hz, 1H, Ar–H), 6.88–6.85 (m, 1H, Ar–H), 3.39–3.35 (m, 4H, thiomorpholine-NCH_2_ × 2), 2.95 (t, *J* = 6.2 Hz, 2H, NCH_2_), 2.63–2.57 (m, 4H, thiomorpholine-SCH_2_ × 2), 1.74–1.56 (m, 5H, cyclohexyl-CH_2_ × 2 and CH_2_ × 1/2), 1.47–1.34 (m, 1H, cyclohexyl-CH), 1.27–1.08 (m, 3H, cyclohexyl-CH_2_ and CH_2_ × 1/2), 0.94–0.80 (m, 2H, cyclohexyl-CH_2_). ^13^C NMR (100 MHz, DMSO-*d*_6_): δ = 167.97, 151.83, 146.82, 143.01, 137.30, 135.50, 134.72, 132.33, 129.53, 128.81, 126.04, 124.18, 123.02, 120.83, 120.21, 116.41, 51.53, 45.78, 37.67, 31.01, 26.51, 26.29, 25.86. HPLC: Method A; *r*_t_ = 8.9630 min. HRMS (ESI) calculated for C_27_H_32_N_4_O_3_S_2_ [M + H]^+^ 525.1989, found 525.1980.

#### *N*-(Cyclohexylmethyl)-5-(4-methylpiperazin-1-yl)-2-[(quinolin-8-ylsulfonyl)­amino]­benzamide (**10**)

Following the general procedure (method E), starting from intermediate **64** and using 8-quinolinesulfonyl chloride, after stirring for 1 h, compound **10** was obtained after extraction and purification by flash chromatography column eluting with CHCl_3_/MeOH (95:5) as a yellow solid in 46% yield (0.18 g). ^1^H NMR (400 MHz, DMSO-*d*_6_): δ = 10.98 (s, 1H, NHSO_2_), 9.05 (dd, J = 1.4 and 4.0 Hz, 1H, Ar–H), 8.55 (dd, *J* = 1.2 and 8.3 Hz, 1H, Ar–H), 8.42–8.37 (m, 1H, Ar–H), 8.36–8.28 (m, 2H, Ar–H), 7.78–7.71 (m, 2H, Ar–H), 7.51 (d, *J* = 9.0 Hz, 1H, Ar–H), 6.98 (dd, *J* = 2.4 and 9.0 Hz, 1H, Ar–H), 6.95–6.91 (m, 1H, Ar–H), 3.06 (bs, 4H, piperazine-NCH_2_ × 2), 3.00 (t, *J* = 6.2 Hz, 2H, NCH_2_), 2.43 (bs, 4H, piperazine-NCH_2_ × 2), 2.23 (s, 3H, NCH_3_), 1.78–1.63 (m, 5H, cyclohexyl- CH_2_ × 2 and CH_2_ × 1/2), 1.53–1.40 (m, 1H, cyclohexyl-CH), 1.31–1.14 (m, 3H, cyclohexyl-CH_2_ and CH_2_ × 1/2), 0.99–0.85 (m, 2H, cyclohexyl-CH_2_). ^13^C NMR (100 MHz, DMSO-*d*_6_): δ = 168.00, 151.79, 146.83, 143.03, 137.27, 135.55, 134.66, 132.37, 129.53, 128.80, 126.01, 123.96, 122.98, 120.73, 118.98, 115.10, 54.92, 48.39, 46.15, 45.80, 37.65, 31.02, 26.51, 25.86. HPLC: Method A; *r*_t_ = 4.167 min. HRMS (ESI) calculated for C_28_H_35_N_5_O_3_S [M + H]^+^ 522.2534, found 522.2530.

#### *N*-(Cyclohexylmethyl)-5-(1,4-dioxa-8-azaspiro­[4.5]­dec-8-yl)-2-[(quinolin-8-ylsulfonyl)­amino]­benzamide (**11**)

Following the general procedure (method E), starting from intermediate **65** and using 8-quinolinesulfonyl chloride, after stirring for 1 h, compound **11** was obtained after filtration and purification by crystallization by Cy/EtOAc as a white solid in 12% yield (0.07 g). ^1^H NMR (400 MHz, CDCl_3_): δ = 9.40 (s, 1H, NHSO_2_), 9.06 (dd, *J* = 1.7 and 4.2 Hz, 1H, Ar–H), 8.28 (dd, *J* = 1.3 and 7.3 Hz, 1H, Ar–H), 8.19 (dd, *J* = 1.6 and 8.4 Hz, 1H, Ar–H), 7.96 (dd, *J* = 1.2 and 8.2 Hz, 1H, Ar–H), 7.55–7.46 (m, 2H, Ar–H), 6.93 (d, *J* = 2.9 Hz, 1H, Ar–H), 6.89 (d, *J* = 8.9 Hz, 1H, Ar–H), 6.63 (dd, *J* = 2.9 and 9.0 Hz, 1H, Ar–H), 6.43 (t, *J* = 5.5 Hz, 1H, CONH), 3.88 (s, 4H, “dioxolane”-OCH_2_ × 2), 3.16–3.11 (m, 4H, “piperidine”-NCH_2_ × 2), 3.06 (t, *J* = 6.4 Hz, 2H, NCH_2_), 1.71–1.63 (m, 8H, “piperidine”-CH_2_ × 2 and cyclohexyl-CH_2_ × 2), 1.63–1.56 (m, 1H, cyclohexyl-CH_2_ × 1/2), 1.48–1.37 (m, 1H, cyclohexyl-CH), 1.24–1.03 (m, 3H, cyclohexyl-CH_2_ and CH_2_ × 1/2), 0.95–0.82 (m, 2H, cyclohexyl-CH_2_). ^13^C NMR (100 MHz, CDCl_3_): δ = 168.01, 151.81, 146.59, 143.01, 137.29, 135.51, 134.70, 132.34, 129.34, 128.80, 126.02, 124.07, 123.01, 120.78, 119.77, 115.84, 106.62, 64.15, 47.32, 45.77, 37.66, 34.43, 31.01, 26.51, 25.86. HPLC: Method A; *r*_t_ = 8.330 min. HRMS (ESI) calculated for C_30_H_36_N_4_O_5_S [M + H]^+^565.2479, found 565.2474.

#### *N*-(Cyclohexylmethyl)-5-(4-phenylpiperidin-1-yl)-2-[(quinolin-8-ylsulfonyl)­amino]­benzamide (**12**)

Following the general procedure (method E), starting from intermediate **66** and using 8-quinolinesulfonyl chloride, after stirring for 1 h, compound **12** was obtained after filtration and purification by crystallization by Cy/EtOAc as a white solid in 16% yield (0.07 g). ^1^H NMR (400 MHz, CDCl_3_): δ = 9.42 (s, 1H, NHSO_2_), 9.07 (dd, *J* = 1.5 and 4.1 Hz, 1H, Ar–H), 8.31–8.27 (m, 1H, Ar–H), 8.19 (dd, *J* = 1.5 and 8.3 Hz, 1H, Ar–H), 7.90–7.94 (m, 1H, Ar–H), 7.55–7.48 (m, 2H, Ar–H), 7.26–7.20 (m, 2H, Ar–H), 7.13–7.10 (m, 3H, Ar–H), 6.99 (d, *J* = 2.4 Hz, 1H, Ar–H), 6.92 (d, *J* = 8.9 Hz, 1H, Ar–H), 6.68 (dd, *J* = 2.7 and 8.9 Hz, 1H, Ar–H), 6.46 (t, *J =* 5.4 Hz, 1H, CONH), 3.65–3.54 (m, 2H, piperidine-NCH_2_), 3.07 (t, *J* = 6.4 Hz, 2H, NCH_2_), 2.72–2.63 (m, 2H, piperidine-NCH_2_), 2.58–2.47 (m, 1H, piperidine-CH), 1.87–1.80 (m, 2H, piperidine-CH_2_), 1.78–1.56 (m, 7H, piperidine-CH_2_ and cyclohexyl-CH_2_ × 2 and CH_2_ × 1/2), 1.48–1.36 (m, 1H, cyclohexyl-CH), 1.25–1.04 (m, 3H, cyclohexyl-CH_2_ and CH_2_ × 1/2), 0.96–0.83 (m, 2H, cyclohexyl-CH_2_). ^13^C NMR (100 MHz, DMSO-*d*_6_): 168.07, 151.82, 147.34, 146.39, 143.03, 137.29, 135.51, 134.70, 132.38, 129.30, 128.84, 128.81, 127.16, 126.57, 126.03, 124.06, 123.01, 120.78, 119.63, 115.77, 49.74, 45.78, 41.79, 37.67, 33.15, 31.02, 26.52, 25.86. HPLC: Method A; *r*_t_ = 9.990 min. HRMS (ESI) calculated for C_34_H_38_N_4_O_3_S [M + H]^+^ 583.2737, found 583.2732.

#### *N*-(Cyclohexylmethyl)-5-(4-hydroxypiperidin-1-yl)-2-[(quinolin-8-ylsulfonyl)­amino]­benzamide (**13**)

Following the general procedure (method E), starting from intermediate **67** and using 8-quinolinesulfonyl chloride, after stirring for 1 h, compound **13** was obtained after filtration and purification by flash chromatography column eluting with CHCl_3_/MeOH (97:3) as a yellow solid in 39% yield (0.25 g).^1^H NMR (400 MHz, DMSO-*d*_6_): δ = 10.95 (s, 1H, NHSO_2_), 9.05 (dd, *J* = 1.8 and 4.2 Hz, 1H, Ar–H), 8.55 (dd, *J* = 1.7 and 8.4 Hz, 1H, Ar–H), 8.39 (dd, *J* = 1.4 and 7.4 Hz, 1H, Ar–H), 8.33–8.28 (m, 2H, Ar–H), 7.79–7.72 (m, 2H, Ar–H and CONH), 7.50 (d, *J* = 9.0 Hz, 1H, Ar–H), 6.98 (dd, *J* = 2.8 and 9.1 Hz, 1H, Ar–H), 6.94–6.91 (m, 1H, Ar–H), 4.68 (d, *J* = 4.2 Hz, 1H, OH), 3.65–3.55 (m, 1H, piperidine-CH), 3.47–3.40 (m, 2H, piperidine-NCH_2_), 2.99 (t, *J* = 6.3 Hz, 2H, NCH_2_), 2.80–2.71 (m, 2H, piperidine-NCH_2_), 1.84–1.64 (m, 7H, piperidine-CH_2_ and cyclohexyl-CH_2_ × 2 and CH_2_ × 1/2), 1.53–1.37 (m, 3H, piperidine-CH_2_ and cyclohexyl-CH), 1.31–1.16 (m, 3H, cyclohexyl-CH_2_ and CH_2_ × 1/2), 0.99–0.86 (m, 2H, cyclohexyl-CH_2_). ^13^C NMR (100 MHz, DMSO-*d*_6_): 168.06, 151.81, 147.09, 143.02, 137.28, 135.50, 134.68, 132.36, 129.01, 128.79, 126.02, 124.13, 123.00, 120.84, 119.40, 115.55, 66.19, 46.84, 45.76, 37.66, 34.24, 31.01, 26.52, 25.86. HPLC: Method A; *r*_t_ = 6.720 min. HRMS (ESI) calculated for C_28_H_34_N_4_O_4_S [M + H]^+^ 523.2374, found 523.2372.

#### *N*-(Cyclohexylmethyl)-5-(octahydroisoquinolin-2­(1*H*)-yl)-2-[(quinolin-8-ylsulfonyl)­amino]­benzamide (**14**)

Following the general procedure (method E), starting from intermediate **68** and using 8-quinolinesulfonyl chloride, after stirring for 1 h, compound **14** was obtained after filtration and purification by flash chromatography column eluting with CHCl_3_/Acetone (98:2) as a solid in 45% yield (0.31 g). ^1^H NMR (400 MHz, DMSO-*d*_6_): δ = 10.84 (s, 1H, NHSO_2_), 9.02–8.98 (m, 1H, Ar–H), 8.52–8.47 (m, 1H, Ar–H), 8.35–8.30 (m, 1H, Ar–H), 8.27–8.21 (m, 2H, Ar–H), 7.72–7.67 (m, 2H, Ar–H and CONH), 7.43 (d, *J* = 9.1 Hz, 1H, Ar–H), 6.91 (dd, *J* = 2.4 and 9.2 Hz, 1H, Ar–H), 6.84 (d, *J* = 2.5 Hz, 1H, Ar–H), 3.62–3.54 (m, 1H, perhydroisoquinoline-NCH_2_ × 1/2), 3.46–3.39 (m, 1H, perhydroisoquinoline-NCH_2_ × 1/2), 3.01–2.86 (m, 2H, NCH_2_), 2.21–2.12 (m, 1H, perhydroisoquinoline-NCH_2_ × 1/2), 1.73–1.48 (m, 10H, aliphatic-H), 1.46–1.34 (m, 1H, cyclohexyl-CH), 1.29–1.05 (m, 8H, aliphatic-H), 1.01–0.81 (m, 5H, aliphatic-H). ^13^C NMR (100 MHz, DMSO-*d*_6_): δ = 168.08, 151.81, 147.31, 143.02, 137.28, 135.51, 134.67, 132.34, 128.79 (2C), 126.01, 124.33, 123.00, 120.95, 119.30, 115.51, 55.46, 49.64, 45.71, 41.41, 41.38, 37.69, 32.87, 32.57, 31.00, 30.21, 26.51, 26.32, 25.95, 25.85. HPLC: Method A; *r*_t_ = 10.457 min. HRMS (ESI) calculated for C_32_H_40_N_4_O_3_S [M + H]^+^ 561.2894, found 561.2890.

#### *N*-(Cyclohexylmethyl)-5-(3,5-dimethylpiperidin-1-yl)-2-[(quinolin-8-ylsulfonyl)­amino]­benzamide (**15**)

Following the general procedure (method E), starting from intermediate **69** and using 8-quinolinesulfonyl chloride, after stirring for 3 h, compound **15** was obtained after filtration and purification by flash chromatography column eluting with CHCl_3_/MeOH (99:1) as a solid in 60% yield (0.22 g). ^1^H NMR (400 MHz, DMSO-*d*_6_): δ = 11.59 (s, 1H, NHSO_2_), 9.79 (dd, *J* = 1.6 and 4.2 Hz, 1H, Ar–H), 9.29 (dd, *J* = 1.6 and 8.4 Hz, 1H, Ar–H), 9.12 (dd, *J* = 1.2 and 4.7 Hz, 1H, Ar–H), 9.07–8.99 (m, 2H, CONH and Ar–H), 8.53–8.45 (m, 2H, Ar–H), 8.21 (d, *J* = 9.1 Hz, 1H, Ar–H), 7.71 (dd, *J* = 2.7 and 9.1 Hz, 1H, Ar–H), 7.63 (d, *J* = 2.7 Hz, 1H, Ar–H), 4.33–4.25 (m, 2H, piperidine-NCH_2_), 3.73 (t, *J* = 6.2 Hz, 2H, NCH_2_), 2.83 (t, *J* = 11.7 Hz, 2H, piperidine-NCH_2_), 2.53–2.31 (m, 9H, aliphatic-H), 2.26–2.14 (m, 1H, aliphatic-H), 2.05–1.87 (m, 2H, aliphatic-H), 1.74–1.64 (m, 2H, aliphatic-H), 1.63 (d, *J* = 6.6 Hz, 6H, piperidine-NCH_3_ × 2), 1.45–1.33 (m, 1H, aliphatic-H). ^13^C NMR (100 MHz, DMSO-*d*_6_): δ = 168.08, 151.82, 147.11, 143.01, 137.28, 135.50, 134.67, 132.33, 128.80, 128.69, 126.02, 124.52, 123.01, 121.06, 119.35, 115.65, 56.41, 45.67, 42.02, 37.72, 30.99, 30.62, 26.51, 25.85, 19.61. HPLC: Method A; *r*_t_ = 9.877 min. HRMS (ESI) calculated for C_30_H_38_N_4_O_3_S [M + H]^+^ 535.2738, found 535.2731.

#### *N*-(Cyclohexylmethyl)-5-(4-methylpiperidin-1-yl)-2-[(quinolin-8-ylsulfonyl)­amino]­benzamide (**16**)

Following the general procedure (method E), starting from intermediate **70** and using 8-quinolinesulfonyl chloride, after stirring for 1 h, compound **16** was obtained after filtration and purification by flash chromatography column eluting with CHCl_3_/Acetone (98:2) as a solid in 47% yield (0.19 g). ^1^H NMR (400 MHz, DMSO-*d*_6_): δ = 10.89 (s, 1H, NHSO_2_), 8.99 (dd, *J* = 1.5 and 4.1 Hz, 1H, Ar–H), 8.49 (dd, *J* = 1.3 and 8.4 Hz, 1H, Ar–H), 8.35–8.31 (m, 1H, Ar–H), 8.28–8.22 (m, 2H, Ar–H), 7.72–7.66 (m, 2H, Ar–H and CONH), 7.44 (d, *J* = 9.1 Hz, 1H, Ar–H), 6.92 (dd, *J* = 2.5 and 9.1 Hz, 1H, Ar–H), 6.86 (d, *J* = 2.6 Hz, 1H, Ar–H), 3.55–3.47 (m, 2H, piperidine-NCH_2_), 2.94 (t, *J* = 6.3 Hz, 2H, NCH_2_), 2.48–2.45 (m, 2H, piperidine-NCH_2_), 1.72–1.57 (m, 7H, aliphatic-H), 1.47–1.35 (m, 1H, cyclohexyl-CH), 1.26–1.05 (m, 6H, aliphatic-H), 0.89 (d, *J* = 6.5 Hz, 3H, piperidine-CH_3_), 0.86–0.80 (m, 2H, aliphatic-H). ^13^C NMR (100 MHz, DMSO-*d*_6_): δ = 168.07, 151.80, 147.41, 143.02, 137.27, 135.51, 134.67, 132.36, 129.01, 128.79, 126.01, 124.12, 123.00, 120.81, 119.44, 115.58, 49.20, 45.75, 37.66, 33.93, 31.01, 30.42, 26.51, 25.85, 22.15. HPLC: Method A; *r*_t_ = 8.927 min. HRMS (ESI) calculated for C_29_H_36_N_4_O_3_S [M + H]^+^ 521.2581, found 521.2578.

#### *N*-(Cyclohexylmethyl)-2-[(isoquinolin-5-ylsulfonyl)­amino]-5-piperidin-1-ylbenzamide (**28**)

Following the general procedure (method E), starting from intermediate **61** and using isoquinoline-5-sulfonyl chloride, after stirring for 1 h, compound **28** was obtained after extraction and purification by crystallization by Cy/EtOAc as a gray solid in 33% yield (0.13 g). ^1^H NMR (400 MHz, CDCl_3_): δ = 10.33 (s, 1H, NHSO_2_), 9.25 (s, 1H, Ar–H), 8.62 (d, *J* = 6.1 Hz, 1H, Ar–H), 8.39–8.36 (m, 1H, Ar–H), 8.32 (dd, *J* = 1.1 and 7.4 Hz, 1H, Ar–H), 8.12–8.06 (m, 1H, Ar–H), 7.61–7.51 (m, 2H, Ar–H), 6.95 (dd, *J* = 2.8 and 9.0 Hz, 1H, Ar–H), 6.63 (d, *J* = 2.7 Hz, 1H, Ar–H), 5.56 (bs, 1H, CONH), 3.02 (t, *J* = 5.3 Hz, 4H, piperidine-NCH_2_ × 2), 2.79 (t, *J =* 6.4 Hz, 2H, NCH_2_), 1.75–1.63 (m, 6H, piperidine-CH_2_ × 3), 1.62–1.51 (m, 5H, cyclohexyl-CH_2_ × 2 and CH_2_ × 1/2), 1.34–1.23 (m, 1H, cyclohexyl-CH), 1.22–1.09 (m, 3H, cyclohexyl-CH_2_ and CH_2_ × 1/2), 0.89–0.76 (m, 2H, cyclohexyl-CH_2_). ^13^C NMR (100 MHz, CDCl_3_): δ = 168.15, 152.81, 149.18, 144.99, 134.35, 133.72, 133.33, 131.26, 128.94, 128.76, 125.75, 124.69, 124.09, 120.30, 117.65, 114.17, 50.78, 45.85, 37.57, 30.80, 26.92, 26.25, 25.69, 23.92. HPLC: Method A; *r*_t_ = 8.207 min. HRMS (ESI) calculated for C_28_H_34_N_4_O_3_S [M + H]^+^ 507.2425, found 507.2425.

#### *N*-(Cyclohexylmethyl)-5-piperidin-1-yl-2-[(quinoxalin-5-ylsulfonyl)­amino]­benzamide (**29**)

Following the general procedure (method E), starting from intermediate **61** and using quinoxaline-5-sulfonyl chloride, after stirring for 2 h, compound **29** was obtained after filtration and purification by flash chromatography column eluting with CHCl_3_/MeOH (99:1) as a green solid in 79% yield (0.42 g). ^1^H NMR (400 MHz, CDCl_3_): δ = 9.84 (s, 1H, NHSO_2_), 9.06 (d, *J* = 1.8 Hz, 1H, Ar–H), 8.96 (d, *J* = 1.8 Hz, 1H, Ar–H), 8.40 (dd, *J* = 1.4 and 7.3 Hz, 1H, Ar–H), 8.28 (dd, *J* = 1.3 and 8.4 Hz, 1H, Ar–H), 7.81–7.76 (m, 1H, Ar–H), 7.33 (d, *J* = 8.8 Hz, 1H, Ar–H), 6.84–6.78 (m, 2H, Ar–H), 5.97 (bt, 1H, CONH), 3.04–2.96 (m, 6H, piperidine-NCH_2_ × 2 and NCH_2_), 1.78–1.60 (m, 9H, piperidine-CH_2_ × 3 and cyclohexyl-CH_2_ and CH_2_ × 1/2), 1.56–1.49 (m, 2H, cyclohexyl-CH_2_), 1.45–1.33 (m, 1H, cyclohexyl-CH), 1.29–1.10 (m, 3H, cyclohexyl-CH_2_ and CH_2_ × 1/2), 0.97–0.84 (m, 2H, cyclohexyl-CH_2_). ^13^C NMR (100 MHz, CDCl_3_): δ = 167.91, 149.00, 145.86, 145.46, 142.94, 138.87, 136.93, 134.91, 131.96, 128.74, 127.78, 126.95, 123.58, 119.53, 115.03, 50.61, 45.99, 37.77, 30.88, 26.31, 25.74, 25.69, 23.97. HPLC: Method A; *r*_t_ = 7.717 min. HRMS (ESI) calculated for C_27_H_33_N_5_O_3_S [M + H]^+^ 508.2377, found 508.2383.

#### 2-[(2,1,3-Benzoxadiazol-4-ylsulfonyl)­amino]-*N*-(cyclohexylmethyl)-5-piperidin-1-ylbenzamide (**30**)

Following the general procedure (method E), starting from intermediate **61** and using benzoxadiazole-4-sulfonyl chloride, after stirring for 24 h, compound **30** was obtained after filtration and purification by flash chromatography column eluting with CHCl_3_/MeOH (98:2) as an orange solid in 46% yield (0.21 g). ^1^H NMR (400 MHz, CDCl_3_): δ = 10.46 (s, 1H, NHSO_2_), 8.01–7.99 (m, 1H, Ar–H), 7.98–7.96 (m, 1H, Ar–H), 7.58 (d, *J* = 9.0 Hz, 1H, Ar–H), 7.45 (dd, *J =* 6.8 and 9.0 Hz, 1H, Ar–H), 6.95 (dd, *J =* 2.8 and 9.0 Hz, 1H, Ar–H), 6.74 (d, *J* = 2.8 Hz, 1H, Ar–H), 5.82 (bs, 1H, CONH), 3.08–3.02 (m, 6H, piperidine-NCH_2_ × 2 and NCH_2_), 1.79–1.62 (m, 9H, piperidine-CH_2_ × 3 and cyclohexyl-CH_2_ and CH_2_ × 1/2), 1.56–1.52 (m, 2H, cyclohexyl-CH_2_), 1.50–1.39 (m, 1H, cyclohexyl-CH), 1.32–1.10 (m, 3H, cyclohexyl-CH_2_ and CH_2_ × 1/2), 0.98–0.86 (m, 2H, cyclohexyl-CH_2_). ^13^C NMR (100 MHz, CDCl_3_): δ = 168.15, 149.22, 149.16, 144.32, 133.88, 130.17, 129.08, 128.25, 124.67, 123.31, 121.27, 120.18, 114.31, 50.70, 46.06, 37.66, 30.86, 26.31, 25.72 (2C), 23.95. HPLC: Method A; *r*_t_ = 8.987 min. HRMS (ESI) calculated for C_25_H_31_N_5_O_4_S [M + H]^+^ 498.2170, found 498.2161.

#### 2-{[(3-Cyanophenyl)­sulfonyl]­amino}-*N*-(cyclohexylmethyl)-5-piperidin-1-ylbenzamide (**32**)

Following the general procedure (method E), starting from intermediate **61** and using 3-cyanobenzylsulfonyl chloride, after stirring for 1 h, compound **32** was obtained after filtration and purification by flash chromatography column eluting with CHCl_3_/MeOH (99.5:0.5) as a gray solid in 50% yield (0.24 g). ^1^H NMR (400 MHz, CDCl_3_): δ = 10.20 (s, 1H, NHSO_2_), 7.98–7.93 (m, 2H, Ar–H), 7.73 (dt, *J* = 1.3 and 7.7 Hz, 1H, Ar–H), 7.58 (d, *J* = 9.0 Hz, 1H, Ar–H), 7.54–7.48 (m, 1H, Ar–H), 7.02 (dd, *J* = 2.8 and 9.0 Hz, 1H, Ar–H), 6.78 (d, *J* = 2.7 Hz, 1H, Ar–H), 5.91 (bs, 1H, CONH), 3.14–3.07 (m, 6H, piperidine-NCH_2_ × 2 and NCH_2_), 1.79–1.62 (m, 9H, piperidine-CH_2_ × 3 and cyclohexyl-CH_2_ and CH_2_ × 1/2), 1.62–1.54 (m, 2H, cyclohexyl-CH_2_), 1.52–1.39 (m, 1H, cyclohexyl-CH), 1.31–1.11 (m, 3H, cyclohexyl-CH_2_ and CH_2_ × 1/2), 0.99–0.86 (m, 2H, cyclohexyl-CH_2_). ^13^C NMR (100 MHz, CDCl_3_): 168.28, 149.55, 141.28, 135.45, 131.36, 130.83, 129.71, 128.46, 124.98, 124.49, 120.47, 117.15, 113.91, 113.19, 50.57, 46.15, 37.74, 30.86, 26.26, 25.72, 25.71, 23.94. HPLC: a gradient consisting of ACN and water containing 0.1% formic acid, with a linear increase of ACN from 30% to 100% over 10 min, followed by 100% ACN; flow: 0.5 mL/min.; *r*_t_ = 8.560 min. HRMS (ESI) calculated for C_26_H_32_N_4_O_3_S [M + H]^+^ 481.2268, found 481.2270.

#### *N*-(Cyclohexylmethyl)-5-piperidin-1-yl-2-[(pyridin-3-ylsulfonyl)­amino]­benzamide (**33**)

Following the general procedure (method E), starting from intermediate **61** and using pyridine-3-sulfonyl chloride, after stirring for 48 h, compound **33** was obtained after filtration and purification by flash chromatography column eluting with CHCl_3_/MeOH (99:1) as a gray solid in 33% yield (0.19 g). ^1^H NMR (400 MHz, CDCl_3_): δ = 9.98 (s, 1H, NHSO_2_), 8.77 (d, *J* = 2.0 Hz, 1H, Ar–H), 8.67 (dd, *J* = 1.5 and 4.8 Hz, 1H, Ar–H), 8.03 (dt, *J* = 1.8 and 8.0 Hz, 1H, Ar–H), 7.61 (d, *J* = 9.0 Hz, 1H, Ar–H), 7.32 (dd, *J* = 4.8 and 8.0 Hz, 1H, Ar–H), 7.02 (dd, *J* = 2.8 and 9.0 Hz, 1H, Ar–H), 6.76 (d, *J* = 2.7 Hz, 1H, Ar–H), 5.81 (bs, 1H, CONH), 3.10 (t, *J =* 5.3 Hz, 4H, piperidine-NCH_2_ × 2), 3.03 (t, *J* = 6.4 Hz, 2H, NCH_2_), 1.80–1.60 (m, 11H, piperidine-CH_2_ × 3 and cyclohexyl-CH_2_ × 2 and CH_2_ × 1/2), 1.48–1.37 (m, 1H, cyclohexyl-CH), 1.31–1.11 (m, 3H, cyclohexyl-CH_2_ and CH_2_ × 1/2), 0.97–0.84 (m, 2H, cyclohexyl-CH_2_). ^13^C NMR (100 MHz, CDCl_3_): 168.28, 152.87, 149.61, 148.15, 135.95, 135.01, 128.29, 125.63, 125.35, 123.39, 120.32, 113.97, 50.59, 46.14, 37.67, 30.87, 26.27, 25.73, 25.72, 23.94. HPLC: Method A; *r*_t_ = 8.270. HRMS (ESI) calculated for C_24_H_32_N_4_O_3_S [M + H]^+^ 457.2268, found 457.2264.

#### *N*-(Cyclohexylmethyl)-2-{[(2-nitrophenyl)­sulfonyl]­amino}-5-piperidin-1-ylbenzamide (**71**)

Following the general procedure (method E), starting from intermediate **61** and using 2-nitrobenzenesulfonyl chloride, after stirring for 24 h, compound **71** was obtained after filtration and purification by flash chromatography column eluting with CH_2_Cl_2_/MeOH (99:1) as an orange solid in 30% yield (0.14 g). ^1^H NMR (400 MHz, CDCl_3_): δ = 9.49 (s, 1H, NHSO_2_), 7.94 (dd, *J* = 1.4 and 7.8 Hz, 1H, Ar–H), 7.78 (dd, *J* = 1.3 and 7.9 Hz, 1H, Ar–H), 7.66 (td, *J* = 1.5 and 7.7 Hz, 1H, Ar–H), 7.59 (td, *J* = 1.4 and 7.7 Hz, 1H, Ar–H), 7.43 (d, *J* = 8.9 Hz, 1H, Ar–H), 6.94 (dd, *J* = 2.8 and 9.0 Hz, 1H, Ar–H), 6.90 (d, *J* = 2.7 Hz, 1H, Ar–H), 6.10 (bs, 1H, CONH), 3.15 (t, *J* = 6.4 Hz, 2H, NCH_2_), 3.14–3.08 (m, 4H, piperidine-NCH_2_ × 2), 1.76–1.63 (m, 8H, piperidine-CH_2_ × 3 and cyclohexyl-CH_2_), 1.61–1.54 (m, 3H, cyclohexyl-CH_2_ and CH_2_ × 1/2), 1.54–1.43 (m, 1H, cyclohexyl-CH), 1.29–1.09 (m, 3H, cyclohexyl-CH_2_ and CH_2_ × 1/2), 0.98–0.86 (m, 2H, cyclohexyl-CH_2_).

#### 2-{[(2-Aminophenyl)­sulfonyl]­amino}-*N*-(cyclohexylmethyl)-5-piperidin-1-ylbenzamide (**31**)

Following the general procedure (method D), starting from intermediate **71** dissolved in EtOH, after stirring for 1 h, compound **31** was obtained after purification by flash column eluting with CH_2_Cl_2_/MeOH (95:5) as a solid in 43% yield (0.08 g). ^1^H NMR (400 MHz, CDCl_3_): δ = 9.70 (s, 1H, NHSO_2_), 7.49 (d, *J* = 9.0 Hz, 1H, Ar–H), 7.42 (dd, *J* = 1.4 and 8.0 Hz, 1H, Ar–H), 7.20–7.15 (m, 1H, Ar–H), 6.95 (dd, *J* = 2.8 and 9.0 Hz, 1H, Ar–H), 6.84–6.80 (m, 1H, Ar–H), 6.62–6.54 (m, 2H, Ar–H), 5.83 (bt, 1H, CONH), 4.94 (s, 2H, NH_2_), 3.13–3.03 (m, 6H, piperidine-NCH_2_ × 2 and NCH_2_), 1.80–1.65 (m, 9H, piperidine-CH_2_ × 3 and cyclohexyl-CH_2_ and CH_2_ × 1/2), 1.60–1.54 (m, 2H, cyclohexyl-CH_2_), 1.52–1.43 (m, 1H, cyclohexyl-CH), 1.32–1.13 (m, 3H, cyclohexyl-CH_2_ and CH_2_ × 1/2), 1.02–0.88 (m, 2H, cyclohexyl-CH_2_). ^13^C NMR (100 MHz, CDCl_3_): 168.37, 148.32, 145.50, 133.96, 129.97, 129.62, 125.99, 124.65, 120.32, 120.13, 117.12, 116.81, 115.20, 51.35, 46.24, 37.66, 30.93, 26.33, 25.80, 25.56, 23.80. HPLC: Method A; *r*_t_ = 8.330 min. HRMS (ESI) calculated for C_25_H_34_N_4_O_3_S [M + H]^+^ 471.2425, found 471.2426.

### General Procedure for the Amide Coupling Reaction (Method F)

Under N_2_ atmosphere, to a solution of the appropriate carboxylic acid (1.00 equiv) in DMSO or DMF dry as indicated for each compound (9 mL per mmol), TBTU (1.80 equiv), DIPEA (4.00 equiv), and the appropriate amine (1.50 equiv) were added, and the reaction was stirred at rt for 1–48 h. Upon completion, the reaction mixture was poured into ice/water, and the pH was adjusted as necessary. The mixture was either filtered (if a precipitate formed) or extracted with EtOAc (x3), and the combined organic layers were washed with brine, dried over Na_2_SO_4_ and evaporated to dryness. The obtained crude product was purified as described below for each compound.

#### *N*-(2-{[(Cyclohexylmethyl)­amino]­carbonyl}-4-piperidin-1-ylphenyl)­quinoline-8-carboxamide (**34**)

Following the general procedure (method F), starting from 8-quinolinecarboxylic acid dissolved in dry DMSO and using the amine derivative **61**, after stirring for 22 h and basifying with a saturated solution of Na_2_CO_3_ (pH = 9), compound **34** was obtained after filtration and purification by flash chromatography column eluting with CHCl_3_/MeOH (99.5:0.5) as a yellow solid in 30% yield (0.12 g). ^1^H NMR (400 MHz, CDCl_3_): δ = 13.46 (s, 1H, NHCO), 9.04 (dd, *J* = 1.8 and 4.2 Hz, 1H, Ar–H), 8.90 (dd, *J* = 1.4 and 7.4 Hz, 1H, Ar–H), 8.31 (dd, *J* = 1.7 and 8.3 Hz, 1H, Ar–H), 8.01 (dd, *J* = 1.4 and 8.1 Hz, 1H, Ar–H), 7.92 (d, *J* = 8.9 Hz, 1H, Ar–H), 7.71 (t, *J* = 7.7 Hz, 1H, Ar–H), 7.53 (dd, *J* = 4.3 and 8.3 Hz, 1H, Ar–H), 7.12 (d, *J* = 2.8 Hz, 1H, Ar–H), 7.04 (dd, *J* = 2.8 and 8.9 Hz, 1H, Ar–H), 6.47 (bs, 1H, CONH), 3.22–3.15 (m, 6H, piperidine-NCH_2_ × 2 and NCH_2_), 1.76–1.67 (m, 4H, piperidine-CH_2_ × 2), 1.63–1.53 (m, 4H, piperidine-CH_2_ and cyclohexyl-CH_2_), 1.48–1.36 (m, 3H, cyclohexyl-CH_2_ and CH_2_ × 1/2), 1.35–1.25 (m, 1H, cyclohexyl-CH), 0.98–0.71 (m, 5H, cyclohexyl-CH_2_ × 2 and CH_2_ × 1/2). ^13^C NMR (100 MHz, CDCl_3_): δ = 168.92, 165.31, 149.73, 149.51, 145.43, 137.57, 133.99, 132.19, 132.15, 128.93, 128.41, 126.85, 126.54 (2C), 121.13, 118.73, 115.73, 50.81, 46.11, 37.89, 30.78, 26.16, 25.74, 25.57, 24.22. HPLC: Method A; *r*_t_ = 6.333 min. HRMS (ESI) calculated for C_29_H_34_N_4_O_2_ [M + H]^+^ 471.2755, found 471.2748.

#### *N*-(Cyclohexylmethyl)-5-piperidin-1-yl-2-[(pyridin-2-ylacetyl)­amino]­benzamide (**35**)

Following the general procedure (method F), starting from 2-pyridineacetic acid dissolved in dry DMSO and using the amine derivative **61**, after stirring for 24 h, compound **35** was obtained after filtration and purification by flash chromatography column eluting with Cy/EtOAc (60:40) as a gray solid in 51% yield (0.19 g). ^1^H NMR (400 MHz, CDCl_3_): δ = 10.88 (s, 1H, NHCO), 8.60–8.52 (m, 2H, Ar–H), 8.38 (d, *J* = 9.3 Hz, 1H, Ar–H), 7.34–7.30 (m, 2H, Ar–H), 7.09–7.03 (m, 2H, Ar–H), 6.32 (bs, 1H, CONH), 3.67 (s, 2H, CH_2_), 3.24 (t, *J* = 6.4 Hz, 2H, NCH_2_), 3.11 (t, *J* = 5.3 Hz, 4H, piperidine-NCH_2_ × 2), 1.81–1.66 (m, 9H, piperidine-CH_2_ × 3 and cyclohexyl-CH_2_ and CH_2_ × 1/2), 1.62–1.48 (m, 3H, cyclohexyl-CH_2_ and CH), 1.33–1.12 (m, 3H, cyclohexyl-CH_2_ and CH_2_ × 1/2), 1.06–0.93 (m, 2H, cyclohexyl-CH_2_). ^13^C NMR (100 MHz, CDCl_3_): δ = 169.06, 167.38, 149.81 (2C), 147.54, 144.01, 131.77, 124.65 (2C), 122.64, 122.27, 120.84, 115.08, 51.67, 46.18, 44.60, 37.94, 30.92, 26.33, 25.79, 25.61, 23.85. HPLC: Method A; *r*_t_ = 3.813 min. HRMS (ESI) calculated for C_26_H_34_N_4_O_2_ [M + H]^+^ 435.2755, found 471.2749.

#### *N*-(Cyclohexylmethyl)-5-piperidin-1-yl-2-[(pyridin-3-ylacetyl)­amino]­benzamide (**36**)

Following the general procedure (method F), starting from 3-pyridineacetic acid dissolved in dry DMSO and using the amine derivative **61**, after stirring for 22 h, compound **36** was obtained after filtration and purification by flash chromatography column eluting with CHCl_3_/MeOH (98:2) as a solid in 32% yield (0.13 g). ^1^H NMR (400 MHz, CDCl_3_): δ = 10.77 (s, 1H, NHCO), 8.62–8.57 (m, 1H, Ar–H), 8.55–8.50 (m, 1H, Ar–H), 8.36 (d, *J* = 9.1 Hz, 1H, Ar–H), 7.76–7.70 (m, 1H, Ar–H), 7.29–7.25 (m, 1H, Ar–H), 7.03 (dd, *J* = 2.7 and 9.1 Hz, 1H, Ar–H), 6.95 (d, *J* = 2.6 Hz, 1H, Ar–H), 6.22 (bs, 1H, Ar–H), 3.68 (s, 2H, CH_2_), 3.24 (t, *J* = 6.4 Hz, 2H, NCH_2_), 3.08 (t, *J* = 5.4 Hz, 4H, piperidine-NCH_2_ × 2), 1.81–1.66 (m, 9H, piperidine-CH_2_ × 3 and cyclohexyl-CH_2_ and CH_2_ × 1/2), 1.61–1.49 (m, 3H, cyclohexyl-CH_2_ and CH), 1.33–1.14 (m, 3H, cyclohexyl-CH_2_ and CH_2_ × 1/2), 1.05–0.93 (m, 2H, cyclohexyl-CH_2_). ^13^C NMR (100 MHz, CDCl_3_): δ = 169.19, 168.12, 150.44, 148.47, 148.24, 136.91, 131.24, 130.71, 123.47, 122.66, 122.41, 120.70, 114.60, 51.30, 46.16, 42.24, 37.93, 30.92, 26.34, 25.80 (2C), 24.01. HPLC: Method B; *r*_t_ = 6.700 min. HRMS (ESI) calculated for C_26_H_34_N_4_O_2_ [M + H]^+^ 435.2755, found 435.2755.

#### *N*-(Cyclohexylmethyl)-5-piperidin-1-yl-2-[(pyridin-4-ylacetyl)­amino]­benzamide (**37**)

Following the general procedure (method F), starting from 4-pyridineacetic acid dissolved in dry DMSO and using the amine derivative **61**, after stirring for 22 h, compound **37** was obtained after filtration and purification by flash chromatography column eluting with CHCl_3_/MeOH (98:2) as a solid in 33% yield (0.29 g). ^1^H NMR (400 MHz, CDCl_3_): δ = 10.87 (s, 1H, NHCO), 8.58–8.54 (m, 2H, Ar–H), 8.39 (d, *J* = 9.3 Hz, 1H, Ar–H), 7.34–7.31 (m, 2H, Ar–H), 7.10–7.04 (m, 2H, Ar–H), 6.28 (bs, 1H, CONH), 3.68 (s, 2H, CH_2_), 3.25 (t, *J* = 6.4 Hz, 2H, NCH_2_), 3.11 (t, *J* = 5.4 Hz, 4H, piperidine-NCH_2_ × 2), 1.80–1.71 (m, 8H, piperidine-CH_2_ × 3 and cyclohexyl-CH_2_), 1.69–1.67 (m, 1H, cyclohexyl-CH), 1.62–1.51 (m, 3H, cyclohexyl-CH_2_ and CH_2_ × 1/2), 1.32–1.14 (m, 3H, cyclohexyl-CH_2_ and CH_2_ × 1/2), 1.05–0.93 (m, 2H, cyclohexyl-CH_2_). ^13^C NMR (100 MHz, CDCl_3_): δ = 169.06, 167.38, 149.81, 147.55, 144.00, 131.76, 124.64, 122.64, 122.27, 120.84, 115.07, 51.67, 46.18, 44.60, 37.94, 30.92, 26.33, 25.79, 25.61, 23.85. HPLC: a gradient consisting of ACN and water containing 0.1% formic acid, with a linear increase of ACN from 20% to 100% over 10 min, followed by 100% ACN; flow: 0.4 mL/min.; *r*_t_ = 4.127 min. HRMS (ESI) calculated for C_26_H_34_N_4_O_2_ [M + H]^+^ 435.2755, found 435.2718.

#### Ethyl 5-Fluoro-2-nitrobenzoate (**72**)

Under N_2_ atmosphere, to a solution of derivative **43** (0.20 g, 1.08 mmol) in dry DMF (4 mL), iodoethane (0.13 mL, 1.62 mmol) and K_2_CO_3_ (0.37 g, 2.70 mmol) were added. After stirring at rt for 5 h, the reaction mixture was poured into ice/water and extracted with EtOAc (×3). The combined organic layers were washed with brine, dried over Na_2_SO_4_, and evaporated under vacuum to give a yellow oil in 52% yield (0.12 g). ^1^H NMR (400 MHz, CDCl_3_): δ = 8.02 (dd, *J* = 4.6 and 9.0 Hz, 1H, H-3), 7.38 (dd, *J* = 2.7 and 7.8 Hz, 1H, H-6), 7.32–7.27 (m, 1H, H-4), 4.41 (q, *J* = 7.1 Hz, 2H, O*CH*_2_CH_3_), 1.37 (t, *J* = 7.1 Hz, 3H, OCH_2_ *CH*_3_).

#### Ethyl 2-Nitro-5-piperidin-1-ylbenzoate (**73**)

Following the general procedure (method B), starting from intermediate **71** and using 5.00 equiv of piperidine, after stirring at 110 °C for 1 h, compound **73** was obtained after filtration as a yellow solid in 82% yield (0.51 g). ^1^H NMR (400 MHz, CDCl_3_): δ = 8.03–7.99 (m, 1H, Ar–H), 6.82–6.80 (m, 1H, Ar–H), 6.80–6.78 (m, 1H, Ar–H), 4.40 (q, *J* = 7.2 Hz, 2H, O*CH*_2_CH_3_), 3.46–3.42 (m, 4H, piperidine-NCH_2_ × 2), 1.72–1.65 (m, 6H, piperidine-CH_2_ × 3), 1.37 (t, *J* = 7.2 Hz, 3H, OCH_2_ *CH*_3_).

#### Ethyl 2-Amino-5-piperidin-1-ylbenzoate (**74**)

Following the general procedure (method D), starting from intermediate **73** dissolved in EtOAc, after stirring for 48 h, compound **74** was obtained as a brown oil in 96% yield (5.01 g). ^1^H NMR (400 MHz, CDCl_3_): δ = 7.47 (d, *J* = 2.5 Hz, 1H, H-6), 7.12–7.05 (m, 1H, H-4), 6.63 (d, *J* = 8.8 Hz, 1H, H-3), 5.40 (bs, 2H, NH_2_), 4.33 (q, *J* = 7.1 Hz, 2H, O*CH*_2_CH_3_), 2.98 (t, *J* = 5.3 Hz, 4H, piperidine-NCH_2_ × 2), 1.78–1.70 (m, 4H, piperidine-CH_2_ × 2), 1.58–1.50 (m, 2H, piperidine-CH_2_), 1.38 (t, *J* = 7.1 Hz, 3H, OCH_2_ *CH*_3_).

#### Ethyl 5-Piperidin-1-yl-2-[(quinolin-8-ylsulfonyl)­amino]­benzoate (**75**)

Following the general procedure (method E), starting from intermediate **74** and using 8-quinolinesulfonyl chloride, after stirring for 5 h, compound **75** was obtained after filtration as a yellow solid in 79% yield (2.80 g). ^1^H NMR (400 MHz, DMSO-*d*_6_) δ = 10.56 (s, 1H, NHSO_2_), 8.99 (dd, *J* = 1.7 and 4.2 Hz, 1H, Ar–H), 8.50 (dd, *J* = 1.6 and 8.4 Hz, 1H, Ar–H), 8.40 (dd, *J* = 1.3 and 7.3 Hz, 1H, Ar–H), 8.28 (dd, *J* = 1.2 and 8.2 Hz, 1H, Ar–H), 7.76–7.67 (m, 2H, Ar–H), 7.49 (d, *J* = 9.1 Hz, 1H, Ar–H), 7.12 (d, *J* = 2.9 Hz, 1H, Ar–H), 7.06 (dd, *J* = 3.0 and 9.2 Hz, 1H, Ar–H), 4.29 (q, *J* = 7.1 Hz, 2H, O*CH*_2_CH_3_), 2.94 (t, *J* = 4.9 Hz, 4H, piperidine-NCH_2_ × 2), 1.55–1.39 (m, 6H, piperidine-CH_2_ × 3), 1.27 (t, *J* = 7.1 Hz, 3H, OCH_2_ *CH*_3_).

#### 5-Piperidin-1-yl-2-[(quinolin-8-ylsulfonyl)­amino]­benzoic Acid (**76**)

To a solution of derivative **75** (0.10 g, 0.23 mmol) in 1,4-dioxane (4 mL), aq. 1 M LiOH (1.14 mL, 1.14 mmol) was added dropwise. After stirring at rt for 12 h, the reaction mixture was poured into ice/water, acidified with 2 M HCl (pH = 5), and extracted with EtOAc (x3). The combined organic layers were washed with brine, dried over Na_2_SO_4_, and evaporated under vacuum to give a yellow solid in 83% yield (0.08 g). ^1^H NMR (400 MHz, DMSO-*d*_6_) δ = 13.65 (bs, 1H, CO_2_H), 11.08 (s, 1H, NHSO_2_), 9.01 (dd, *J* = 1.8 and 4.2 Hz, 1H, Ar–H), 8.55 (dd, *J* = 1.7 and 8.4 Hz, 1H, Ar–H), 8.48 (dd, *J* = 1.3 and 7.3 Hz, 1H, Ar–H), 8.33 (dd, *J* = 1.3 and 8.3 Hz, 1H, Ar–H), 7.82–7.72 (m, 2H, Ar–H), 7.56 (d, *J* = 9.2 Hz, 1H, Ar–H), 7.21 (d, *J* = 3.0 Hz, 1H, Ar–H), 7.09 (dd, *J* = 3.0 and 9.2 Hz, 1H, Ar–H), 2.98 (t, *J* = 4.9 Hz, 4H, piperidine-NCH_2_ × 2), 1.60–1.52 (m, 4H, piperidine-CH_2_ × 2), 1.51–1.45 (m, 2H, piperidine-CH_2_).

#### *N*-Benzyl-5-piperidin-1-yl-2-[(quinolin-8-ylsulfonyl)­amino]­benzamide (**17**)

Following the general procedure (method F), starting from derivative **76** dissolved in dry DMF and using benzylamine, after stirring for 1 h and acidifying with 2 M HCl (pH = 6), compound **17** was obtained after filtration and purification by crystallization by EtOH/DMF as a solid in 46% yield (0.49 g). ^1^H NMR (400 MHz, DMSO-*d*_6_): δ = 11.08 (s, 1H, NHSO_2_), 8.92 (t, *J* = 5.0, 1H, CONH), 8.84 (d, *J* = 3.9 Hz, 1H, Ar–H), 8.53 (d, *J* = 8.5 Hz, 1H, Ar–H), 8.41 (d, *J* = 7.1 Hz, 1H, Ar–H), 8.31 (d, *J* = 8.1 Hz, 1H, Ar–H), 7.76 (t, *J* = 7.9 Hz, 1H, Ar–H), 7.68–7.63 (m, 1H, Ar–H), 7.53 (d, *J* = 8.5 Hz, 1H, Ar–H), 7.45–7.39 (m, 2H, Ar–H), 7.38–7.33 (m, 1H, Ar–H), 7.32–7.28 (m, 2H, Ar–H), 7.03–6.96 (m, 2H, Ar–H), 4.40 (d, *J* = 5.7 Hz, 2H, NCH_2_), 3.07–3.00 (m, 4H, piperidine-NCH_2_ × 2), 1.62–1.54 (m, 4H, piperidine-CH_2_ × 2), 1.53–1.46 (m, 2H, piperidine-CH_2_). ^13^C NMR (100 MHz, DMSO-*d*_6_): δ = 168.07, 151.81, 147.70, 142.98, 139.56, 137.22, 135.38, 134.74, 132.49, 129.37, 128.85, 128.75, 127.81, 126.03, 120.52, 119.79, 115.57, 49.89, 42.92, 25.66, 24.10. HPLC: a gradient consisting of acetonitrile (ACN) and water containing 0.1% diethyl amine, with a linear increase of ACN from 10% to 100% over 10 min, followed by 100% ACN; flow: 0.6 mL/min.; *r*_t_ = 10.467 min. HRMS (ESI) calculated for C_28_H_28_N_4_O_3_S [M + H]^+^ 501.1955, found 501.1956.

#### *N*-(2-Hydroxybenzyl)-5-piperidin-1-yl-2-[(quinolin-8-ylsulfonyl)­amino]­benzamide (**18**)

Following the general procedure (method F), starting from derivative **76** dissolved in dry DMF and using 2-hydroxybenzylamine, after stirring for 48 h and acidifying with 2 M HCl (pH = 5), compound **18** was obtained after filtration and purification by crystallization by Cy/EtOAc as a yellow solid in 11% yield (0.04 g). ^1^H NMR (400 MHz, DMSO-*d*_6_): δ = 11.03 (s, 1H, NHSO_2_), 9.57 (s, 1H, OH), 8.82–8.76 (m, 1H, Ar–H), 8.75–8.67 (m, 1H, Ar–H), 8.47 (d, *J* = 8.8 Hz, 1H, Ar–H), 8.36 (d, *J* = 7.0 Hz, 1H, Ar–H), 8.26 (d, *J* = 7.0 Hz, 1H, Ar–H), 7.71 (t, *J* = 8.0 Hz, 1H, CONH), 7.63–7.54 (m, 1H, Ar–H), 7.46 (d, *J* = 8.9 Hz, 1H, Ar–H), 7.16–7.08 (m, 1H, Ar–H), 7.06–6.97 (m, 2H, Ar–H), 6.96–6.83 (m, 2H, Ar–H), 6.82–6.74 (m, 1H, Ar–H), 4.38–4.23 (m, 2H, NCH_2_), 3.09–2.89 (m, 4H, piperidine-NCH_2_ × 2), 1.60–1.48 (m, 4H, piperidine-CH_2_), 1.47–1.40 (m, 2H, piperidine-CH_2_ × 2). ^13^C NMR (100 MHz, DMSO-*d*_6_): δ = 168.30, 155.30, 151.80, 147.67, 142.98, 137.20, 135.39, 134.73, 132.50, 129.38, 128.75, 128.69, 128.35, 126.02, 125.21, 123.26, 120.35, 123.02, 119.73, 119.32, 115.73, 115.46, 49.90, 38.14, 26.81, 24.12. HPLC: Method B; *r*_t_ = 10.840 min. HRMS (ESI) calculated for C_28_H_28_N_4_O_4_S [M + H]^+^ 517.1904, found 517.1908.

#### *N*-(3-Hydroxybenzyl)-5-piperidin-1-yl-2-[(quinolin-8-ylsulfonyl)­amino]­benzamide (**19**)

Following the general procedure (method F), starting from derivative **76** dissolved in dry DMF and using 3-hydroxybenzylamine, after stirring for 1 h and acidifying with 2 M HCl (pH = 7), compound **19** was obtained after filtration and purification by flash chromatography column eluting with CHCl_3_/MeOH (99:1) as a yellow solid in 27% yield (0.10 g). ^1^H NMR (400 MHz, CDCl_3_): δ = 9.36 (s, 1H, NHSO_2_), 9.00 (dd, *J* = 1.7 and 4.2 Hz, 1H, Ar–H), 8.34 (dd, *J* = 1.3 and 7.3 Hz, 1H, Ar–H), 8.26 (dd, *J* = 1.6 and 8.4 Hz, 1H, Ar–H), 8.05 (dd, *J* = 1.2 and 8.2 Hz, 1H, Ar–H), 7.63–7.56 (m, 1H, Ar–H), 7.53 (dd, *J* = 4.2 and 8.3 Hz, 1H, Ar–H), 7.18 (t, *J* = 7.8 Hz, 1H, Ar–H), 7.10–7.07 (m, 1H, Ar–H), 7.03 (d, *J* = 2.8 Hz, 1H, Ar–H), 6.86 (bs, 1H, CONH), 6.83–6.78 (m, 2H, Ar–H), 6.76 (dd, *J* = 2.1 and 8.0 Hz, 1H, Ar–H), 6.64 (dd, *J* = 2.9 and 9.0 Hz, 1H, Ar–H), 6.06 (bs, 1H, OH), 4.52 (d, *J* = 5.8 Hz, 2H, NCH_2_), 3.03 (t, *J* = 5.1 Hz, 4H, piperidine-NCH_2_ × 2), 1.64–1.55 (m, 4H, piperidine-CH_2_ × 2), 1.54–1.47 (m, 2H, piperidine-CH_2_). ^13^C NMR (100 MHz, CDCl_3_): δ = 168.09, 156.71, 151.58, 149.67, 143.21, 139.35, 136.77, 135.87, 133.69, 131.38, 130.65, 129.77, 128.73, 125.67, 125.55, 124.65, 122.47, 119.35, 118.66, 115.79, 114.62 (2C), 50.14, 43.66, 25.55, 24.01. HPLC: Method B; *r*_t_ = 9.497 min. HRMS (ESI) calculated for C_28_H_28_N_4_O_4_S [M + H]^+^ 517.1904, found 517.1905.

#### *N*-(4-Hydroxybenzyl)-5-piperidin-1-yl-2-[(quinolin-8-ylsulfonyl)­amino]­benzamide (**20**)

Following the general procedure (method F), starting from derivative **76** dissolved in dry DMF and using 4-hydroxybenzylamine, after stirring for 2 h and acidifying with 2 M HCl (pH = 5), compound **20** was obtained after filtration and purification by flash chromatography column eluting with CHCl_3_/MeOH (95:5) as a green solid in 31% yield (0.15 g). ^1^H NMR (400 MHz, DMSO-*d*_6_): δ = 10.83 (s, 1H, NHSO_2_), 9.12 (s, 1H, OH), 8.56 (dd, *J* = 1.7 and 4.2 Hz, 1H, Ar–H), 8.53 (t, *J* = 5.8 Hz, 1H, CONH), 8.25 (dd, *J* = 1.6 and 8.4 Hz, 1H, Ar–H), 8.13 (dd, *J* = 1.4 and 7.3 Hz, 1H, Ar–H), 8.03 (dd, *J* = 1.2 and 8.3 Hz, 1H, Ar–H), 7.51–7.46 (m, 1H, Ar–H), 7.37 (dd, *J* = 4.2 and 8.3 Hz, 1H, Ar–H), 7.26–7.22 (m, 1H, Ar–H), 6.86–6.81 (m, 2H, Ar–H), 6.72–6.67 (m, 2H, Ar–H), 6.54–6.49 (m, 2H, Ar–H), 4.00 (d, *J* = 5.8 Hz, 2H, NCH_2_), 2.75 (t, *J* = 4.8 Hz, 4H, piperidine-NCH_2_ × 2), 1.33–1.26 (m, 4H, piperidine-CH_2_ × 2), 1.25–1.18 (m, 2H, piperidine-CH_2_). ^13^C NMR (100 MHz, DMSO-*d*_6_): δ = 167.88, 156.83, 151.84, 147.65, 142.99, 137.19, 135.40, 134.71, 132.49, 129.72, 129.40, 129.23 (2C), 128.73, 126.02, 123.32, 123.00, 120.41, 119.71, 115.62, 115.55 (2C), 49.91, 42.51, 25.66, 24.10. HPLC: Method A; *r*_t_ = 5.617 min. HRMS (ESI) calculated for C_28_H_28_N_4_O_4_S [M + H]^+^ 517.1904, found 517.1897.

#### 5-Piperidin-1-yl-*N*-(pyridin-2-ylmethyl)-2-[(quinolin-8-ylsulfonyl)­amino]­benzamide (**21**)

Following the general procedure (method F), starting from derivative **76** dissolved in dry DMF and using 2-picolylamine, after stirring for 24 h and acidifying with acetic acid (pH = 5), compound **21** was obtained after filtration and purification by flash chromatography column eluting with CHCl_3_/MeOH (99:1) as a yellow solid in 28% yield (0.10 g). ^1^H NMR (400 MHz, DMSO-*d*_6_): δ = 11.03 (s, 1H, NHSO_2_), 8.98 (t, *J* = 5.8 Hz, 1H, CONH), 8.89 (d, *J* = 3.5 Hz, 1H, Ar–H), 8.59 (d, *J* = 4.0 Hz, 1H, Ar–H), 8.52 (d, *J* = 7.7 Hz, 1H, Ar–H), 8.41 (d, *J* = 7.2 Hz, 1H, Ar–H), 8.31 (d, *J* = 8.0 Hz, 1H, Ar–H), 7.84 (t, *J* = 8.1 Hz, 1H, Ar–H), 7.76 (t, *J* = 7.8 Hz, 1H, Ar–H), 7.67 (dd, *J* = 4.1 and 8.1 Hz, 1H, Ar–H), 7.52 (d, *J* = 9.0 Hz, 1H, Ar–H), 7.39–7.35 (m, 1H, Ar–H), 7.30 (d, *J* = 7.8 Hz, 1H, Ar–H), 7.09–7.06 (m, 1H, Ar–H), 6.99 (dd, *J =* 2.4 and 9.1 Hz, 1H, Ar–H), 4.49 (d, *J* = 5.4 Hz, 2H, NCH_2_), 3.09–3.02 (m, 4H, piperidine-NCH_2_ × 2), 1.63–1.54 (m, 4H, piperidine-CH_2_ × 2), 1.53–1.47 (m, 2H, piperidine-CH_2_). ^13^C NMR (100 MHz, DMSO-*d*_6_): δ = 168.24, 158.67, 151.79, 149.41, 147.74, 142.97, 137.28 (2C), 137.23, 135.41, 134.74, 132.45, 129.27, 129.75, 126.03, 123.41, 123.03, 122.71, 121.56, 120.61, 119.76, 115.64, 49.88, 44.87, 25.66, 24.12. HPLC: Method B; *r*_t_ = 7.120 min. HRMS (ESI) calculated for C_27_H_27_N_5_O_3_S [M + H]^+^ 502.1908, found 502.1913.

#### 5-Piperidin-1-yl-*N*-(pyridin-3-ylmethyl)-2-[(quinolin-8-ylsulfonyl)­amino]­benzamide (**22**)

Following the general procedure (method F), starting from derivative **76** dissolved in dry DMF and using 3-picolylamine, after stirring for 24 h and acidifying with acetic acid (pH = 5), compound **22** was obtained after filtration and purification by flash chromatography column eluting with CHCl_3_/MeOH (97:3) as a white solid in 15% yield (0.05 g). ^1^H NMR (400 MHz, DMSO-*d*_6_): δ = 10.99 (s, 1H, NHSO_2_), 8.94 (t, *J* = 5.6 Hz, 1H, CONH), 8.87 (dd, *J* = 1.6 and 4.2 Hz, 1H, Ar–H), 8.59–8.55 (m, 2H, Ar–H), 8.53 (dd, *J* = 1.6 and 8.4 Hz, 1H, Ar–H), 8.40 (dd, *J* = 1.2 and 7.2 Hz, 1H, Ar–H), 8.31 (dd, *J* = 1.2 and 8.2 Hz, 1H, Ar–H), 7.76 (t, *J* = 7.8 Hz, 1H, Ar–H), 7.72–7.64 (m, 2H, Ar–H), 7.54–7.49 (m, 1H, Ar–H), 7.45 (dd, *J* = 4.8 and 7.7 Hz, 1H, Ar–H), 7.02–6.96 (m, 2H, Ar–H), 4.42 (d, *J* = 5.7 Hz, 2H, NCH_2_), 3.03 (t, *J* = 4.8 Hz, 4H, piperidine-NCH_2_ × 2), 1.61–1.54 (m, 4H, piperidine-CH_2_ × 2), 1.53–1.46 (m, 2H, piperidine-CH_2_). ^13^C NMR (100 MHz, DMSO-*d*_6_): δ = 16 8.23, 151.77, 149.35, 148.72, 147.73, 142.97, 137.28, 135.66, 135.33, 135.01, 134.76, 132.52, 129.30, 128.75, 126.05, 124.03, 123.13, 123.02, 120.58, 119.88, 115.58, 49.87, 40.71, 25.65, 24.09. HPLC: Method B; *r*_t_ = 6.027 min. HRMS (ESI) calculated for C_27_H_27_N_5_O_3_S [M + H]^+^ 502.1908, found 502.1911.

#### 5-Piperidin-1-yl-*N*-(pyridin-4-ylmethyl)-2-[(quinolin-8-ylsulfonyl)­amino]­benzamide (**23**)

Following the general procedure (method F), starting from derivative **76** dissolved in dry DMF and using 4-picolylamine, after stirring for 24 h and acidifying with acetic acid (pH = 6), compound **23** was obtained after filtration and purification by flash chromatography column eluting with CHCl_3_/MeOH (98:2) as a yellow solid in 43% yield. ^1^H NMR (400 MHz, CDCl_3_): δ = 9.19 (s, 1H, NHSO_2_), 9.09 (dd, *J* = 1.7 and 4.3 Hz, 1H, Ar–H), 8.58–8.52 (m, 2H, Ar–H), 8.34–8.27 (m, 2H, Ar–H), 8.07 (dd, *J* = 1.3 and 8.2 Hz, 1H, Ar–H), 7.63–7.56 (m, 2H, Ar–H), 7.50 (t, *J* = 6.5 Hz, 1H, CONH), 7.31–7.27 (m, 2H, Ar–H), 7.20 (bs, 1H, Ar–H), 6.63–6.61 (m, 2H, Ar–H), 4.61 (d, *J =* 6.0 Hz, 2H, NCH_2_), 3.07 (t, *J* = 5.1 Hz, 4H, piperidine-NCH_2_ × 2), 1.64–1.57 (m, 4H, piperidine-CH_2_ × 2), 1.56–1.48 (m, 2H, piperidine-CH_2_). ^13^C NMR (100 MHz, CDCl_3_): δ = 167.77, 151.50, 150.13, 149.93 (2C), 147.36, 143.24, 136.99, 135.76, 133.66, 131.57, 130.89, 128.80, 125.72, 125.32, 125.20, 122.45 (2C), 122.42, 118.66, 116.45, 49.98, 42.86, 25.55, 24.03. HPLC: Method B; *r*_t_ = 5.683 min. HRMS (ESI) calculated for C_27_H_27_N_5_O_3_S [M + H]^+^ 502.1908, found 502.1908.

#### *N*-[4-(Aminosulfonyl)­benzyl]-5-piperidin-1-yl-2-[(quinolin-8-ylsulfonyl)­amino]­benzamide (**24**)

Following the general procedure (method F), starting from derivative **76** dissolved in dry DMF and using 4-(aminomethyl)­benzenesulfonamide, after stirring for 1 h and acidifying with 2 M HCl (pH = 5), compound **24** was obtained after filtration and purification by flash chromatography column eluting with CHCl_3_/MeOH (97:3) as a yellow solid in 62% yield (0.22 g). ^1^H NMR (400 MHz, DMSO-*d*_6_): δ = 10.98 (s, 1H, NHSO_2_), 8.94 (t, *J =* 5.7 Hz, 1H, CONH), 8.76 (dd, *J* = 1.5 and 4.1 Hz, 1H, Ar–H), 8.47 (dd, *J* = 1.5 and 8.3 Hz, 1H, Ar–H), 8.38–8.33 (m, 1H, Ar–H), 8.28–8.24 (m, 1H, Ar–H), 7.84–7.79 (m, 2H, Ar–H), 7.71 (t, *J* = 7.9 Hz, 1H, Ar–H), 7.59 (dd, *J* = 4.2 and 8.4 Hz, 1H, Ar–H), 7.49–7.41 (m, 3H, Ar–H), 7.38 (s, 2H, SO_2_NH_2_), 6.99 (d, *J* = 2.6 Hz, 1H, Ar–H), 6.94 (dd, *J* = 2.6 and 9.1 Hz, 1H, Ar–H), 4.41 (d, *J* = 5.7 Hz, 2H, NCH_2_), 3.00 (t, *J =* 4.6 Hz, 4H, piperidine-NCH_2_ × 2), 1.58–1.49 (m, 4H, piperidine-CH_2_ × 2), 1.48–1.41 (m, 2H, piperidine-CH_2_). ^13^C NMR (100 MHz, DMSO-*d*_6_): δ = 168.19, 151.74, 147.74, 143.63, 143.23, 142.96, 137.23, 135.34, 134.76, 132.49, 129.35, 128.74, 128.12, 126.24, 126.04, 123.09, 123.03, 120.58, 119.89, 115.59, 49.88, 42.60, 25.66, 24.10. HPLC: Method A; *r*_t_ = 5.510 min. HRMS (ESI) calculated for C_28_H_29_N_5_O_5_S_2_ [M + H]^+^ 580.1683, found 580.1688.

#### *N*-{1-[(Dimethylamino)­methyl]­propyl}-5-piperidin-1-yl-2-[(quinolin-8-ylsulfonyl)­amino]­benzamide (**25**)

Following the general procedure (method F), starting from derivative **76** dissolved in dry DMF and using 1.30 equiv of TBTU, 4.60 equiv of DIPEA and 1.30 equiv of (2-aminobutyl)­dimethylamine, after stirring for 1 h and basifying with a saturated solution of Na_2_CO_3_ (pH = 9), compound **25** was obtained after extraction and purification by flash chromatography column eluting with CHCl_3_/MeOH (95:5) as a yellow solid in 24% yield (0.09 g). ^1^H NMR (400 MHz, CDCl_3_): δ = 9.08 (dd, *J* = 1.7 and 4.2 Hz, 1H, Ar–H), 8.45 (dd, *J* = 1.3 and 7.3 Hz, 1H, Ar–H), 8.23 (dd, *J* = 1.7 and 8.4 Hz, 1H, Ar–H), 8.02 (dd, *J* = 1.3 and 8.2 Hz, 1H, Ar–H), 7.60 (t, *J =* 7.5 Hz, 1H, Ar–H), 7.53 (dd, *J* = 4.3 and 8.3 Hz, 1H, Ar–H), 7.06 (d, *J* = 2.8 Hz, 1H, Ar–H), 7.03 (d, *J* = 9.0 Hz, 1H, Ar–H), 6.98 (bs, 1H, NHSO_2_), 6.69 (dd, *J* = 2.9 and 9.0 Hz, 1H, Ar–H), 4.63 (bs, 1H, CONH), 4.14–4.03 (m, 1H, NCH), 3.02 (t, *J =* 5.3 Hz, 4H, piperidine-NCH_2_ × 2), 2.63–2.54 (m, 1H, CH_2_ × 1/2), 2.38–2.34 (m, 1H, CH_2_ × 1/2), 2.32 (s, 6H, N­(CH_3_)_2_), 1.75–1.65 (m, 1H, CH_2_ × 1/2), 1.64–1.57 (m, 5H, CH_2_ × 1/2 and piperidine-CH_2_ × 2), 1.54–1.47 (m, 2H, piperidine-CH_2_), 0.97 (t, *J =* 7.4 Hz, 3H, CH_3_). ^13^C NMR (100 MHz, CDCl_3_): δ = 167.88, 151.37, 148.94, 143.49, 136.69, 136.39, 133.49, 132.14, 128.82, 128.55, 127.32, 125.81, 122.99, 122.22, 119.16, 116.39, 62.28, 50.44, 49.37, 45.68, 26.16, 25.68, 24.06, 10.17. HPLC: Method B; *r*_t_ = 6.413 min, HRMS (ESI) calculated for C_27_H_35_N_5_O_3_S [M + H]^+^ 510.2534, found 510.2530.

#### *N*-[(1-Methylpiperidin-4-yl)­methyl]-5-piperidin-1-yl-2-[(quinolin-8-ylsulfonyl)­amino]­benzamide (**26**)

Following the general procedure (method F), starting from derivative **76** dissolved in dry DMF and using (1-methylpiperidin-4-yl)­methanamine, after stirring for 8 h and basifying with a saturated solution of Na_2_CO_3_ (pH = 9), compound **26** was obtained after extraction and purification by flash chromatography column eluting with CHCl_3_/MeOH (90:10) as a yellow solid in 20% yield (0.03 g). ^1^H NMR (400 MHz, DMSO-*d*_6_): δ = 11.12–10.40 (m, 1H, NHSO_2_), 9.01–8.98 (m, 1H, Ar–H), 8.49 (d, *J* = 8.8 Hz, 1H, Ar–H), 8.47–8.37 (m, 1H, CONH), 8.33 (d, *J* = 6.9 Hz, 1H, Ar–H), 8.25 (d, *J* = 8.4 Hz, 1H, Ar–H), 7.722–7.66 (m, 2H, Ar–H), 7.43 (d, *J* = 8.7 Hz, 1H, Ar–H), 6.96–6.87 (m, 2H, Ar–H), 3.03–2.93 (m, 6H, piperidine-NCH_2_ × 3), 2.78 (d, *J* = 9.2 Hz, 2H, NCH_2_), 2.19 (s, 3H, CH_3_), 1.88 (t, *J* = 9.7 Hz, 2H, piperidine-NCH_2_), 1.61–1.49 (m, 6H, piperidine-CH_2_ × 3), 1.49–1.41 (m, 2H, piperidine-CH_2_), 1.40–1.32 (m, 1H, piperidine-CH), 1.20–1.08 (m, 2H, piperidine-CH_2_). ^13^C NMR (100 MHz, DMSO-*d*_6_): δ = 168.12, 151.75, 147.53, 143.07, 137.27, 135.81, 134.55, 132.29, 129.63, 128.77, 126.01, 123.99, 122.96, 120.78, 119.59, 115.80, 55.37, 50.00, 46.38, 45.01, 35.30, 30.05, 25.69, 24.12. HPLC: Method B; *r*_t_ = 5.823 min. HRMS (ESI) calculated for C_28_H_35_N_5_O_3_S [M + H]^+^ 522.2534, found 522.2538.

#### Ethyl 2-[Methyl­(quinolin-8-ylsulfonyl)­amino]-5-piperidin-1-ylbenzoate (**77**)

Under N_2_ atmosphere, to a solution of derivative **75** (0.10 g, 0.23 mmol) in dry DMF (5 mL), iodomethane (0.02 mL, 0.39 mmol) and K_2_CO_3_ (0.06 g, 0.46 mmol) were added. After stirring at rt for 8 h, the reaction mixture was poured into ice/water and basified with a saturated solution of Na_2_CO_3_ (pH = 9) to give a white precipitate that was filtered under vacuum to obtain compound **77** as a white solid in 52% yield. ^1^H NMR (400 MHz, CDCl_3_): δ = 9.12 (dd, *J* = 1.8 and 4.2 Hz, 1H, Ar–H), 8.26–8.20 (m, 2H, Ar–H), 7.97 (dd, *J* = 1.2 and 8.2 Hz, 1H, Ar–H), 7.54 (dd, *J* = 4.2 and 8.3 Hz, 1H, Ar–H), 7.49–7.44 (m, 1H, Ar–H), 7.28–7.26 (m, 1H, Ar–H), 6.61 (dd, *J* = 3.0 and 8.8 Hz, 1H, Ar–H), 6.40 (d, *J* = 8.8 Hz, 1H, Ar–H), 4.19 (q, *J* = 7.2 Hz, 2H, O*CH*_2_CH_3_), 3.71 (s, 3H, N­(CH_3_)­SO_2_), 3.12 (t, *J* = 5.0 Hz, 4H, piperidine-NCH_2_ × 2), 1.60–1.49 (m, 6H, piperidine-CH_2_ × 3), 1.36 (t, *J* = 7.1 Hz, 3H, OCH_2_ *CH*_3_).

#### 2-[Methyl­(quinolin-8-ylsulfonyl)­amino]-5-piperidin-1-ylbenzoic Acid (**78**)

Following the same procedure as compound **76**, starting from derivative **77** (0.28 g, 0.62 mmol) after stirring at 70 °C for 96 h, compound **78** was obtained after extraction as a light brown solid in 53% yield (0.14 g). ^1^H NMR (400 MHz, DMSO-*d*_6_): δ = 12.82 (bs, 1H, CO_2_H), 9.19 (dd, *J* = 1.8 and 4.2 Hz, 1H, Ar–H), 8.63 (dd, *J* = 1.7 and 8.4 Hz, 1H, Ar–H), 8.32 (dd, *J* = 1.3 and 8.2 Hz, 1H, Ar–H), 8.12 (dd, *J* = 1.4 and 7.4 Hz, 1H, Ar–H), 7.80 (dd, *J* = 4.2 and 8.4 Hz, 1H, Ar–H), 7.71–7.65 (m, 1H, Ar–H), 7.15 (d, *J* = 3.0 Hz, 1H, Ar–H), 6.81 (dd, *J* = 3.0 and 8.9 Hz, 1H, Ar–H), 6.35 (d, *J* = 8.8 Hz, 1H, Ar–H), 3.65 (s, 3H, N­(CH_3_)­SO_2_), 3.20–3.13 (m, 4H, piperidine-NCH_2_ × 2), 1.63–1.51 (m, 6H, piperidine-CH_2_ × 3).

#### *N*-(Cyclohexylmethyl)-2-[methyl­(quinolin-8-ylsulfonyl)­amino]-5-piperidin-1-ylbenzamide (**27**)

Following the general procedure (method F), starting from derivative **78** dissolved in dry DMF and using cyclohexanemethylamine, after stirring for 1 h and acidifying with acetic acid (pH = 5), compound **27** was obtained after filtration and purification by flash column chromatography eluting with CHCl_3_/Et_2_O (93:7) as a white solid in 56% yield (0.09 g). ^1^H NMR (400 MHz, CDCl_3_): δ = 9.14 (dd, *J* = 1.7 and 4.2 Hz, 1H, Ar–H), 8.33–8.27 (m, 2H, Ar–H), 8.07 (dd, *J* = 1.2 and 8.2 Hz, 1H, Ar–H), 7.91 (t, *J* = 5.4 Hz, 1H, CONH), 7.61–7.54 (m, 2H, Ar–H), 7.21 (d, *J* = 3.0 Hz, 1H, Ar–H), 6.41 (dd, *J* = 3.1 and 8.9 Hz, 1H, Ar–H), 5.95 (d, *J* = 8.8 Hz, 1H, Ar–H), 3.61 (s, 3H, N­(CH_3_)­SO_2_), 3.55 (t, *J =* 6.2 Hz, 2H, NCH_2_), 3.12 (t, *J* = 4.8 Hz, 4H, piperidine-NCH_2_ × 2), 1.95–1.87 (m, 2H, piperidine-CH_2_), 1.81–1.72 (m, 2H, piperidine-CH_2_), 1.71–1.59 (m, 6H, piperidine-CH_2_ and cyclohexyl-CH_2_ × 2), 1.55–1.49 (m, 2H, cyclohexyl-CH and CH_2_ × 1/2), 1.37–1.17 (m, 3H, cyclohexyl-CH_2_ and CH_2_ × 1/2), 1.14–1.03 (m, 2H, cyclohexyl-CH_2_). ^13^C NMR (100 MHz, CDCl_3_): δ = 167.60, 151.46, 144.21, 138.22, 137.87, 136.56, 133.55, 133.44 (2C), 129.05, 128.52, 127.38, 125.63, 122.21, 117.02, 117.00, 49.38, 46.55, 42.57, 38.06, 31.02, 26.47, 25.96, 25.51, 24.14. HPLC: Method A; *r*_t_ = 9.273 min. HRMS (ESI) calculated for C_29_H_36_N_4_O_3_S [M + H]^+^ 521.2581, found 521.2583.

### Cell Lines and Viruses

Huh-7 cells (human hepatocarcinoma; JCRB), Vero cells (African green monkey kidney fibroblast; ATCC) RD cells (Human Rhabdomyosarcoma; ATCC) and SH-SY5Y (human neuroblastoma; ATCC) were cultured in DMEM medium (Gibco) with 4.5 g/L glucose, 10% (v/v) fetal bovine serum (FBS) and 1% (v/v) penicillin/streptomycin (P/S) at 37 °C in 5% CO_2_. Caco2 cells (human colon adenocarcinoma; ATCC) were cultured in DMEM medium, supplemented with GlutaMAX, 10% FBS and 1% P/S at 37 °C in 5% CO_2_. BHK-21 cells (baby hamster kidney fibroblast; ATCC) in RPMI 1640 medium (Gibco) supplemented with 10% FBS and 1% P/S at 37 °C in 5% CO_2_.

DENV-1 (EDEN-1, Genbank accession EU081230), DENV-2 (EDEN-2, GenBank accession: EU081177), DENV-3 (EDEN-3, GenBank accession EU081190), and DENV-4 (EDEN-4, GenBank accession GQ398256) was obtained from the Early Dengue infection and outcome (EDEN) study.^74^ JEV SA14–14–2 (GenBank accession AF315119), Yellow Fever vaccine strain YF17D (GenBank accession X03700), CHIKV laboratory strain ‘ROSS’, and the human clinical isolate CHIKV EAS (East African lineage strain – DMERI09/08) were generously provided by Prof. Ooi Eng Eong (Duke-NUS). WNV_Kunjin_ (GenBank accession KX394398.1) was obtained from the National Environmental Agency, Singapore. ZIKV (H/PF/2013 GenBank Accession KJ776791) was obtained from the European Virus Archive. Human coronavirus 229E was a local isolate strain by Prof. Gavin Smith’s group from Duke-NUS. Enterovirus EV71 S41 (GenBank accession AF316321) was obtained from Prof. Sylvie Alonso’s group from NUS.

### Ethics Statement

Institutional approval has been granted by Duke-NUS Medical School to perform experiments with flaviviruses, alphaviruses and coronaviruses.

### Antiviral Inhibition Assay with Flaviviruses

For single point inhibition assay, 1 × 10^5^ cells/well of Huh-7 cells were seeded in 24-well plates and incubated at 37 °C overnight. The following day, Huh-7 cells were infected with DENV at a MOI (Multiplicity of Infection) value of 0.3 in a serum-free medium for 1 h at 37 °C, followed by treatment with compounds at a concentration of 10 μM for 48 h. The lyophilized compound was reconstituted in 100% DMSO to create a 20 mM stock solution. The highest drug concentration (100 μM) was prepared by diluting the 20 mM stock in the culture media, with the final DMSO percentage being less than 0.1%. Viral supernatants following treatment were collected, clarified, and subjected to plaque quantification. For dose–response inhibition assay against DENV, 1 × 10^5^ cells/well of Huh-7 cells were seeded in 24-well plates and incubated at 37 °C overnight. The next day, Huh-7 cells were infected with DENV at MOI 0.3 in serum-free media for 1 h at 37 °C, followed by treatment with compounds at serial concentrations of 0.01 μM, 0.1 μM, 1 μM, 10 μM, 50 μM, and 100 μM for 48 h. Supernatants were collected, clarified, and subjected to plaque quantification in Baby Hamster Kidney-21­(BHK-21) cells and quantitative real-time PCR.

For other Flaviviruses, Huh-7 cells were infected with Zika virus, at an MOI value of 0.5 and treated with the compounds for 24 h, whereas Vero cells were used for JEV SA 14–2–2 (MOI 0.3), WNV_Kunjin_ (MOI 0.03), and YF17D (MOI 0.3), with all treatments performed for 48 h. Following infection and compound treatment, viral supernatants were collected, clarified, and subjected to plaque quantification or quantitative real-time PCR. The efficacy of the compounds (EC_50_: concentration at which the virus infection is reduced by 50%) was determined by the sigmoidal dose–response curve of virus titer against concentration in GraphPad Prism.

### Antiviral Inhibition Assay with Alphavirus

For dose–response inhibition assay against CHIK-ROSS and CHIKV-EAS, 1 × 10^5^ cells/well of Vero cells were seeded in 24-well plates and incubated at 37 °C overnight. The next day, Vero cells were infected with CHIKV at MOI 1 in serum-free media for 1 h at 37 °C, followed by treatment with compounds at serial concentrations of 0.01 μM, 0.1 μM, 1 μM, 10 μM, 50 μM, and 100 μM for 24 h. Supernatants were collected, clarified, and subjected to plaque quantification in BHK-21 cells.

### Antiviral Inhibition Assay with Enterovirus

For dose–response against enterovirus EV71 S41 (GenBank accession no. AF316321), SHSY-5Y cells (ATCC CRL-2266) were seeded overnight in 96-well plates at 2 × 10^4^ cells/well. The next day, SHSY-5Y cells were infected with S41 a MOI 0.1 in serum-free media for 1 h at 37 °C, followed by treatment with compound at serial concentrations of 0.0488 μM, 0.195 μM, 0.781 μM, 3.125 μM, 12.5 μM, and 50 μM for 48 h. Supernatant were collected, clarified, and subjected to plaque quantification in Rhabdomyosarcoma (RD) cells.

### Antiviral Inhibition Assay with Coronavirus

For dose–response against Coronavirus, Caco2 cells were seeded overnight in 24-well plates at 1 × 10^5^ cells/well. The next day, Caco2 cells were infected with coronavirus at MOI 0.01 in serum-free media for 1 h at 37 °C, followed by treatment with the compound at serial concentrations of 0.01 μM, 0.1 μM, 1 μM, 10 μM, 50 μM, and 100 μM for 72 h. The supernatant was collected, clarified, and subjected to quantitative real-time PCR.

### Cell Viability Assay

Two × 10^4^ Huh-7 and Vero cells were plated onto a 96-well white opaque plate (Grenier) and treated with the compounds at the indicated concentrations for 48 h. Cytotoxicity was determined by CellTiter Glo Luminescent Assay (Promega) kit according to the manufacturer’s instructions. For the cell viability test in SH-SY5Y, Alamar blue was added to the cells after 48 h. Cell viability curve is presented as a percentage of luminescence derived from the treated sample to that of the untreated cell control.

### Total Proteome Analysis of Compound 1-Treated Cells

Huh-7 cells were seeded at a density of 4 × 10^5^/2 mL in a 6-well plate and incubated at 37 °C overnight. The next day, cells were infected with DENV-2 at an MOI of 5 for 1 h, followed by treatment with compound **1** at 10 μM for 24 h. Cells were washed twice with PBS and trypsinized. The cells were lysed by adding 100 μL of lysis buffer (8 M Urea, 20 mM Tris-pH 8.0). Cell debris was separated from the lysate by centrifugation at 10,000g for 30 min. The supernatant was collected. Samples were reduced and alkylated using 500 μM Tris­(2-carboxyethyl) phosphine and 550 μM chloroacetamide. Samples were then diluted with 100 mM triethylammonium bicarbonate buffer to reduce urea concentration and digested with lysozyme-c and trypsin. The digested peptides were desalted using C18 spin columns. The eluted peptides were dried using a vacuum concentrator and resuspended with 20 μL of 0.1% formic acid. 2 μL of samples were injected into LC-MS (Orbitrap HF-X). The peptides were separated on a 50 cm Easy-Spray RP-C18 column (Thermo Scientific) in a 60 min gradient of solvent A (0.1% Formic acid in water and solvent B (99.9% acetonitrile) on an EASY-nLC 1000 coupled Orbitrap HF-X mass spectrometer (Thermo Scientific). MS spectra were acquired under data-dependent acquisition mode (DDA). The spectra were annotated against target-decoy Human and Dengue virus 2 database with carbamidomethyl as fixed modifications and oxidation, deamidation, and N-terminal acetylation as variable modifications. FDR was set to 1%, and label-free quantification was performed on Proteome Discoverer software (Thermo Scientific).

### Puromycin Labeling

Huh-7 cells were seeded at a density of 1 × 10^5^ cells/well in a 24-well plate and incubated overnight at 37 °C. The following day, cells were infected with DENV at MOI 1 for 1 h followed by compounds treatment at 10 μM for 1 h, 3 and 24 h, respectively. Following treatment, the cells were then pulsed with puromycin (10ug/mL) (Sigma-Aldrich) for 40 min and washed once with ice-cold PBS. Cells were lysed with commercial lysis buffer (Cell Signaling #9803) and total protein was quantified by Bradford. A 10 μg of proteins from each sample were resolved on 12% SDS-PAGE followed by Western blotting using an antipuromycin antibody (Sigma-Aldrich, USA).

### Transfection of Eukaryotic Cells with Plasmid DNA

The Flag-NS5 and Flag-CPSF3 expression plasmids were obtained from the preprint by Tan et al.^75^ Site-directed mutagenesis of the 5′-TOP motif was performed using the QuikChangeII Site-Directed Mutagenesis Kits (Agilent) according to the manufacturer’s instructions, introducing a cytidine to guanine substitution at the first nucleotide position (C > G). The forward primer sequence used was 5′-GTC GCC GTG AAC GTT GTT TTT CGC AAC GGG TTT G-3′, and the reverse primer sequence was 5′-CAA ACC CGT TGC GAA AAA CAA CGT TCA CGG CGA C-3′.

Huh-7 cells were seeded at a density of 1 × 10^5^/mL in a 24-well plate. The following day, the plasmid was transfected into Huh-7 cells using Fugene 6 transfection reagent as per the manufacturer’s instructions (Promega). Briefly, a 2.5 μL of Fugene transfection reagent was added to the 50 μL of serum-free medium and the reaction mixture was incubated at room temperature for 5 min. One μg of plasmid was then added and incubated for an additional 15 min. Following incubation, the transfection mixture was added to the wells, and the plate was gently swirled before incubating for 24 h. For the compound treatment in the transfection assay, 10 μM of the compound was added to the cells 6 h post-transfection, followed by 24 h incubation. Subsequently, the cells were lysed using a commercial lysis buffer (Cell signaling) and the lysates were subjected to Western blot analysis.

### siRNA-Mediated Knockdown

Huh-7 cells were seeded at a density of 1 × 10^5^ cells/well in a 24-well plate and incubated overnight at 37 °C. SMARTPool siRNAs were purchased from Dharmacon and prepared at a stock concentration of 10 μM. Transfections were conducted following the JetPRIME’s manufacturer protocol. Briefly, 30 nM of siRNA was mixed with JetPRIME buffer, vortexed gently. Next, 2 μL of JetPRIME transfection reagent was added and the mixture was incubated at room temperature for 15 min to allow complex formation. The transfection mixture was then added to the cells, swirled gently, and incubated at 37 °C for 48 h. Following transfection, cells were infected with DENV at a MOI of 1 and incubated for 24 h. Subsequently, intracellular RNA was extracted using the RNeasy kit and subjected to RNA quantification with qRT-PCR.

### Antiviral Evaluation in *Ex Vivo* Human Brain Organoid Model

For antiviral testing, day 60 organoids were infected with ZIKV at MOI 1 for 2 h. Following infection, organoids were washed twice with culture media. SAA compounds and control antiviral compounds NITD008 were diluted in culture media to a final concentration of 5 μM and added to the organoids. The organoids were incubated at 37 °C with gentle agitation, and viral supernatants were collected daily for 4 days for viral copy number quantification by qRT-PCR.

### Determination of Kinetic Solubility

A calibration curve was constructed for the test compounds by using the HPLC Jasco LC-4000 equipped with the AS-4550 autosampler (100 μL loop) and a MD-4015 photodiode array (200–600 nm). In summary, a 10 mM stock solution of the compounds in DMSO was diluted 1:100 in MeOH to reach a concentration of 100 μM. Subsequently, three injections (50, 20, and 2 μL) were made in duplicate, corresponding to 50, 20, and 2 μM, respectively. Chromatograms were recorded at 224 nm, which corresponds to the λ_max_ of both SAA analogues. The calibration curve was constructed by plotting the area of the peak against the concentration using the ChromNAV 2.0 software.

A total of 10 μL of the test compounds was added to 990 μL of buffer (PBS_7.4_) from the 10 mM stock solution in DMSO. The mixture was then subjected to a 24-h mixing process at 1000 rpm at a temperature of 25 °C. Subsequently, the samples were subjected to centrifugation at 10,000 rpm at 25 °C for 10 min. Thereafter, 300 μL of the resulting supernatant was collected and diluted with 300 μL of MeOH. A volume of 50 μL from each sample was injected into the HPLC, and the chromatograms were recorded at 224 nm. The measured values were corrected for the corresponding dilution factor (x4). The compounds were tested in duplicate.

### *In Vitro* Membrane Permeability

The donor solutions were prepared at a concentration of 250 μM by diluting DMSO stock solutions with 1X PBS (pH 7.4), ensuring a final DMSO concentration of 50%. For the artificial lipid membrane solution used in the PAMPA assay, phosphatidylcholine was dissolved in dodecane to a final concentration of 1% (w/v). 96-well microfilter plates (MultiScreen-IP, 0.45 μm, catalogue no. MAIPN4550) were used as donor compartments, while the acceptor compartments were set up using the 96-well microtiter plates (MultiScreen-acceptor, catalogue no. MSSACCEPTOR). The filter surface of the donor plate was coated with 5 μL of freshly prepared lipid solution. After applying the lipid membrane, 300 μL of 1X PBS (pH 7.4) containing 50% DMSO was added to each well of the acceptor plate, and 150 μL of the donor solutions were placed in the donor compartment. The donor and acceptor plates were assembled into a sandwich and incubated at room temperature for 2 h, with stirring at 600 rpm using a Heidolph TITRAMAX 100. After incubation, the donor and acceptor plates were separated. The concentrations of compounds in the acceptor compartment were measured by UV spectrophotometry. All experiments were conducted in triplicate, and the apparent permeability values (*P*_app_) were reported as the mean ± standard deviation. The apparent permeability, *P*_app_, was calculated using the modified Faller-Sugano equation:^73^ (1)­

1 $$P_{\mathrm{app}}=-\left[\left(V_{D}V_{R}\right)/{(V}_{D}+V_{R})\mathrm{At}\right]\ln\left(1-r\right)$$

*V*_R_ is the volume of the acceptor compartment (0.300 cm^3^), *V*_D_ is the volume of the donor compartment (0.150 cm^3^), *A* is the accessible filter area (0.266 cm^2^), *t* is the incubation time (in seconds), *r* is the ratio of the concentrations in the acceptor and equilibrium solution.

### Plasma Stability Assay

The plasma stability of compounds **1** and **7** was evaluated by incubating them in human plasma at 37 °C. Biotransformation was initiated upon the addition and mixing of the compounds. At specific time points (0, 60, and 120 min), the reaction was stopped using ice-cold methanol, with an internal standard added during the quenching process. LC-MS analysis was then performed to measure the remaining compound, and stability was calculated as the percentage of the initial concentration at 0 min. Procaine and procainamide were used as reference controls, representing compounds with low and high stability, respectively. All experiments were conducted in duplicate.

### Metabolic Stability Assay in Liver Microsomes

Liver microsomal stability was assessed using human liver microsomes (HLMs) with β-nicotinamide adenine dinucleotide phosphate (NADPH) as cofactor. Samples (10 μM) were incubated at 37 °C for 20, 40, and 60 min, quenched with ice-cold methanol containing an internal standard (IS), centrifuged, and analyzed by LC-MS/MS. A 0 min control was prepared by immediate solvent addition; testosterone and cofactor-free incubations served as positive and negative controls, respectively. All experiments were performed in triplicate. Results were expressed as percentage turnover of the parent compound, from which *in vitro* half-life (*t*_1/2_), intrinsic clearance (CL_int,micr_), and scaled in vivo intrinsic clearance (CL_int_) were calculated.

### LC-MS/MS Conditions and Calibration Curve

Analyses were performed on a Waters ACQUITY UPLC I-Class system coupled online to a Waters Xevo TQ-XS triple-quadrupole mass spectrometer (Waters Technologies Corp., Milford, MA). Chromatographic separation was achieved on a Kinetex Evo C8 column (2.6 μm, 100 Å, 50 × 3 mm; Phenomenex, Bologna, Italy) thermostated at 40 °C, using H_2_O (A) and ACN (B), both containing 0.1% HCOOH, with a linear gradient from 5% to 95% B over 3 min at a flow rate of 0.7 mL min^–1^.

Electrospray ionization was operated in the positive mode. Multiple reaction monitoring (MRM) transitions were *m*/*z* 476.19 > 106.05 (**1**) and *m*/*z* 507.27 > 315.21 (**7**). Key MS settings included: source temperature of 150 °C, desolvation temperature of 650 °C, capillary voltage of 5 kV, cone voltage of 30 V, cone gas flow of 150 L/h, desolvation gas flow of 1100 L/h, argon as collision gas, and collision energy of 26 eV. Data were acquired and processed using MassLynx v4.2 and TargetLynx XS v4.2.

Calibration curves were prepared from DMSO stock solutions serially diluted in methanol to obtain the desired concentrations. Tolbutamide (50 nM) served as the IS. Curves (0.005–1 μM, six levels, triplicate) showed excellent linearity (*R* ^2^ ≥ 0.999) with regression equations *y* = 0.3676x – 0.0155 (**1**) and *y* = 0.85586x – 0.01192 (**7**).

### Metabolite Identification (MetID) Following Microsomal Incubation

MetID analyses were performed on a Vanquish UHPLC system coupled to an Orbitrap Exploris 120 mass spectrometer (Thermo Fisher Scientific, Bremen, Germany). Chromatographic separation was performed on a Kinetex EVO C18 column (2.6 μm, 100 × 2.1 mm; Phenomenex) using a gradient of H_2_O (A) and ACN (B), both acidified with 0.1% formic acid (5–95% B over 10 min), at a flow rate of 0.4 mL min^–1^ and a column temperature of 40 °C.

The mass spectrometer was operated in positive ESI mode. Full MS scans were acquired at 60,000 resolution (*m*/*z* 100–1500), ddMS/MS at 15,000 resolution using a 2 *m*/*z* isolation window and 30% normalized HCD energy. Source parameters: sheath/aux/sweep gas 60/15/2, ion transfer and vaporizer temperatures 300 °C, spray voltage +3.4/–3.0 kV, RF lens 70%, AGC target 200%, and maximum injection time 200 ms.

Data were processed using Compound Discoverer 3.3 (Thermo Fisher Scientific) with a customized MetID workflow adapted from Lamberti et al.^76^ In silico predictions of phase I metabolites were generated using GLORYx^77^ via the NERDD web portal (https://nerdd.univie.ac.at/gloryx), and the resulting mass list was imported into Compound Discoverer. The workflow comprised RT alignment, isotope pattern–based metabolite detection, compound grouping, FISh (Fragment Ion Search) scoring, spectral annotation, and elemental composition prediction with gap filling and background subtraction.

## Supplementary Material





## Notes

“Heat map analysis of protein expressions following DENV infection”; “Validation of mass spectrometry hits by western blotting”; “Development of dorsal forebrain organoids derived from H9 embryonic stem cells”; “Dose–response inhibition of compound **3** against the four DENV serotypes”; “Impact of SAA compounds on wild-type and mutant 5**′-**TOP Flag-CPSF3 expression level”; “Investigate the impact of compound **1** on mTOR signaling”; “Assessment of metabolic stability of compounds **1** and **7** after incubation with HLMs and LC-HRMS/MS-based characterization of their metabolites”; “MS^2^ spectra illustrating the proposed fragmentation pattern of metabolites derived from compound **1**”; “MS^2^ spectra of metabolites derived from compound **7** after incubation with HLMs in the presence of NADPH”. ^1^H NMR and ^13^C NMR spectra and HPLC chromatograms of all target compounds (PDF)
Supplementary methods Molecular formula strings of target compounds with associated biological data (CSV)

## Abbreviations Used

5′-TOP: 5′-terminal oligopyrimidine
ADE: antibody-dependent enhancement
CPSF3: cleavage and polyadenylation specific factor 3
DENV: Dengue virus
DF: dengue fever
DHS: dengue hemorrhagic fever
DSS: dengue shock syndrome
HLMs: human liver microsomes
JEV: Japanese encephalitis virus
LC-MS: Liquid Chromatography–Mass Spectrometry
PAMPA: Parallel Artificial Membrane Permeability Assay
SAA: sulfonyl anthranilic acid
SAR: Structure–Activity Relationship
SIs: selectivity indexes
UTRs: untranslated regions
WHO: World Health Organization
WNV: West Nile virus
YFV: Yellow Fever virus
ZIKV: Zika virus
