# Supplementary material for: Sulfonyl Anthranilic Acid Analogues Display Pan-Serotype Anti-Dengue Activity by Downregulating the Expression of Ribosomal Proteins Encoded by 5′-Terminal Oligopyrimidine Motif-Containing mRNA
Source: J Med Chem. 2026 Mar 10;69(6):7111–41. doi: 10.1021/acs.jmedchem.5c03414 (PMC13036783; doi:10.1021/acs.jmedchem.5c03414)
Supplement: Supplementary file 1 [file jm5c03414_si_001.pdf]

Sulfonyl Anthranilic Acid Analogues Display Pan-Serotype Anti-Dengue Activity by Downregulating the Expression of Ribosomal Proteins Encoded by 5′-Terminal Oligopyrimidine Motif-Containing mRNA

Chin Piaw Gwee[a,b,1], Tommaso Felicetti[c,1], Kitti Wing Ki Chan[a], Min Jie Alvin Tan[a], Muhammad Danial Bin Mohd Mazlan[a], Ciro Milite[d], Giacomo Pepe[d], Chiara Sarnari[c], Xiao Dan Ng[a], Wint Wint Phoo[a], Jasmine Hwee Yee Tan[b,e], Marcus G. Mah[a], Satoru Watanabe[a], Jing Xiu Huang[f,g], Serena Massari[c], Oriana Tabarrini[c], Stefano Sabatini[c], Pietro Campiglia[d], Gianluca Sbardella[d], Gavin J.D. Smith,[a] Sylvie Alonso[b,e,h], Alfred Xuyang Sun[f], Radoslaw M. Sobota[i], Subhash G. Vasudevan,[a,j,]* and Giuseppe Manfroni[c,]*.

[a]Program in Emerging Infectious Diseases, Duke-NUS Medical School, 8 College Road, 169857, Singapore

[b]Infectious Diseases Translational Research Programme, Department of Microbiology & Immunology, Yong Loo Lin School of Medicine, 117545, Singapore

[c]Dipartimento di Scienze Farmaceutiche, Università degli Studi di Perugia, Via Del Liceo, 1-06123, Perugia, Italy

[d]Dipartimento di Farmacia, Università degli Studi di Salerno, Via Giovanni Paolo II, 132, 84084 Fisciano, Italy

[e]Immunology programme, Life Sciences Institute, National University of Singapore, 117456, Singapore

[f]Program in Neuroscience and Behavioral Disorders, Duke-NUS Medical School, 8 College Road, 169857, Singapore

[g]Department of Anesthesiology, State Key Laboratory of Oncology in South China, Guangdong Provincial Clinical Research Center for Cancer, Sun Yat-sen University Cancer Center, Guangzhou, 510060, Guangdong, People's Republic of China.

[h]Communicable Diseases Agency, 307684, Singapore.

[i]Institute of Molecular and Cell Biology, Agency for Science (IMCB), Technology and Research (A*STAR),138673, Singapore

[j]Institute for Biomedicine and Glycomics, Griffith University, Queensland, 4222, Australia

[1]Co-first authors

*Co-corresponding authors: subhash.vasudevan@duke-nus.edu.sg (SGV), giuseppe.manfroni@unipg.it (GM)

Contents Pages

Figure S1. Heat map analysis of protein expressions following DENV infection S1

Figure S2. Validation of mass spectrometry hits by western blotting S2

Figure S3. Development of dorsal forebrain organoids derived from H9 embryonic stem cells S3-4

Figure S4. Dose-response inhibition of compound **3** against the four DENV serotypes S4

Figure S5. Impact of SAA compounds on wild-type and mutant 5′-TOP Flag-CPSF3 expression level S5

Figure S6. Investigate the impact of compound **1** on mTOR signaling S6

Figure S7. Assessment of metabolic stability of compounds **1** and **7** after incubation with HLMs and LC-HRMS/MS-based characterization of their metabolites S7-8

Figure S8. MS^2 spectra illustrating the proposed fragmentation pattern of metabolites derived from compound **1** S9-10

Figure S9. MS^2 spectra of metabolites derived from compound **7** after incubation with HLMs in the presence of NADPH S11-13

Figures S10-S77. ^{1}HNMR and ^{13}CNMR spectra of compounds **4-37** S14-S47

Figures S78-S111. HPLC traces of representative compounds S48-S81

Supplementary Methods S82-S83

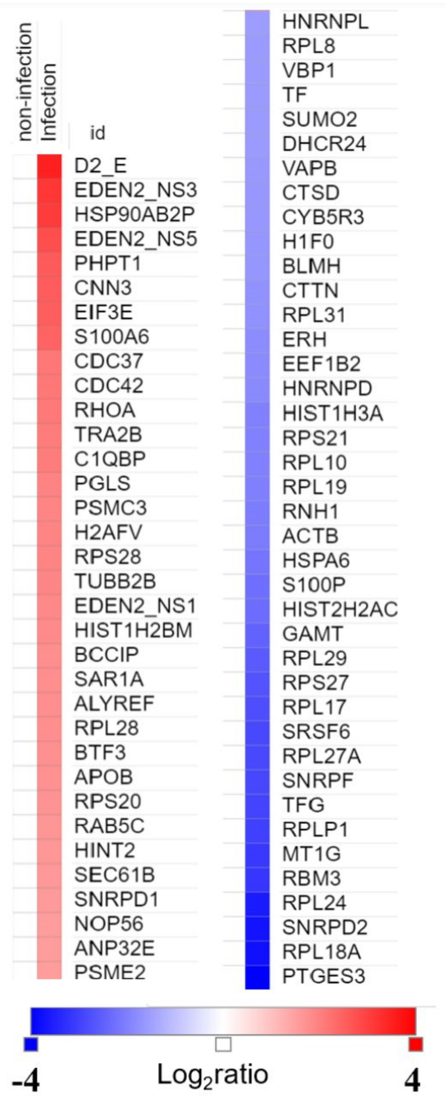

Figure S1: Heat map analysis of protein expressions following DENV infection. Huh-7 cells were infected with DENV-2 at MOI 5 for 24 h. The cells were lysed, and protein abundance was quantified by LC-MS. Proteins encoded in blue are those down-regulated (log2 fold change ≤ -1.5) after DENV infection, while proteins encoded in red are those up-regulated (log2 fold change ≥ 1.5) following infection.

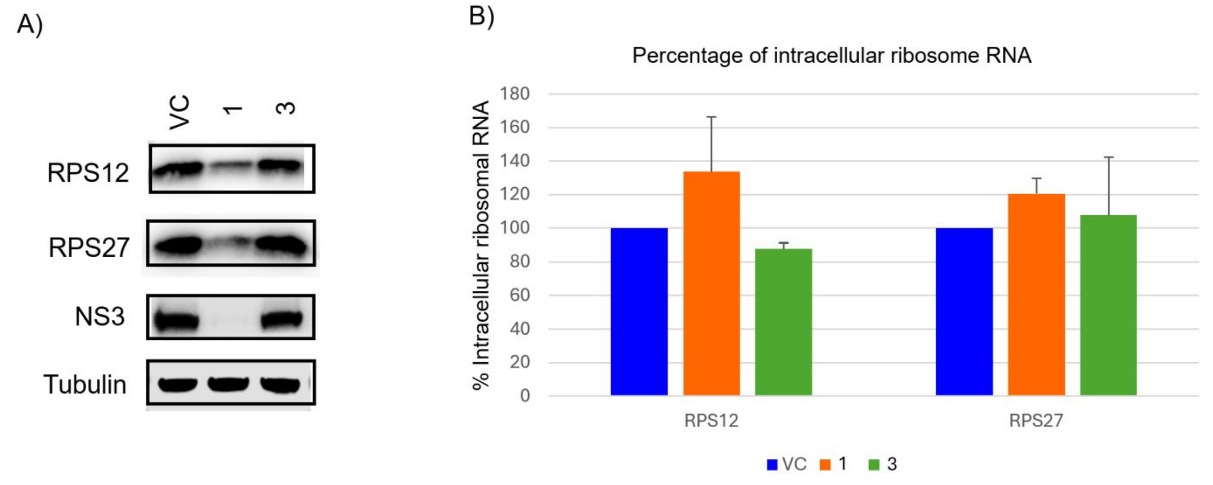

Figure S2. Validation of mass spectrometry hits by western blotting. Huh-7 cells were infected with DENV2 at MOI 1 and subsequently treated with compounds **1** or **3** at a concentration of 10 µM for 24 h. Following treatment, the cells were lysed and a total 10 µg of protein from each sample was analysed by Western blotting using anti-RPS12 and anti-RPS27 antibodies. **A)** Western blot analysis showing the respective ribosomal protein expression levels following SAA treatment. **B)** Transcript levels of the respective ribosomal proteins following SAA treatment. Data represent the mean±SD from technical duplicate in a single experiment.

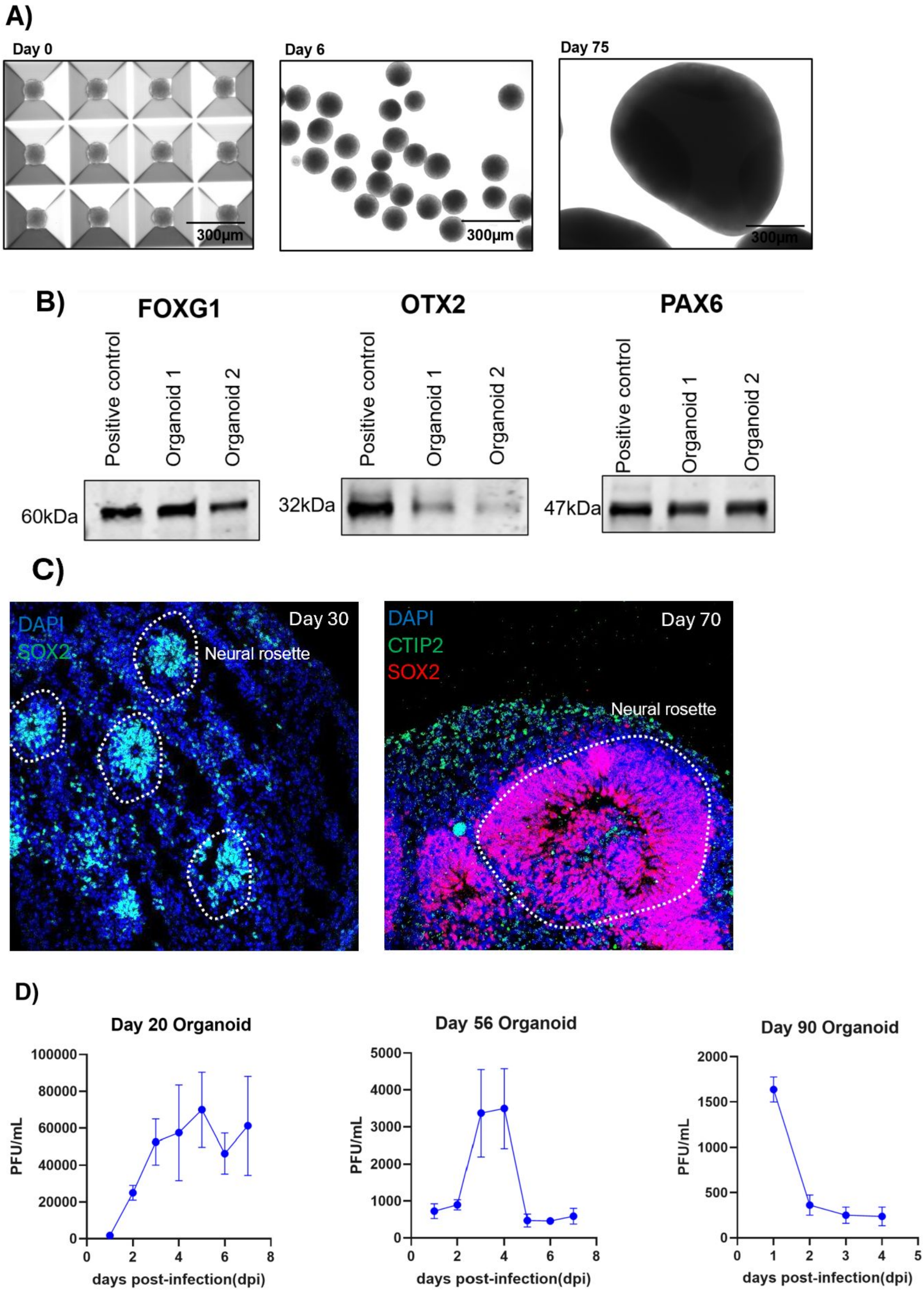

Figure S3. Development of dorsal forebrain organoids derived from H9 embryonic stem cells. Human embryonic stem cell line H9 was differentiated into dorsal forebrain organoid using STEMdiff Dorsal Forebrain Organoid Differentiation Kit (STEMCELL Technologies) according to manufacturer's instruction. The organoids development was monitored visually as well as using specific markers to ensure proper differentiation along the cortical forebrain lineage. **A)** Representative light microscope images showing

forebrain organoids development over time. **B)** Western blot analysis of early-stage forebrain markers including FOXG1, OTX2 and PAX6. **C)** Immunostaining of organoid with DAPI, SOX2 and CTIP2 showing formation of neuronal rosette indicating the differentiation and growth of neuronal cells. Different stage forebrain organoids were exposed to ZIKV H/PF/2013 at MOI 1 and the cultured supernatants harvested at the indicated timepoints were subjected to plaque quantification. **D)** Kinetics of infectious virus production from ZIKV infected forebrain organoids as measured by plaque assay. Line graphs showing virus titer over time, represented as plaque forming unit per milliliter (pfu/mL).

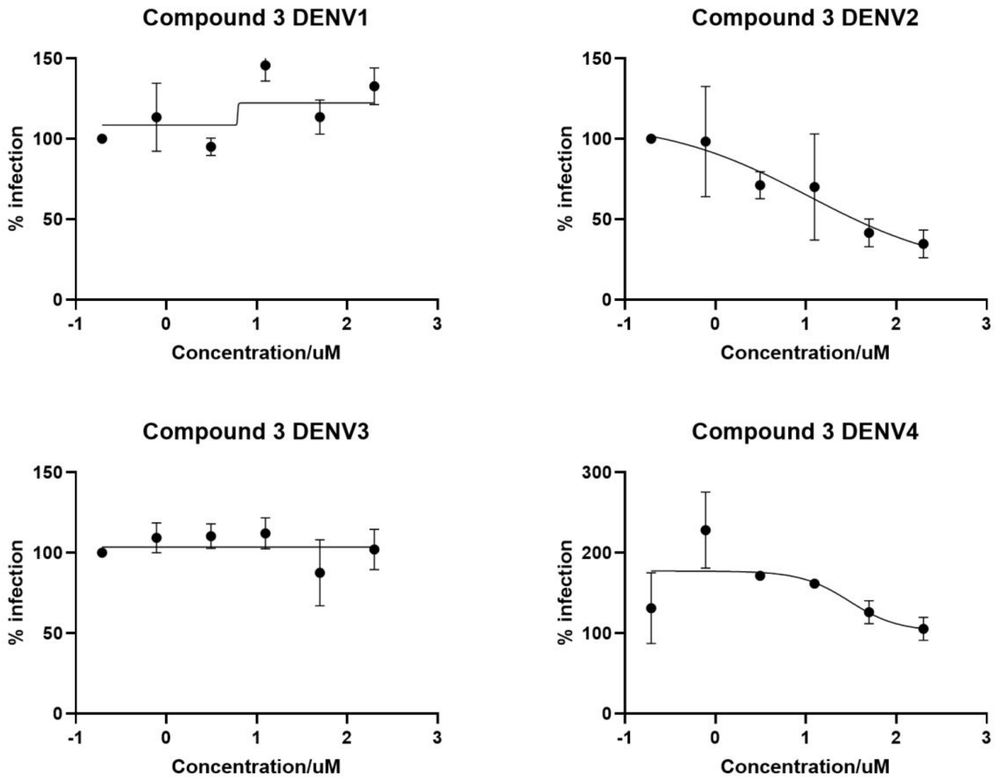

Figure S4. Dose-response inhibition of compound 3 against the four DENV serotypes. Huh-7 cells were infected with the DENV serotypes at MOI 0.3 followed by treatment with increasing concentrations of compound **3** ranging from 0.195, 0.781, 3.125, 12.5, 50 to 200 µM for 48 h. The antiviral activity is determined by the reduction in virus titer as measured by standard BHK-21 plaque assay. No viral reduction was observed for DENV-1, DENV-3 and DENV-4, even at the highest concentration tested 200 µM. Compound **3** exhibited moderate activity against DENV-2 with an apparent EC_{50} value of ~40-50 µM.

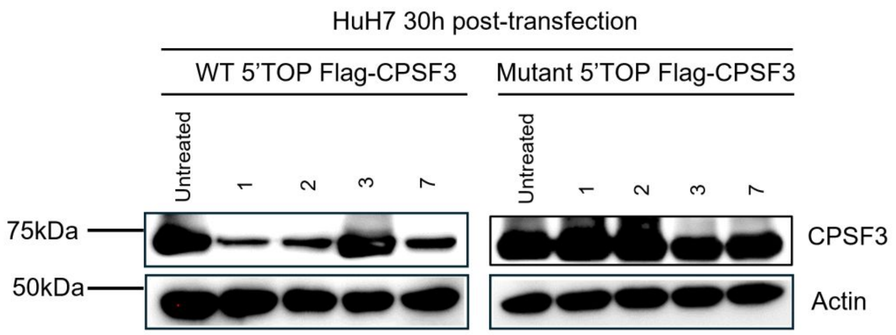

Figure S5. Impact of SAA compounds on wild-type and mutant 5′-TOP Flag-CPSF3 expression level. Huh-7 cells were transfected with wild-type 5′-TOP-CPSF3 and mutant 5′-TOP-CPSF3 plasmids. 6 h post-transfection, the cells were treated with compound **1–3** and **7** at 10 µM and further incubated for 24 h. Cells were lysed and 10 µg of the total lysate was analysed by Western blot using anti-Flag antibodies. Actin was included as the loading control.

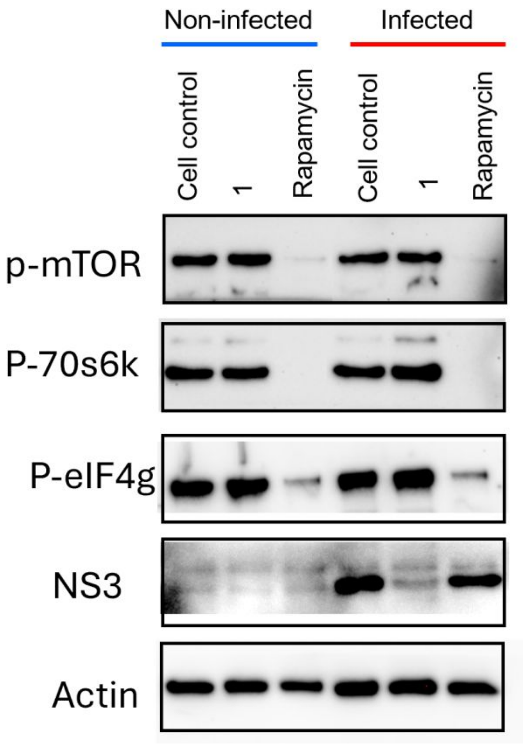

Figure S6. Investigate the impact of Compound 1 on mTOR signaling. Huh-7 cells were infected with DENV and subsequently treated with compound **1** and rapamycin at a concentration of 10 µM for 24 h. The cells were lysed and the lysates were analyzed on western blot using specific antibodies. Western blot analysis showing the expression levels of proteins involved in mTOR signaling following compound **1** and rapamycin treatment.

A)

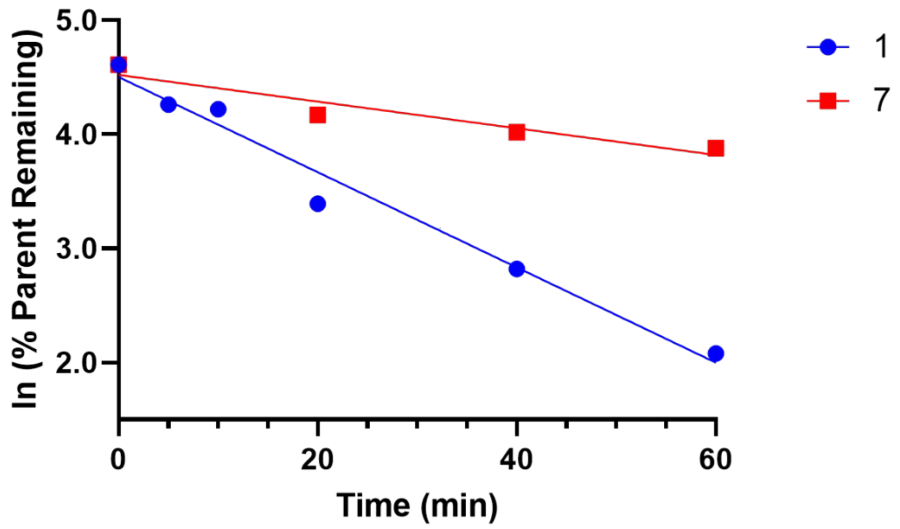
5.0
4.0
3.0
2.0
ln (% Parent Remaining)
0
20
40
60
Time (min)
1
7

B)

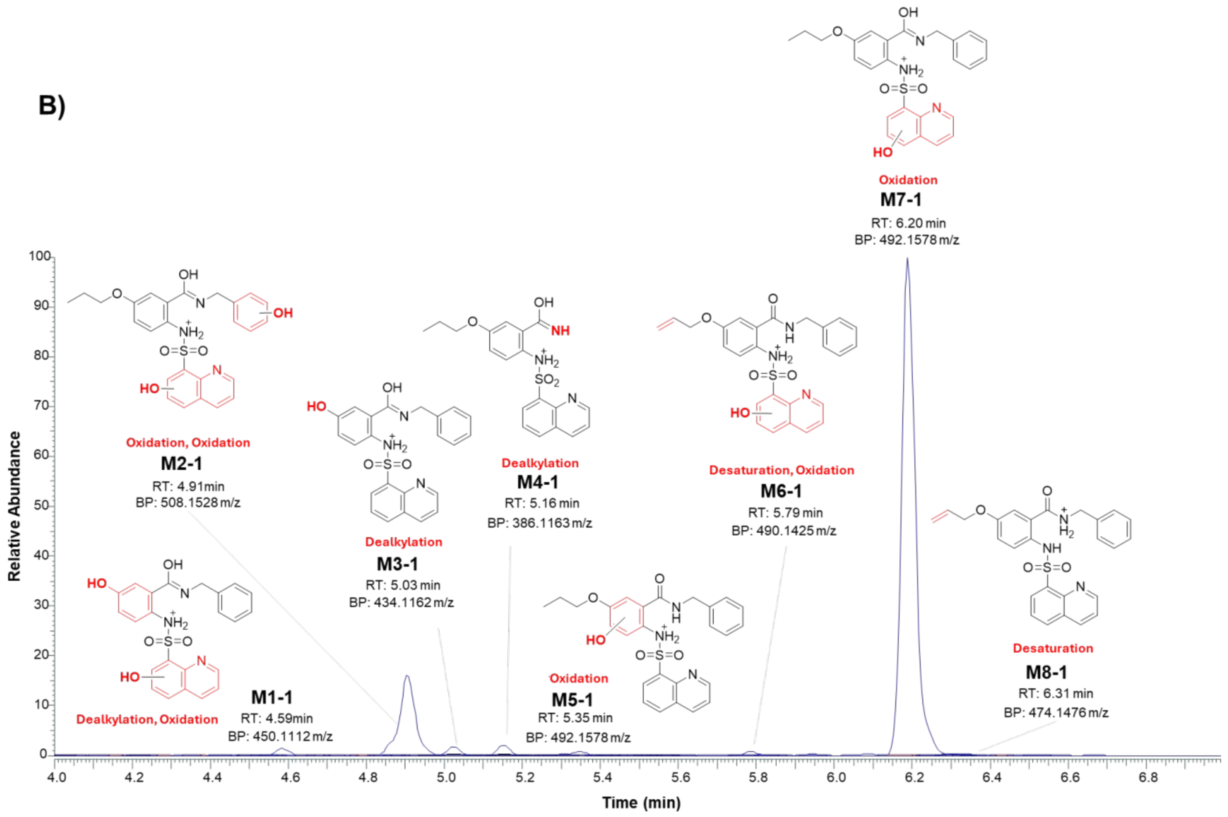
Oxidation
M7-1
RT: 6.20 min
BP: 492.1578 m/z
Oxidation, Oxidation
M2-1
RT: 4.91min
BP: 508.1528 m/z
Dealkylation
M3-1
RT: 5.03 min
BP: 434.1162 m/z
Dealkylation
M4-1
RT: 5.16 min
BP: 386.1163 m/z
Desaturation, Oxidation
M6-1
RT: 5.79 min
BP: 490.1425 m/z
Dealkylation, Oxidation
M1-1
RT: 4.59min
BP: 450.1112 m/z
Oxidation
M5-1
RT: 5.35 min
BP: 492.1578 m/z
Desaturation
M8-1
RT: 6.31 min
BP: 474.1476 m/z
Relative Abundance
Time (min)

C)

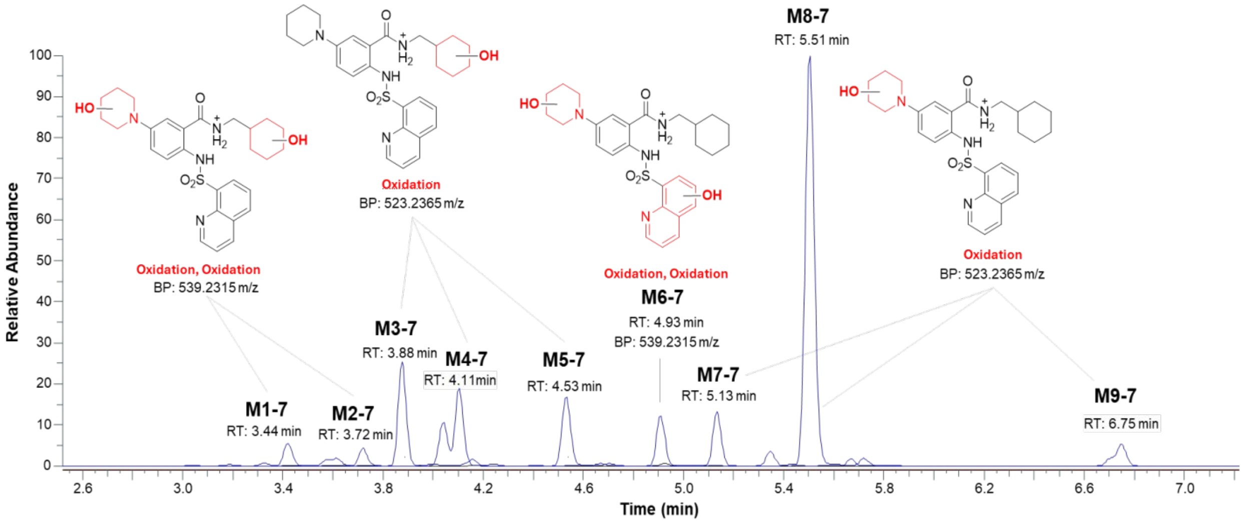

Figure S7: Assessment of metabolic stability of compounds 1 and 7 after incubation with HLMs and LC-HRMS/MS-based characterization of their metabolites. (A) The plot shows the natural logarithm of the percentage of parent compound remaining (ln % parent remaining) versus incubation time (0, 20, 40, and 60 min). Linear regression analysis indicates that compound **1** undergoes faster metabolic degradation than compound **7**, demonstrating its lower metabolic stability under the tested conditions. Since compound **1** was highly metabolized, two additional incubation time points (5 and 10 min) were included to obtain a more accurate estimation of its t1/2. (**B**) LC-HRMS/MS profile of metabolites generated after microsomal incubation of compound **1**. Proposed structures and biotransformation pathways are highlighted in red. The identified metabolites arise mainly from phase I reactions such as oxidation, desaturation, and N-dealkylation, indicating extensive NADPH-dependent oxidative metabolism. (**C**) LC-HRMS/MS profile of compound **7** metabolites after HLM incubation. The chromatogram displays oxidative metabolites, with proposed structures and biotransformation pathways highlighted in red. The predominant metabolic reactions involve mono- and di-oxidation processes.

A)
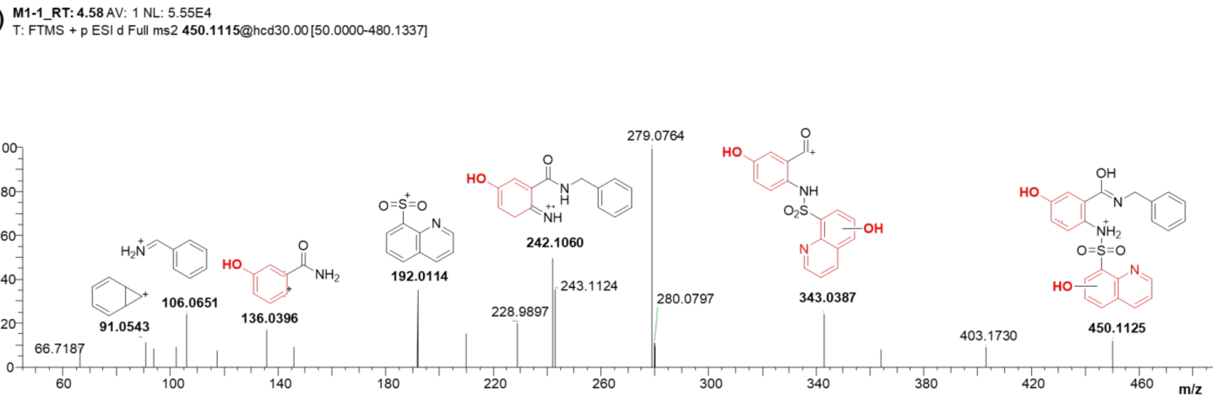
M1-1_RT: 4.58 AV: 1 NL: 5.55E4
T: FTMS + p ESI d Full ms2 450.1115@hcd30.00 [50.0000-480.1337]
Relative Abundance
m/z
279.0764
91.0543
106.0651
136.0396
192.0114
242.1060
243.1124
228.9897
280.0797
343.0387
403.1730
450.1125
66.7187

B)
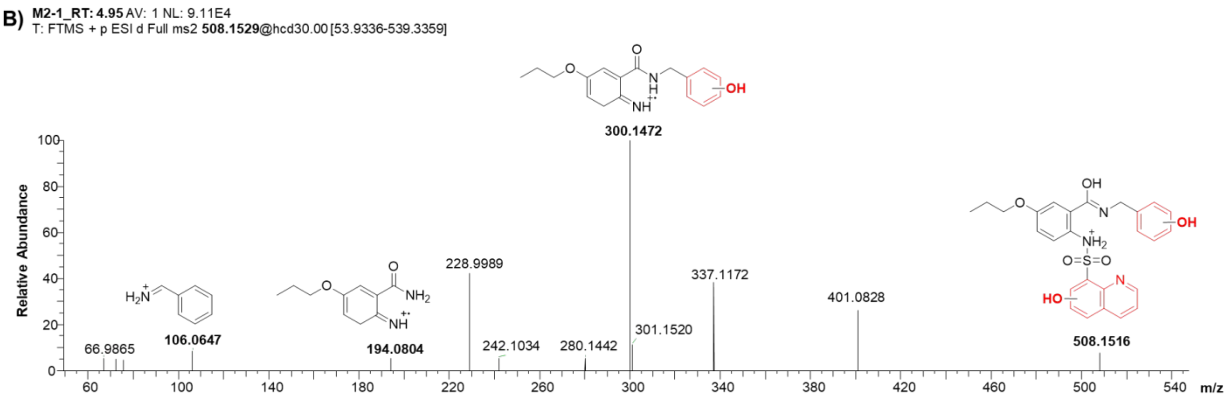
M2-1_RT: 4.95 AV: 1 NL: 9.11E4
T: FTMS + p ESI d Full ms2 508.1529@hcd30.00 [53.9336-539.3359]
Relative Abundance
m/z
300.1472
106.0647
194.0804
228.9989
242.1034
280.1442
301.1520
337.1172
401.0828
508.1516
66.9865

C)
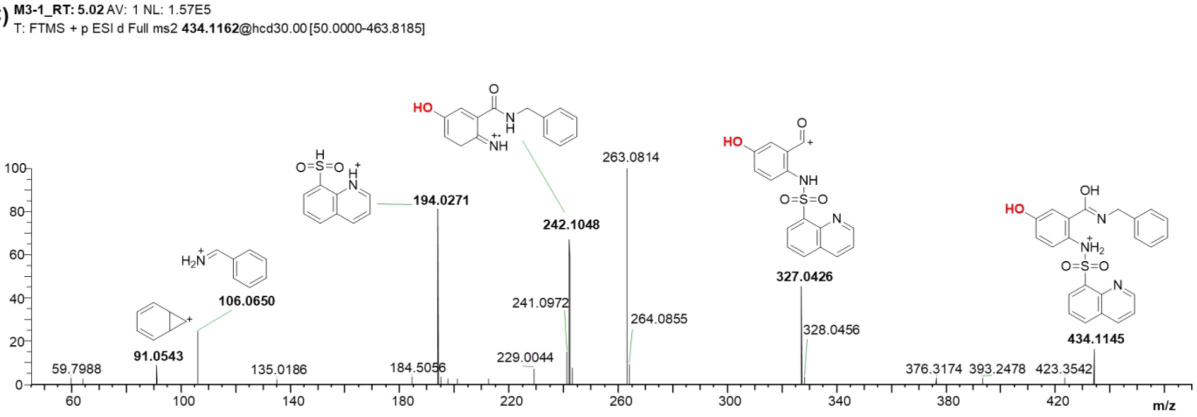
M3-1_RT: 5.02 AV: 1 NL: 1.57E5
T: FTMS + p ESI d Full ms2 434.1162@hcd30.00 [50.0000-463.8185]
Relative Abundance
m/z
194.0271
242.1048
263.0814
106.0650
91.0543
241.0972
264.0855
327.0426
328.0456
434.1145
59.7988
135.0186
184.5056
229.0044
376.3174
393.2478
423.3542

D)
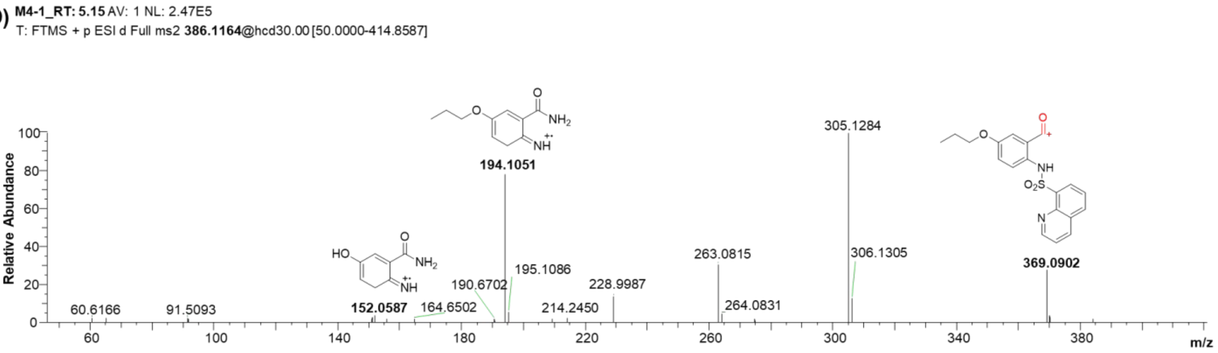
M4-1_RT: 5.15 AV: 1 NL: 2.47E5
T: FTMS + p ESI d Full ms2 386.1164@hcd30.00 [50.0000-414.8587]
Relative Abundance
m/z
194.1051
305.1284
369.0902
152.0587
60.6166
91.5093
164.6502
190.6702
195.1086
214.2450
228.9987
263.0815
264.0831
306.1305

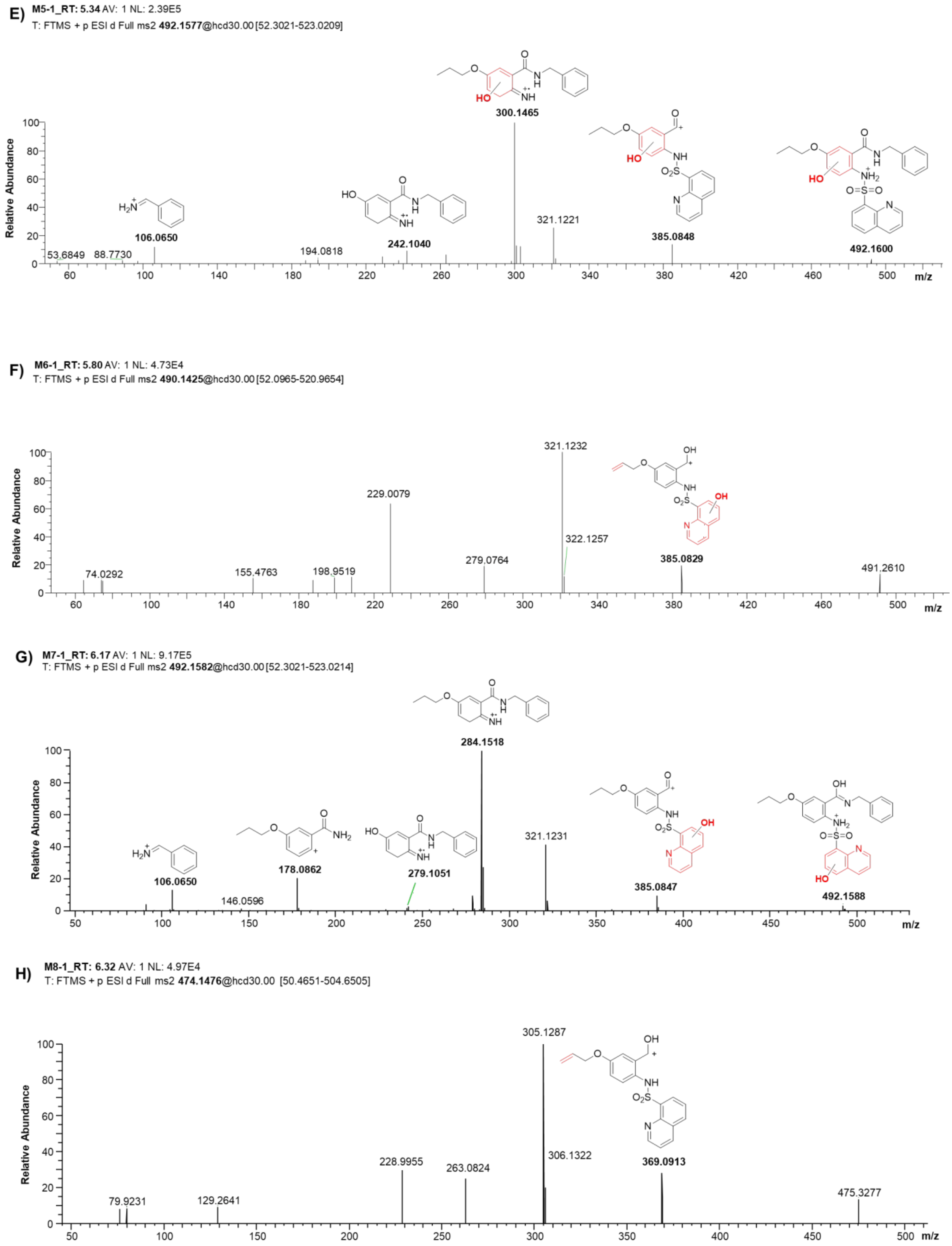

Figure S8: MS^2 spectra illustrating the proposed fragmentation pattern of metabolites derived from compound **1**: M1-1 (**A**, dealkylation and oxidation), M2-1 (**B**, double oxidation), M3-1 (**C**, dealkylation), M4-1 (**D**, dealkylation), M5-1 (**E**, oxidation), M6-1 (**F**, desaturation and oxidation), M7-1 (**G**, oxidation), and M8-1 (**H**, desaturation). Proposed structures and key fragmentations are highlighted in red.

A)
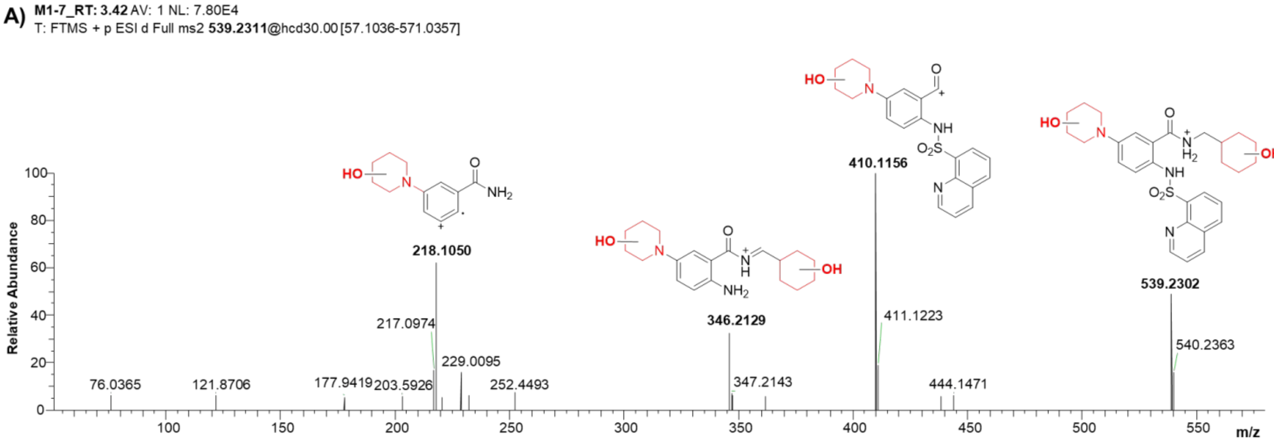
M1-7_RT: 3.42 AV: 1 NL: 7.80E4
T: FTMS + p ESI d Full ms2 539.2311@hcd30.00 [57.1036-571.0357]
Relative Abundance
m/z
218.1050
217.0974
229.0095
252.4493
76.0365
121.8706
177.9419
203.5926
346.2129
347.2143
410.1156
411.1223
444.1471
539.2302
540.2363

B)
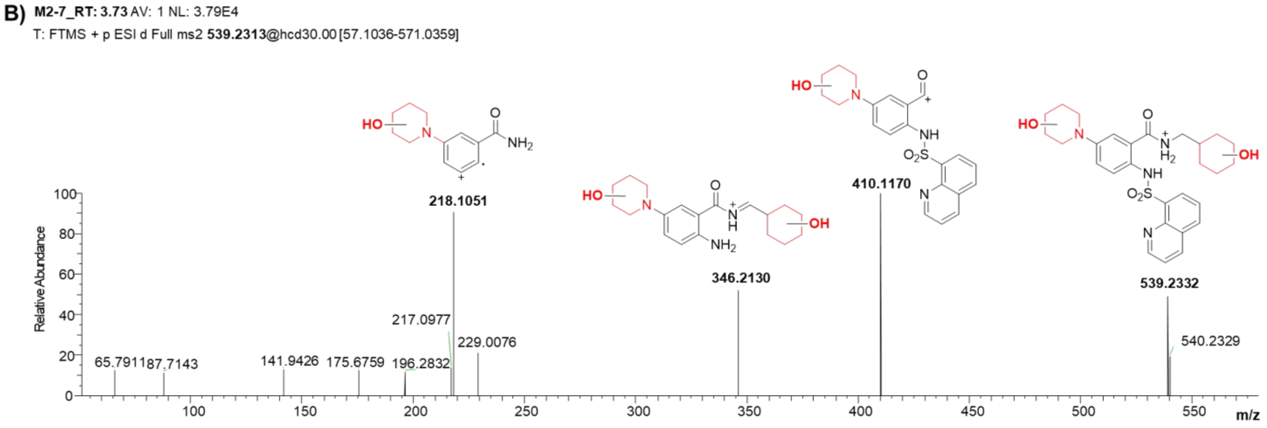
M2-7_RT: 3.73 AV: 1 NL: 3.79E4
T: FTMS + p ESI d Full ms2 539.2313@hcd30.00 [57.1036-571.0359]
Relative Abundance
m/z
218.1051
217.0977
229.0076
65.7911
87.7143
141.9426
175.6759
196.2832
346.2130
410.1170
539.2332
540.2329

C)
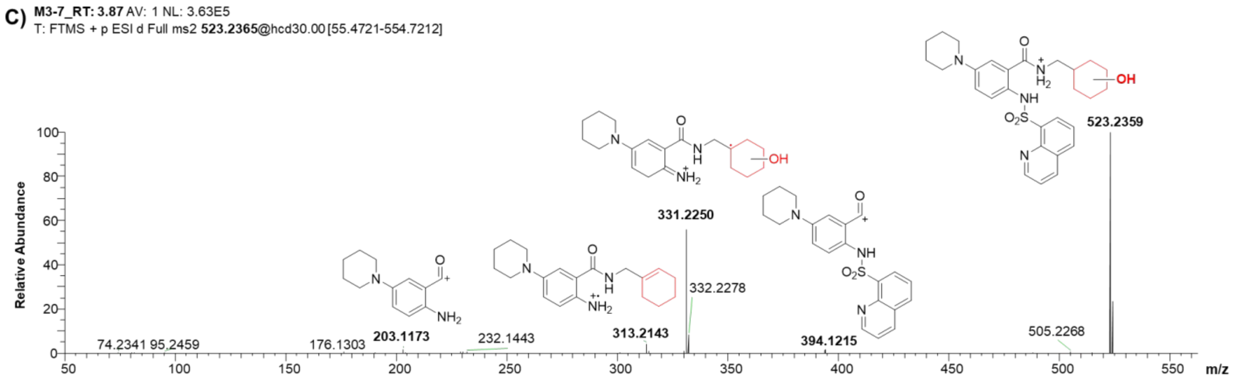
M3-7_RT: 3.87 AV: 1 NL: 3.63E5
T: FTMS + p ESI d Full ms2 523.2365@hcd30.00 [55.4721-554.7212]
Relative Abundance
m/z
523.2359
331.2250
332.2278
74.2341
95.2459
176.1303
203.1173
232.1443
313.2143
394.1215
505.2268

D)
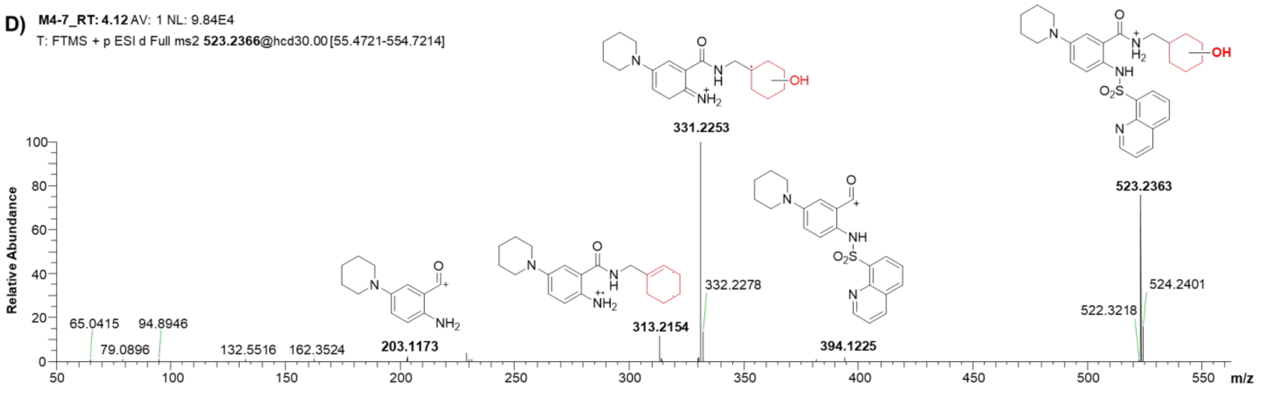
M4-7_RT: 4.12 AV: 1 NL: 9.84E4
T: FTMS + p ESI d Full ms2 523.2366@hcd30.00 [55.4721-554.7214]
Relative Abundance
m/z
331.2253
332.2278
523.2363
522.3218
524.2401
65.0415
94.8946
79.0896
132.5516
162.3524
203.1173
313.2154
394.1225

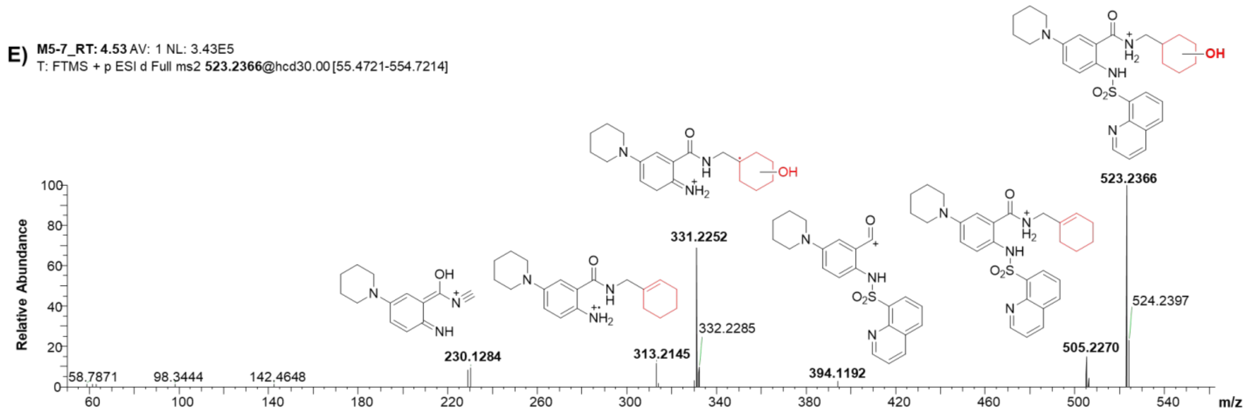
E)
M5-7_RT: 4.53 AV: 1 NL: 3.43E5
T: FTMS + p ESI d Full ms2 523.2366@hcd30.00 [55.4721-554.7214]
Relative Abundance
m/z
523.2366
524.2397
505.2270
394.1192
331.2252
332.2285
313.2145
230.1284
58.7871
98.3444
142.4648

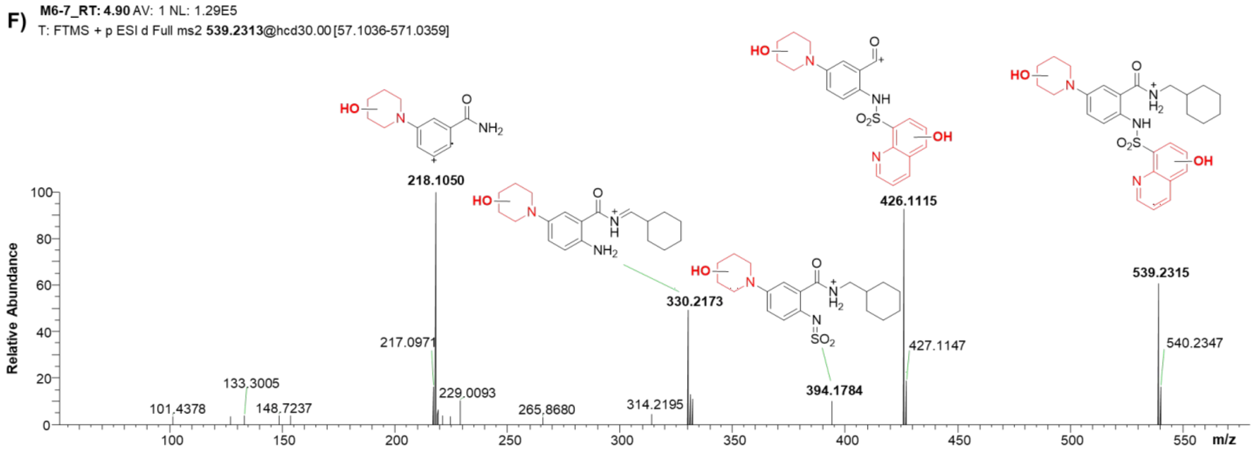
F)
M6-7_RT: 4.90 AV: 1 NL: 1.29E5
T: FTMS + p ESI d Full ms2 539.2313@hcd30.00 [57.1036-571.0359]
Relative Abundance
m/z
218.1050
426.1115
539.2315
540.2347
427.1147
330.2173
394.1784
217.0971
229.0093
265.8680
314.2195
133.3005
101.4378
148.7237

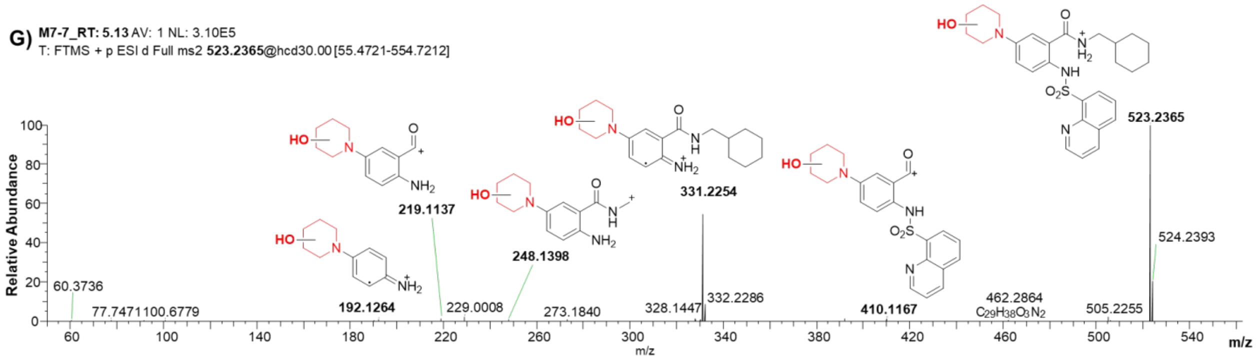
G)
M7-7_RT: 5.13 AV: 1 NL: 3.10E5
T: FTMS + p ESI d Full ms2 523.2365@hcd30.00 [55.4721-554.7212]
Relative Abundance
m/z
523.2365
524.2393
219.1137
331.2254
248.1398
192.1264
410.1167
60.3736
77.7471
100.6779
229.0008
273.1840
328.1447
332.2286
462.2864
C29H38O3N2
505.2255

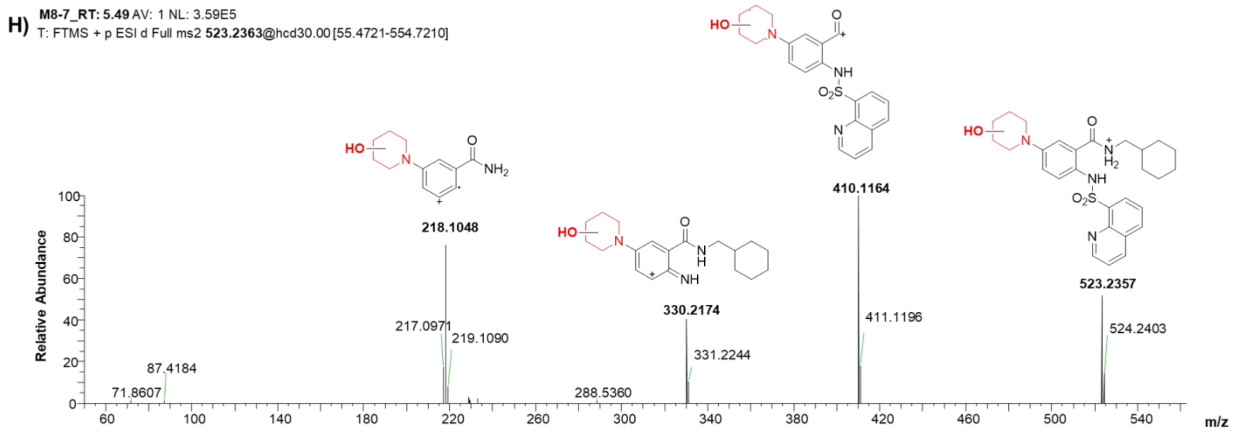
H)
M8-7_RT: 5.49 AV: 1 NL: 3.59E5
T: FTMS + p ESI d Full ms2 523.2363@hcd30.00 [55.4721-554.7210]
Relative Abundance
m/z
218.1048
410.1164
523.2357
330.2174
331.2244
411.1196
524.2403
217.0971
219.1090
87.4184
71.8607
288.5360

I)

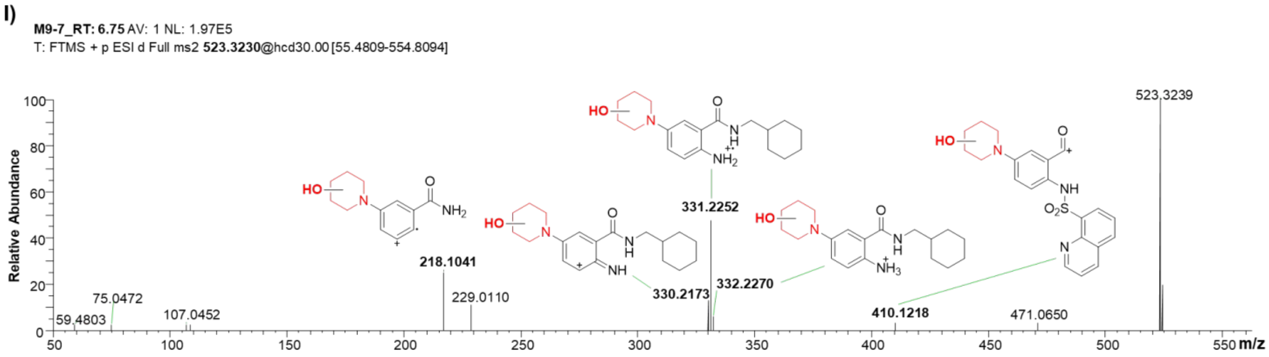

Figure S9: MS^2 spectra of metabolites derived from compound 7 after incubation with HLMs in the presence of NADPH. The spectra illustrate the characteristic fragment ions and proposed fragmentation pattern for metabolites M1-7 (**A**, double oxidation), M2-7 (**B**, double oxidation), M3-7 (**C**, oxidation), M4-7 (**D**, oxidation), M5-7 (**E**, oxidation), M6-7 (**F**, double oxidation), M7-7 (**G**, oxidation), M8-7 (**H**, oxidation), and M9-7 (**I**, oxidation). Proposed structures and key fragmentations are highlighted in red. The observed MS^2 patterns confirm the oxidative nature of the metabolites, consistent with mono- and di-hydroxylation reactions.

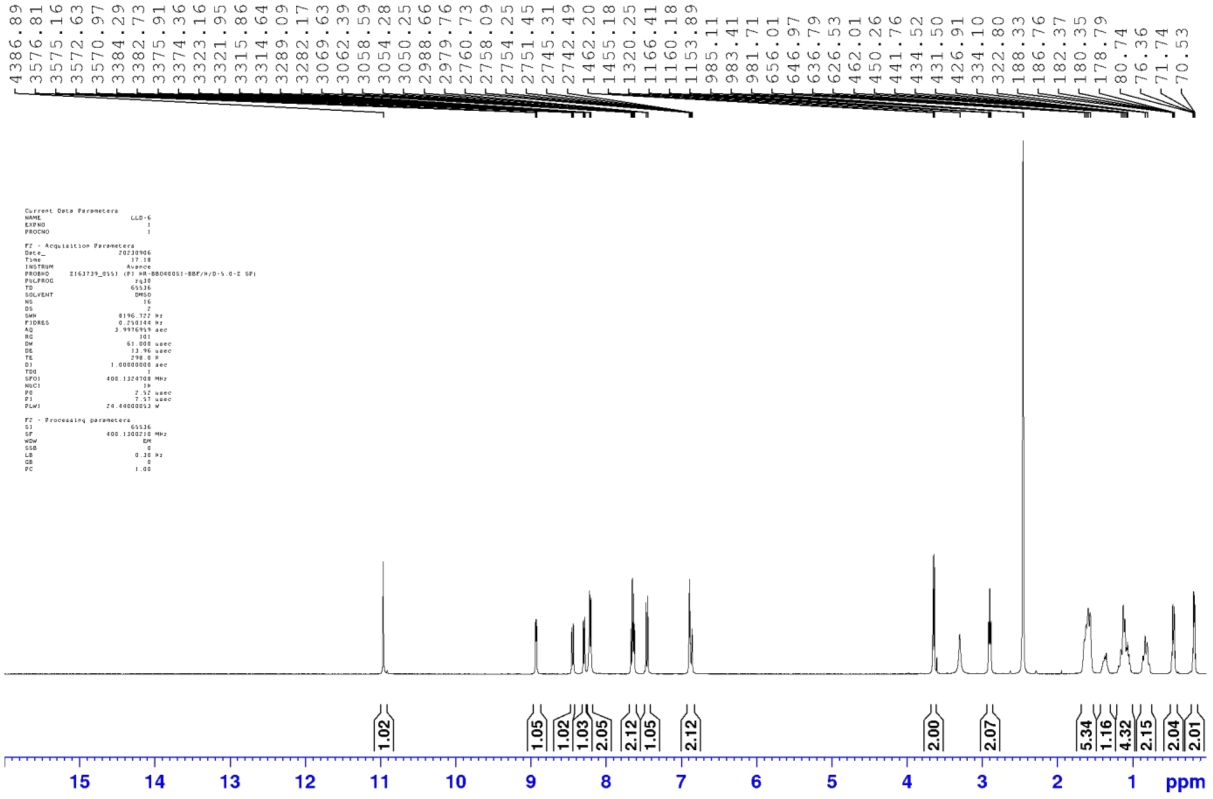

Figure S10. ^{1}HNMR spectrum of compound **4**.

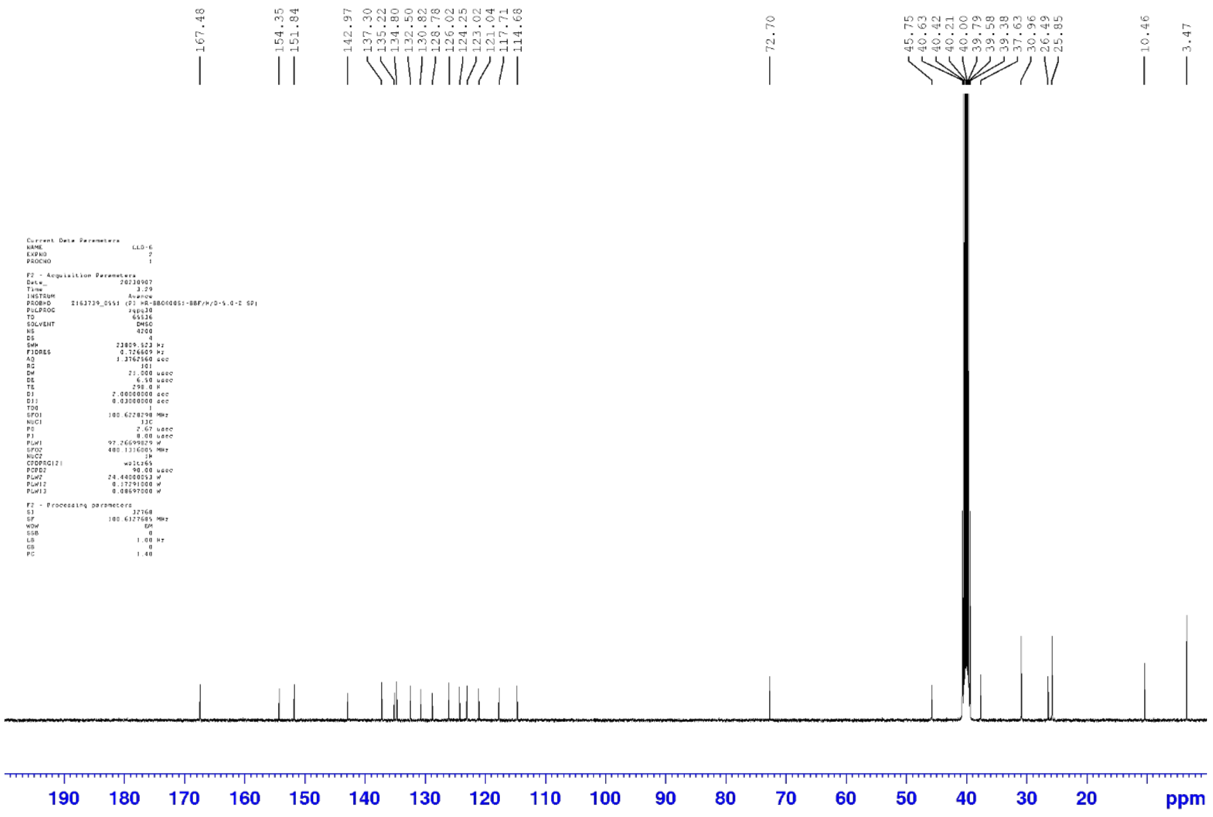

Figure S11. ^{13}CNMR spectrum of compound **4**.

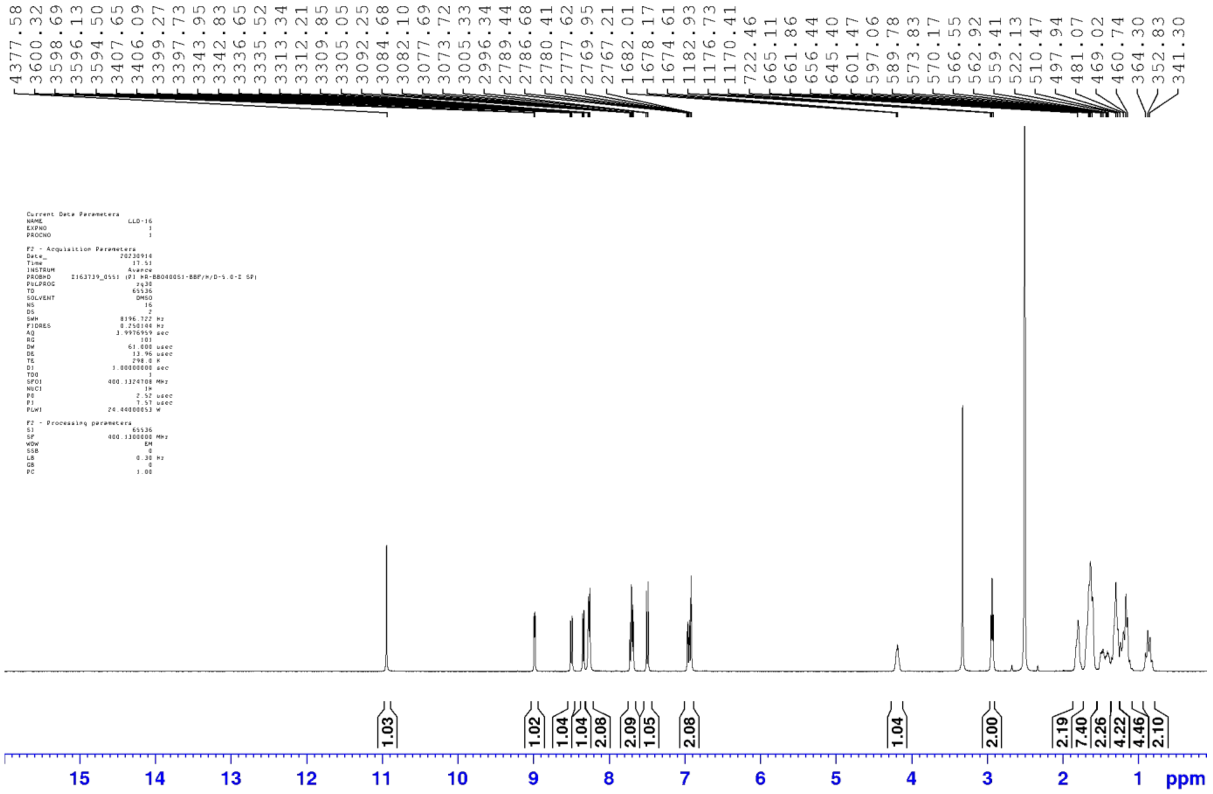

Figure S12. ^{1}HNMR spectrum of compound **5**.

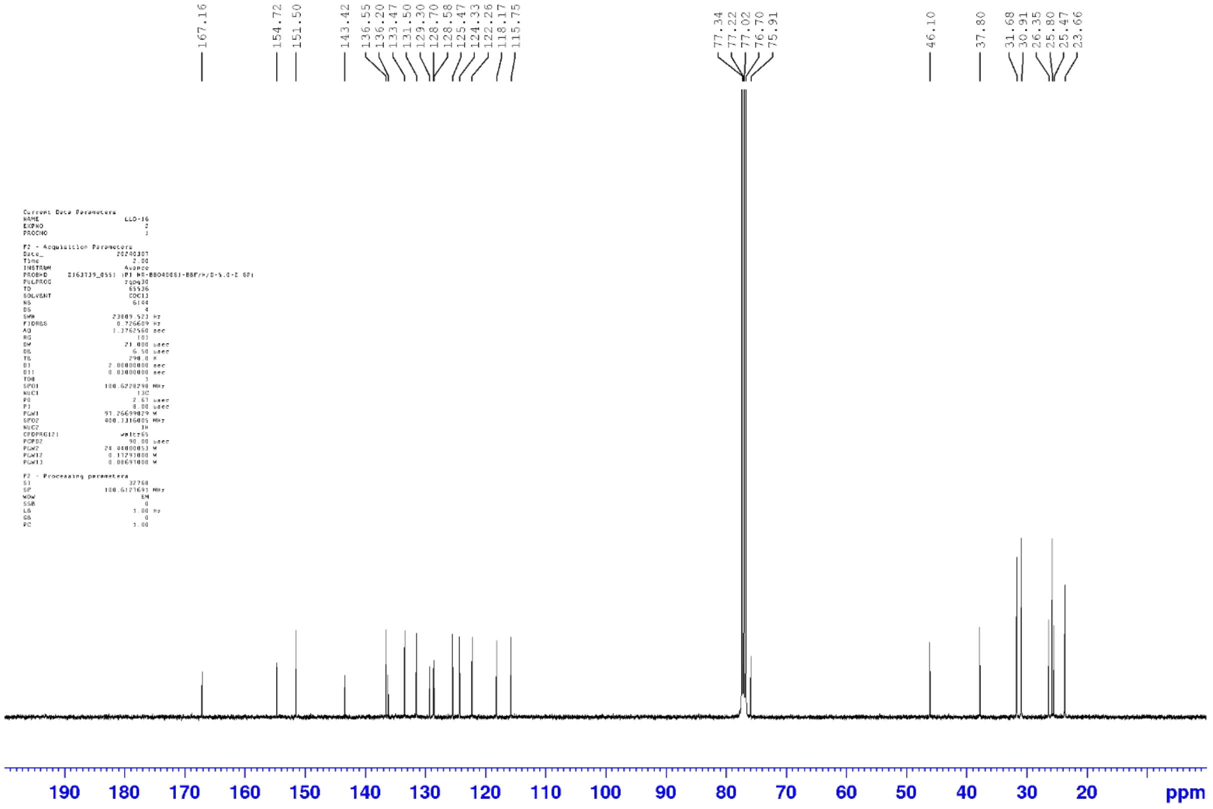

Figure S13. ^{13}CNMR spectrum of compound **5**.

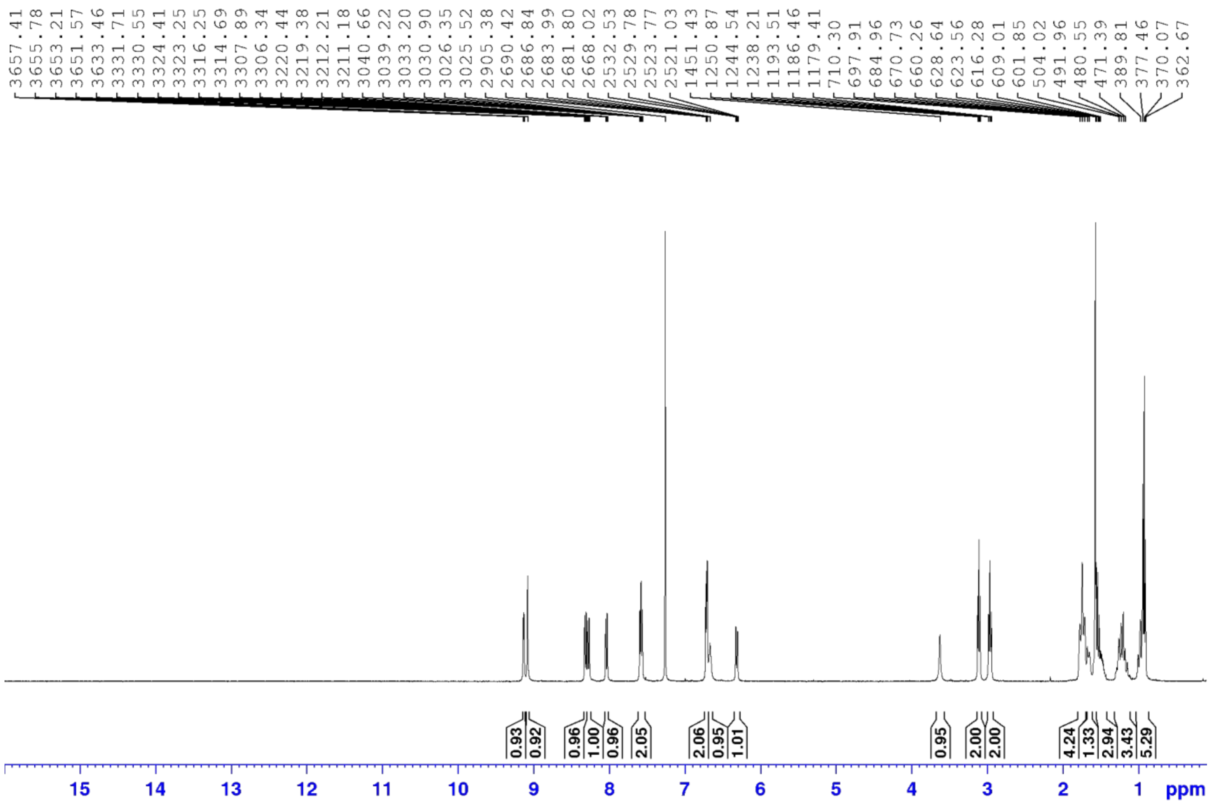

Figure S14. ^{1}HNMR spectrum of compound **6**.

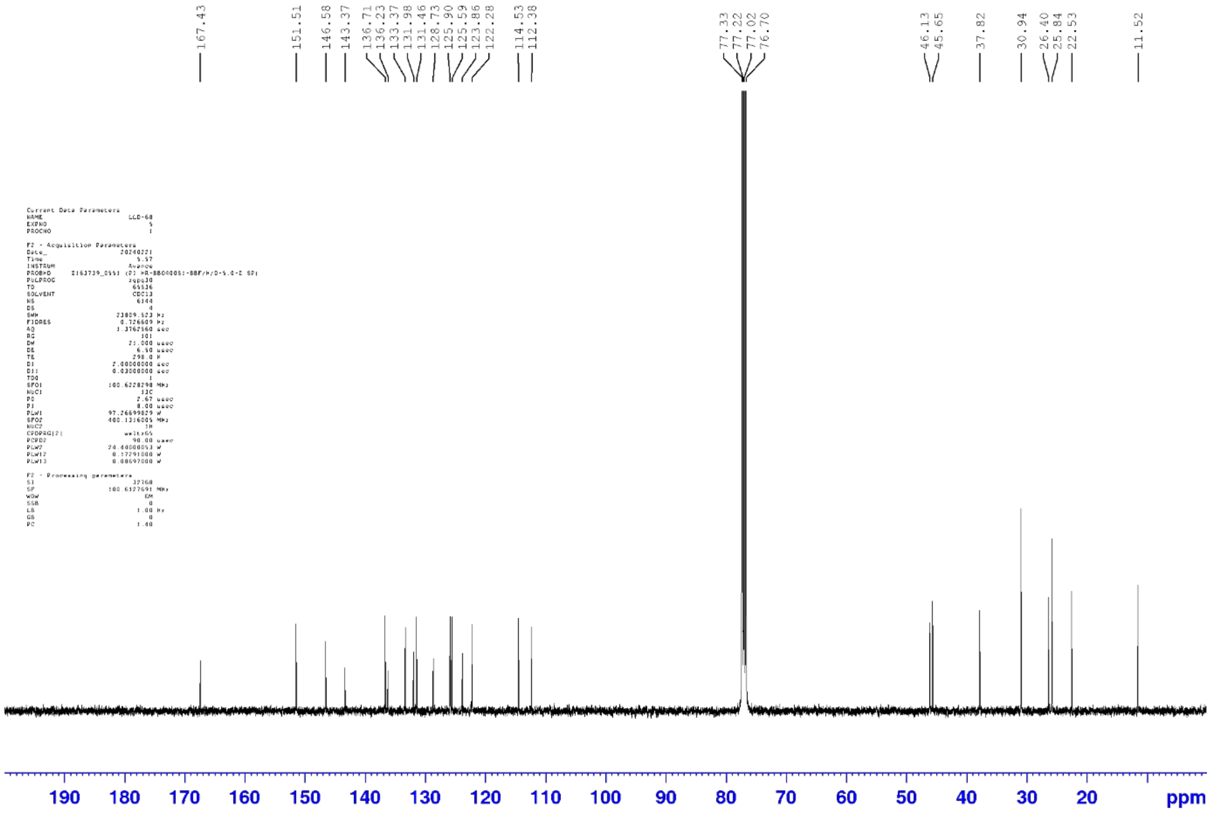

Figure S15. ^{13}CNMR spectrum of compound **6**.

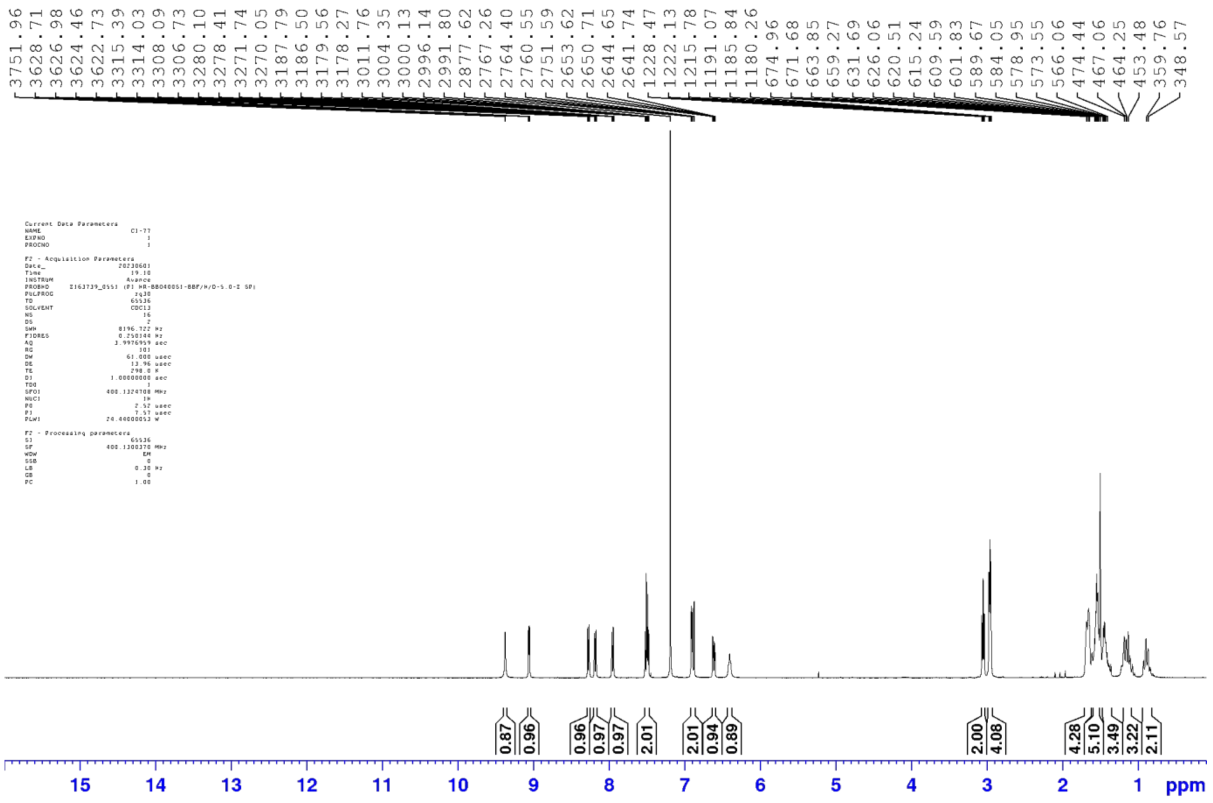

Figure S16. ^{1}HNMR spectrum of compound **7**.

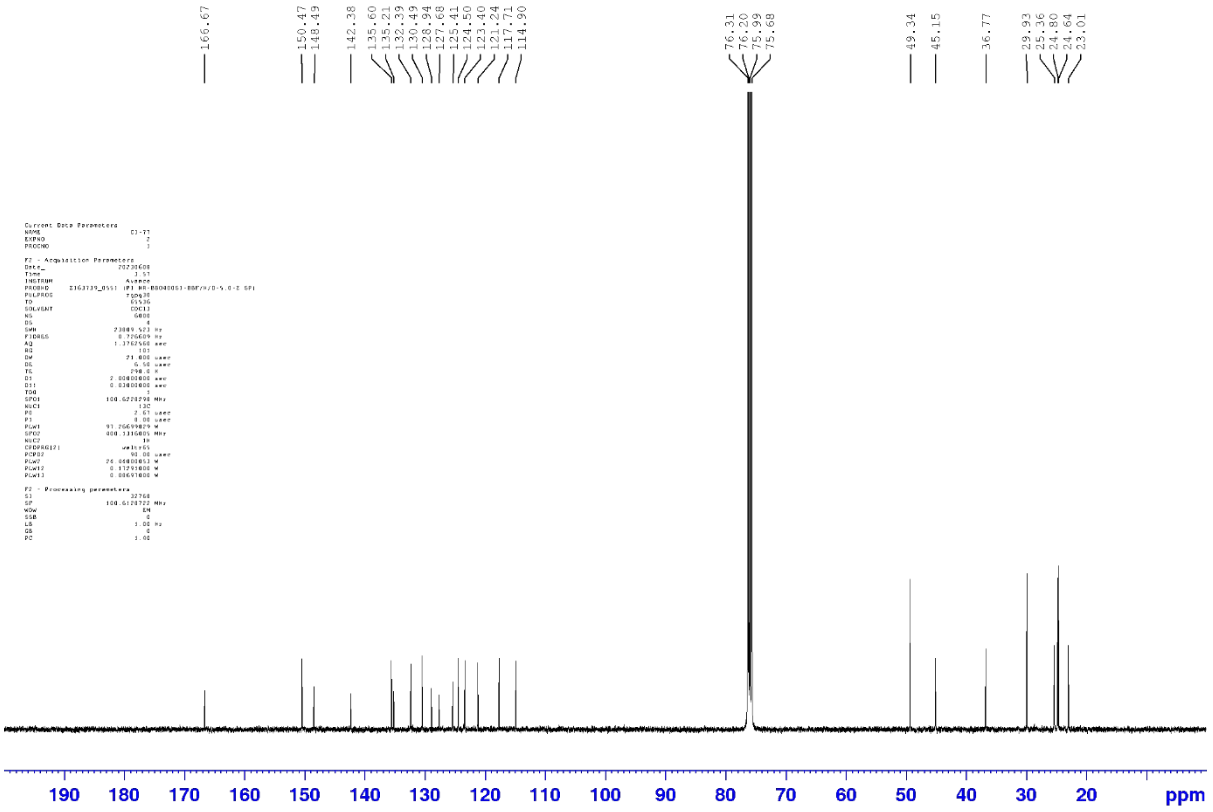

Figure S17. ^{13}CNMR spectrum of compound **7**.

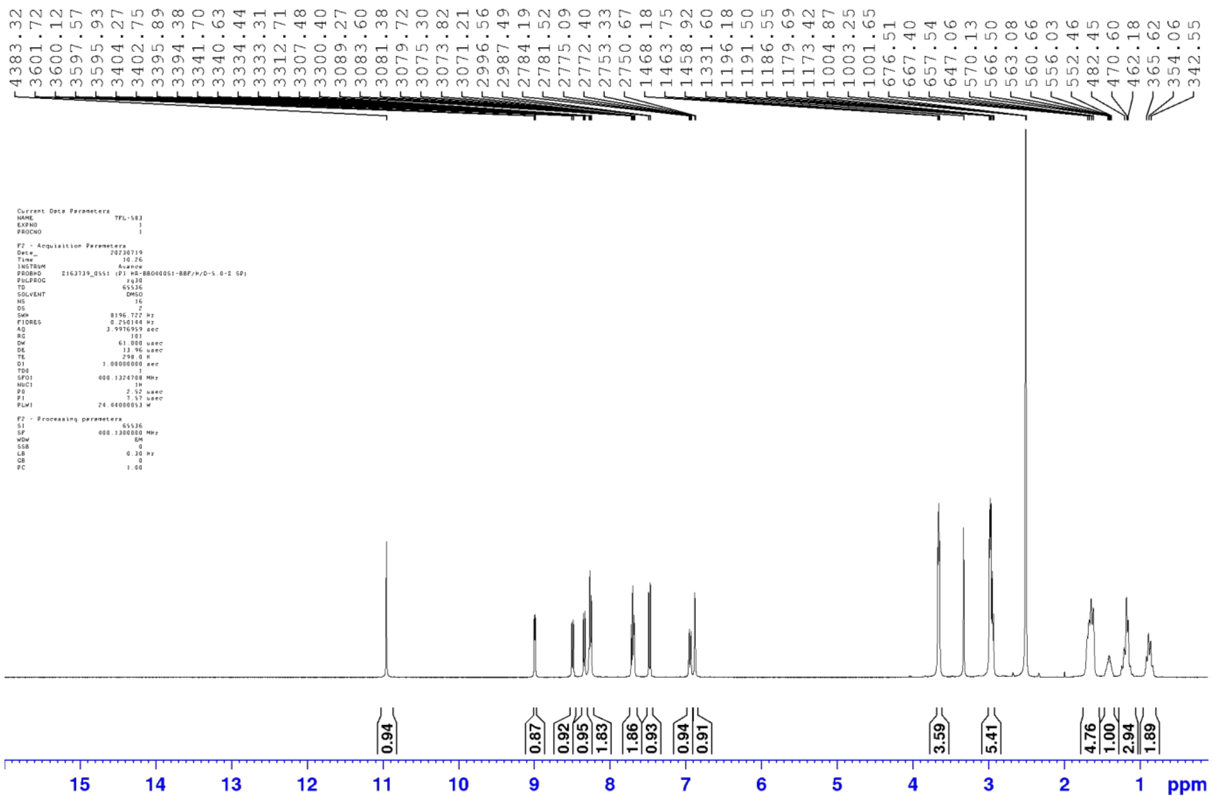

Figure S18. ^{1}HNMR spectrum of compound **8**.

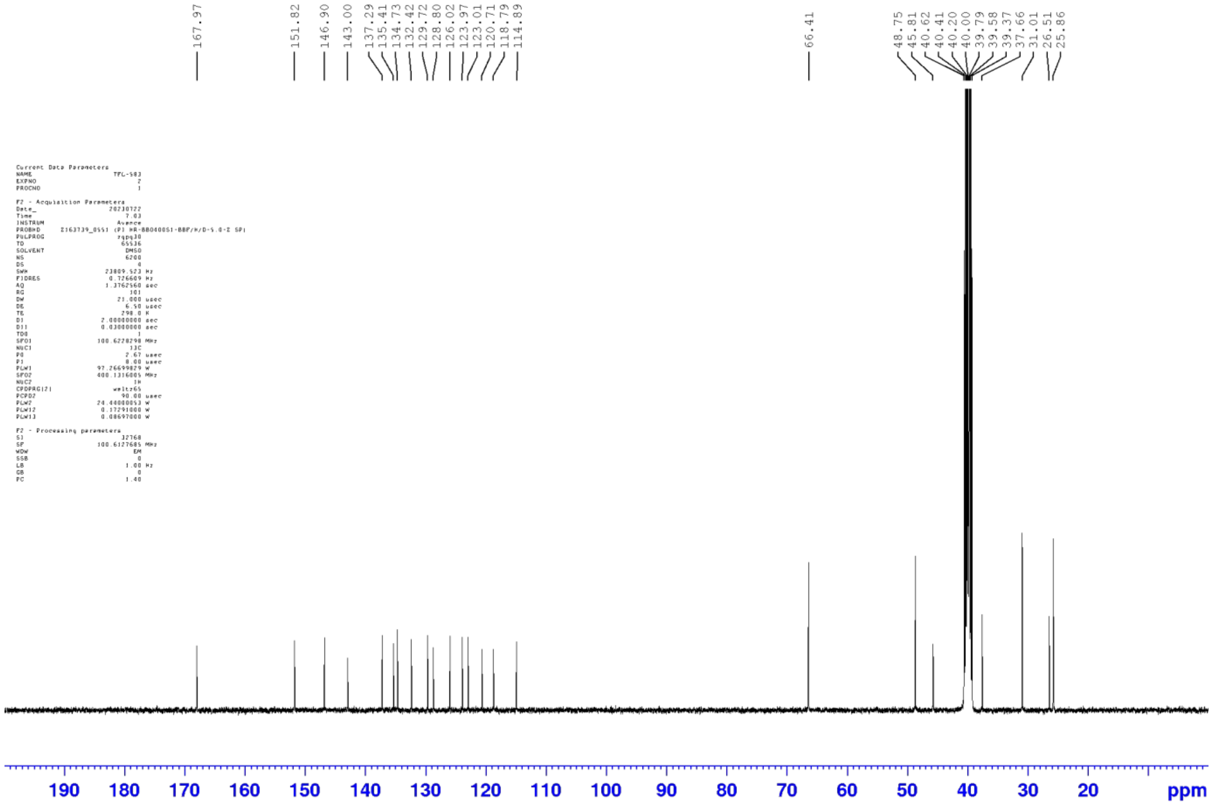

Figure S19. ^{13}CNMR spectrum of compound **8**.

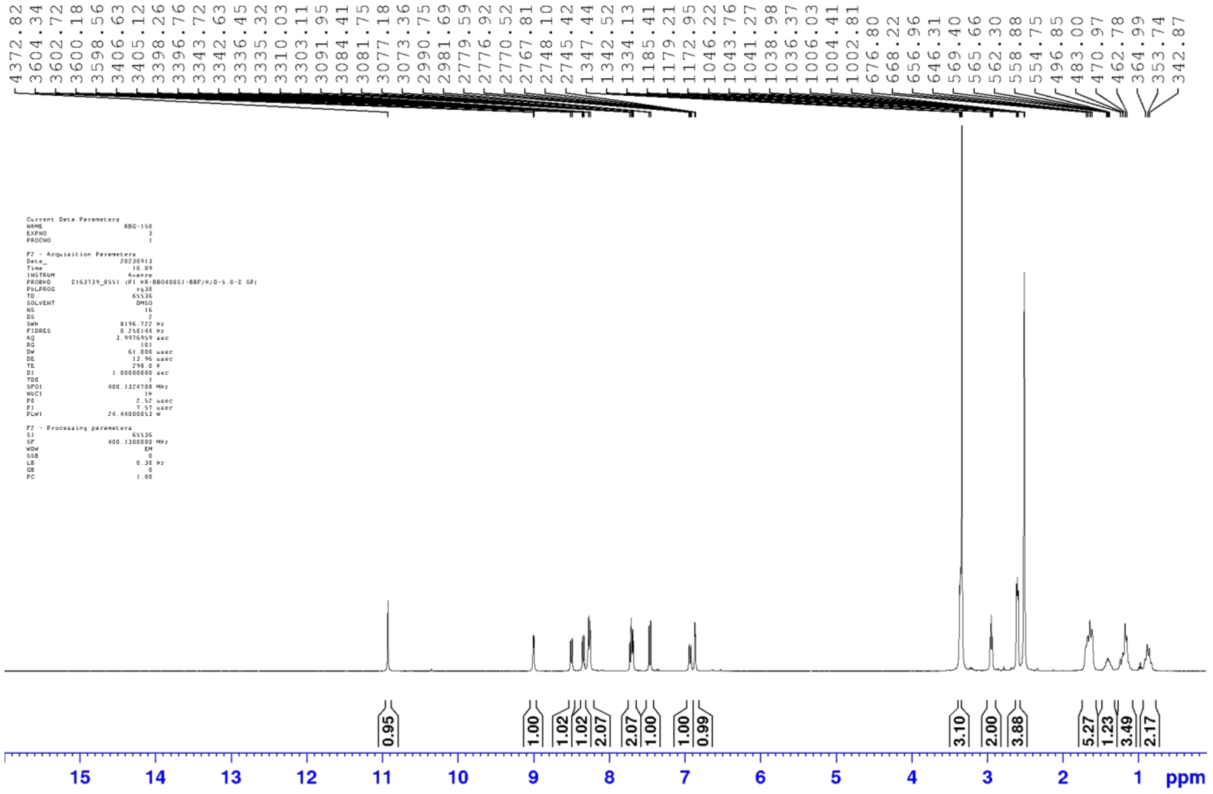

Figure S20. ^{1}HNMR spectrum of compound **9**.

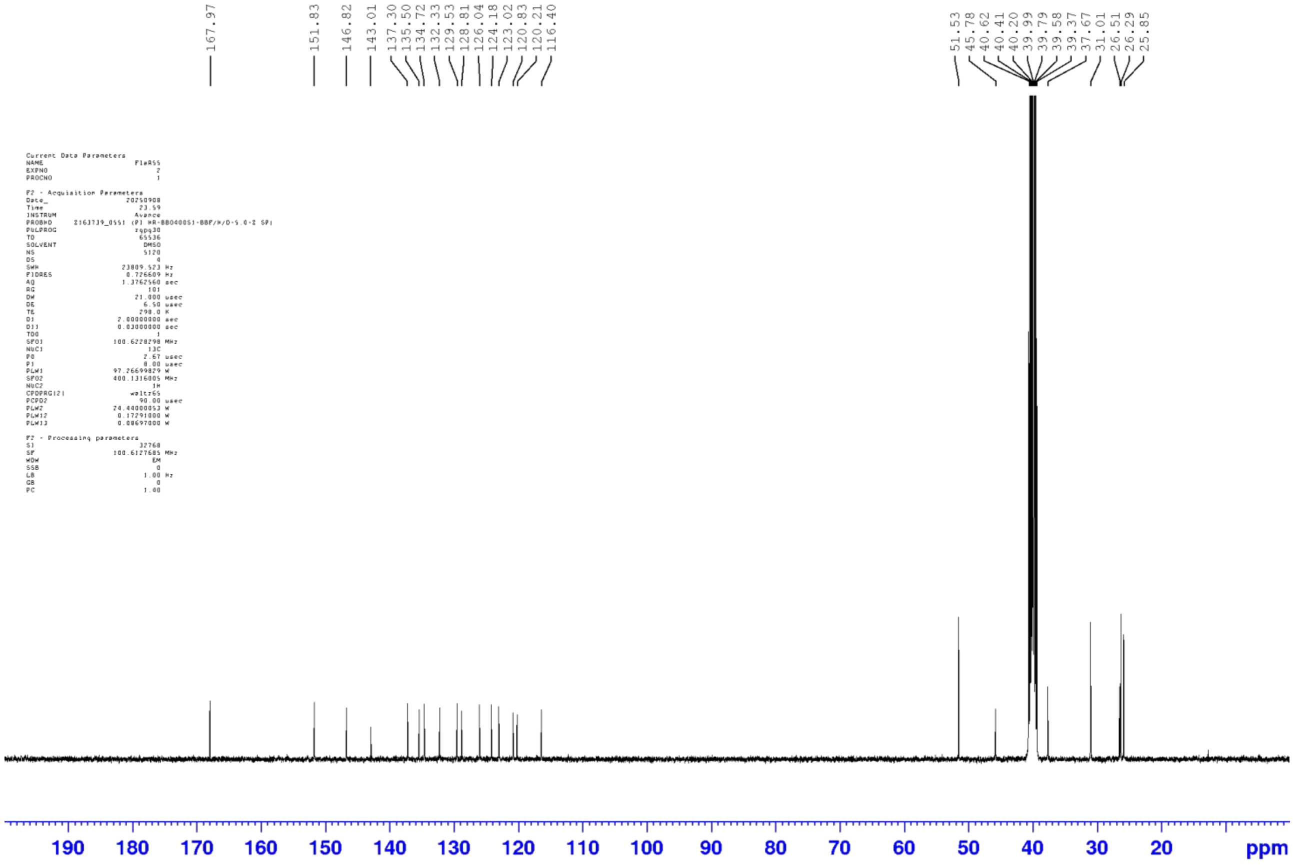

Figure S21. ^{13}CNMR spectrum of compound **9**.

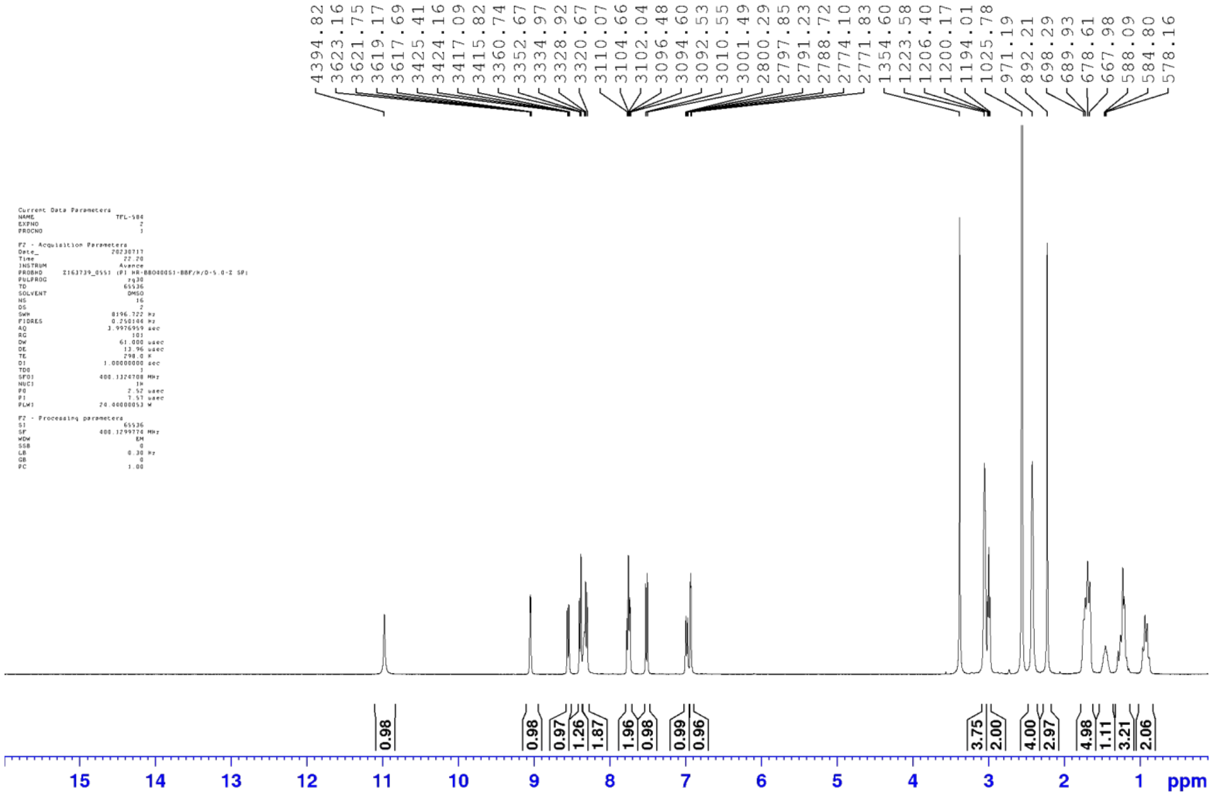

Figure S22. ^{1}HNMR spectrum of compound **10**.

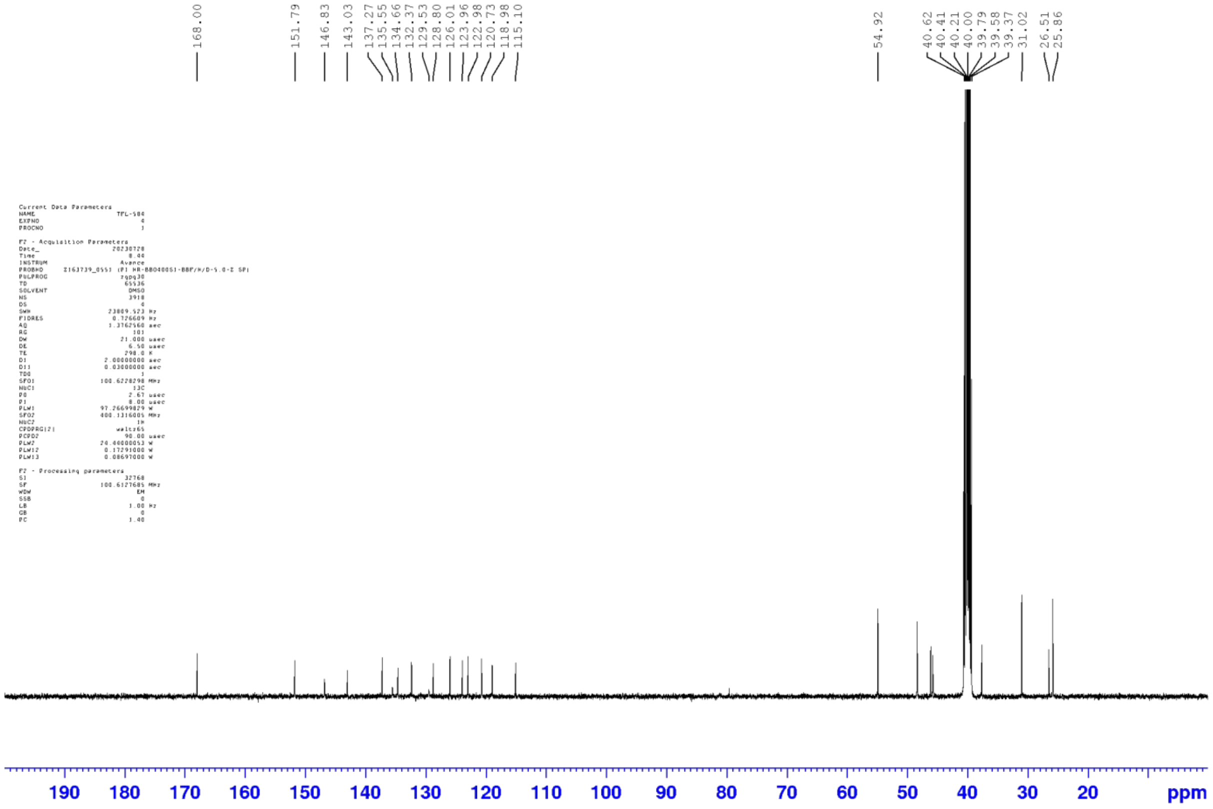

Figure S23. ^{13}CNMR spectrum of compound **10**.

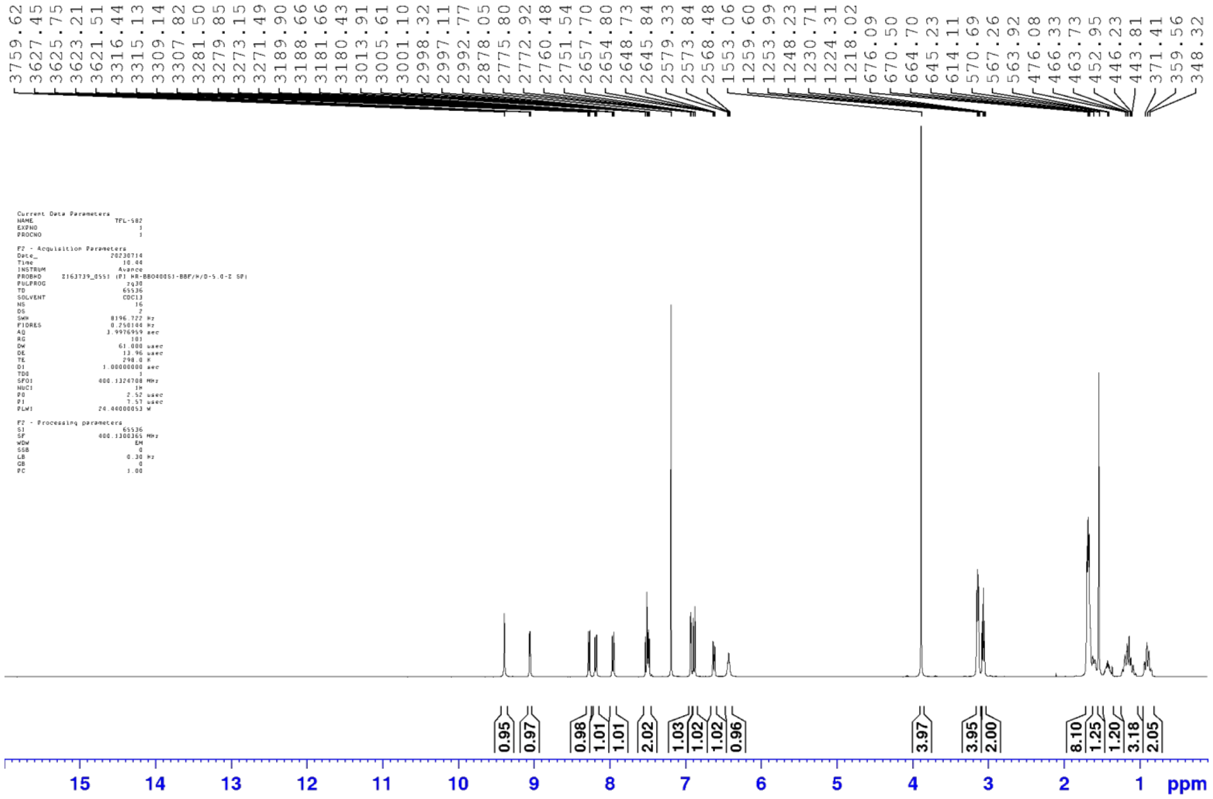

Figure S24. ^{1}HNMR spectrum of compound **11**.

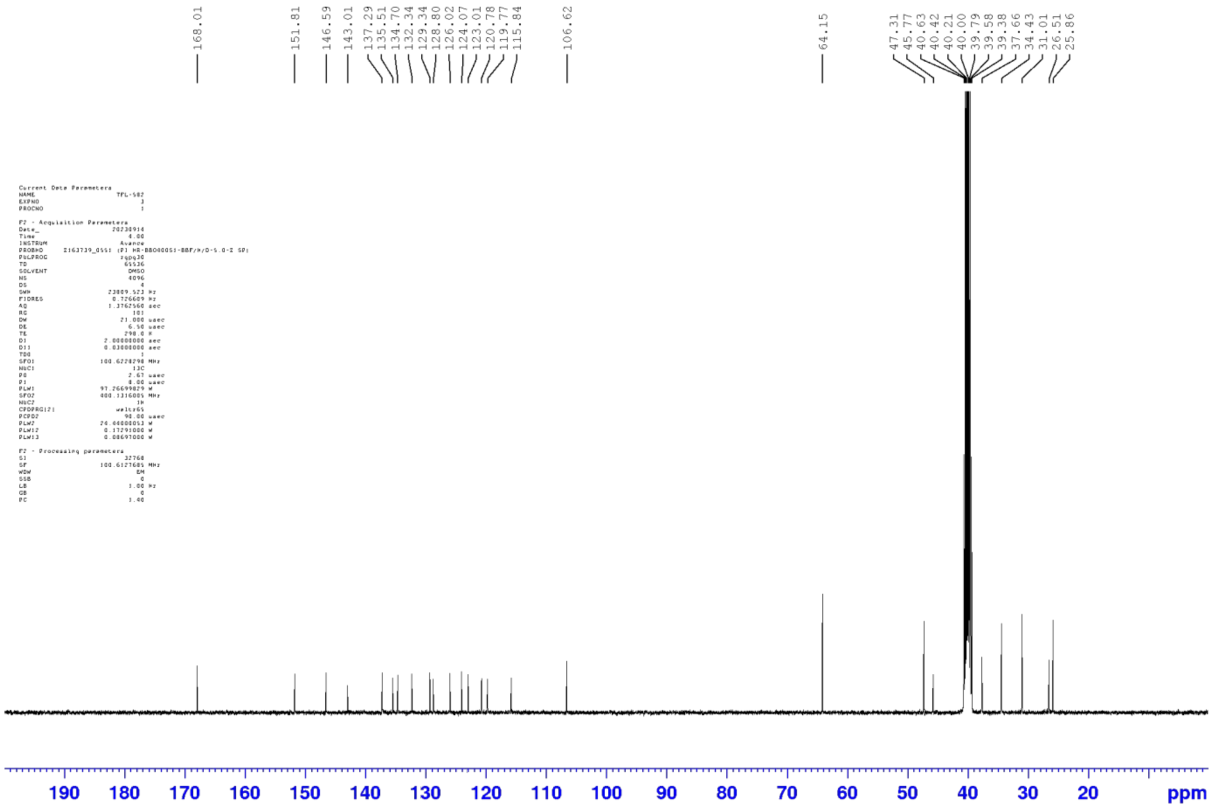

Figure S25. ^{13}CNMR spectrum of compound **11**.

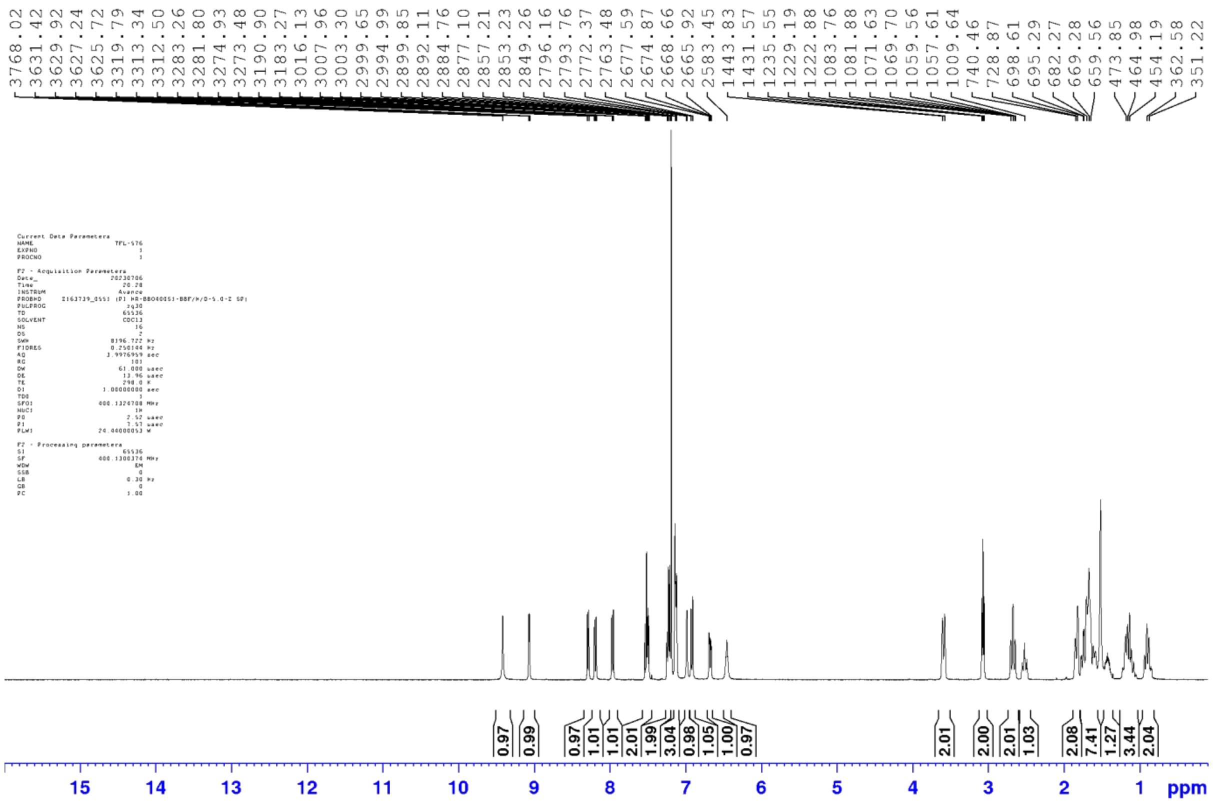

Figure S26. ^{1}HNMR spectrum of compound **12**.

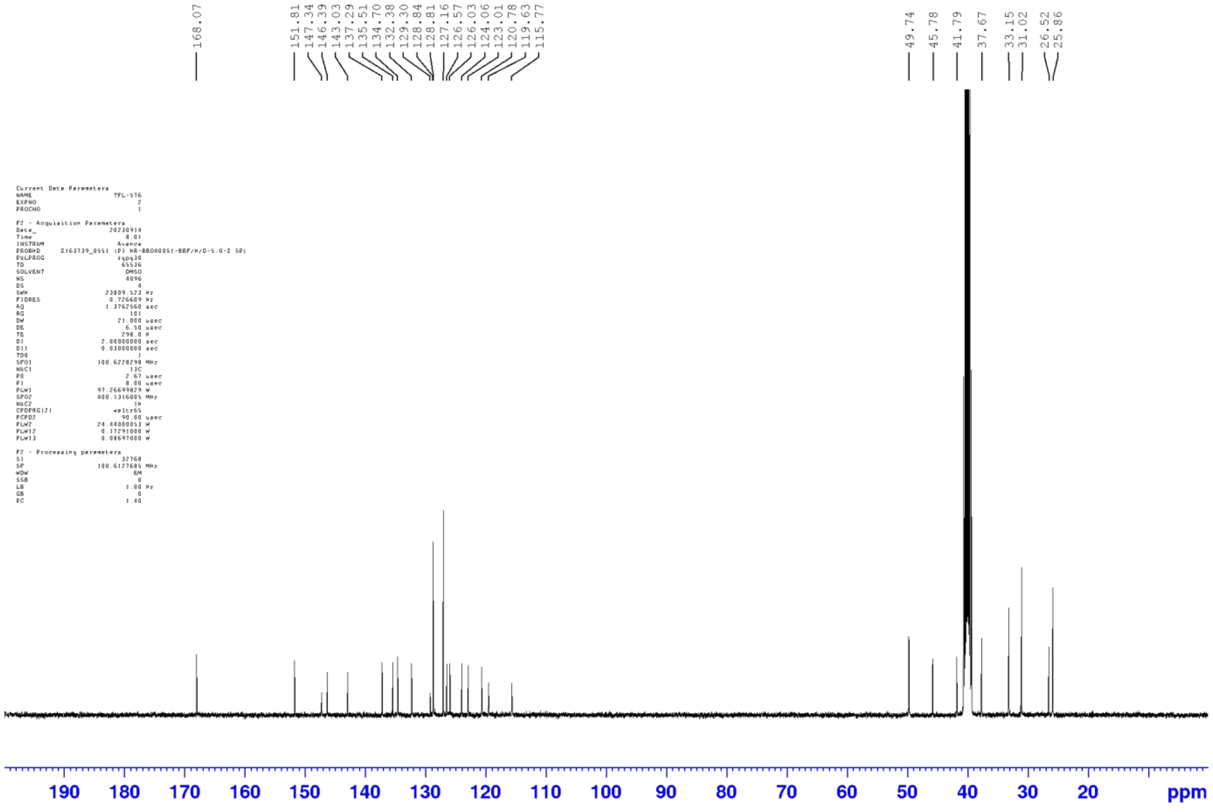

Figure S27. ^{13}CNMR spectrum of compound **12**.

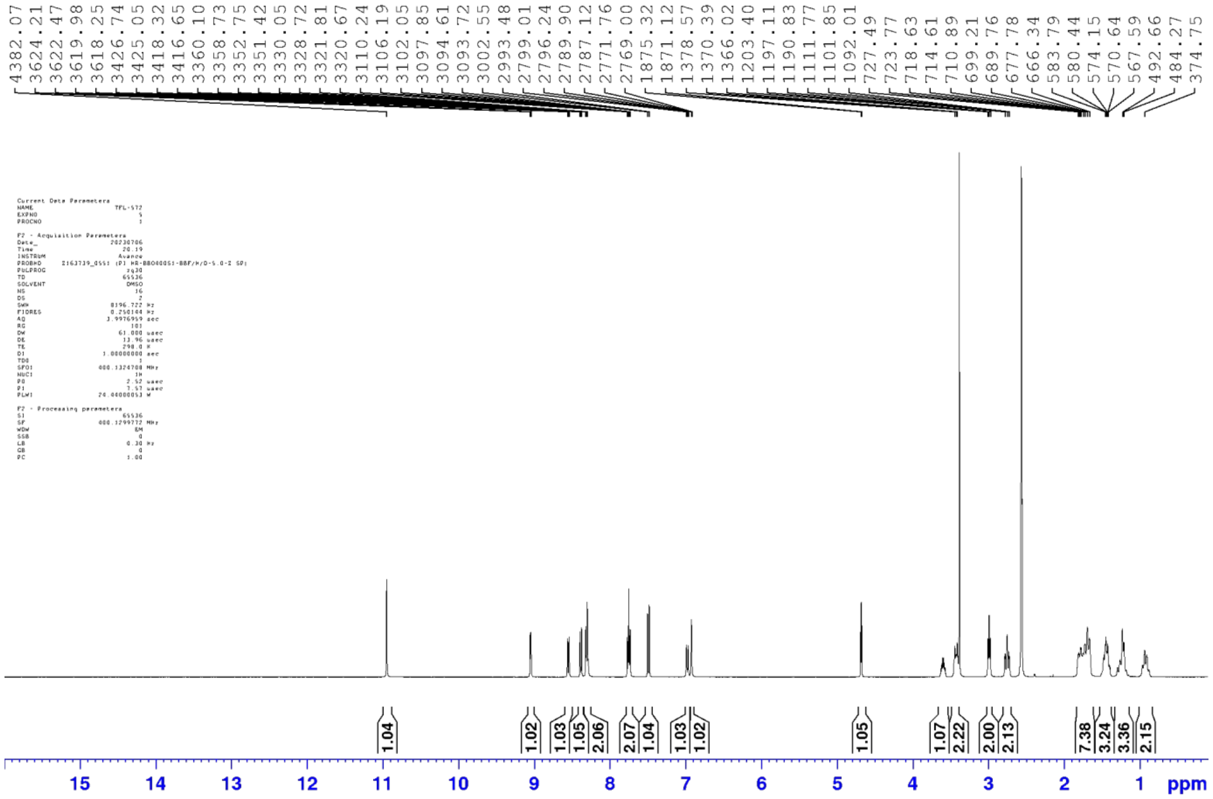

Figure S28. ^{1}HNMR spectrum of compound **13**.

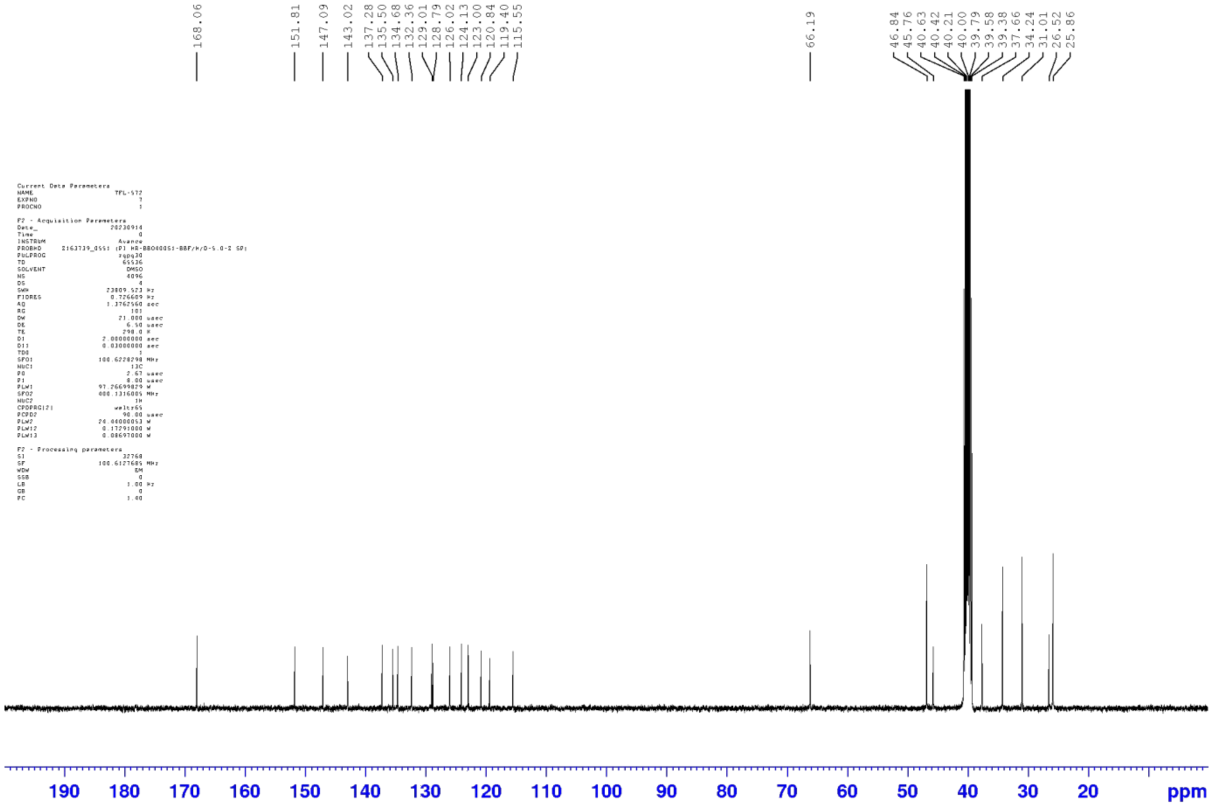

Figure S29. ^{13}CNMR spectrum of compound **13**.

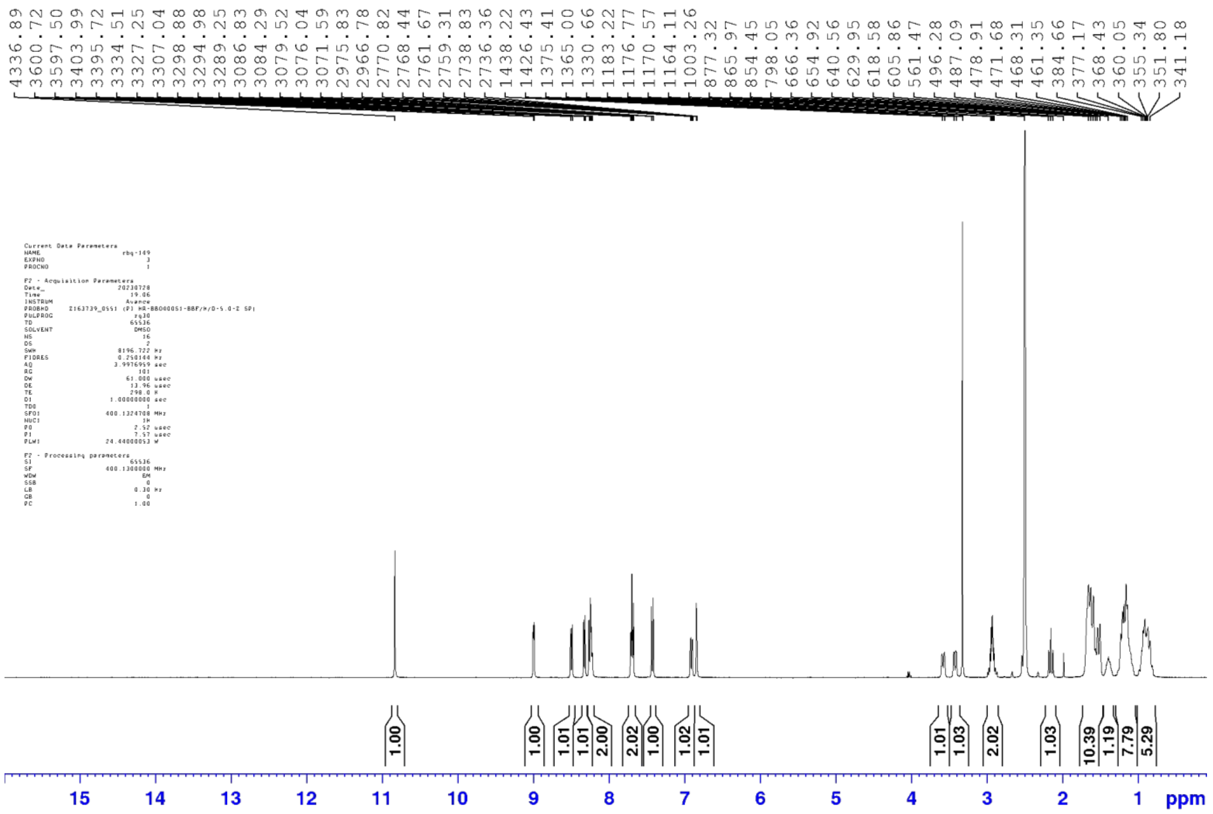

Figure S30. ^{1}HNMR spectrum of compound **14**.

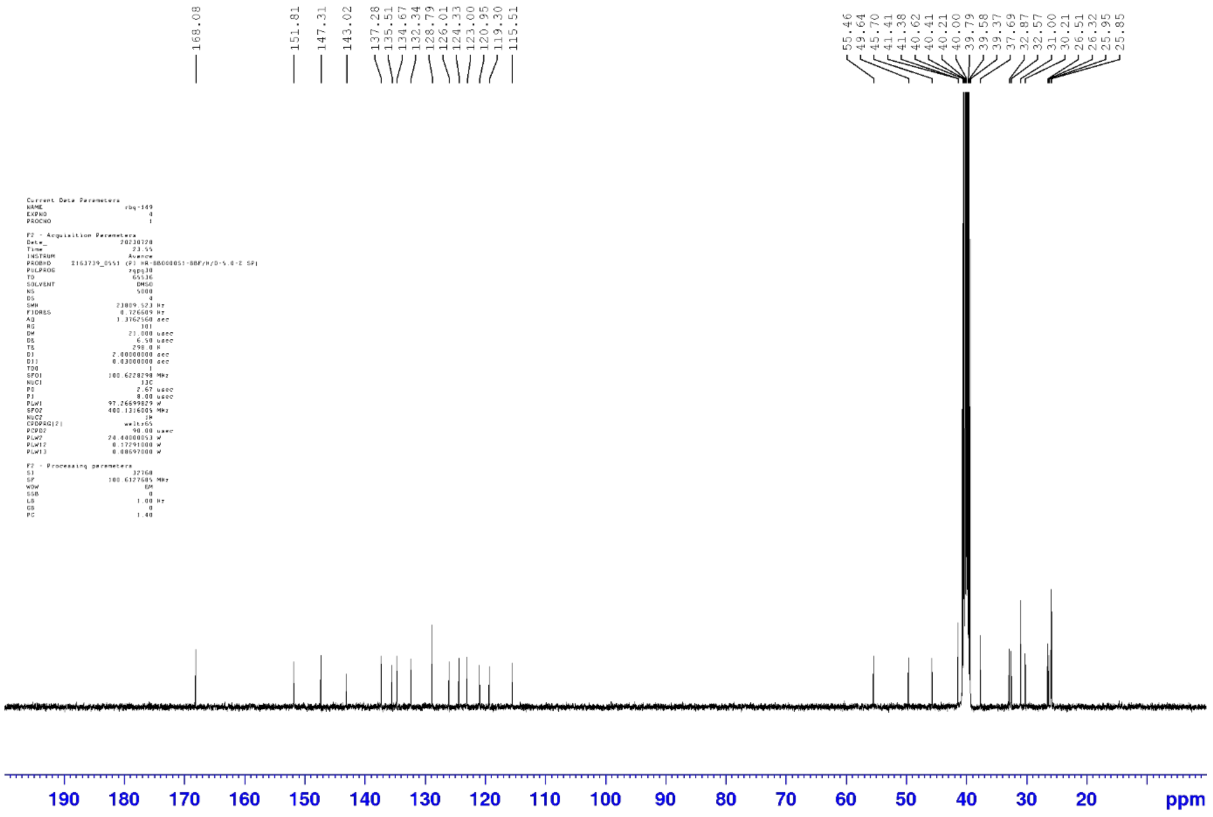

Figure S31. ^{13}CNMR spectrum of compound **14**.

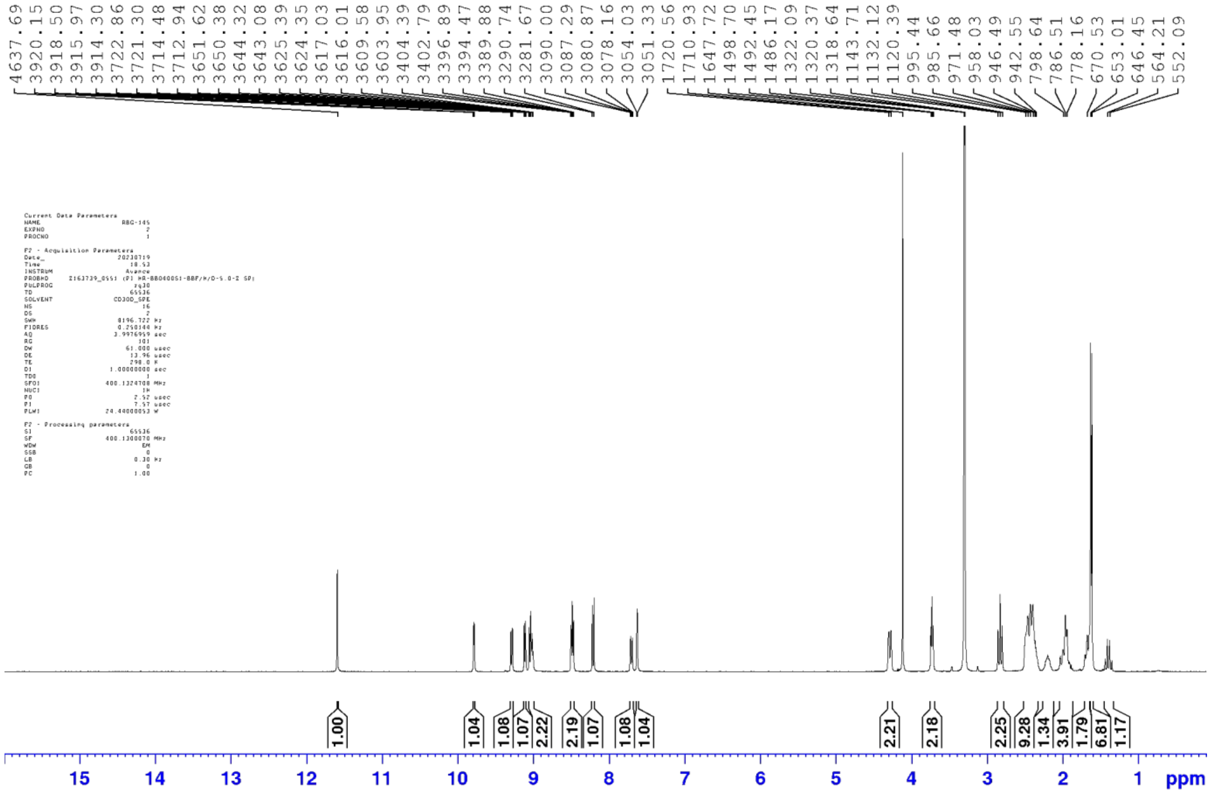

Figure S32. ^{1}HNMR spectrum of compound **15**.

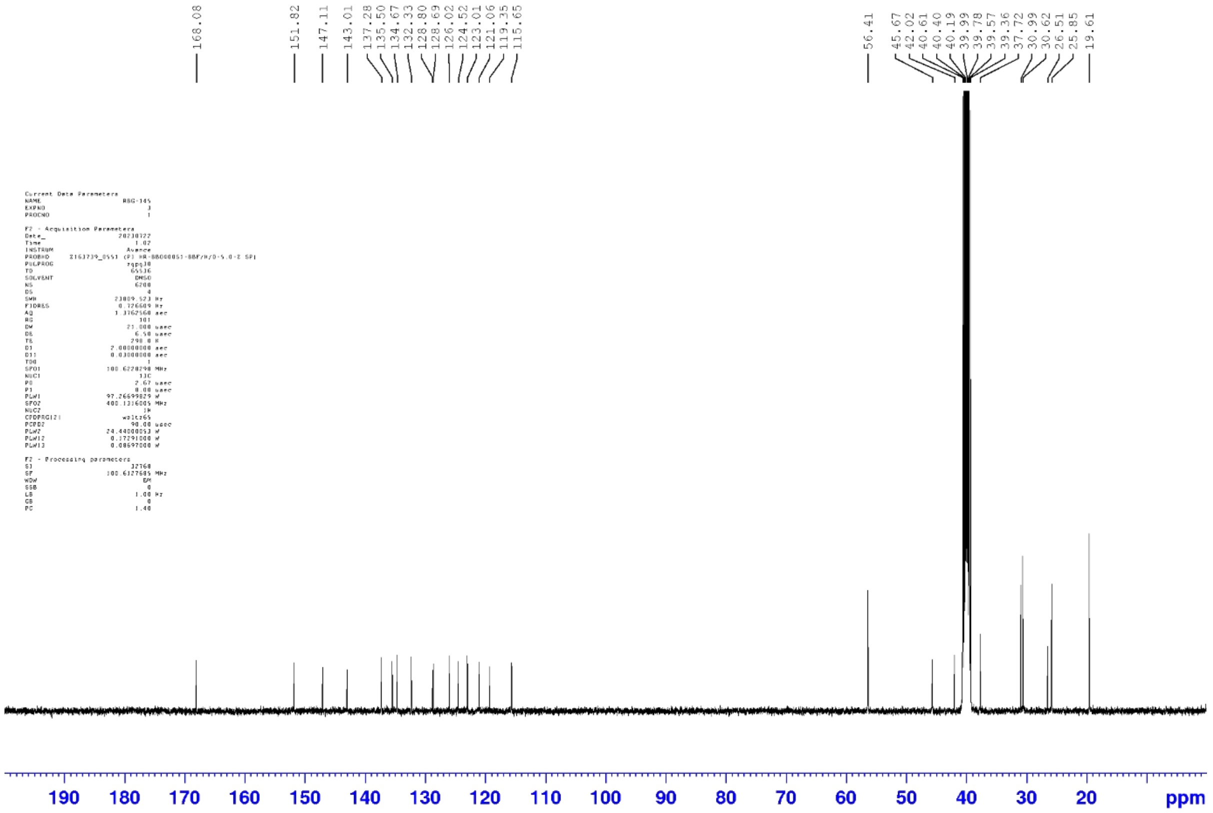

Figure S33. ^{13}CNMR spectrum of compound **15**.

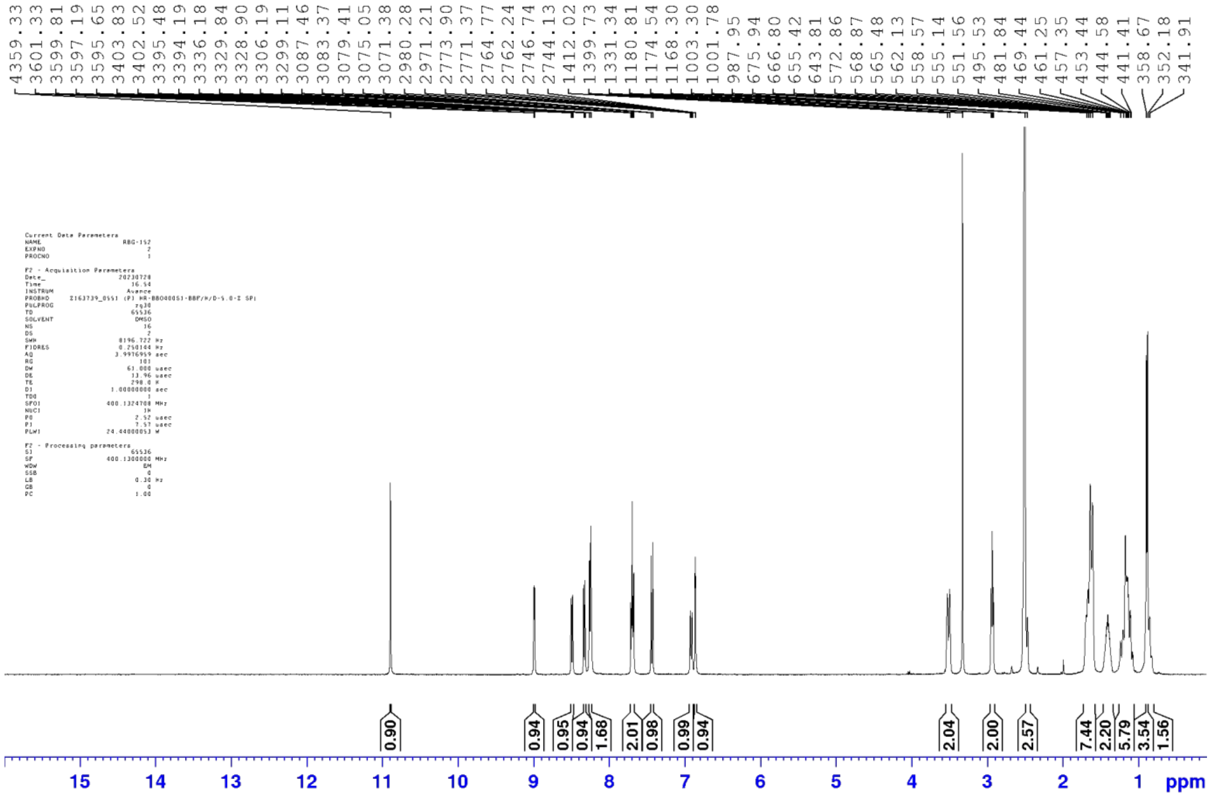

Figure S34. ^{1}HNMR spectrum of compound **16**.

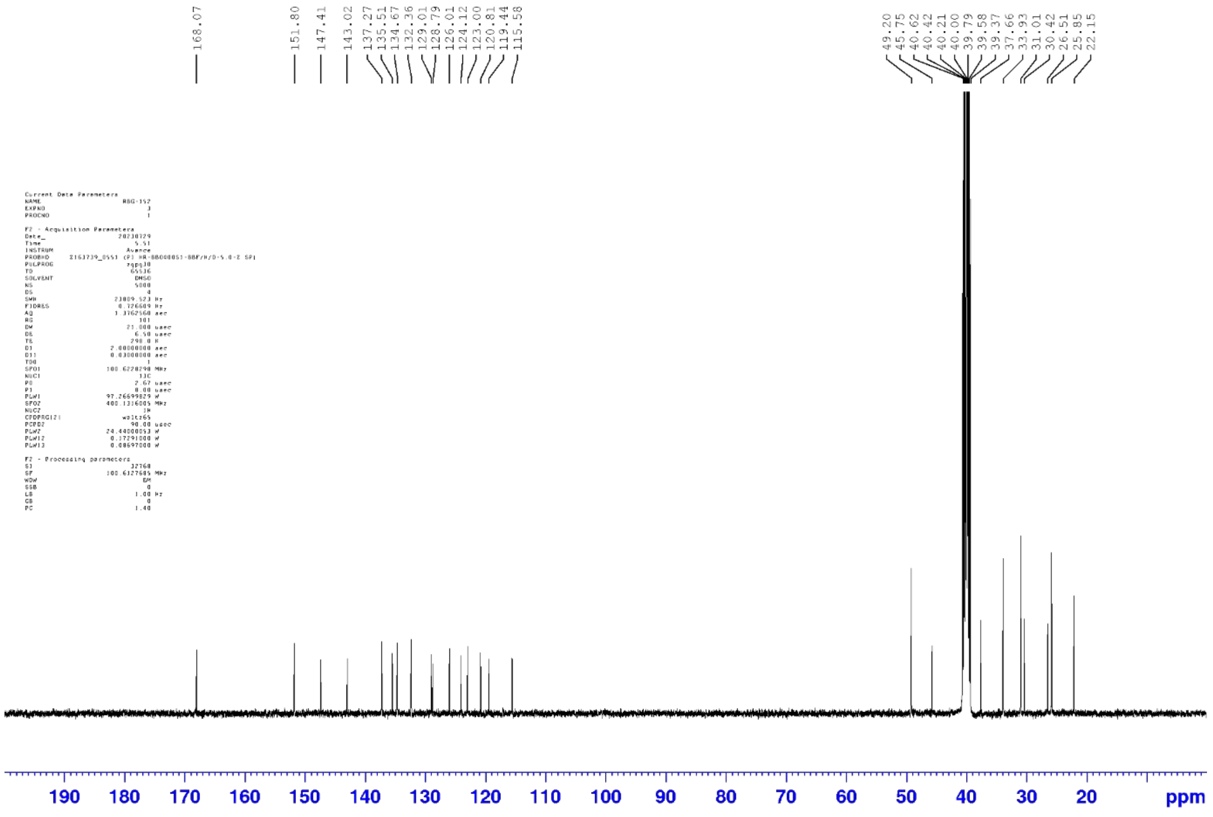

Figure S35. ^{13}CNMR spectrum of compound **16**.

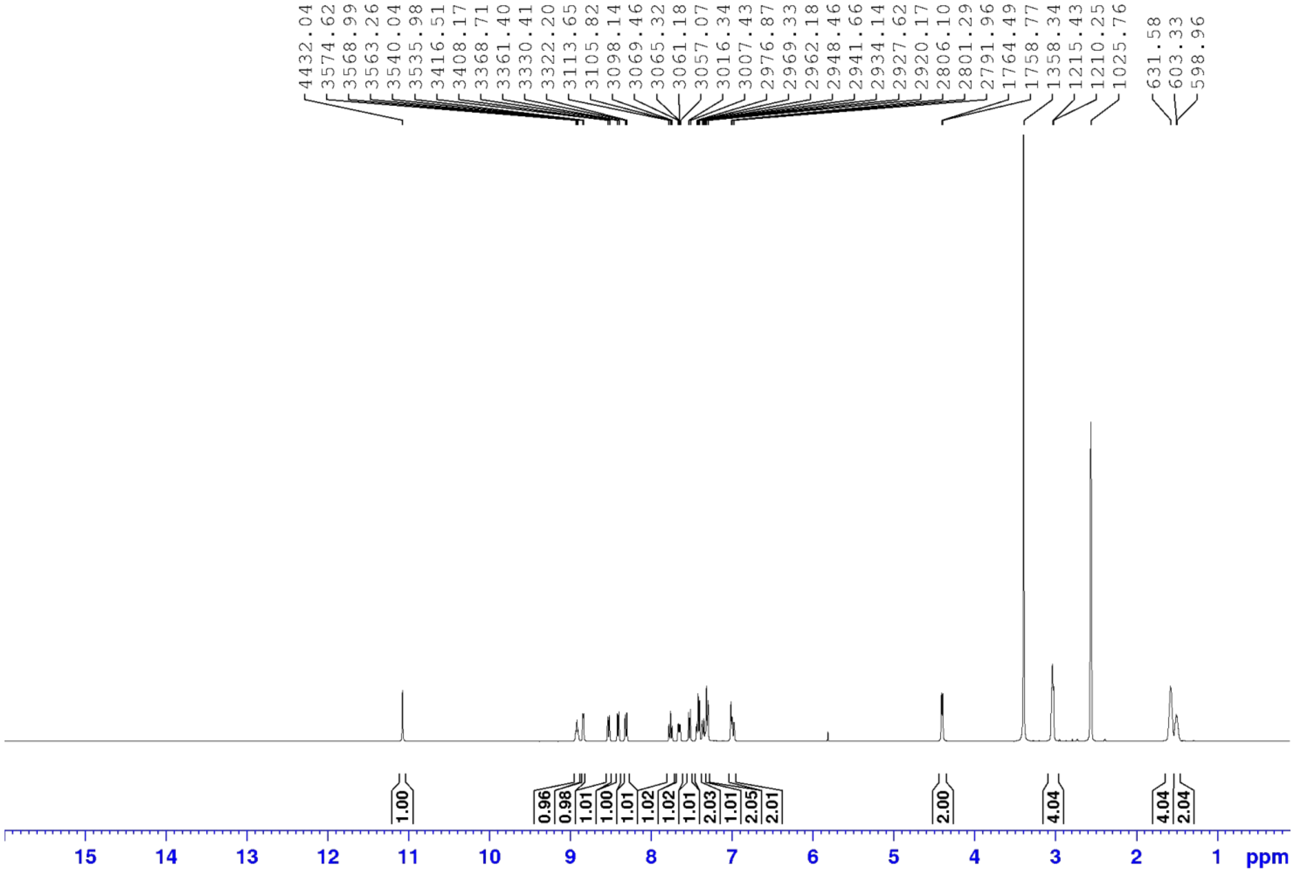

Figure S36. ^{1}HNMR spectrum of compound **17**.

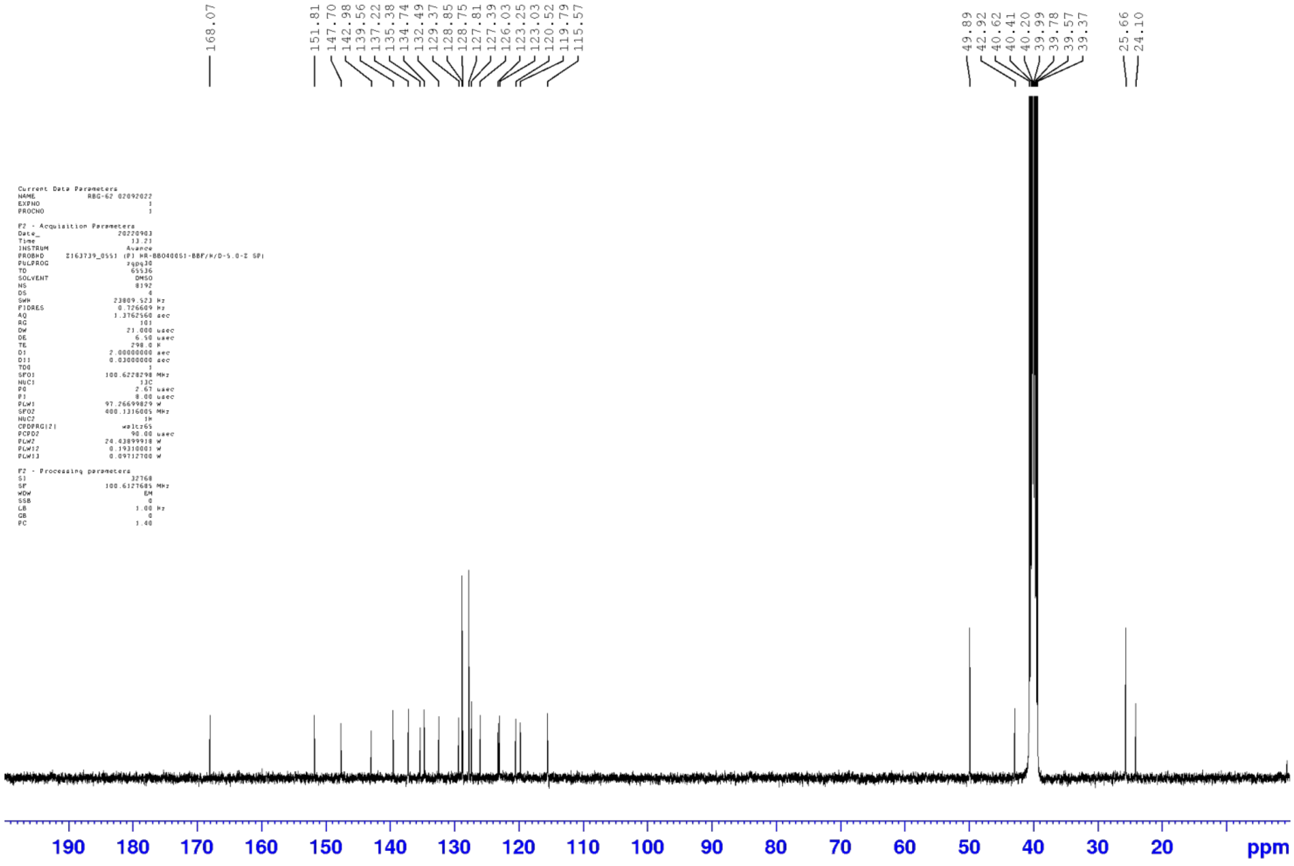

Figure S37. ^{13}CNMR spectrum of compound **17**.

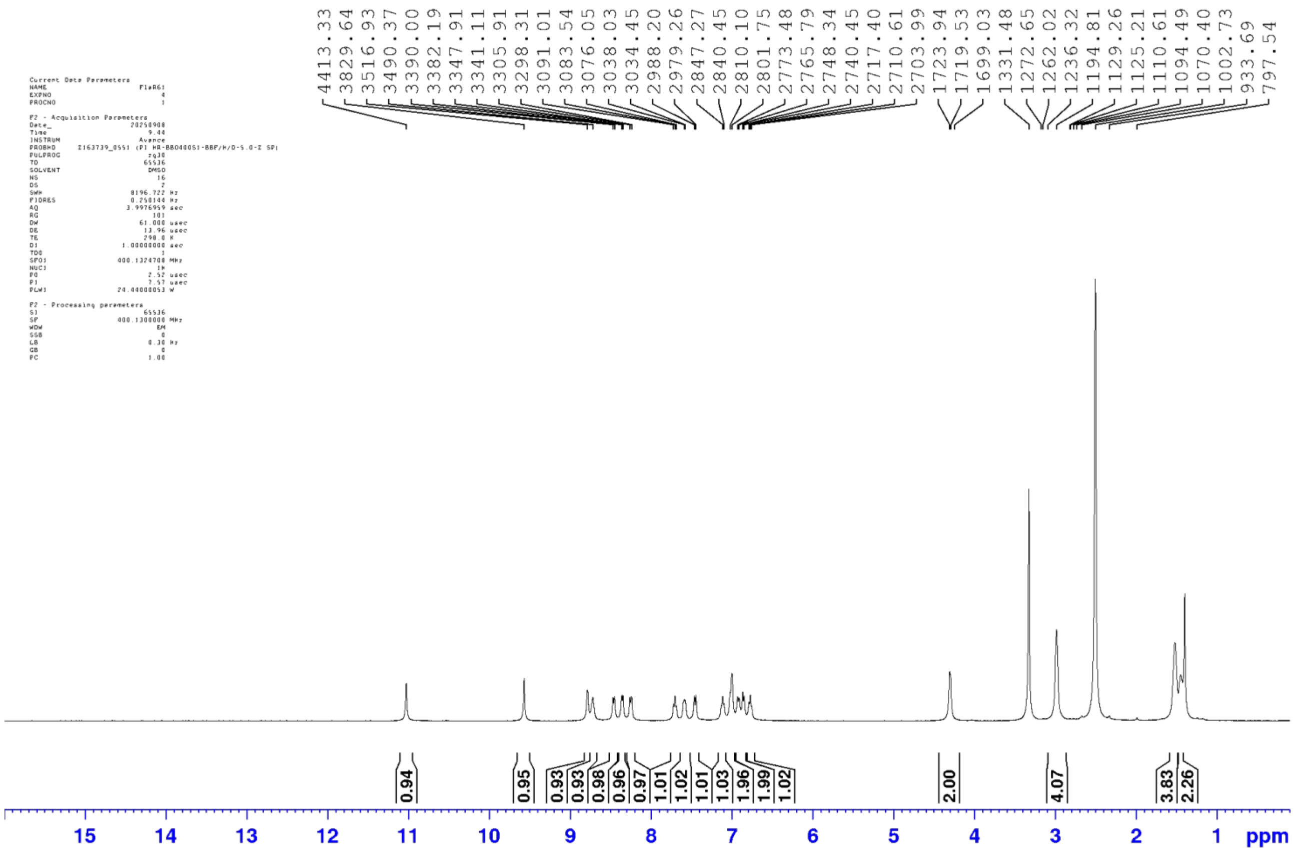

Figure S38. ^{1}HNMR spectra of compound **18**.

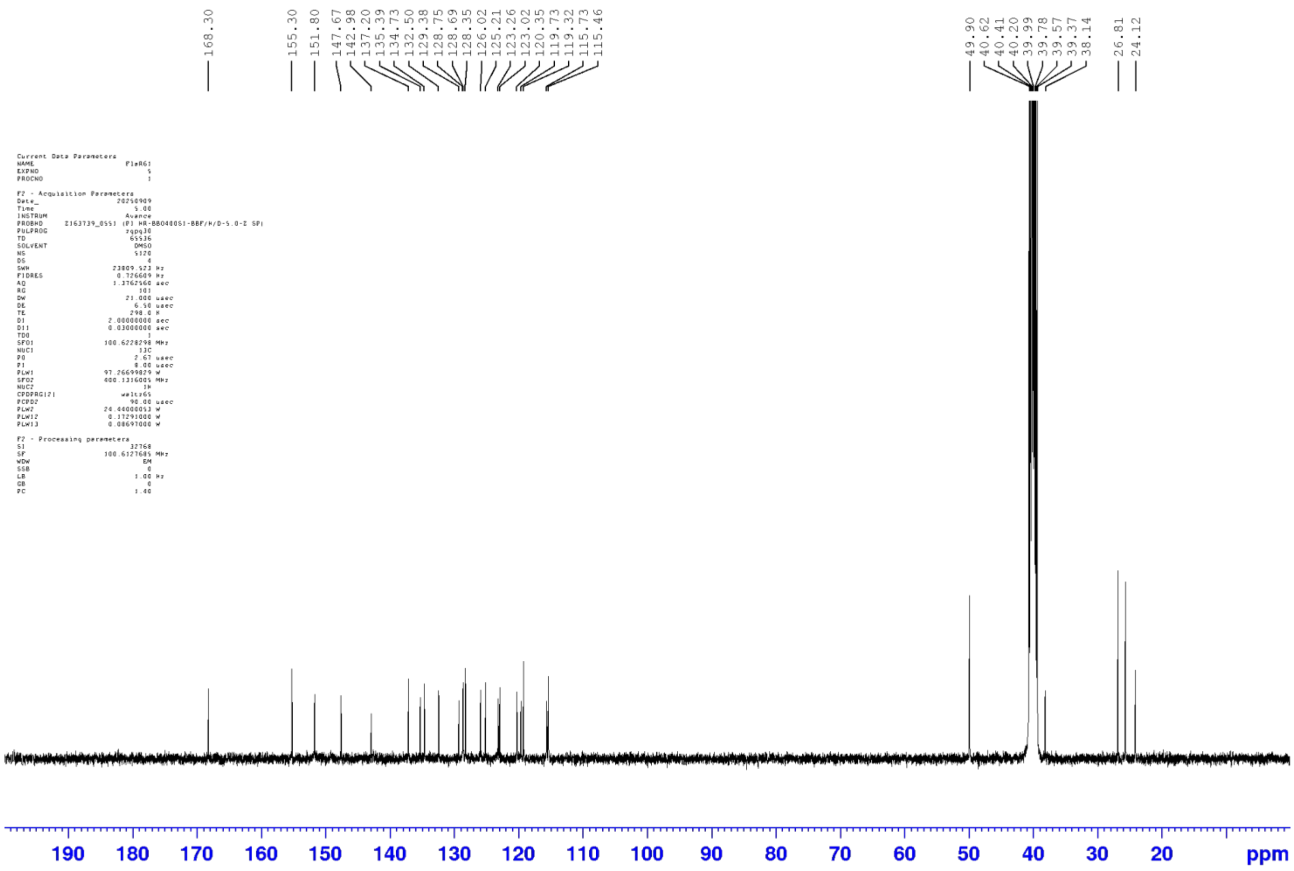

Figure S39. ^{13}CNMR spectrum of compound **18**.

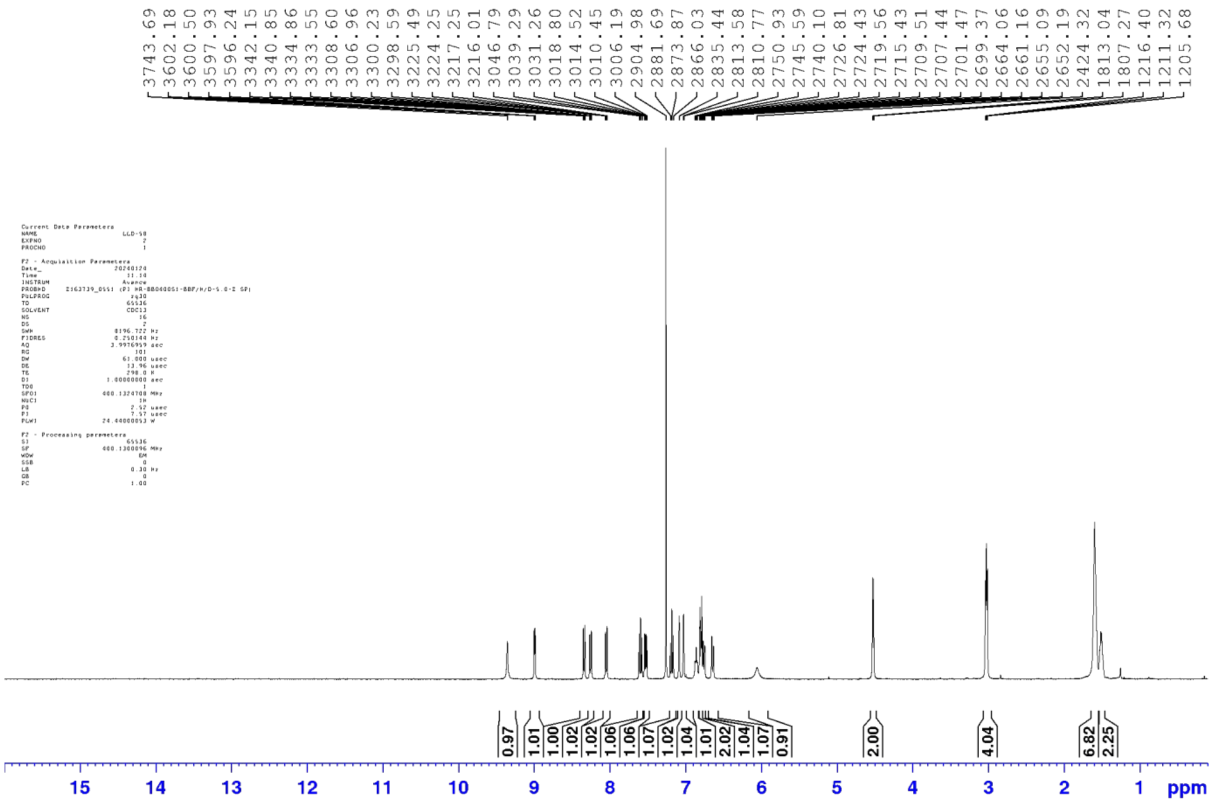

Figure S40. ^{1}HNMR spectrum of compound **19**.

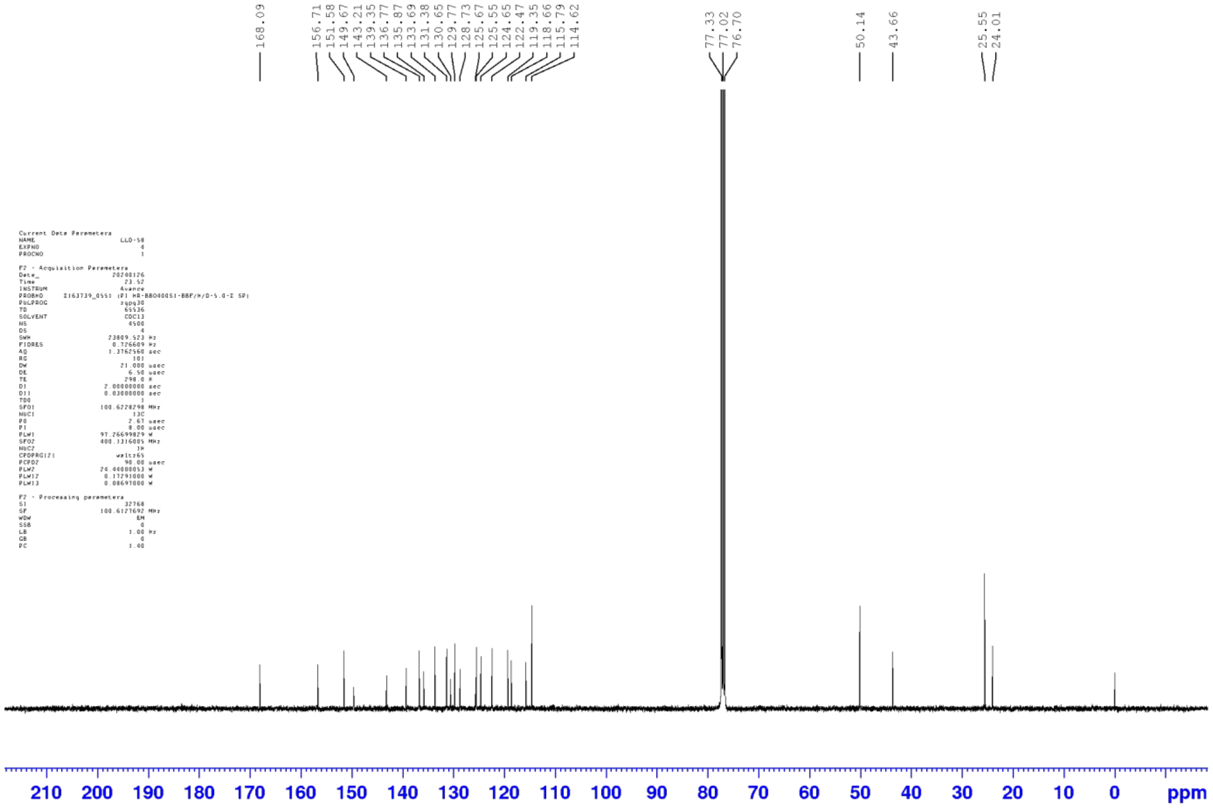

Figure S41. ^{13}CNMR spectrum of compound **19**.

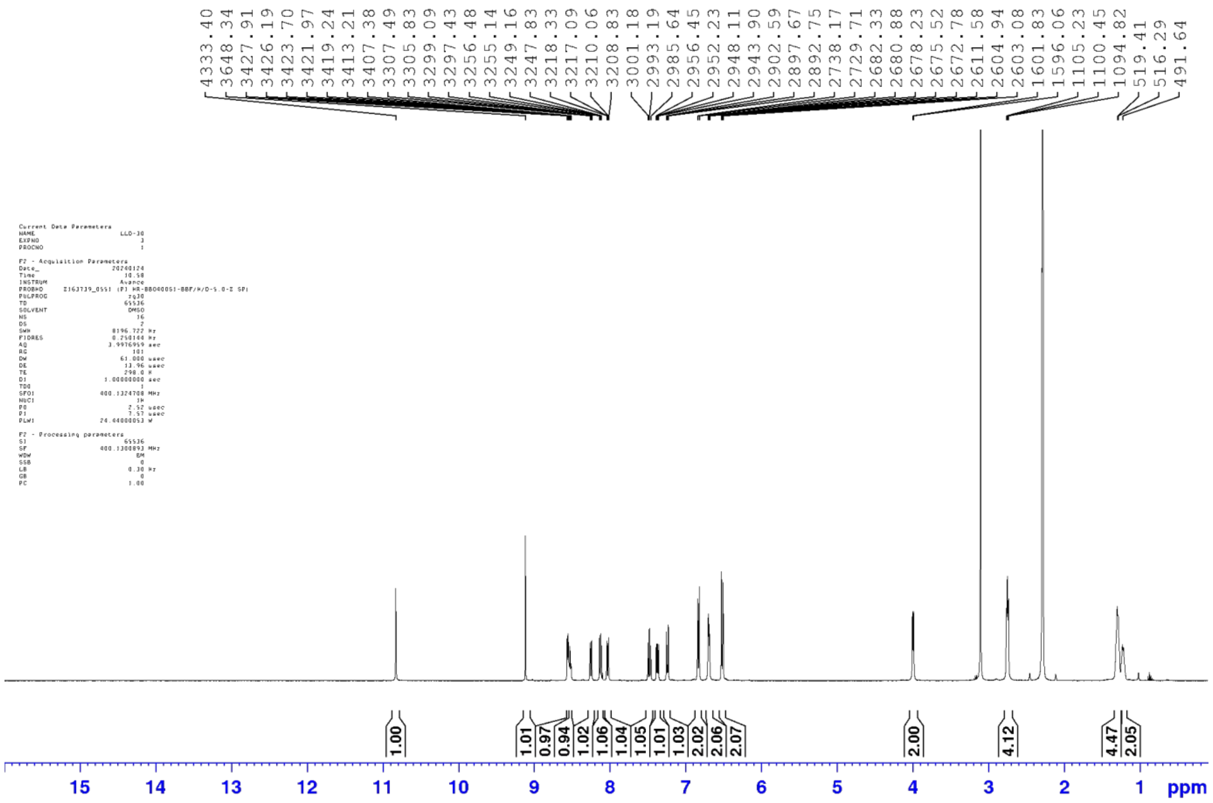

Figure S42. ^{1}HNMR spectrum of compound **20**.

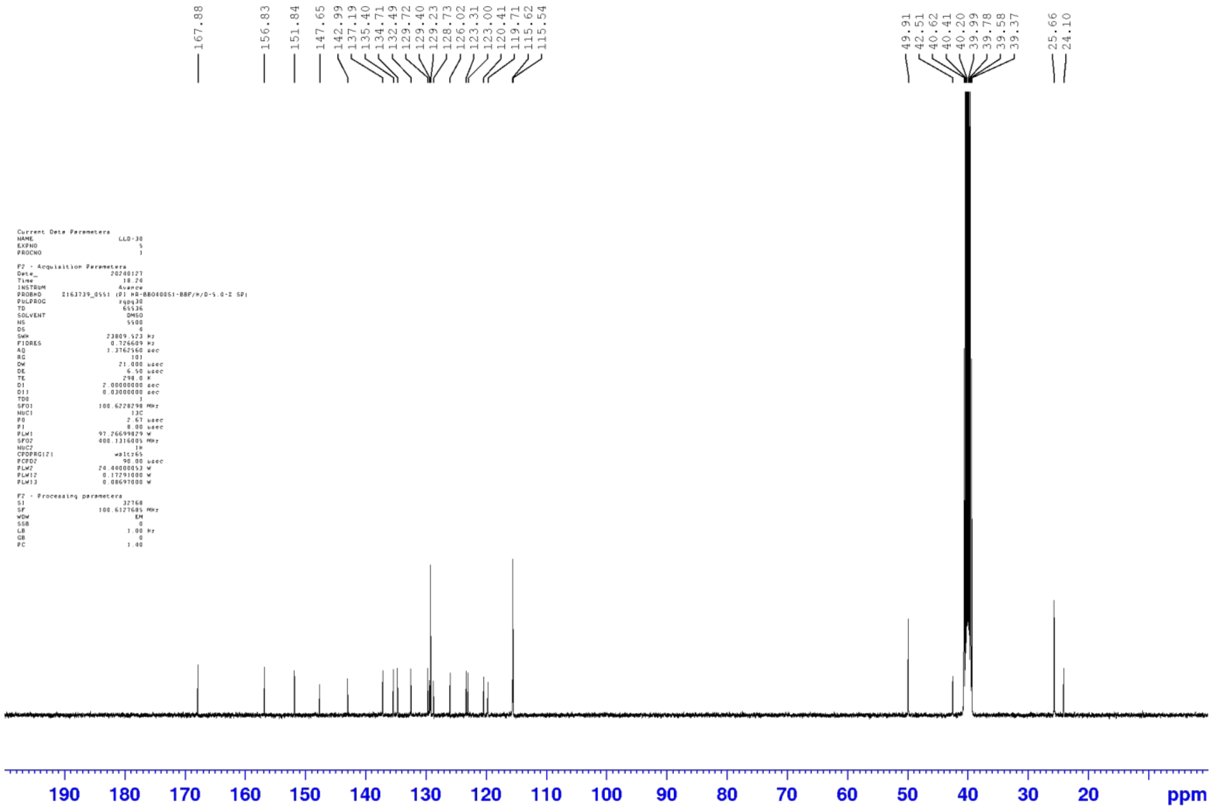

Figure S43. ^{13}CNMR spectrum of compound **20**.

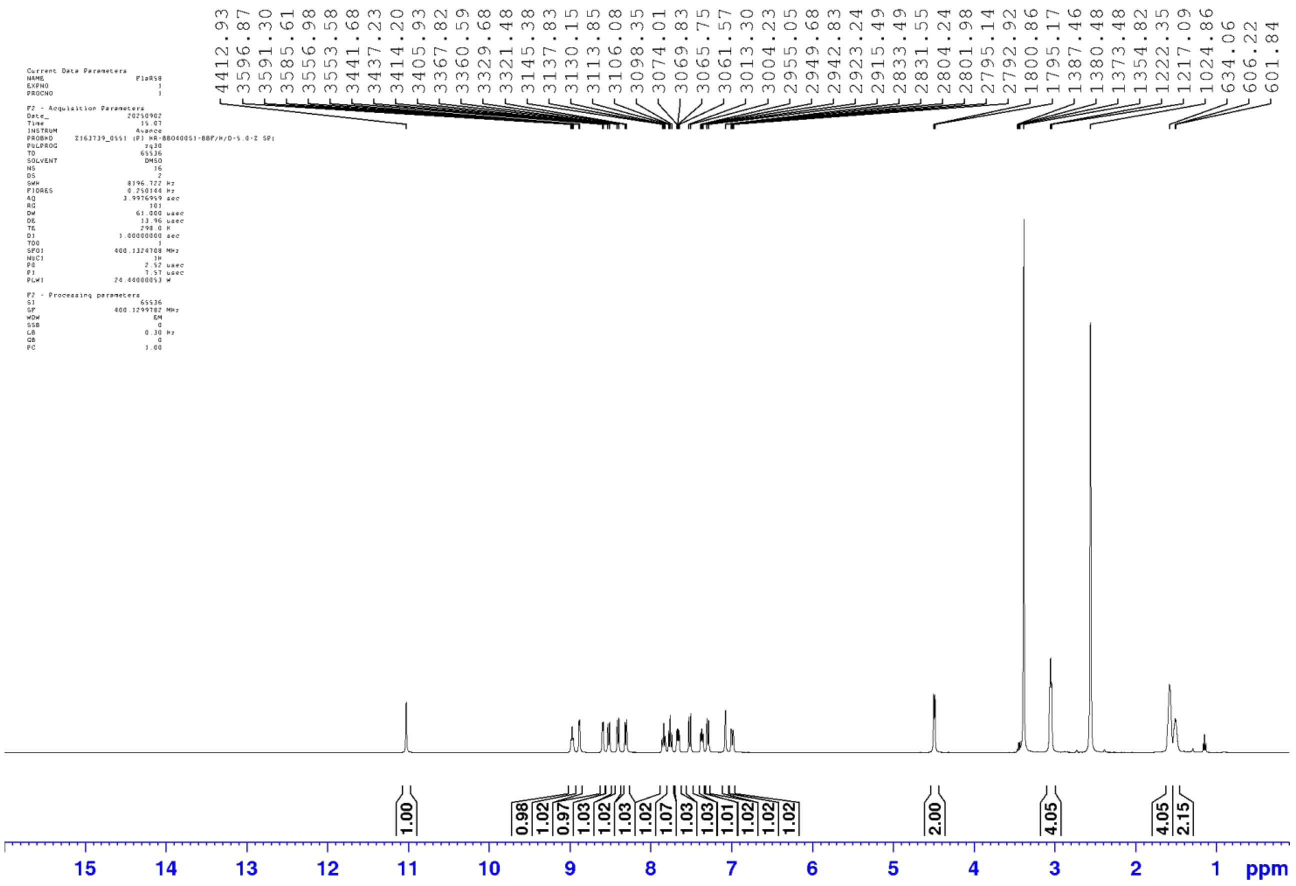

Figure S44. ¹HNMR spectrum of compound **21**.

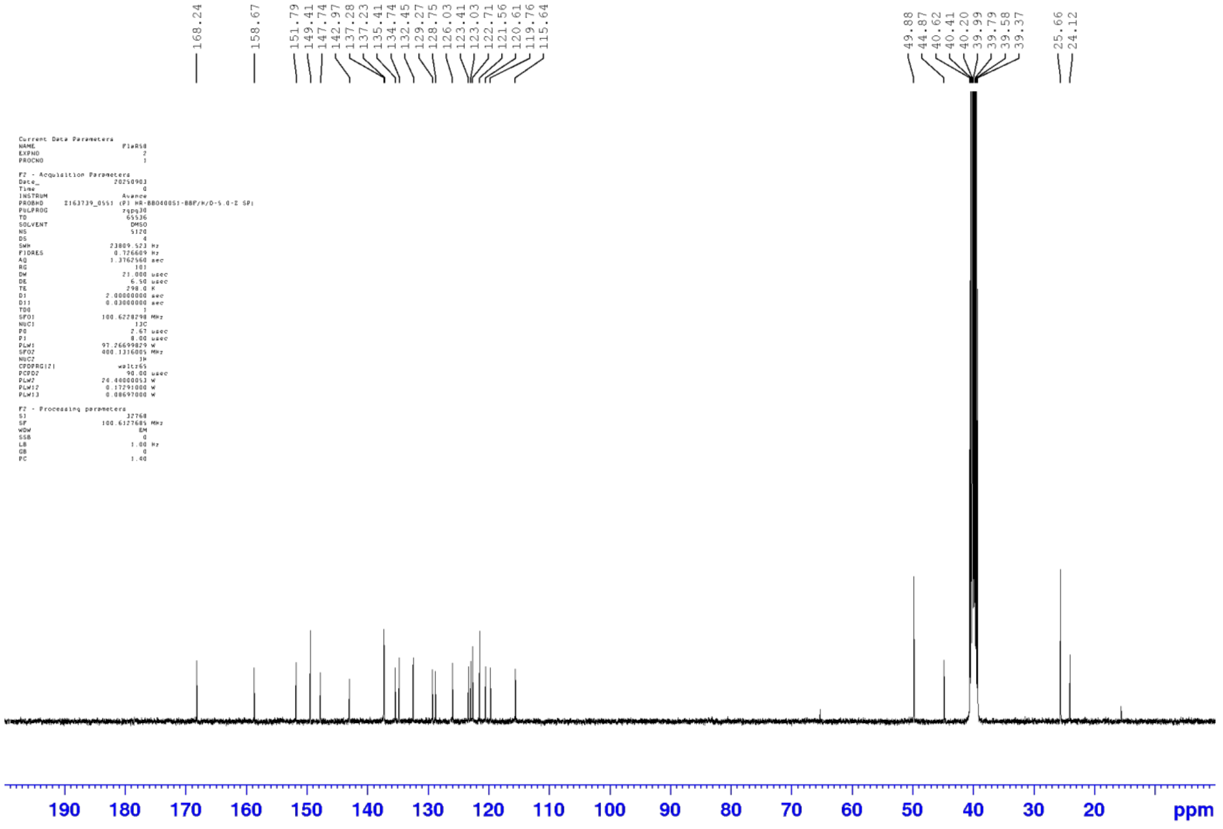

Figure S45. ¹³CNMR spectrum of compound **21**.

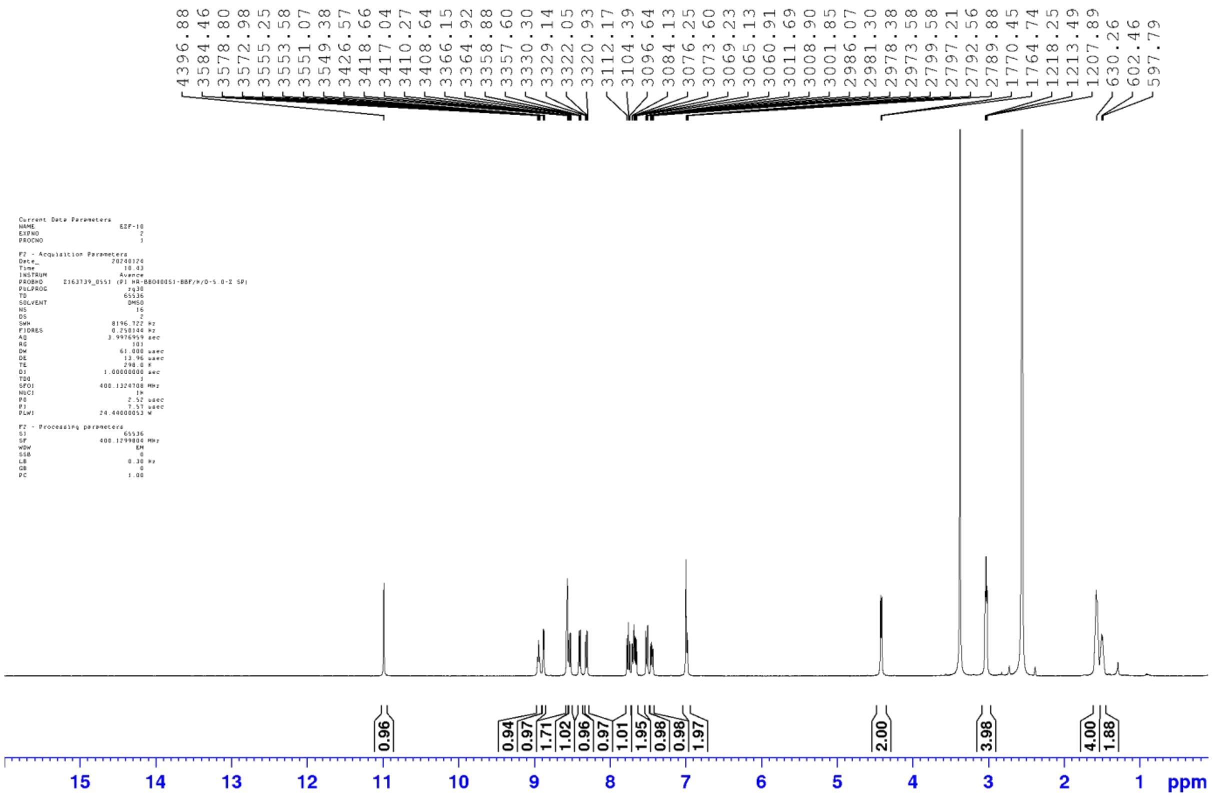

Figure S46. ^{1}HNMR spectrum of compound **22**.

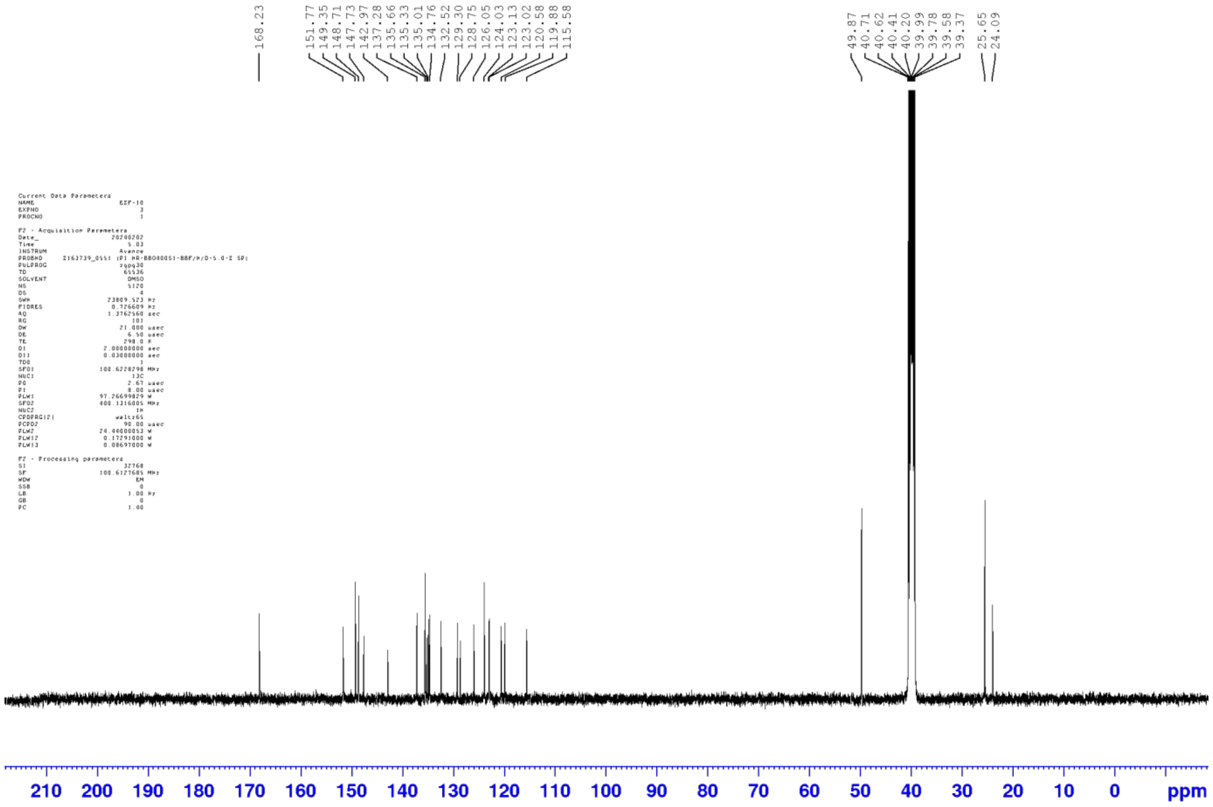

Figure S47. ^{13}CNMR spectrum of compound **22**.

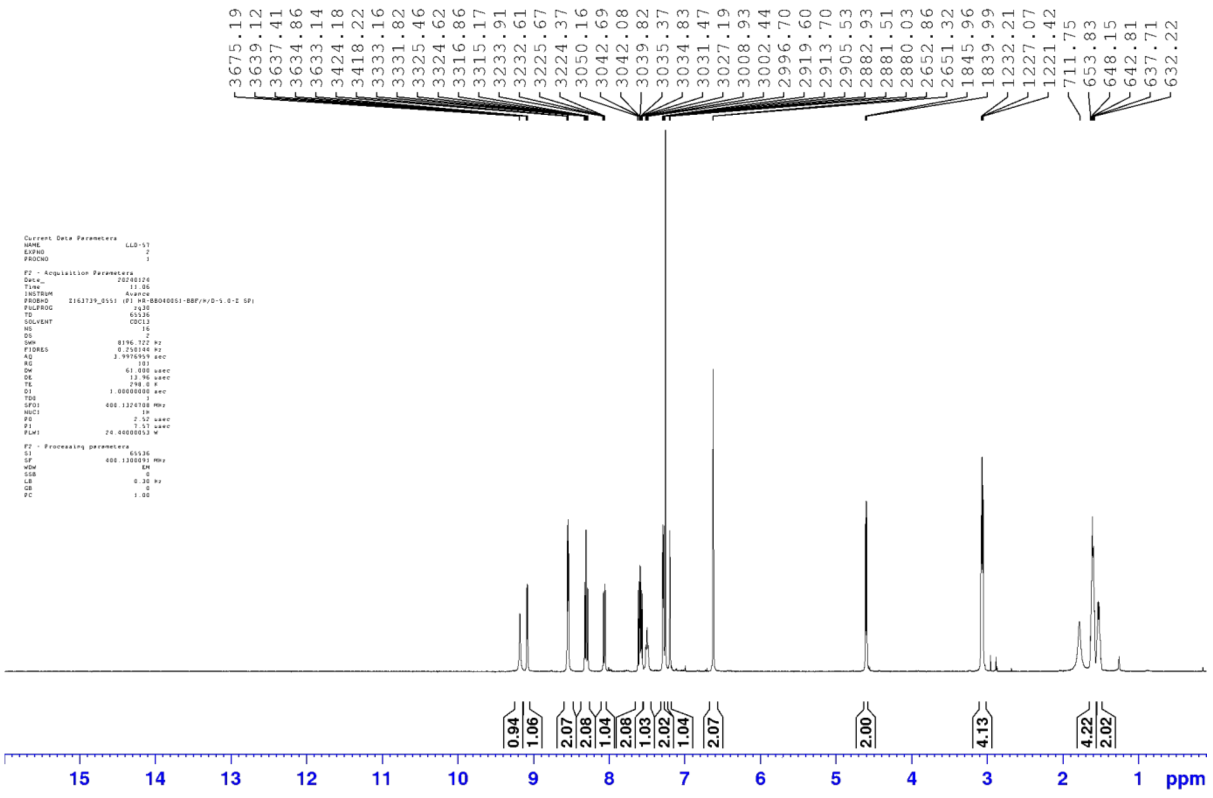

Figure S48. ^{1}HNMR spectrum of compound **23**.

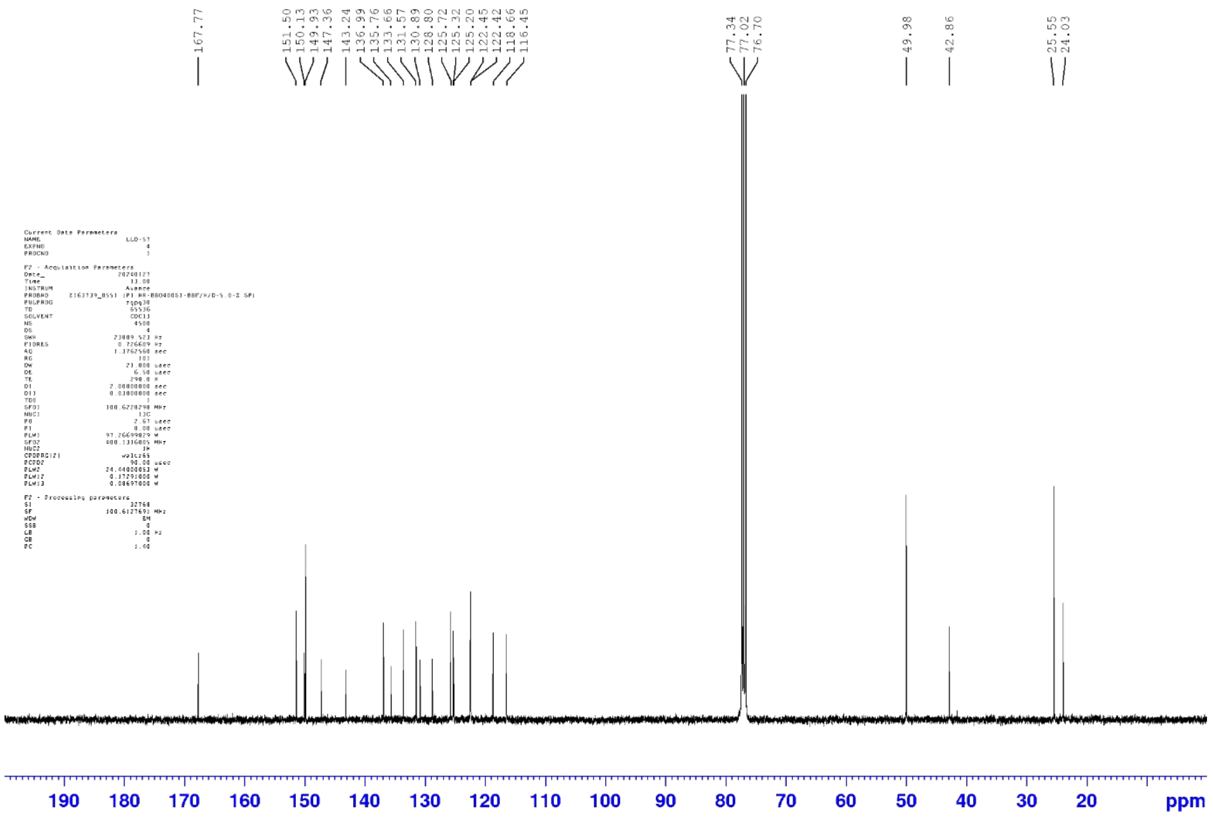

Figure S49. ^{13}CNMR spectrum of compound **23**.

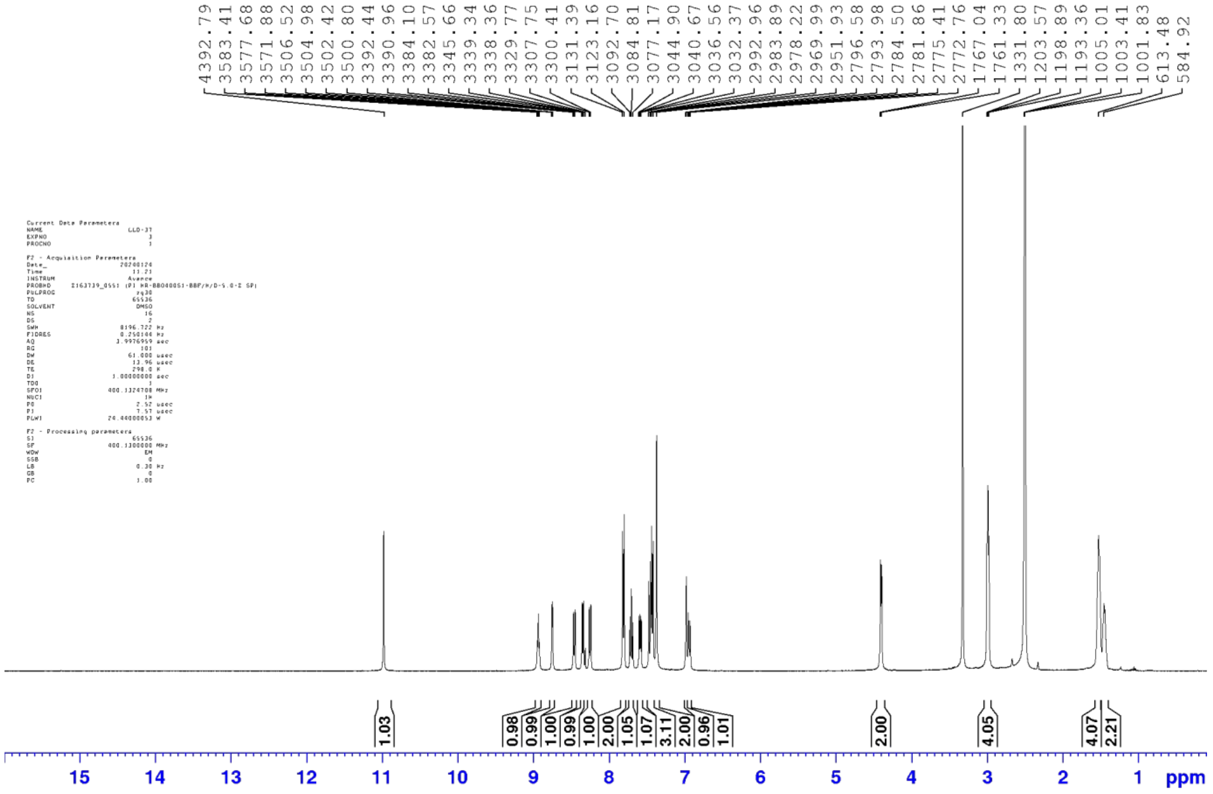

Figure S50. ^{1}HNMR spectrum of compound **24**.

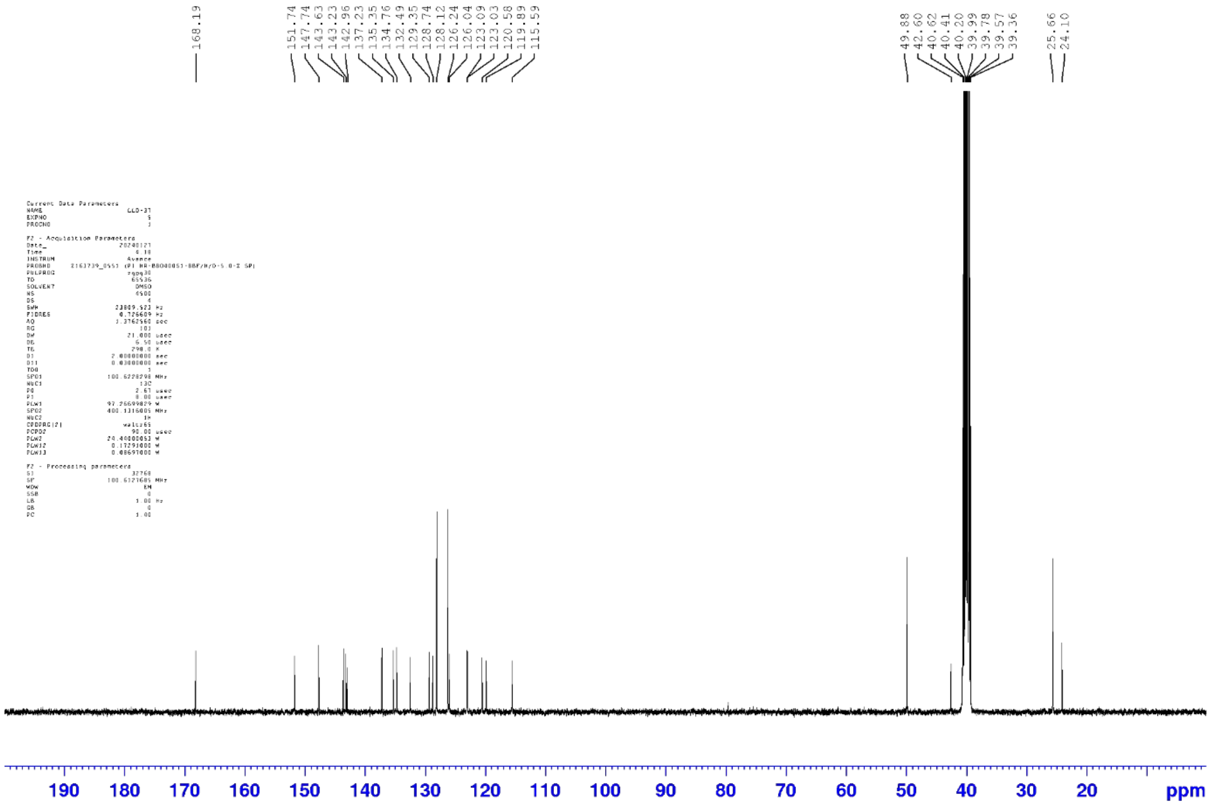

Figure S51. ^{13}CNMR spectrum of compound **24**.

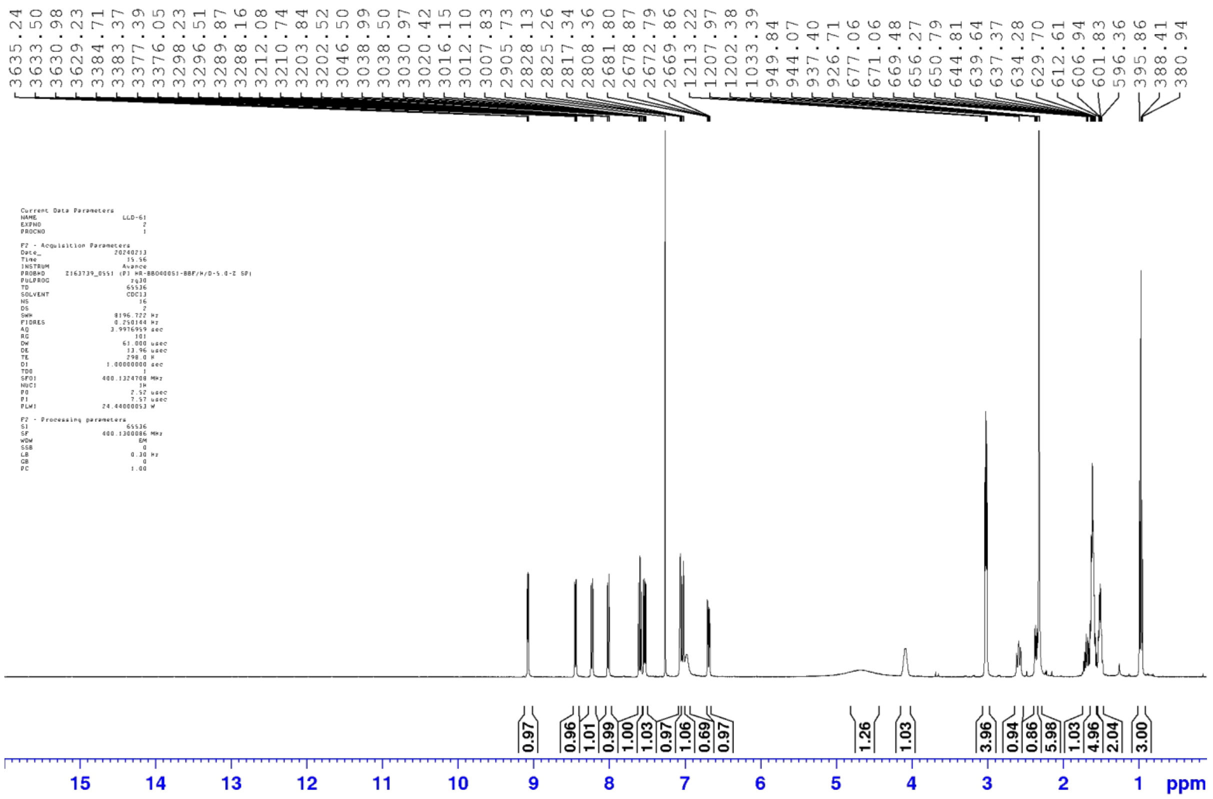

Figure S52. ^{1}HNMR spectrum of compound **25**.

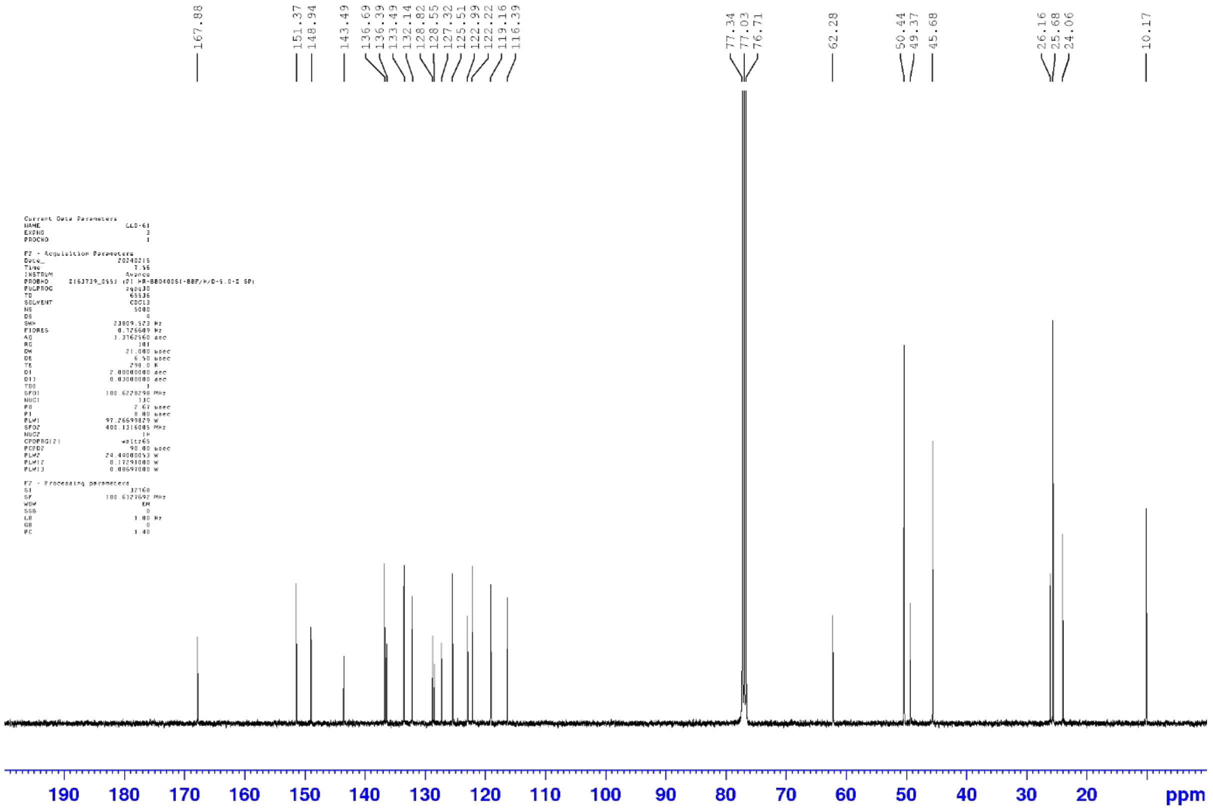

Figure S53. ^{13}CNMR spectrum of compound **25**.

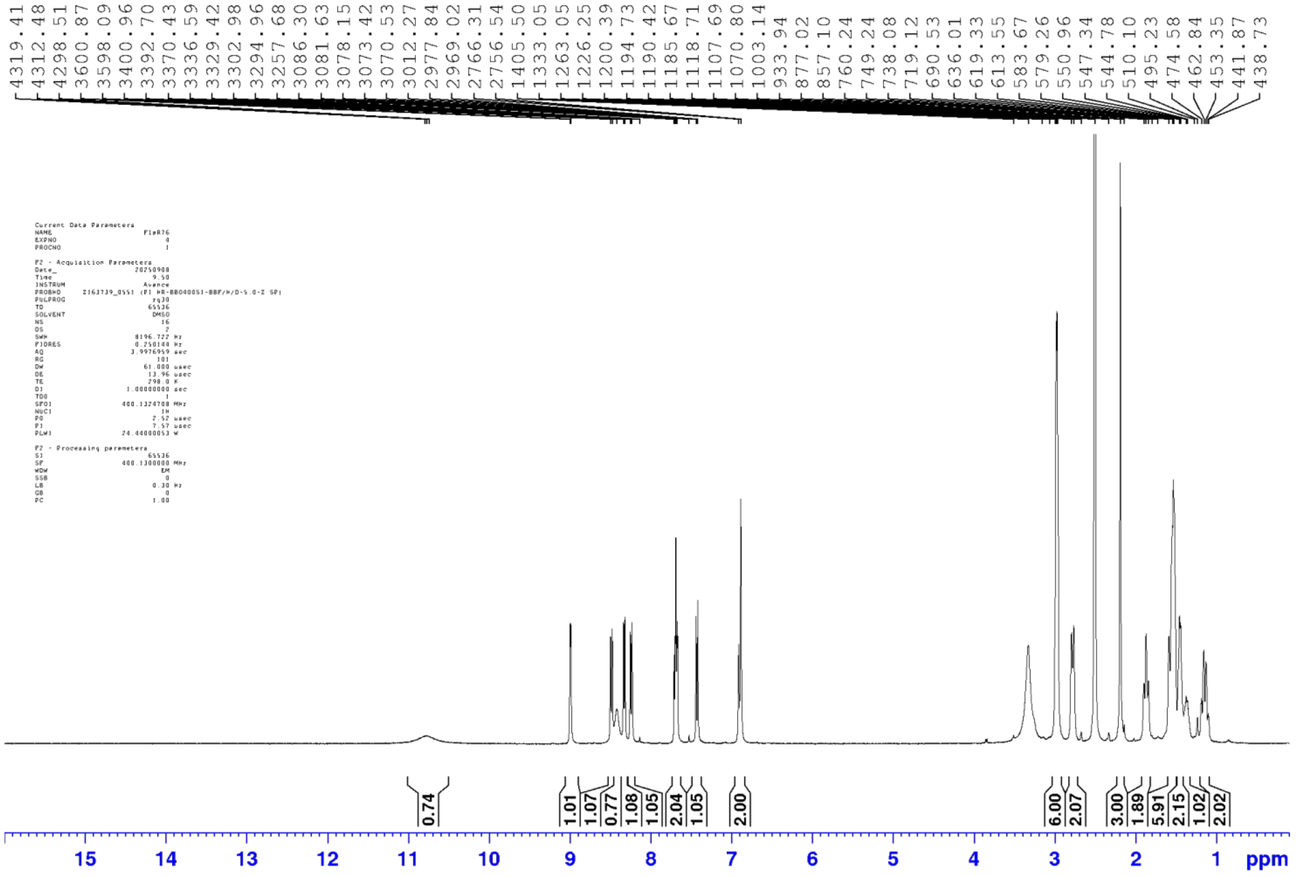

Figure S54. ^{1}HNMR spectrum of compound **26**.

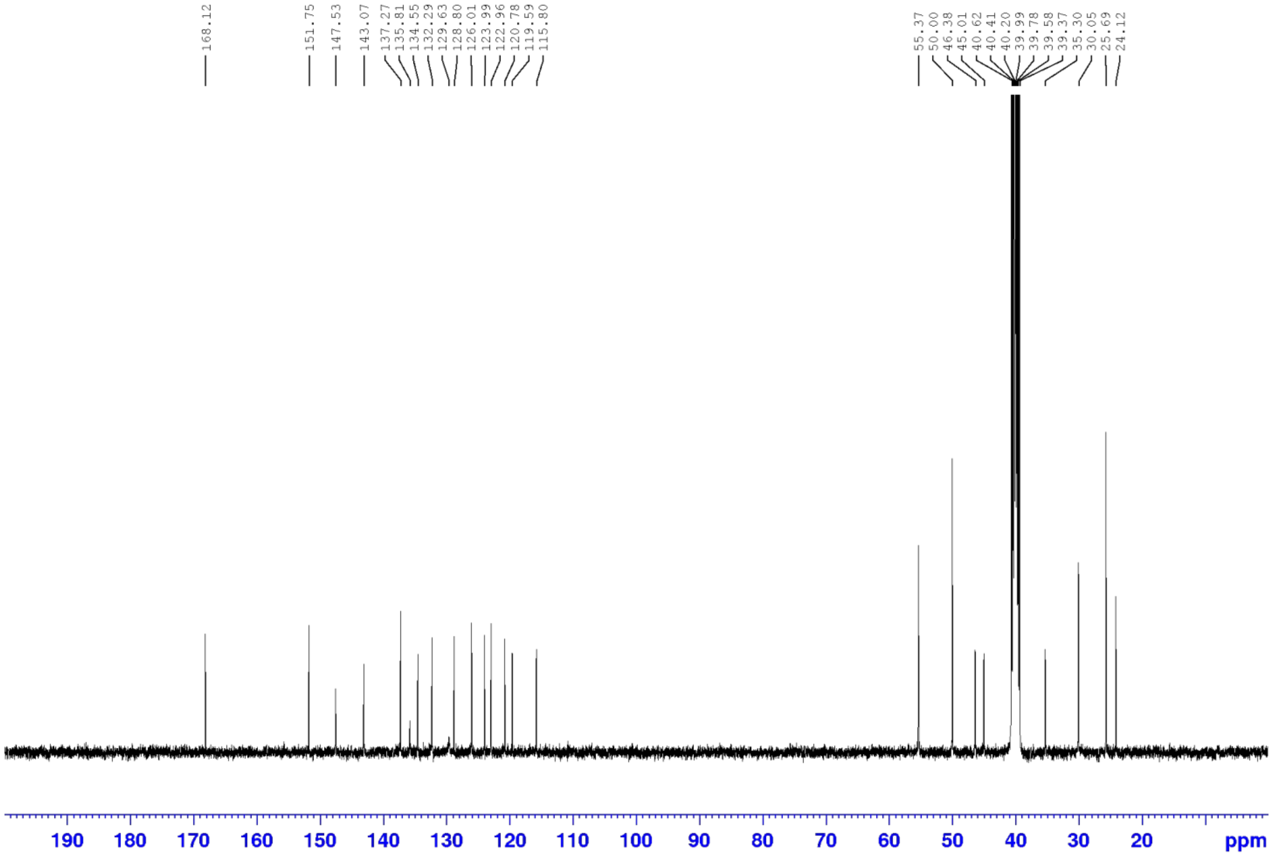

Figure S55. ^{13}CNMR spectrum of compound **26**.

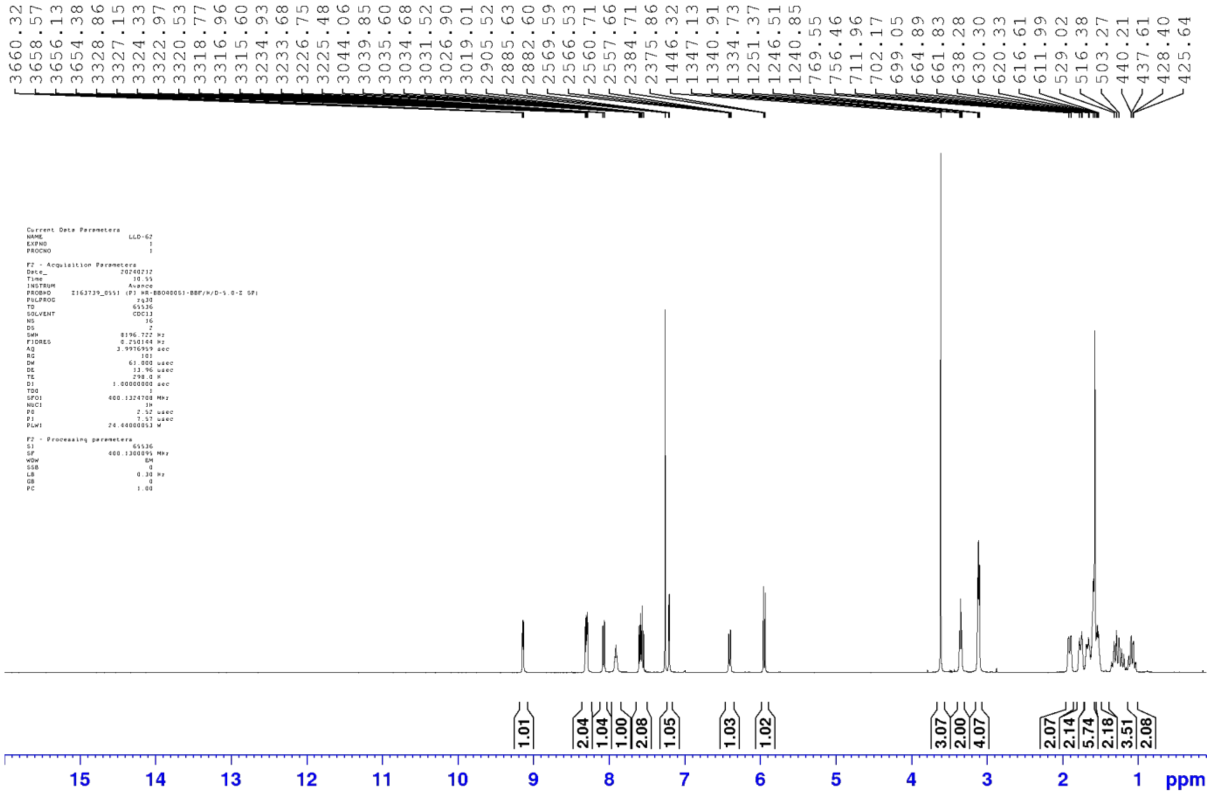

Figure S56. ^{1}HNMR spectrum of compound **27**.

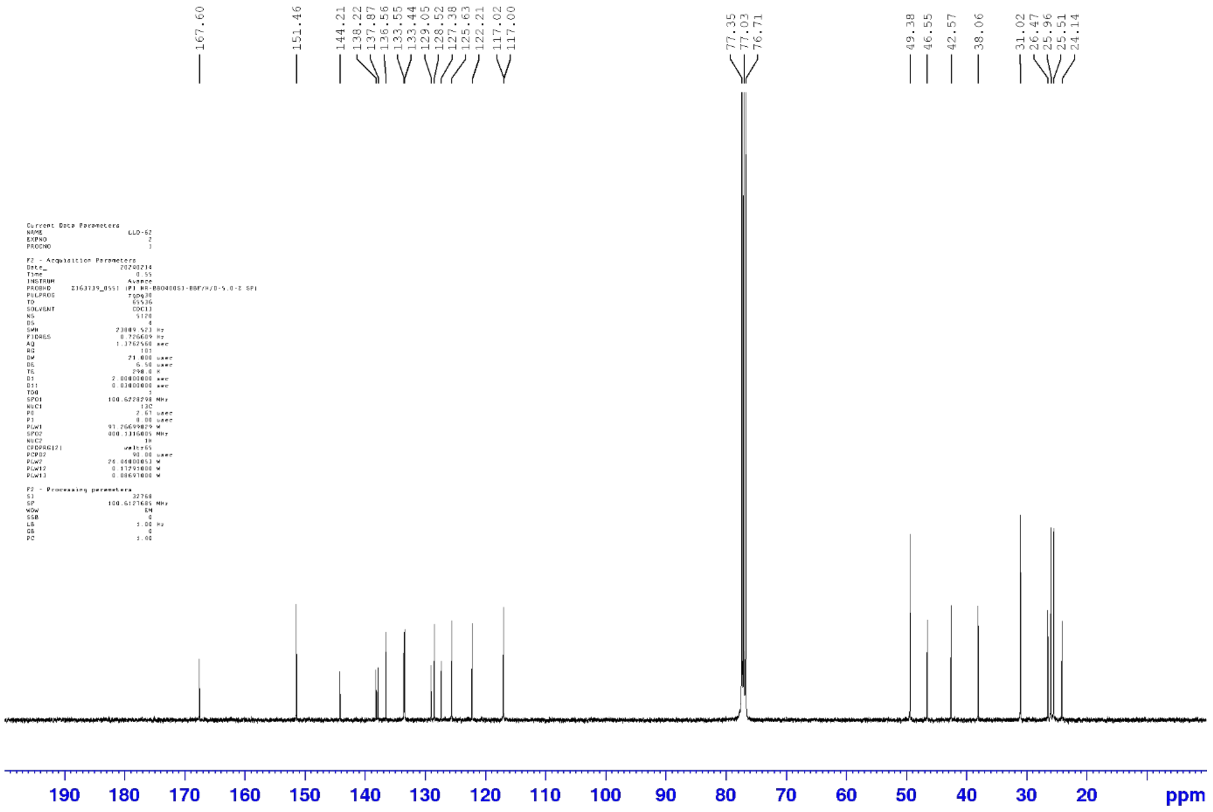

Figure S57. ^{13}CNMR spectrum of compound **27**.

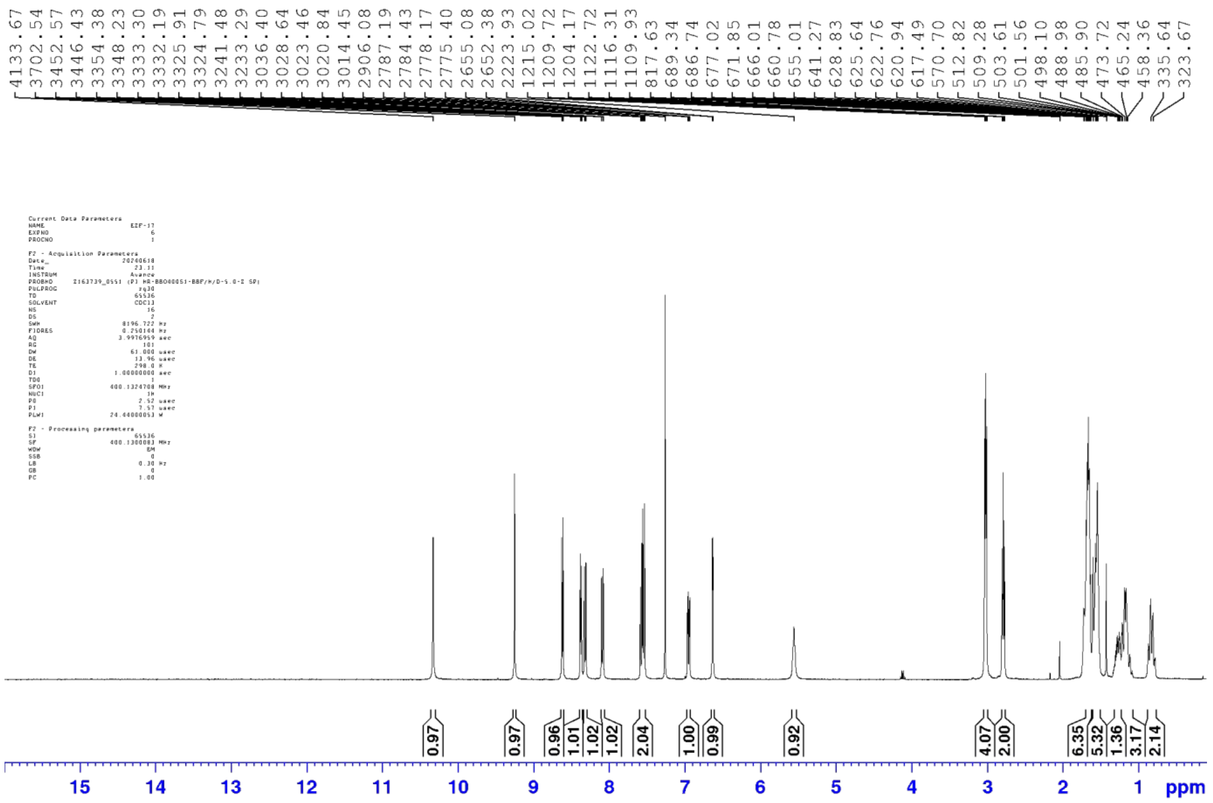

Figure S58. ^{1}HNMR spectrum of compound **28**.

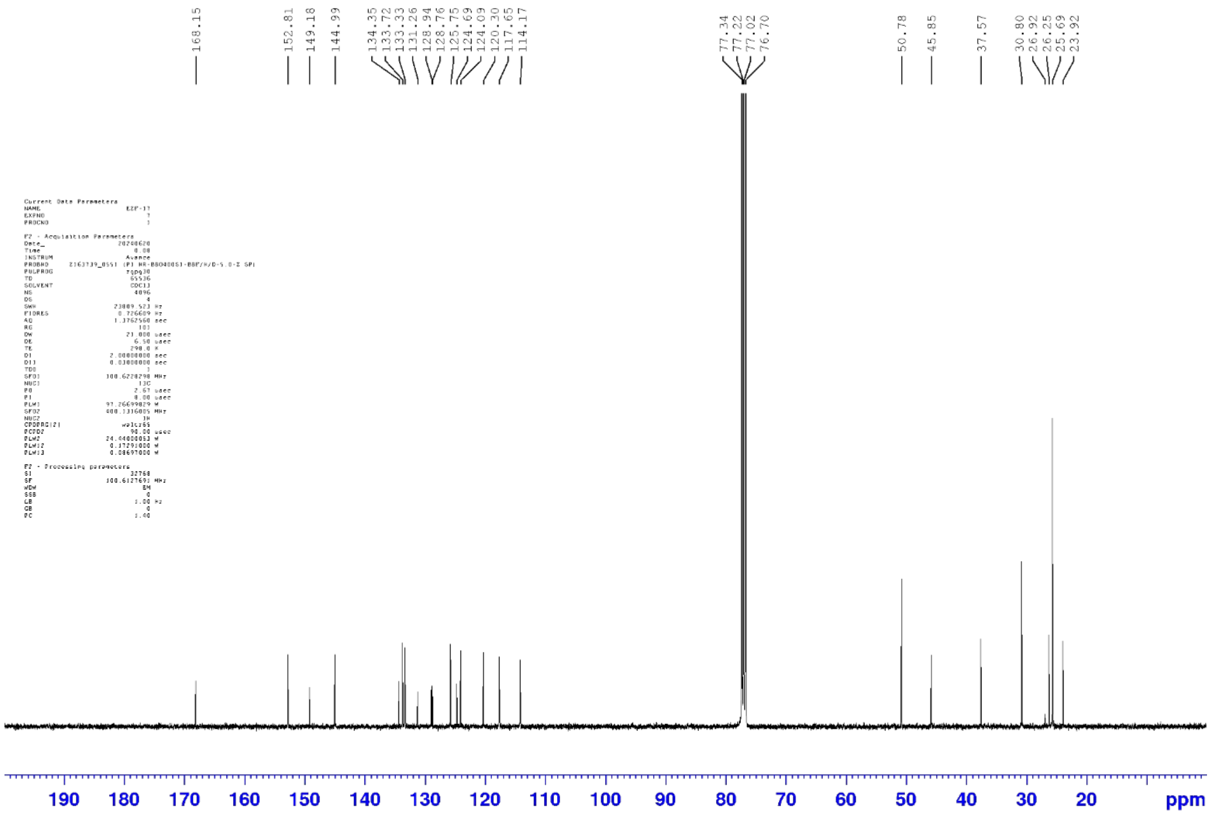

Figure S59. ^{13}CNMR spectrum of compound **28**.

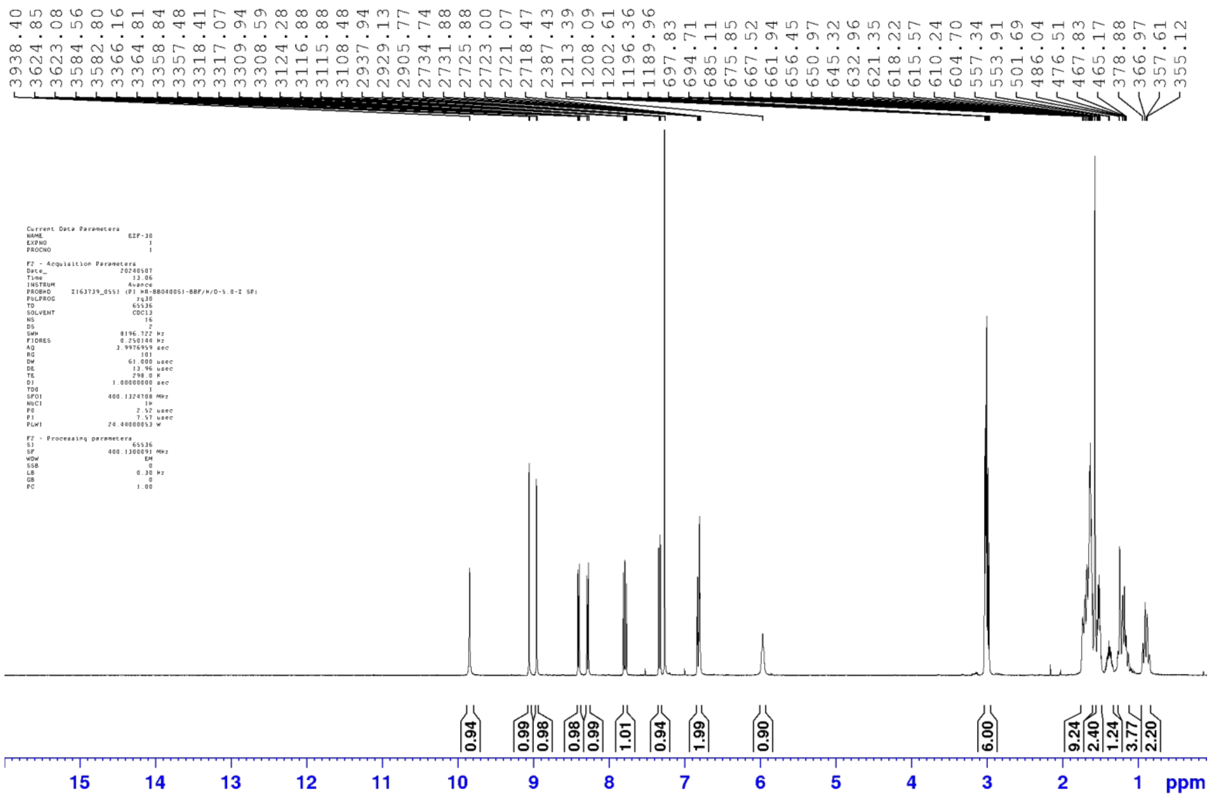

Figure S60. ^{1}HNMR spectrum of compound **29**.

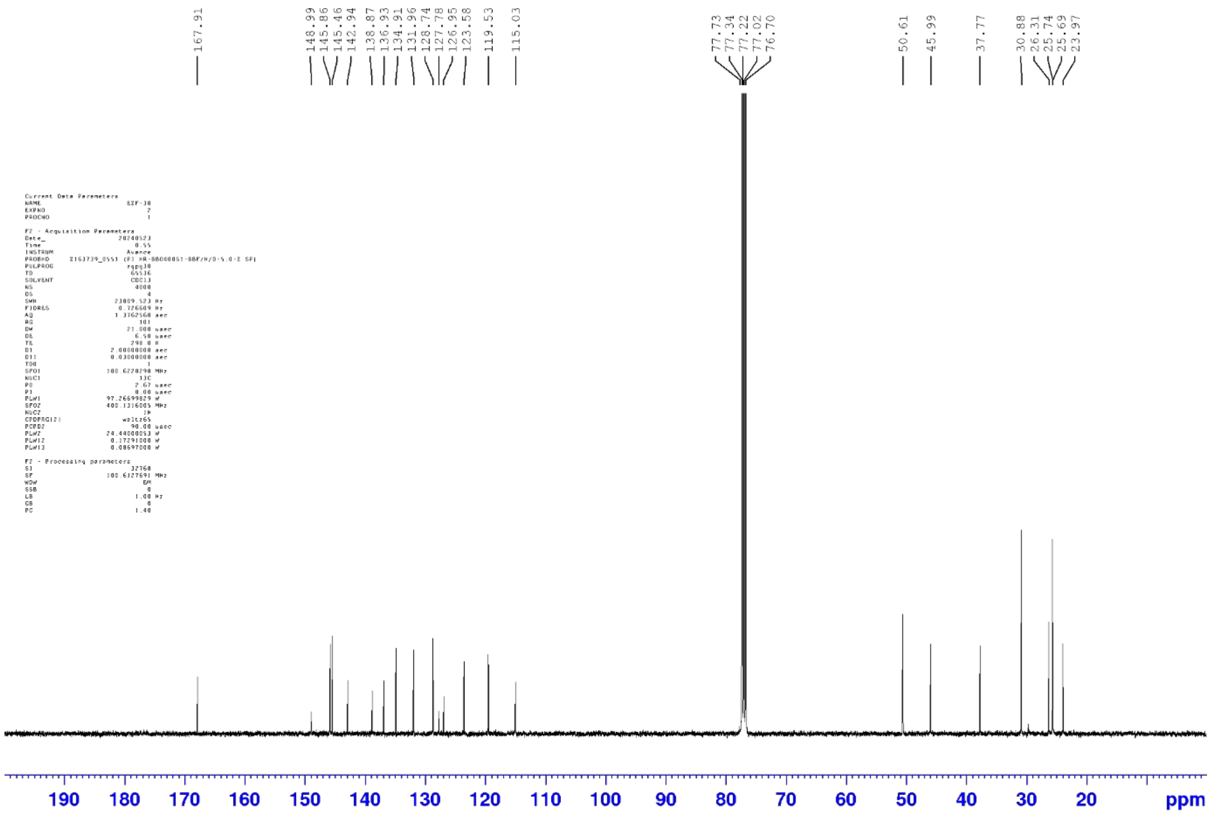

Figure S61. ^{13}CNMR spectrum of compound **29**.

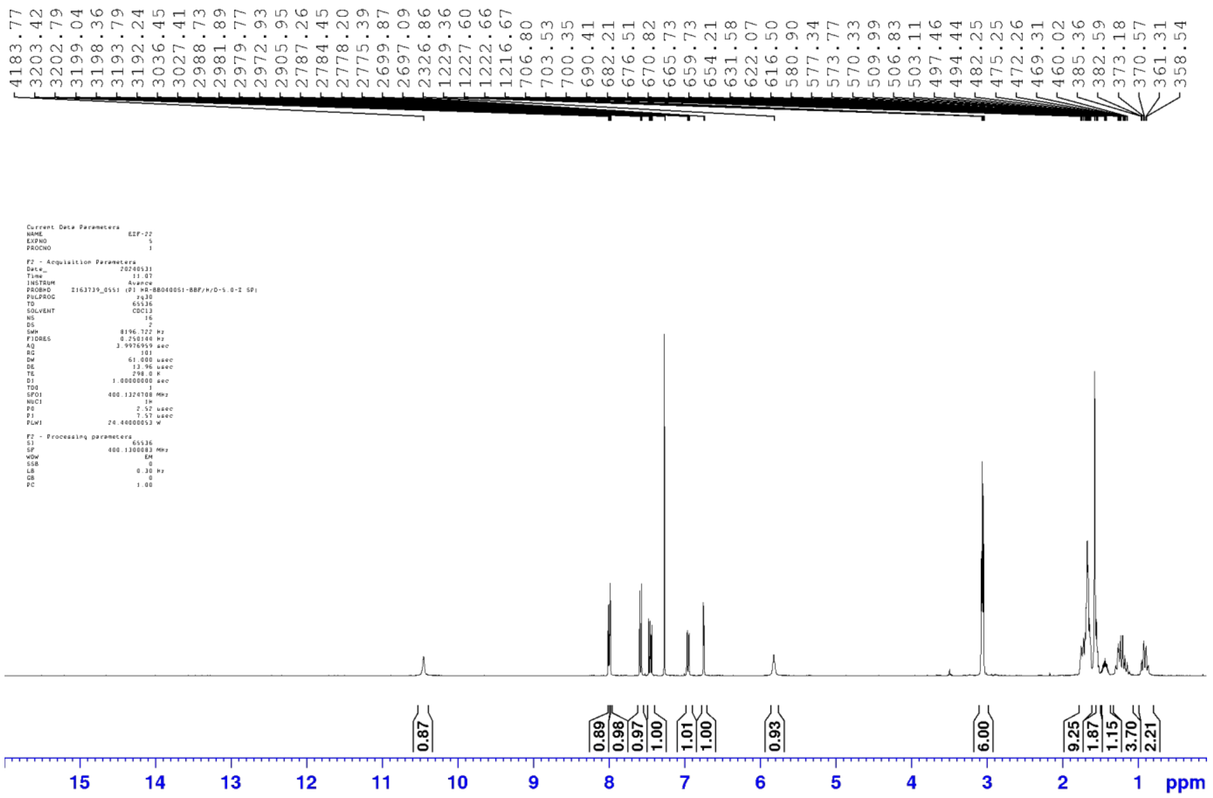

Figure S62. ^{1}HNMR spectrum of compound **30**.

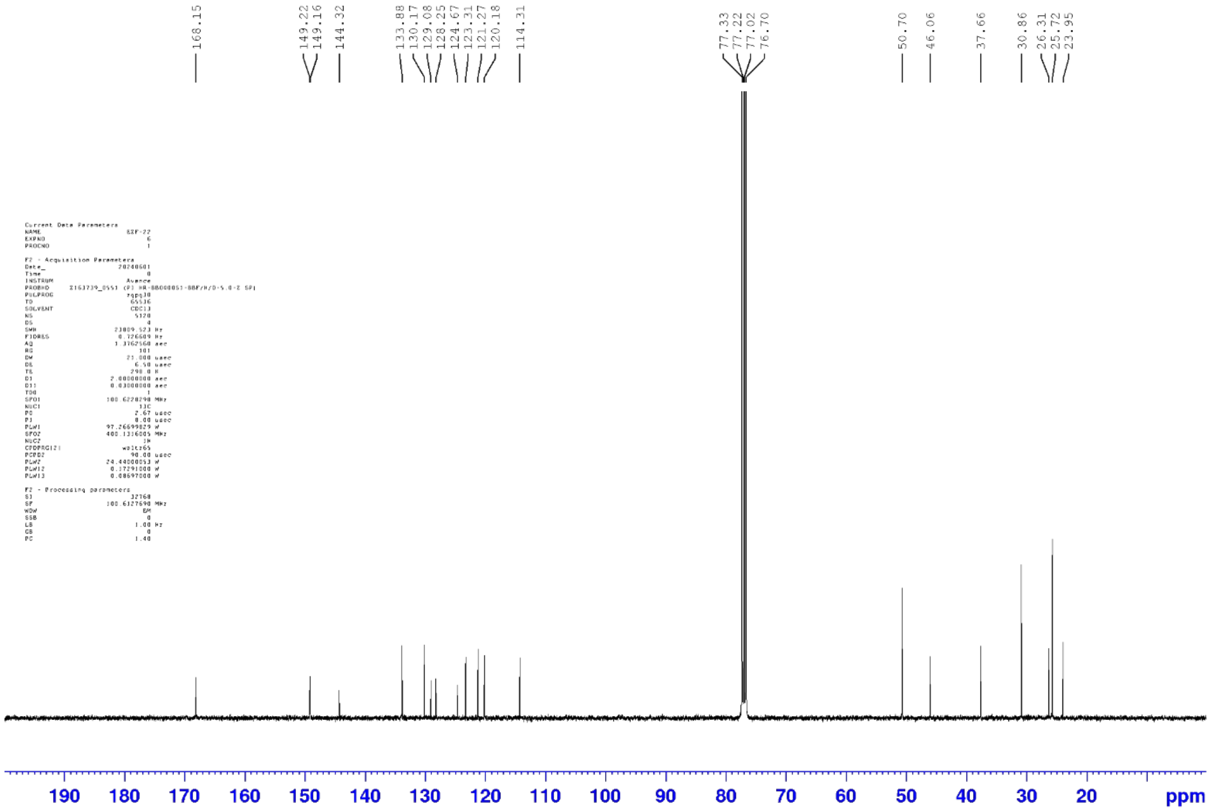

Figure S63. ^{13}CNMR spectrum of compound **30**.

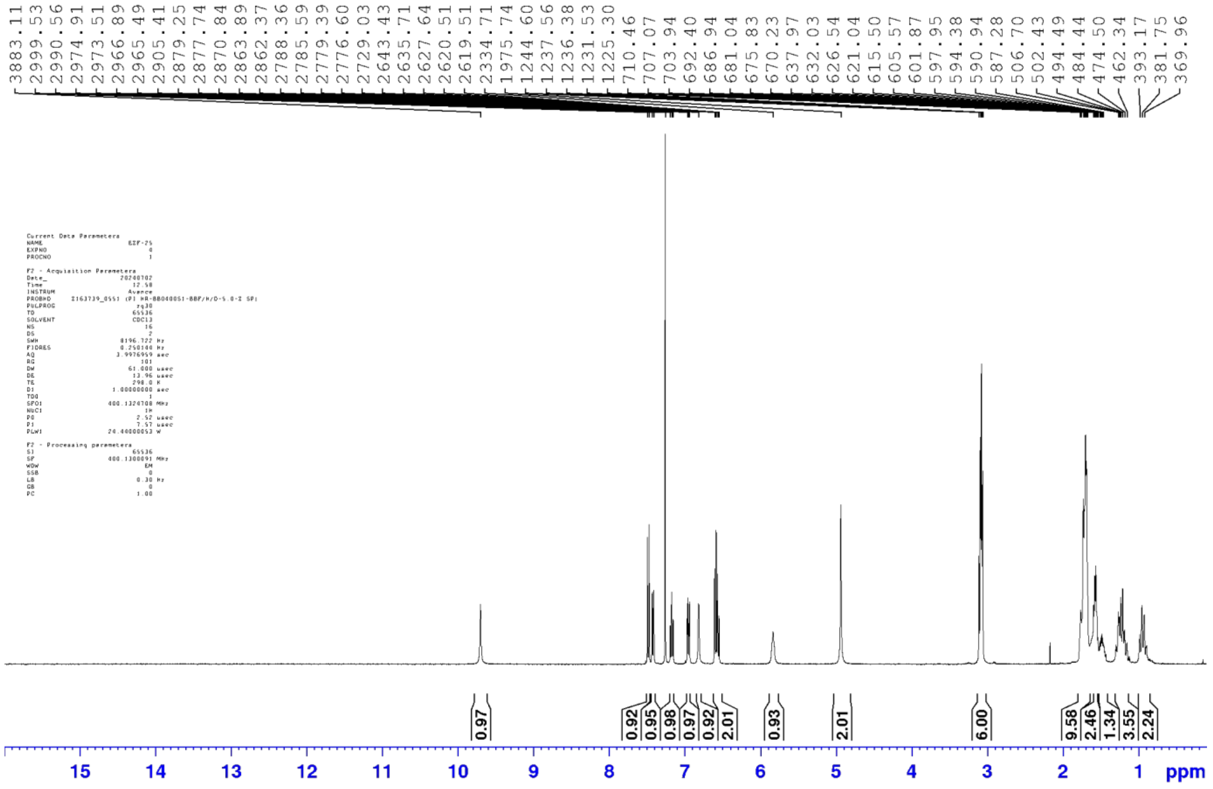

Figure S64. ^{1}HNMR spectrum of compound **31**.

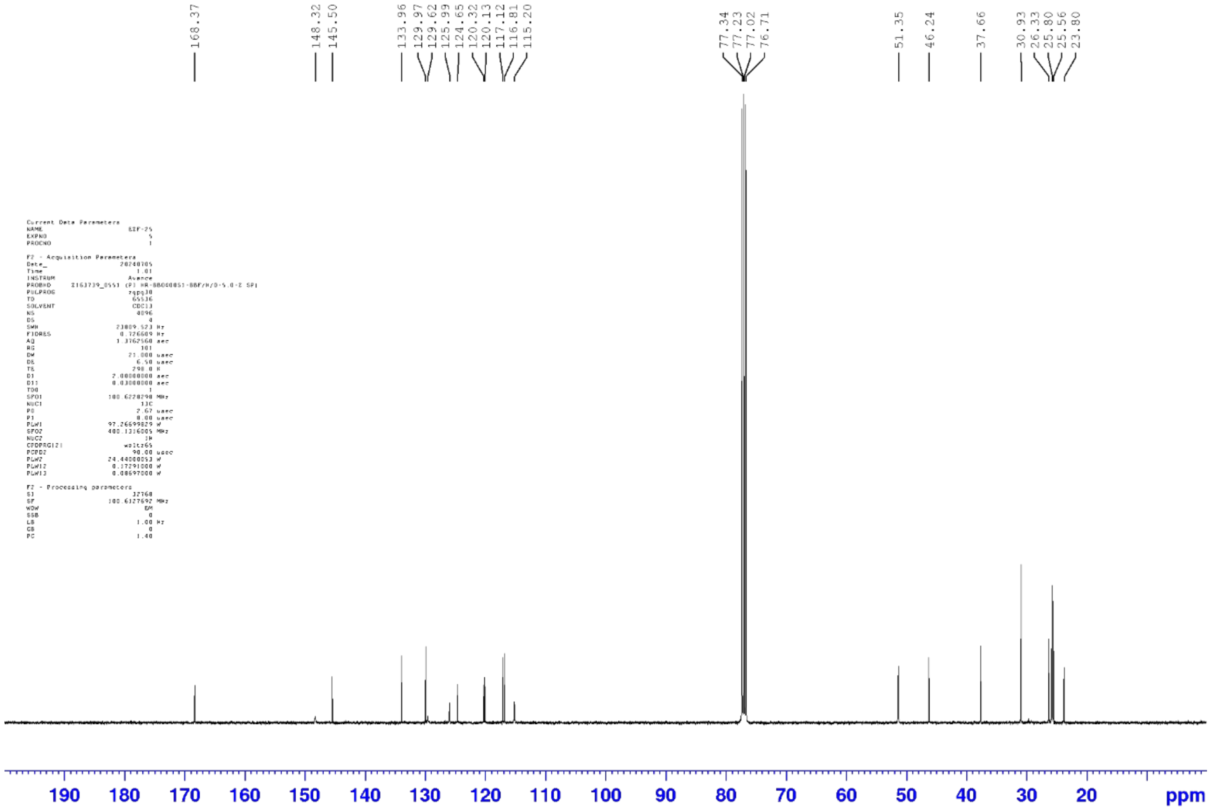

Figure S65. ^{13}CNMR spectrum of compound **31**.

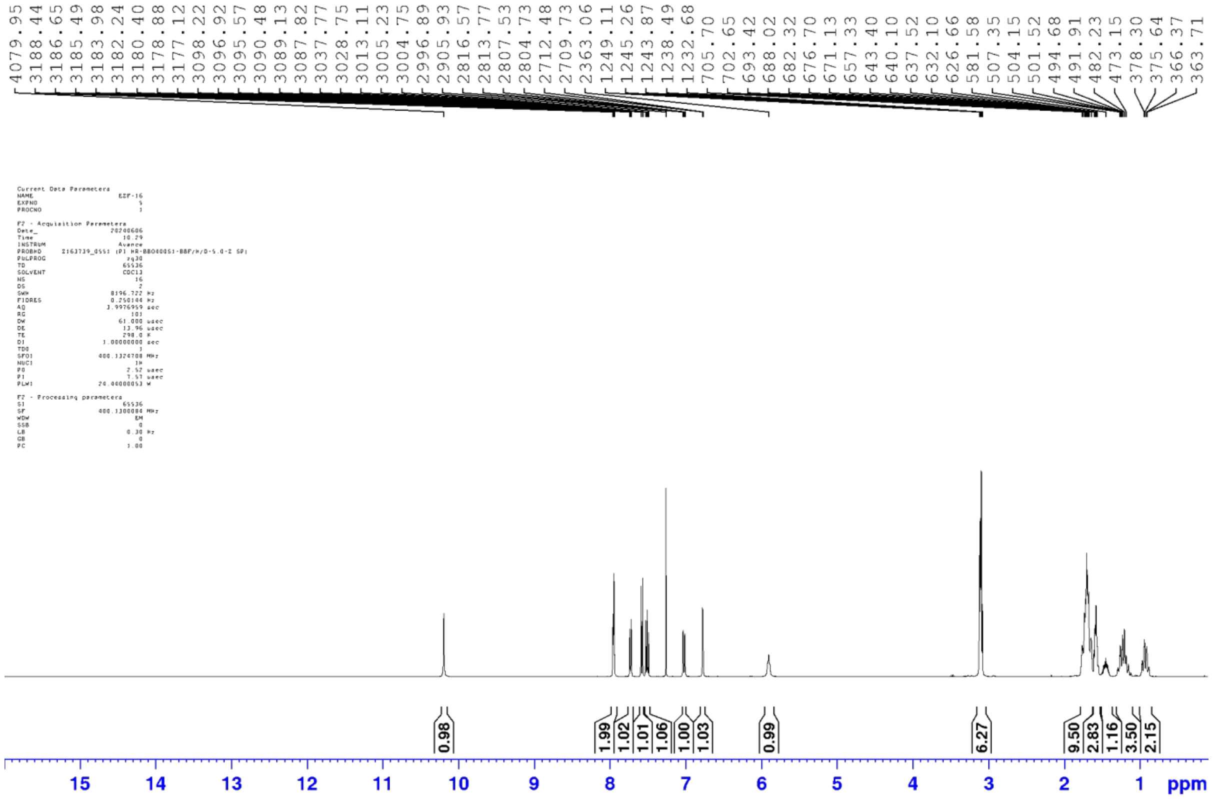

Figure S66. ^{1}HNMR spectrum of compound **32**.

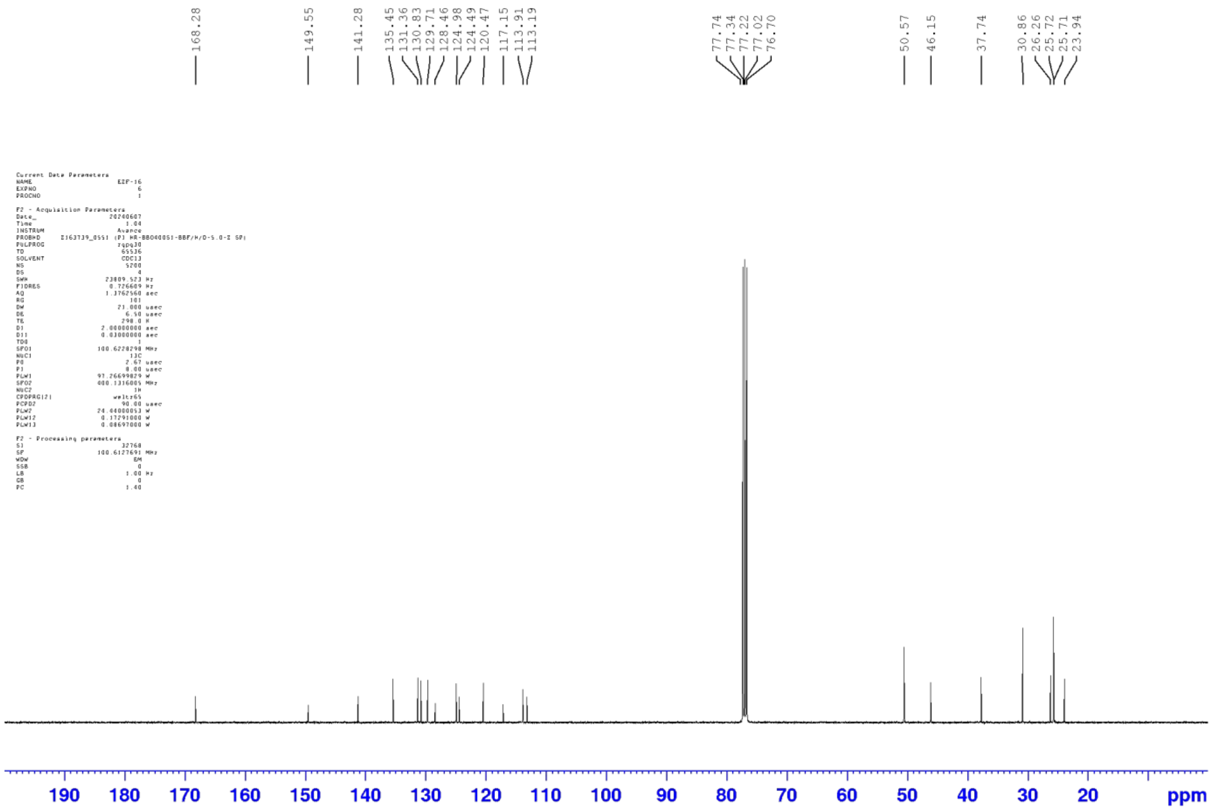

Figure S67. ^{13}CNMR spectrum of compound **32**.

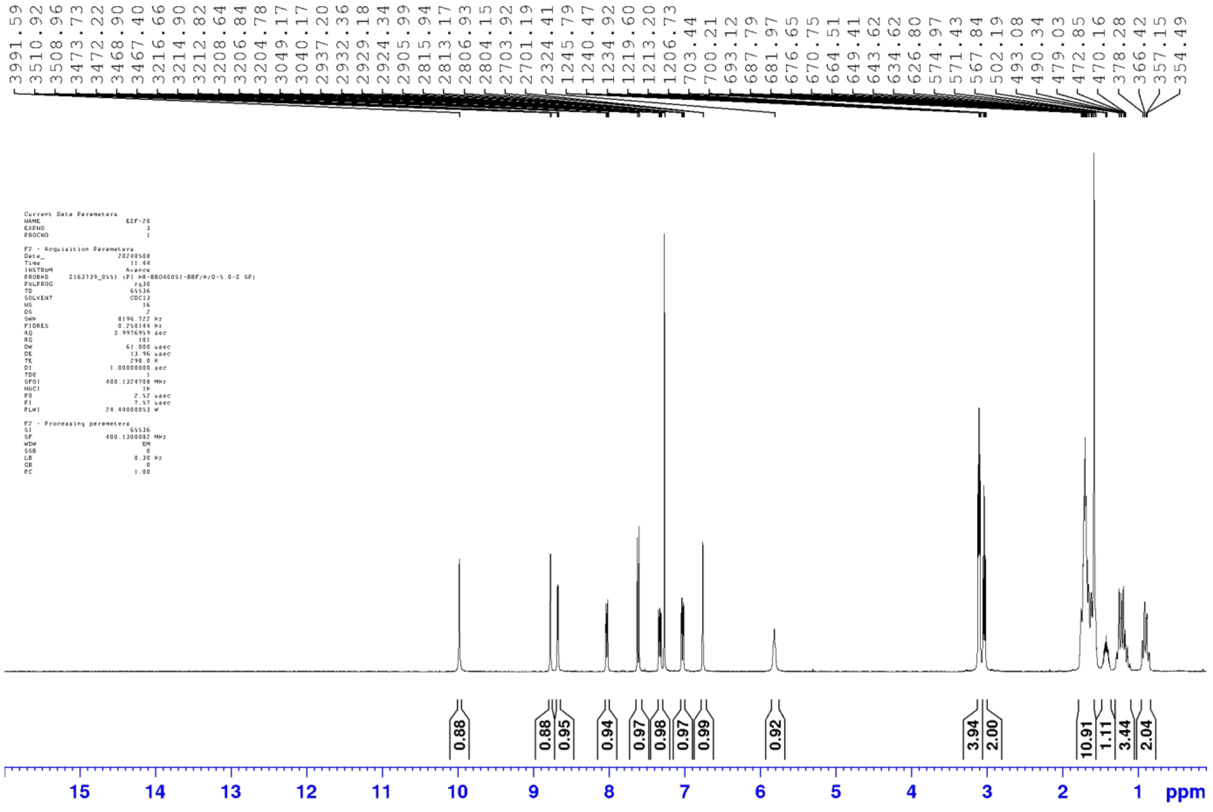

Figure S68. ^{1}HNMR spectrum of compound **33**.

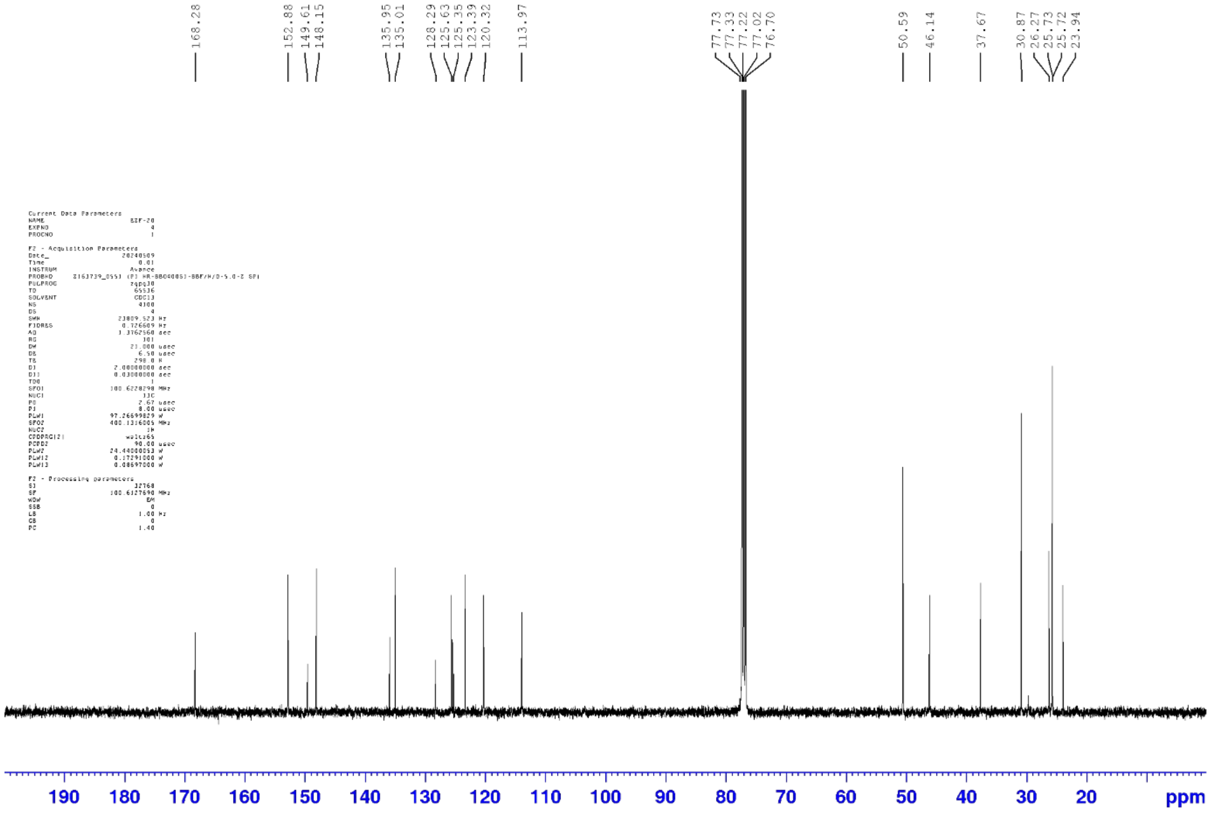

Figure S69. ^{13}CNMR spectrum of compound **33**.

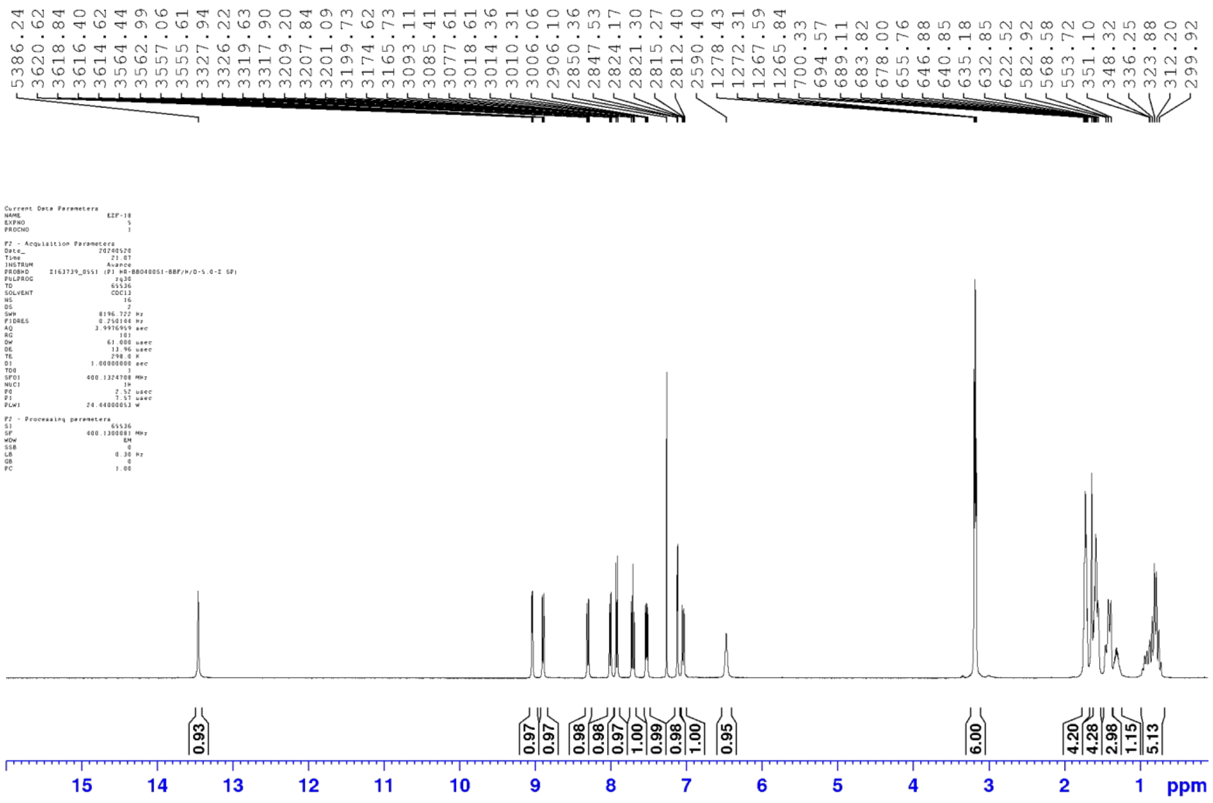

Figure S70. ^{1}HNMR spectrum of compound **34**.

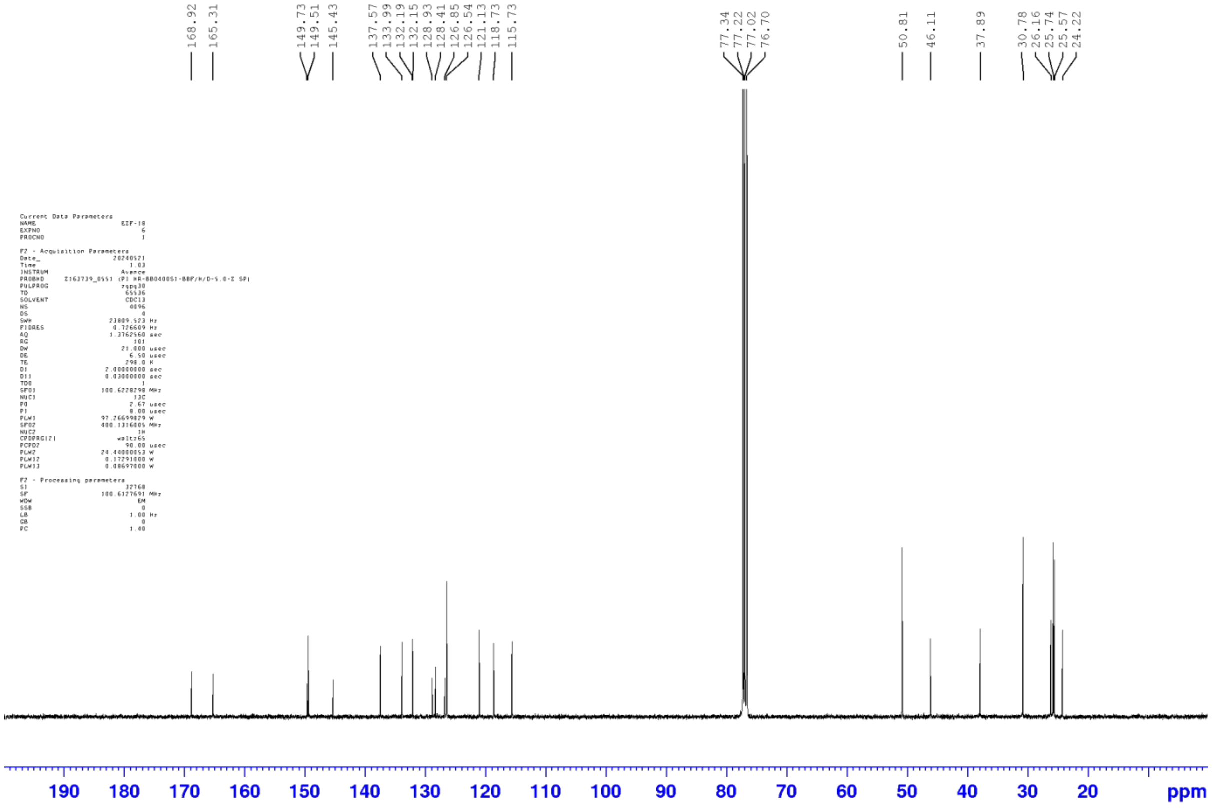

Figure S71. ^{13}CNMR spectrum of compound **34**.

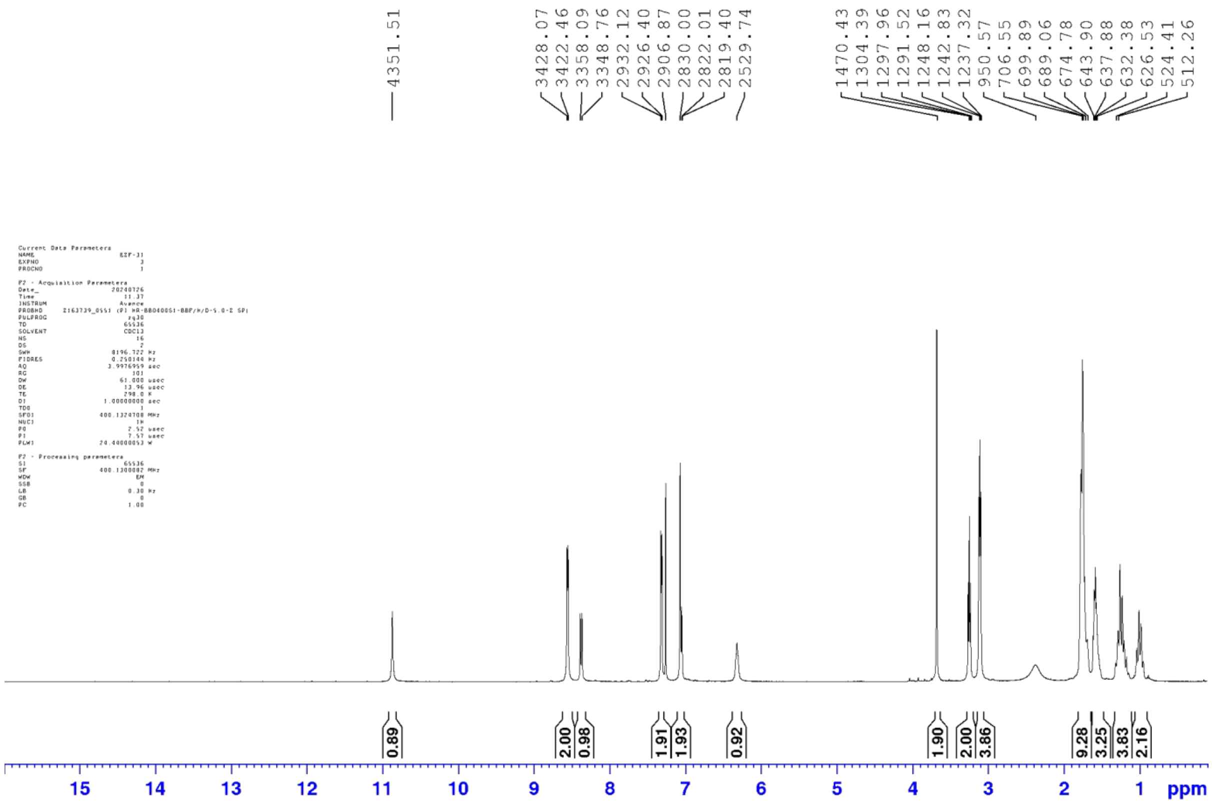

Figure S72. ^{1}HNMR spectrum of compound **35**.

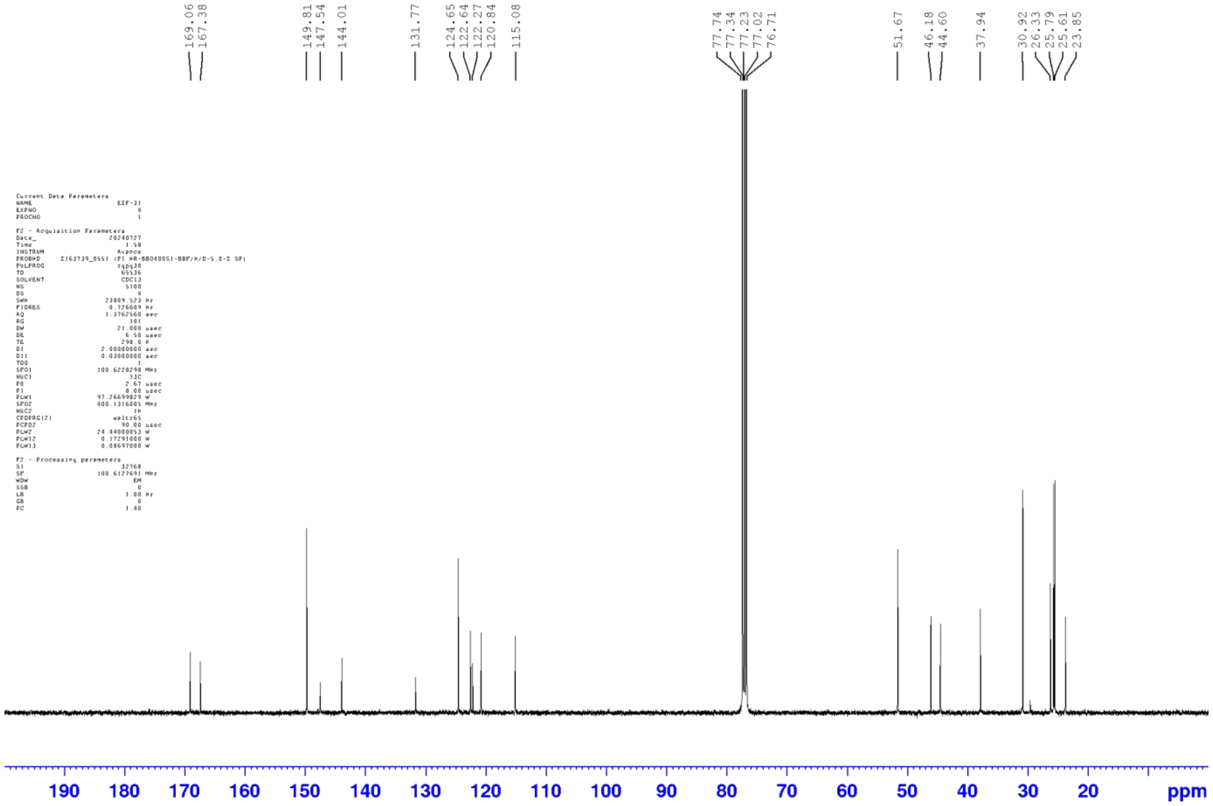

Figure S73. ^{13}CNMR spectrum of compound **35**.

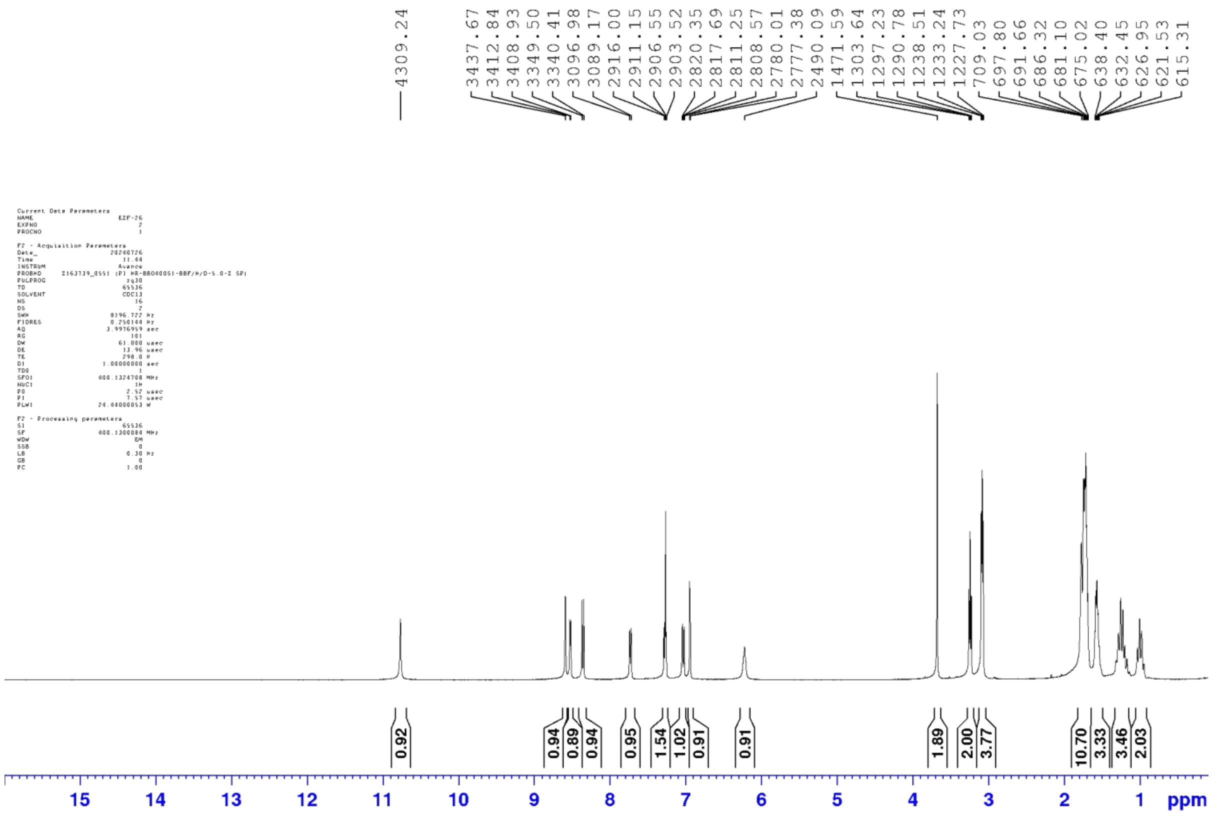

Figure S74. ^{1}HNMR spectrum of compound **36**.

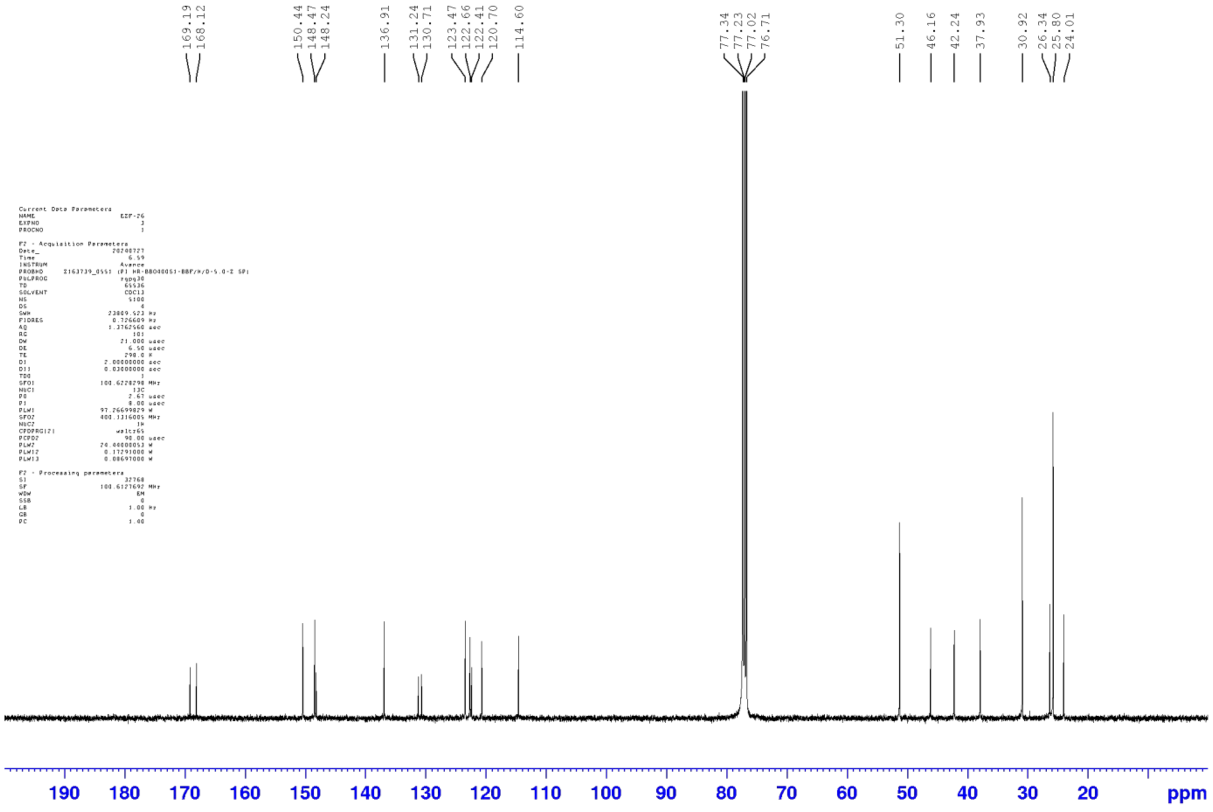

Figure S75. ^{13}CNMR spectrum of compound **36**.

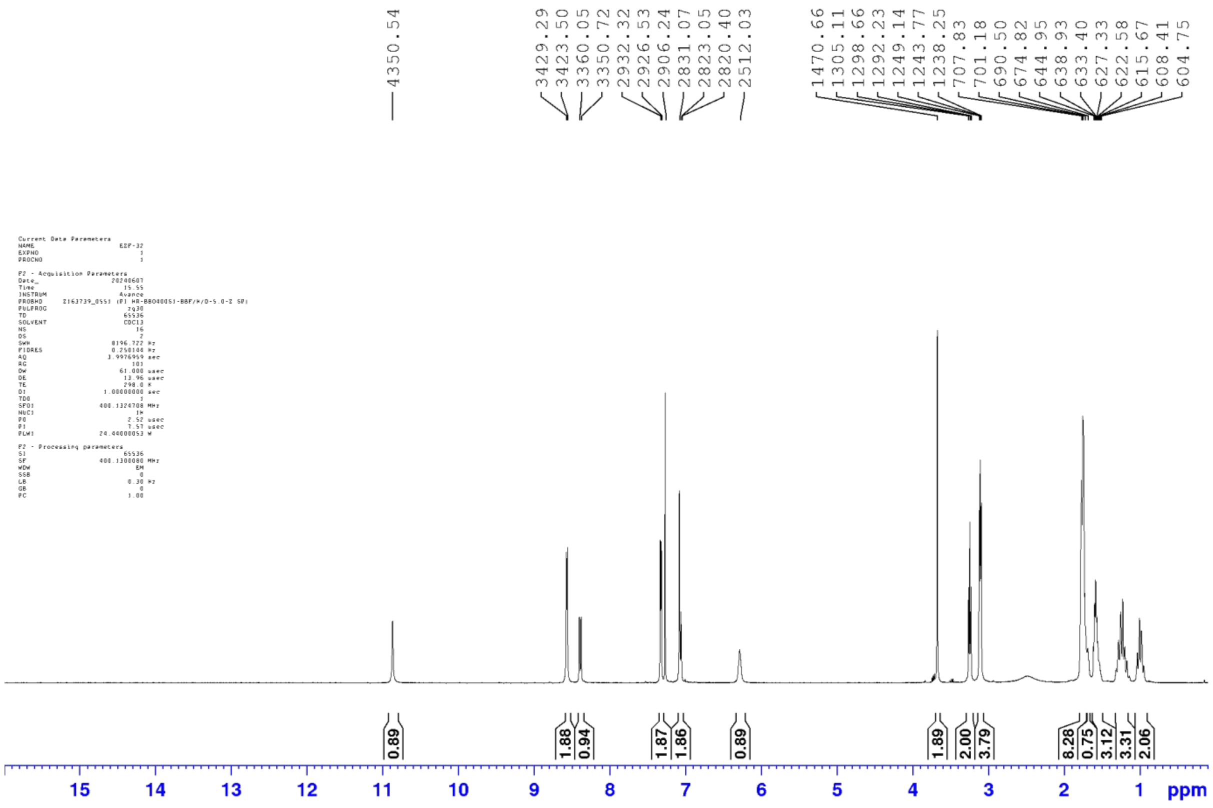

Figure S76. ^{1}HNMR spectra of compound **37**.

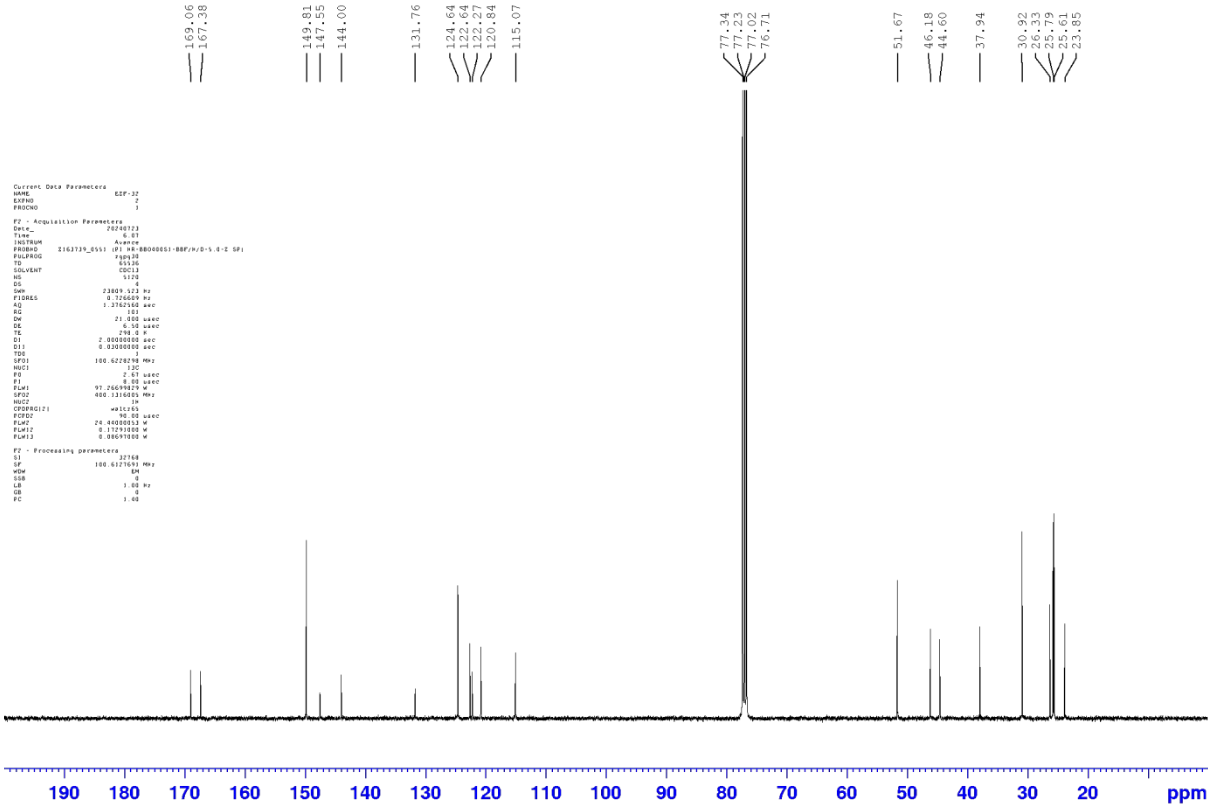

Figure S77. ^{13}CNMR spectrum of compound **37**.

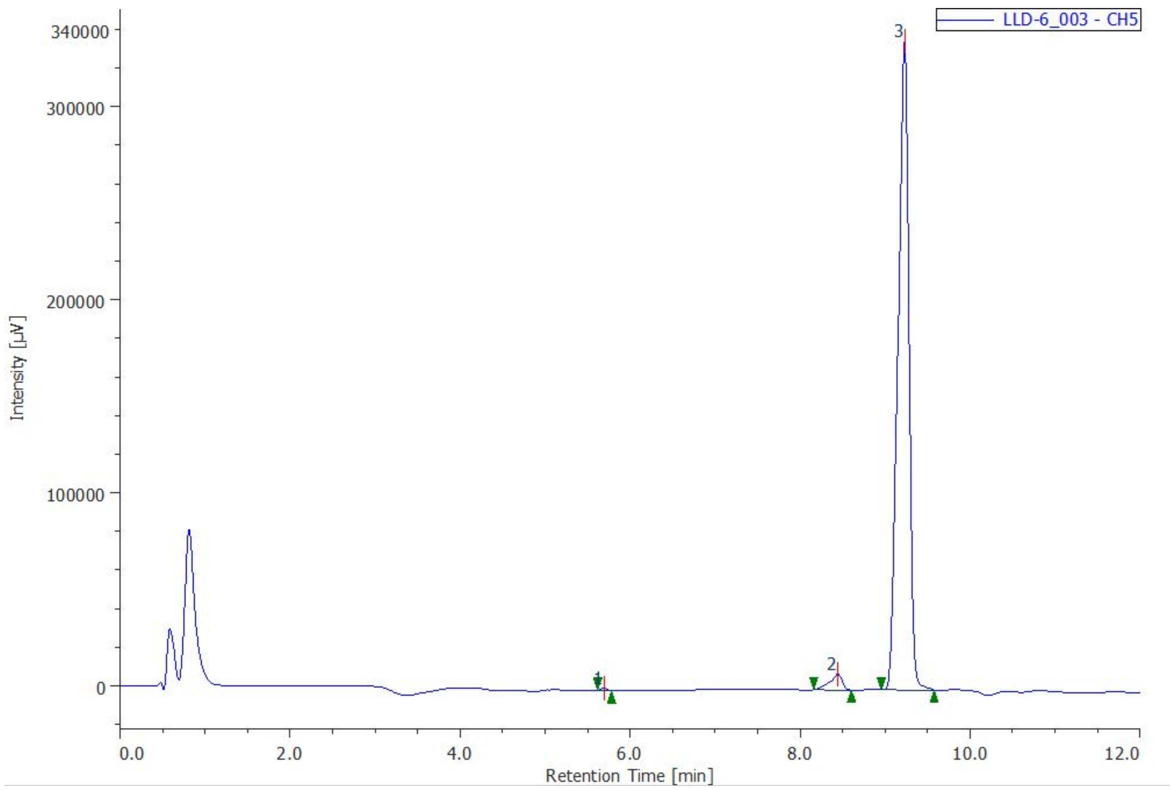

#	Peak Name	CH	tR	Area	Height	Area%	Height%	Quantity	NTP	Resolution	Symmetry Factor	Warning
1	Peak-001	5	5.690	5603	1022	0.181	0.296	N/A	21093	13.531	1.090	
2	Peak-002	5	8.443	84168	8247	2.722	2.390	N/A	18056	3.194	0.735	
3	Peak-003	5	9.227	3002532	335798	97.097	97.314	N/A	23563	N/A	0.862	
Total		5		3092303	345067			0.00000				

Figure S78. HPLC of compound **4**.

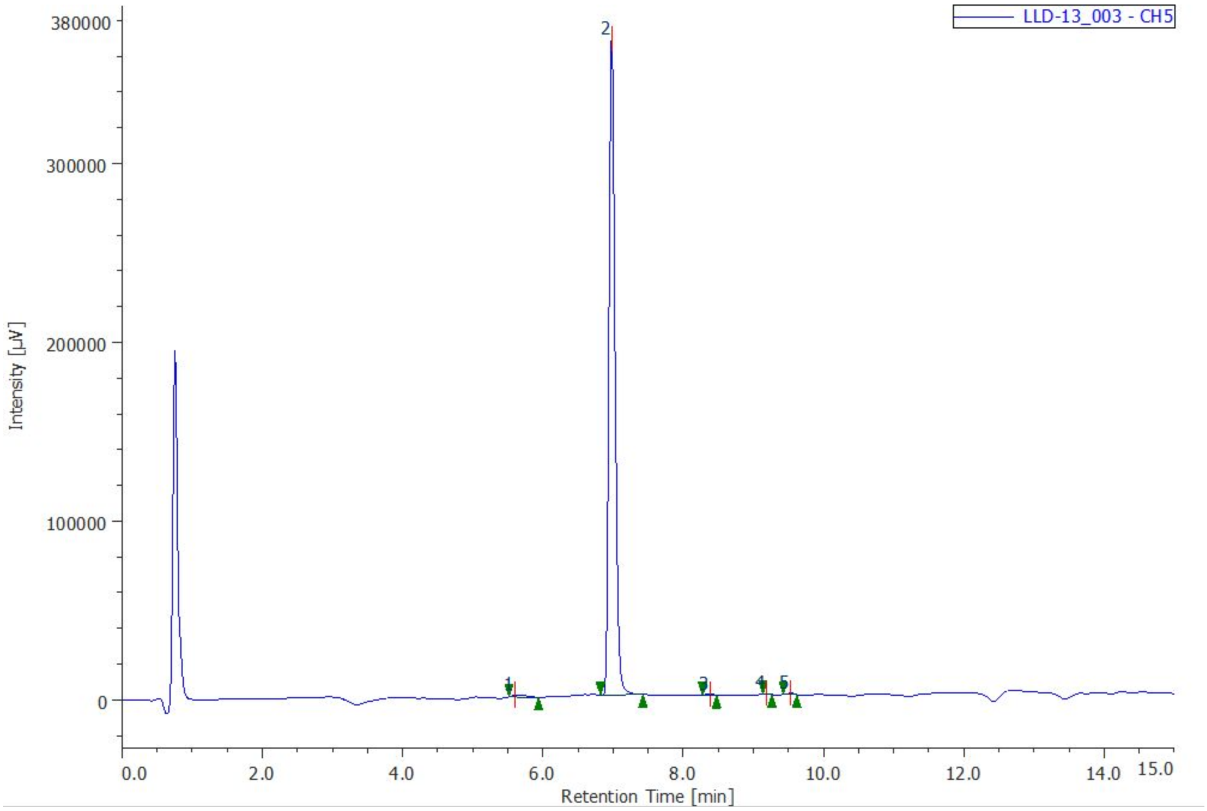

#	Peak Name	CH	tR	Area	Height	Area%	Height%	Quantity	NTP	Resolution	Symmetry Factor	Warning
1	Peak-001	5	5.600	16066	1183	0.717	0.320	N/A	3037	4.913	2.389	
2	Peak-002	5	6.980	2213423	366771	98.788	99.140	N/A	31718	8.526	1.221	
3	Peak-003	5	8.380	3864	621	0.172	0.168	N/A	37746	5.436	0.960	
4	Peak-004	5	9.190	2044	470	0.091	0.127	N/A	84708	2.430	1.143	
5	Peak-005	5	9.533	5177	909	0.231	0.246	N/A	58974	N/A	0.909	
Total		5		2240574	369954			0.00000				

Figure S79. HPLC of compound **5**.

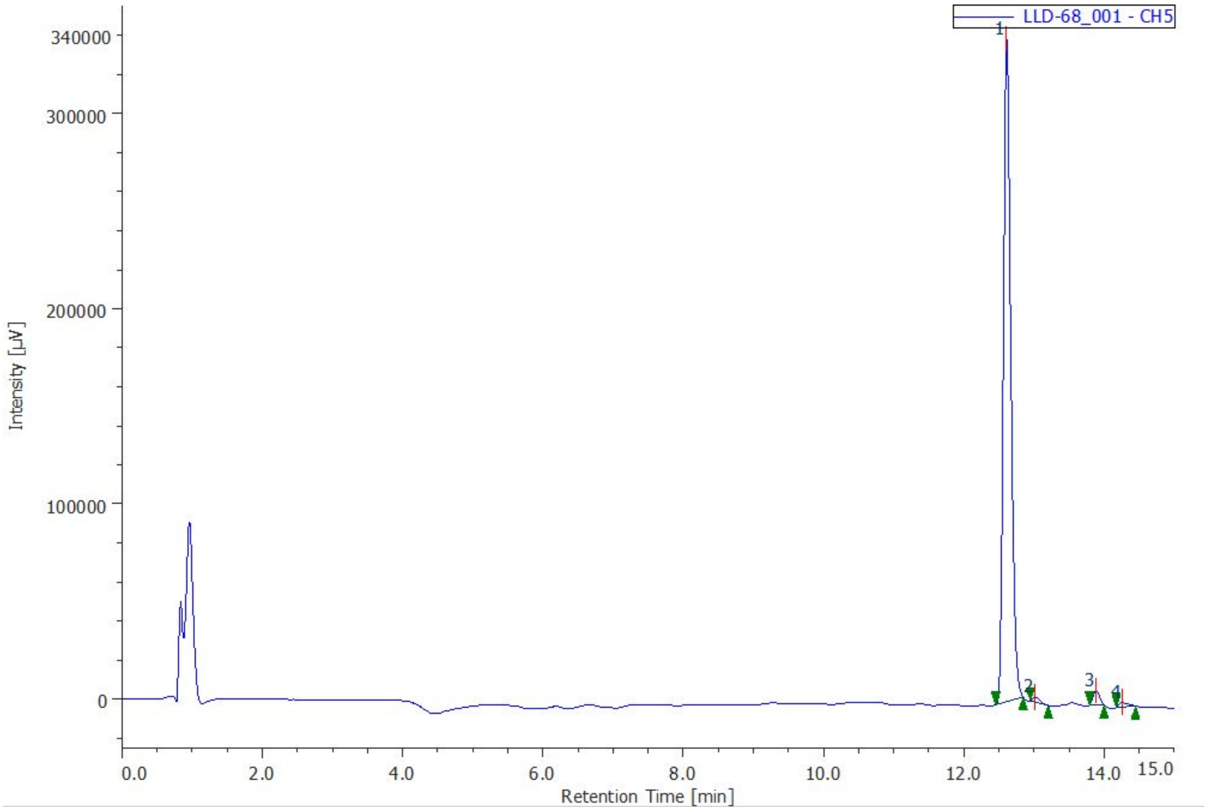

#	Peak Name	CH	tR	Area	Height	Area%	Height%	Quantity	NTP	Resolution	Symmetry Factor	Warning
1	Peak-001	5	12.610	2485227	339582	96.729	96.583	N/A	69967	2.121	1.242	
2	Peak-002	5	13.017	17258	2447	0.672	0.696	N/A	72172	4.661	1.592	
3	Peak-003	5	13.883	47158	7338	1.835	2.087	N/A	96173	1.740	1.116	
4	Peak-004	5	14.257	19612	2227	0.763	0.633	N/A	51589	N/A	1.607	
Total		5		2569255	351594			0.00000				

Figure S80. HPLC of compound **6**.

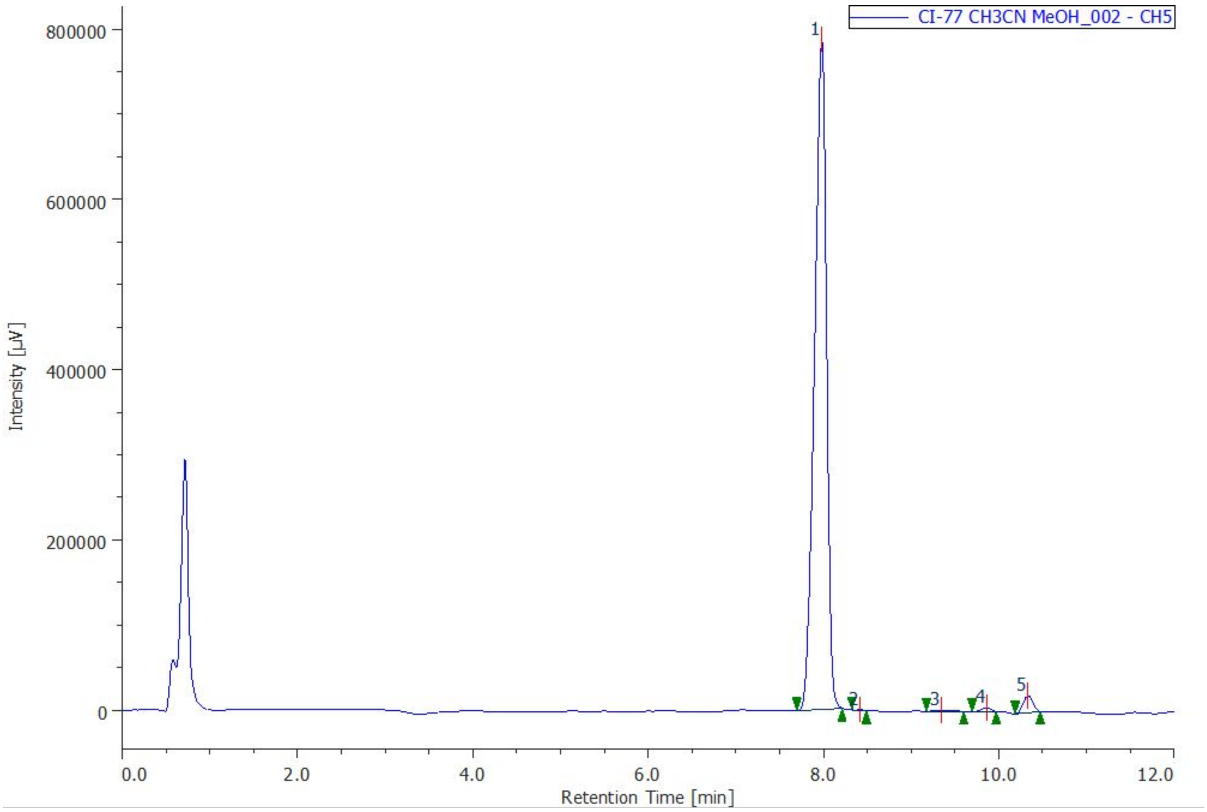

#	Peak Name	CH	tR	Area	Height	Area%	Height%	Quantity	NTP	Resolution	Symmetry Factor	Warning
1	Peak-001	5	7.980	7440854	786959	97.133	96.708	N/A	16224	2.348	0.832	
2	Peak-002	5	8.413	3830	903	0.050	0.111	N/A	79314	3.011	1.187	
3	Peak-003	5	9.347	26334	1572	0.344	0.193	N/A	5545	1.428	1.261	
4	Peak-004	5	9.860	35705	4512	0.466	0.554	N/A	32521	2.193	0.863	
5	Peak-005	5	10.333	153752	19803	2.007	2.434	N/A	37276	N/A	0.989	
Total		5		7660475	813749			0.00000				

Figure S81. HPLC of compound **7**.

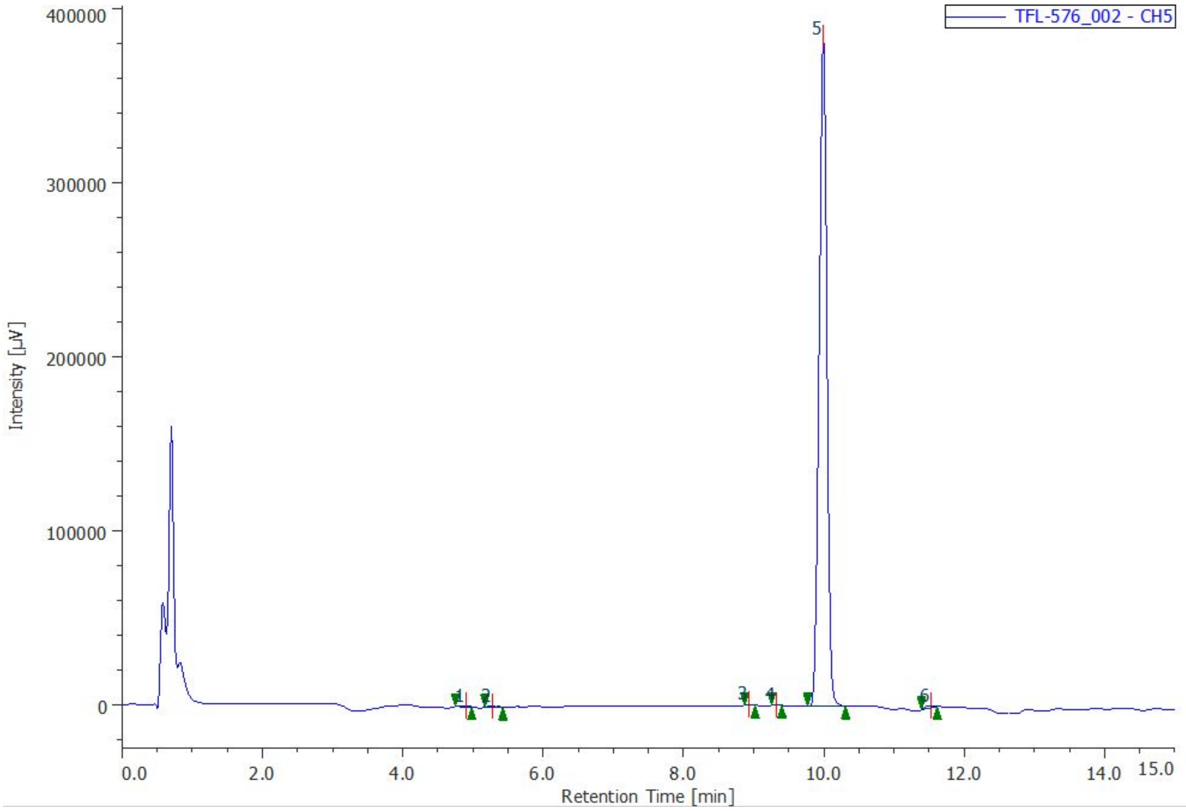

#	Peak Name	CH	tR	Area	Height	Area%	Height%	Quantity	NTP	Resolution	Symmetry Factor	Warning
1	Peak-001	5	4.900	6280	622	0.210	0.161	N/A	3977	1.255	0.756	
2	Peak-002	5	5.273	7351	751	0.246	0.194	N/A	5445	16.522	1.208	
3	Peak-003	5	8.940	1875	342	0.063	0.089	N/A	50459	2.347	1.074	
4	Peak-004	5	9.330	1540	265	0.052	0.069	N/A	46012	3.497	0.955	
5	Peak-005	5	9.990	2958274	383459	99.054	99.188	N/A	38196	6.561	0.965	
6	Peak-006	5	11.520	11221	1158	0.376	0.300	N/A	30662	N/A	0.841	
Total		5		2986541	386597			0.00000				

Figure S82. HPLC of compound **8**.

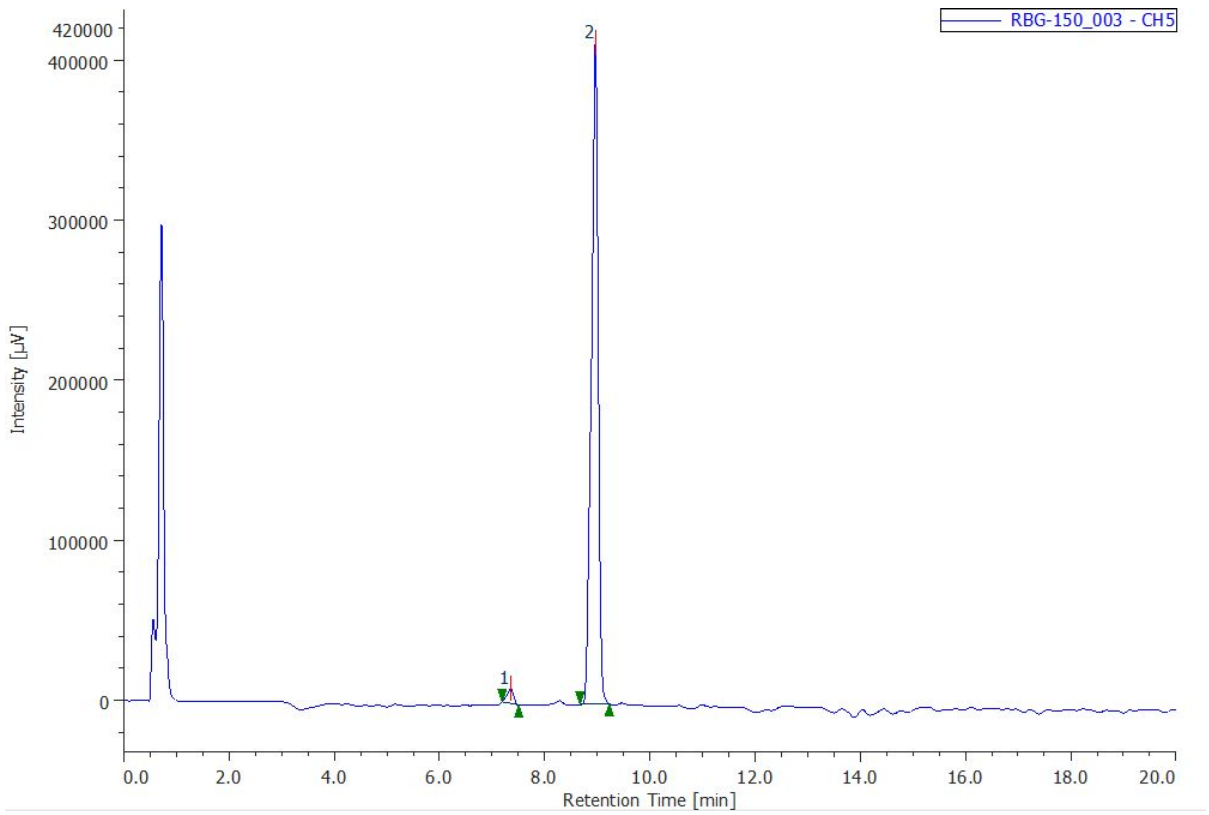

#	Peak Name	CH	tR	Area	Height	Area%	Height%	Quantity	NTP	Resolution	Symmetry Factor	Warning
1	Peak-001	5	7.350	76979	9055	2.041	2.145	N/A	16498	7.016	0.911	
2	Peak-002	5	8.963	3694341	413122	97.959	97.855	N/A	23835	N/A	0.855	
Total		5		3771320	422177			0.00000				

Figure S83. HPLC of compound **9**.

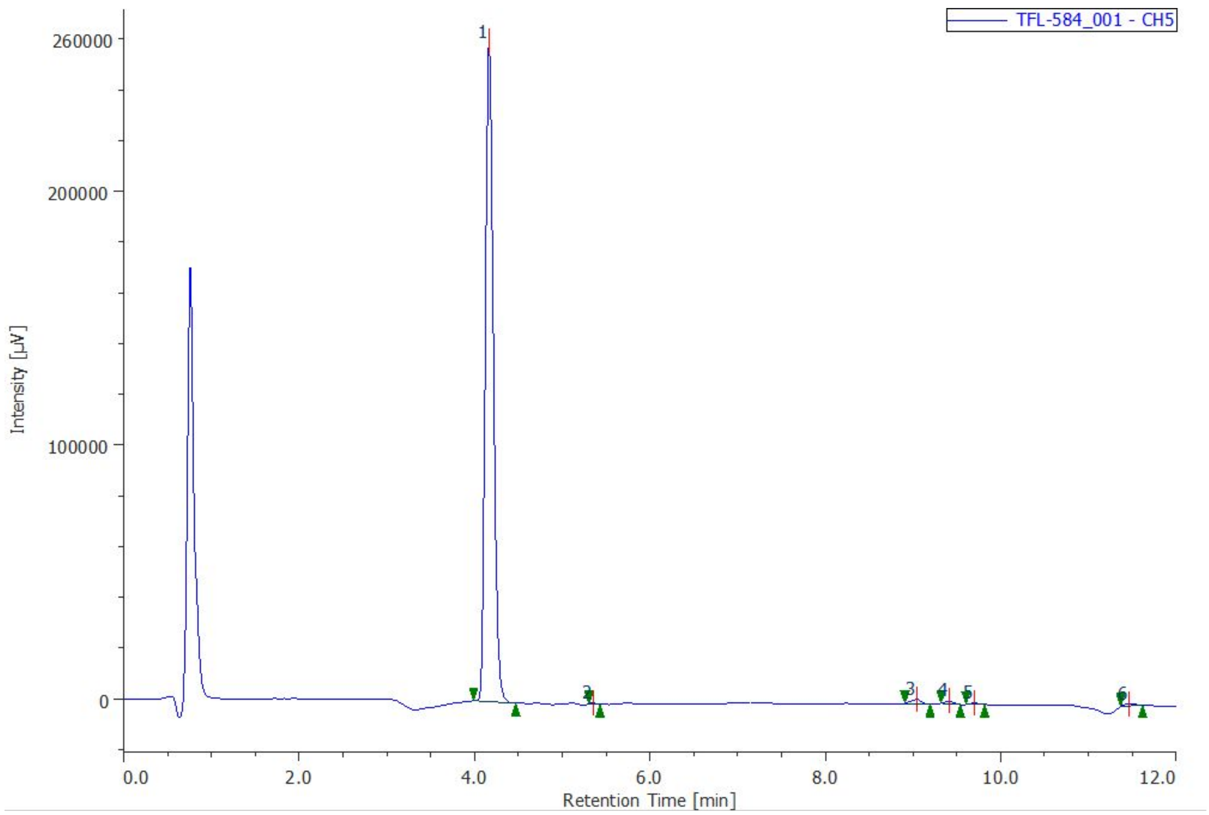

#	Peak Name	CH	tR	Area	Height	Area%	Height%	Quantity	NTP	Resolution	Symmetry Factor	Warning
1	Peak-001	5	4.167	1655234	259711	98.114	98.310	N/A	9890	8.243	1.175	
2	Peak-002	5	5.350	1807	431	0.107	0.163	N/A	31659	22.102	1.261	
3	Peak-003	5	9.037	12951	1694	0.768	0.641	N/A	28470	1.967	0.948	
4	Peak-004	5	9.407	6780	1134	0.402	0.429	N/A	53286	1.593	1.134	
5	Peak-005	5	9.700	3400	474	0.202	0.179	N/A	35367	7.463	1.081	
6	Peak-006	5	11.463	6883	732	0.408	0.277	N/A	29379	N/A	1.225	
Total		5		1687055	264176			0.00000				

Figure S84. HPLC of compound **10**.

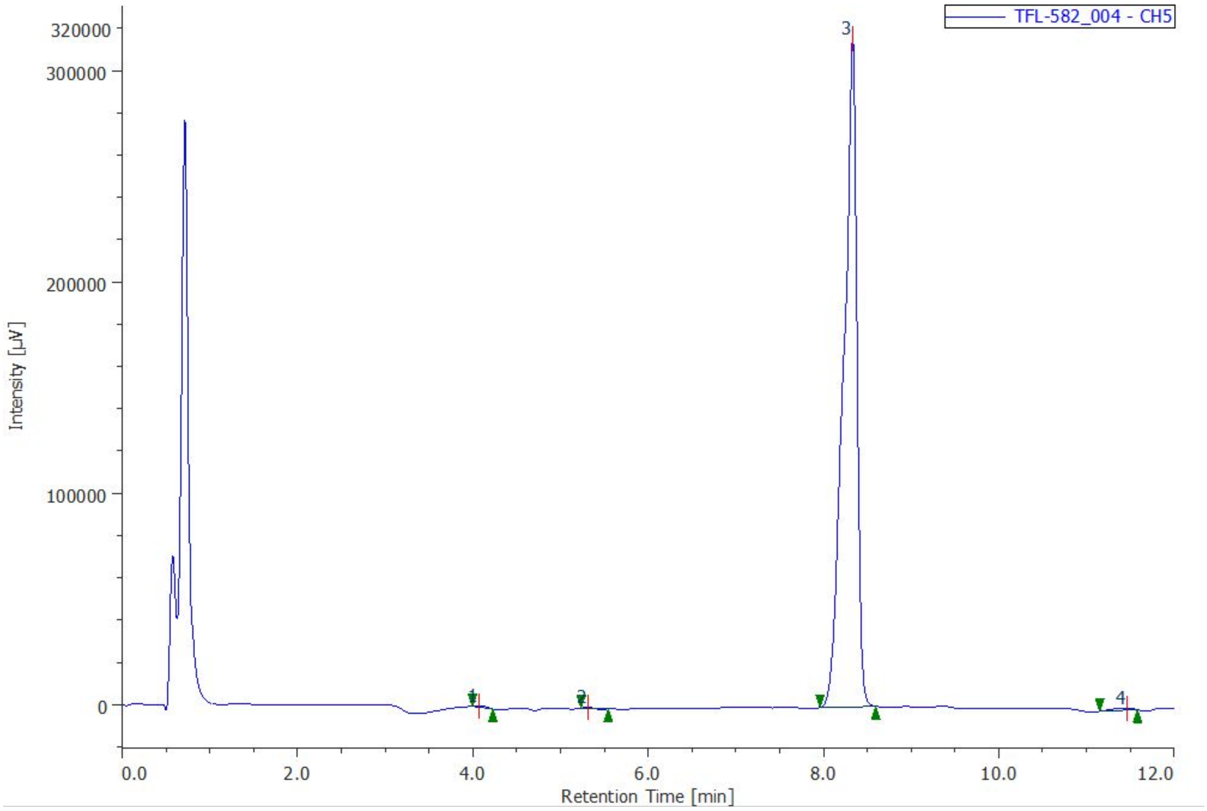

#	Peak Name	CH	tR	Area	Height	Area%	Height%	Quantity	NTP	Resolution	Symmetry Factor	Warning
1	Peak-001	5	4.067	6319	562	0.189	0.177	N/A	3175	5.480	1.665	
2	Peak-002	5	5.317	3160	546	0.094	0.172	N/A	15886	13.485	0.969	
3	Peak-003	5	8.330	3325694	316043	99.226	99.368	N/A	14224	7.617	0.737	
4	Peak-004	5	11.453	16457	904	0.491	0.284	N/A	7122	N/A	0.699	
Total		5		3351630	318055			0.00000				

Figure S85. HPLC of compound **11**.

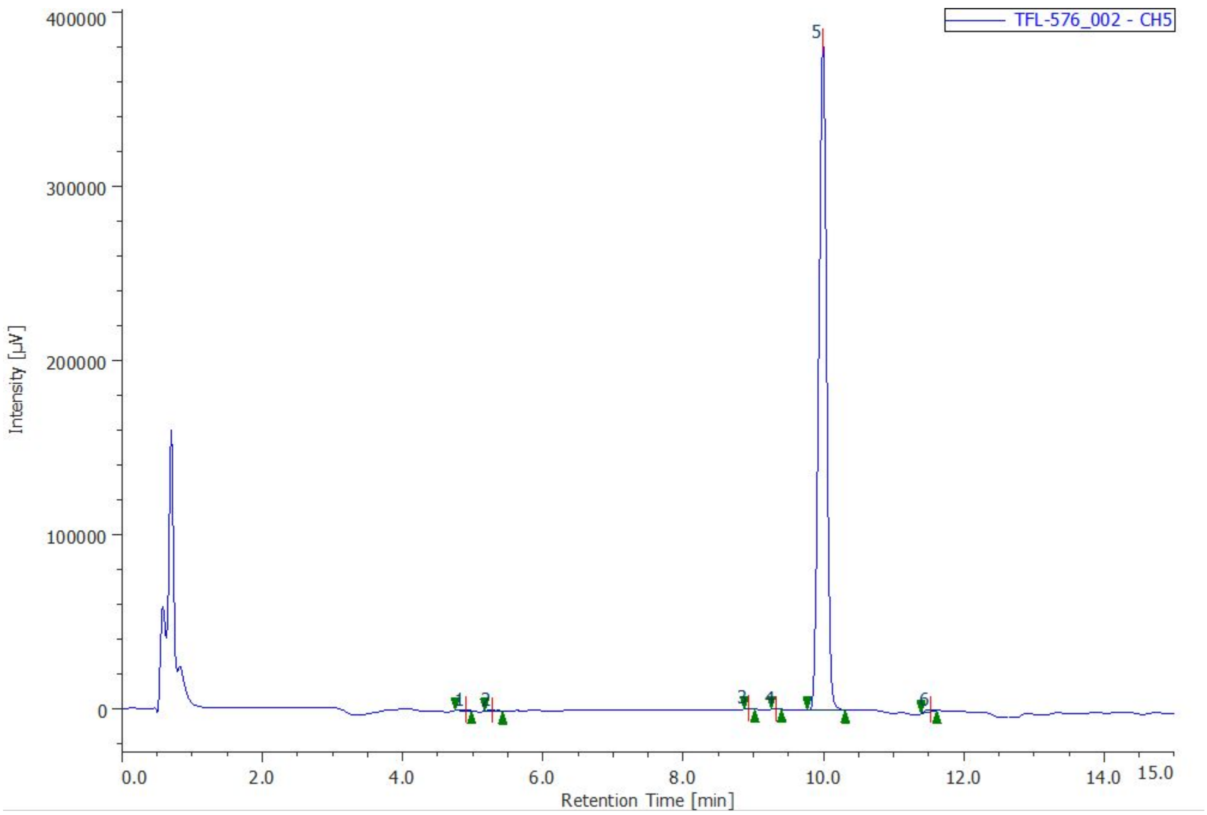

#	Peak Name	CH	tR	Area	Height	Area%	Height%	Quantity	NTP	Resolution	Symmetry Factor	Warning
1	Peak-001	5	4.900	6280	622	0.210	0.161	N/A	3977	1.255	0.756	
2	Peak-002	5	5.273	7351	751	0.246	0.194	N/A	5445	16.522	1.208	
3	Peak-003	5	8.940	1875	342	0.063	0.089	N/A	50459	2.347	1.074	
4	Peak-004	5	9.330	1540	265	0.052	0.069	N/A	46012	3.497	0.955	
5	Peak-005	5	9.990	2958274	383459	99.054	99.188	N/A	38196	6.561	0.965	
6	Peak-006	5	11.520	11221	1158	0.376	0.300	N/A	30662	N/A	0.841	
Total		5		2986541	386597			0.00000				

Figure S86. HPLC of compound **12**.

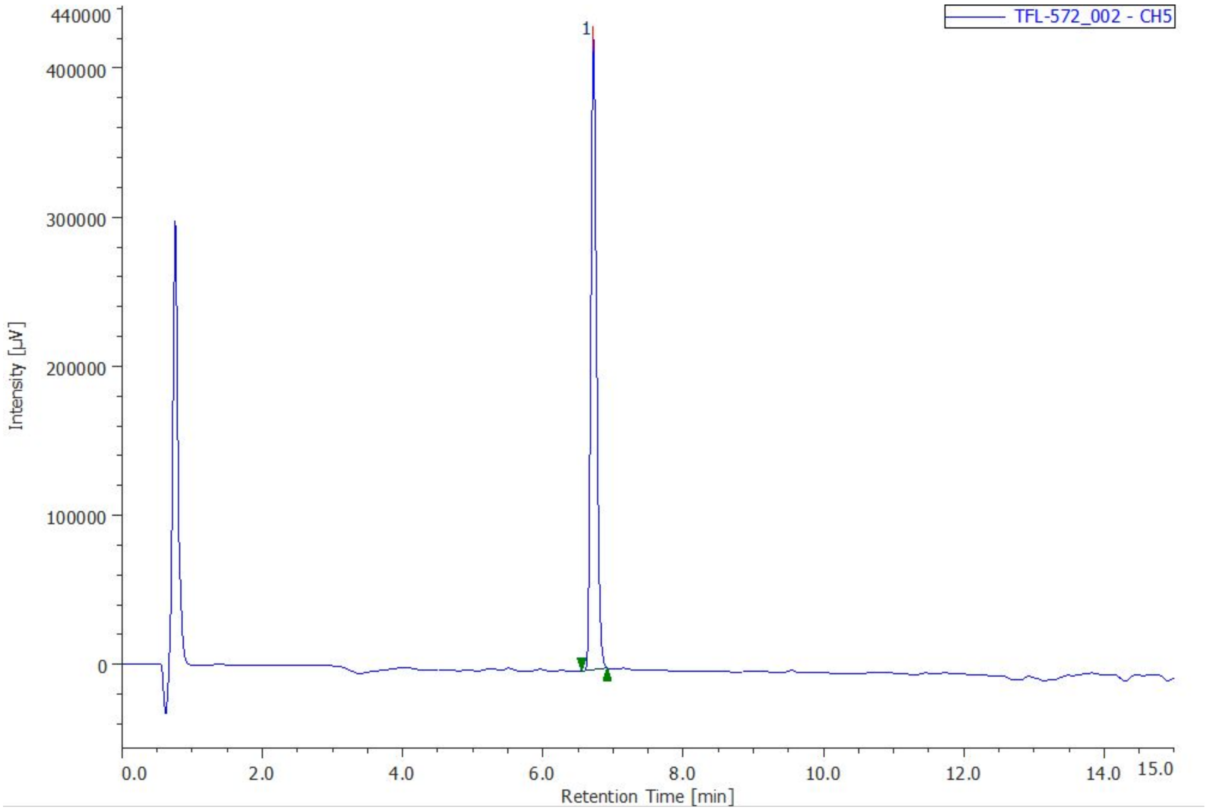

#	Peak Name	CH	tR	Area	Height	Area%	Height%	Quantity	NTP	Resolution	Symmetry Factor	Warning
1	Peak-001	5	6.720	2402952	422892	100.000	100.000	N/A	32893	N/A	1.171	
Total		5		2402952	422892			0.00000				

Figure S87. HPLC of compound **13**.

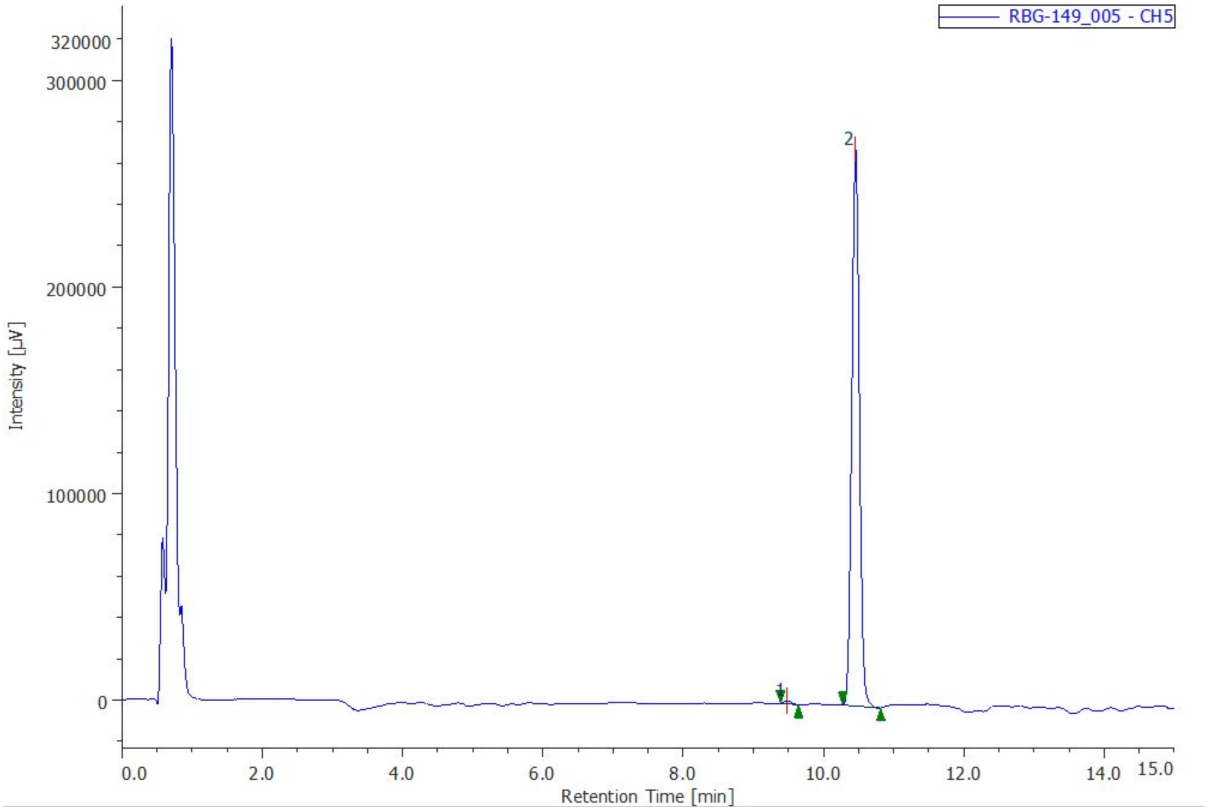

#	Peak Name	CH	tR	Area	Height	Area%	Height%	Quantity	NTP	Resolution	Symmetry Factor	Warning
1	Peak-001	5	9.480	11328	1504	0.556	0.555	N/A	32474	4.801	1.235	
2	Peak-002	5	10.457	2027837	269441	99.444	99.445	N/A	44826	N/A	1.036	
Total		5		2039165	270945			0.00000				

Figure S88. HPLC of compound **14**.

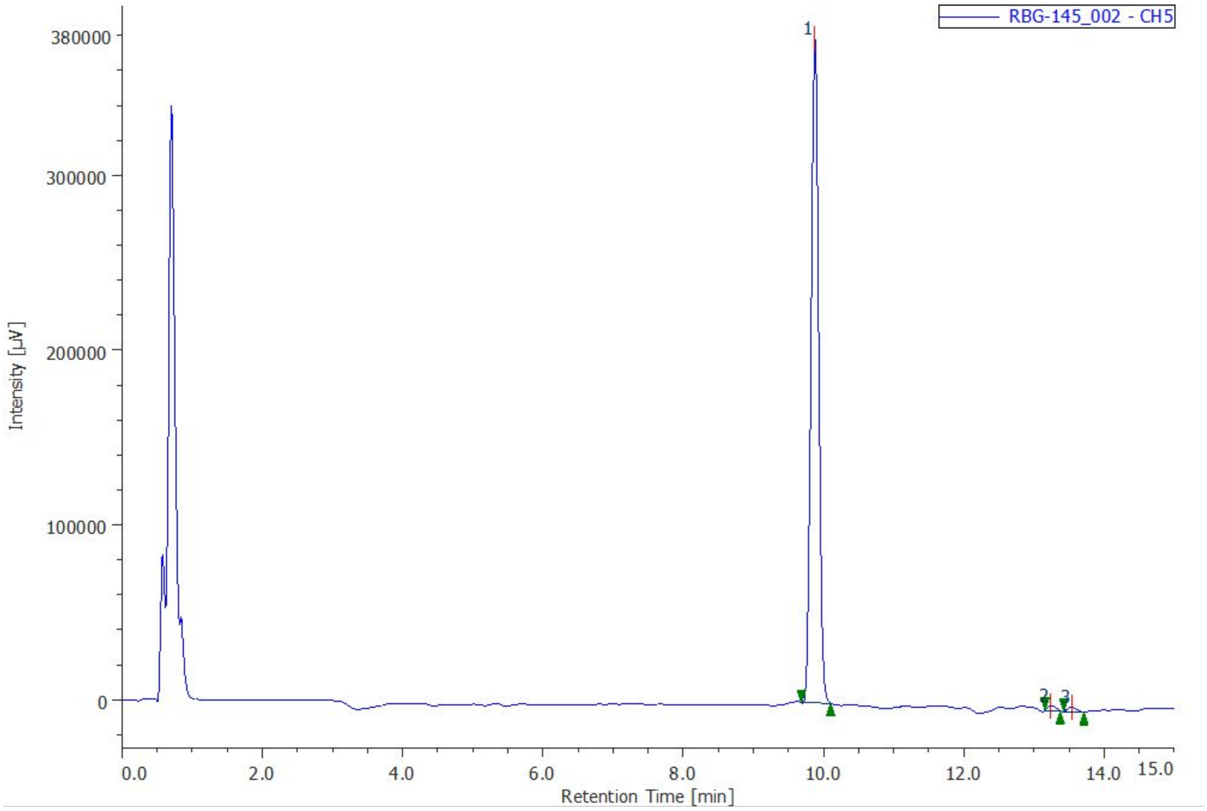

#	Peak Name	CH	tR	Area	Height	Area%	Height%	Quantity	NTP	Resolution	Symmetry Factor	Warning
1	Peak-001	5	9.877	2868510	379810	98.425	98.596	N/A	38716	15.370	1.009	
2	Peak-002	5	13.240	22308	2772	0.765	0.720	N/A	49504	1.178	1.298	
3	Peak-003	5	13.530	23593	2637	0.810	0.685	N/A	44747	N/A	1.228	
Total		5		2914411	385219			0.00000				

Figure S89. HPLC of compound **15**.

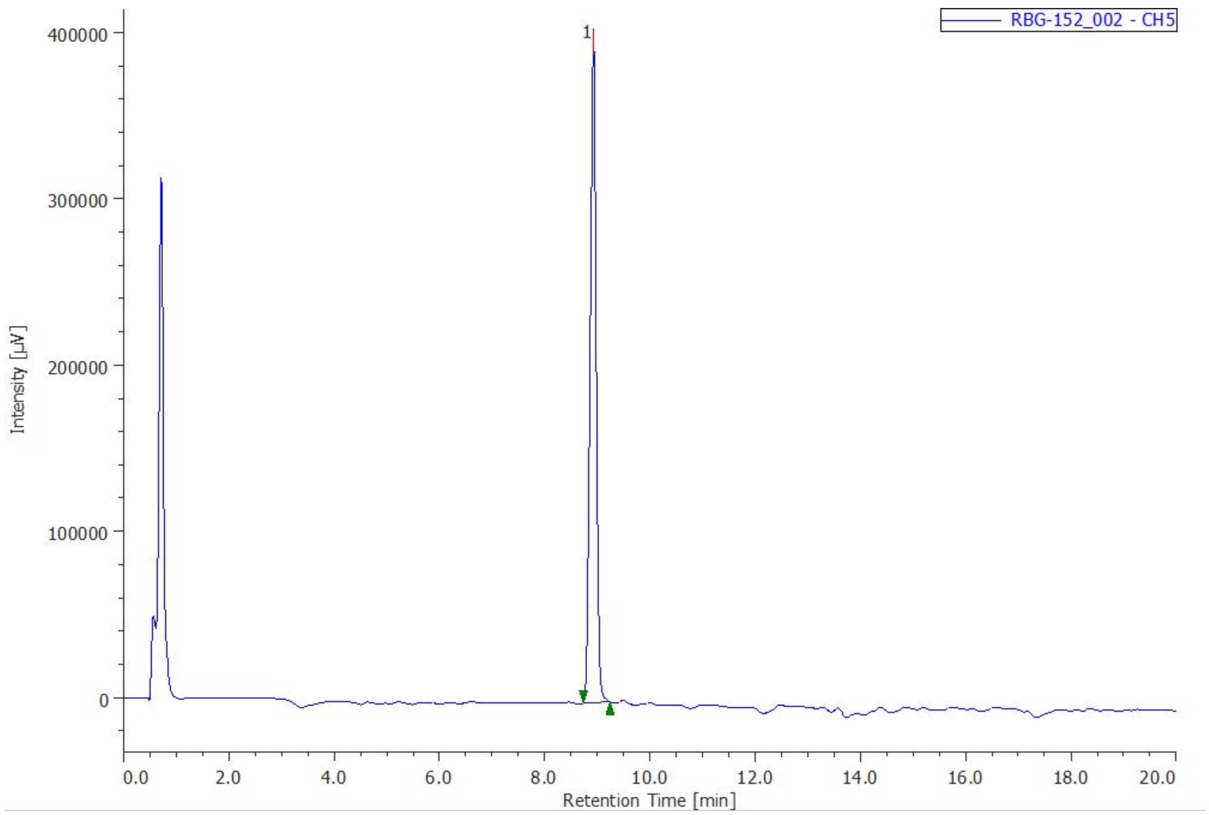

#	Peak Name	CH	tR	Area	Height	Area%	Height%	Quantity	NTP	Resolution	Symmetry Factor	Warning
1	Peak-001	5	8.927	3170854	396863	100.000	100.000	N/A	28639	N/A	0.952	
Total		5		3170854	396863			0.00000				

Figure S90. HPLC of compound **16**.

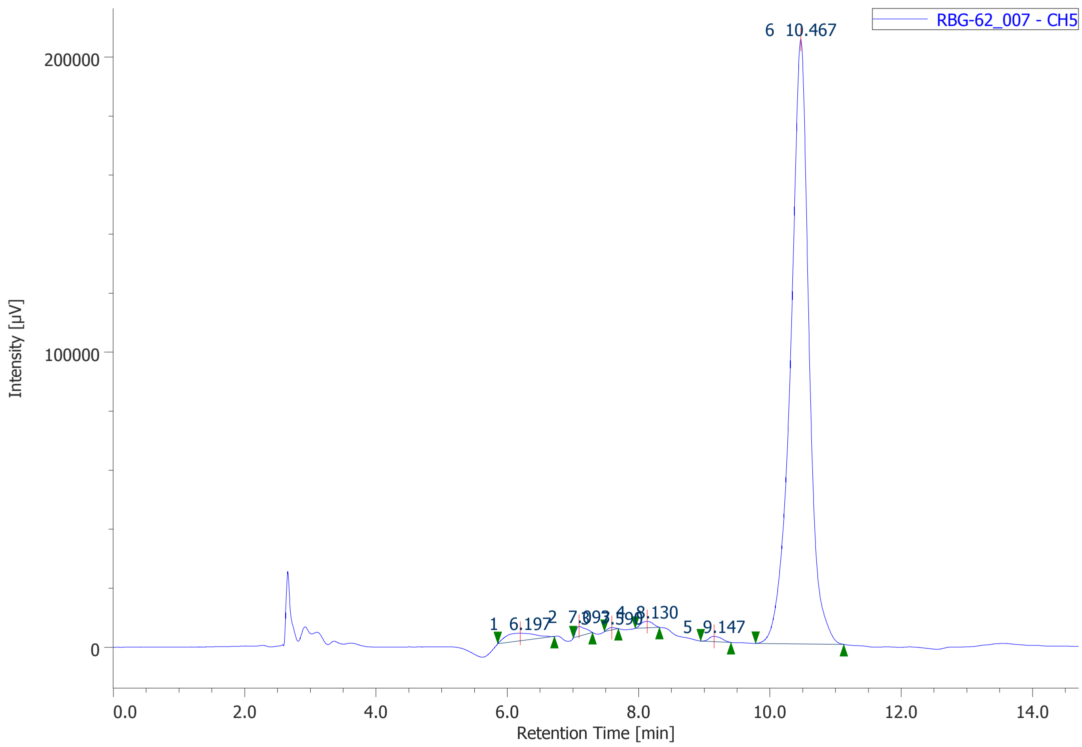

#	Peak Name	CH	tR	Area	Height	Area%	Height%	Quantity	NTP	Resolution	Symmetry Factor	Warning
1	Peak-005	5	6.197	81427	2584	1.966	1.199	N/A	785	1.524	1.186	
2	Peak-006	5	7.093	33056	3309	0.798	1.535	N/A	9227	1.908	1.635	
3	Peak-007	5	7.590	6590	828	0.159	0.384	N/A	17924	1.841	0.951	
4	Peak-008	5	8.130	27876	2203	0.673	1.022	N/A	8089	2.779	1.002	
5	Peak-001	5	9.147	24014	1817	0.580	0.843	N/A	9664	3.143	1.257	
6	Peak-002	5	10.467	3969742	204797	95.825	95.017	N/A	7935	N/A	0.940	

Figure S91. HPLC of compound **17**.

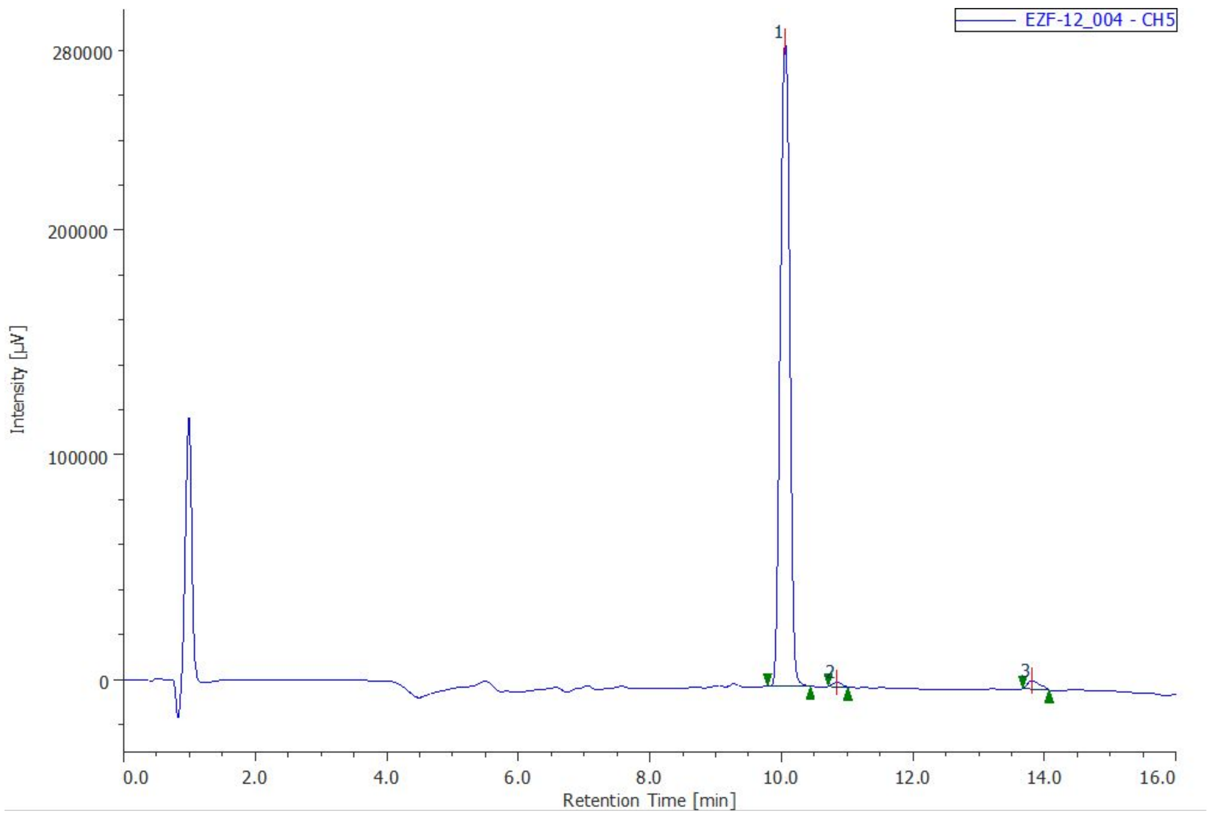

#	Peak Name	CH	tR	Area	Height	Area%	Height%	Quantity	NTP	Resolution	Symmetry Factor	Warning
1	Peak-001	5	10.053	2848199	286424	97.699	98.097	N/A	22008	2.894	1.003	
2	Peak-002	5	10.840	18220	1874	0.625	0.642	N/A	25052	9.136	1.115	
3	Peak-003	5	13.800	48860	3684	1.676	1.262	N/A	21581	N/A	1.516	
Total		5		2915279	291982			0.00000				

Figure S92. HPLC of compound **18**.

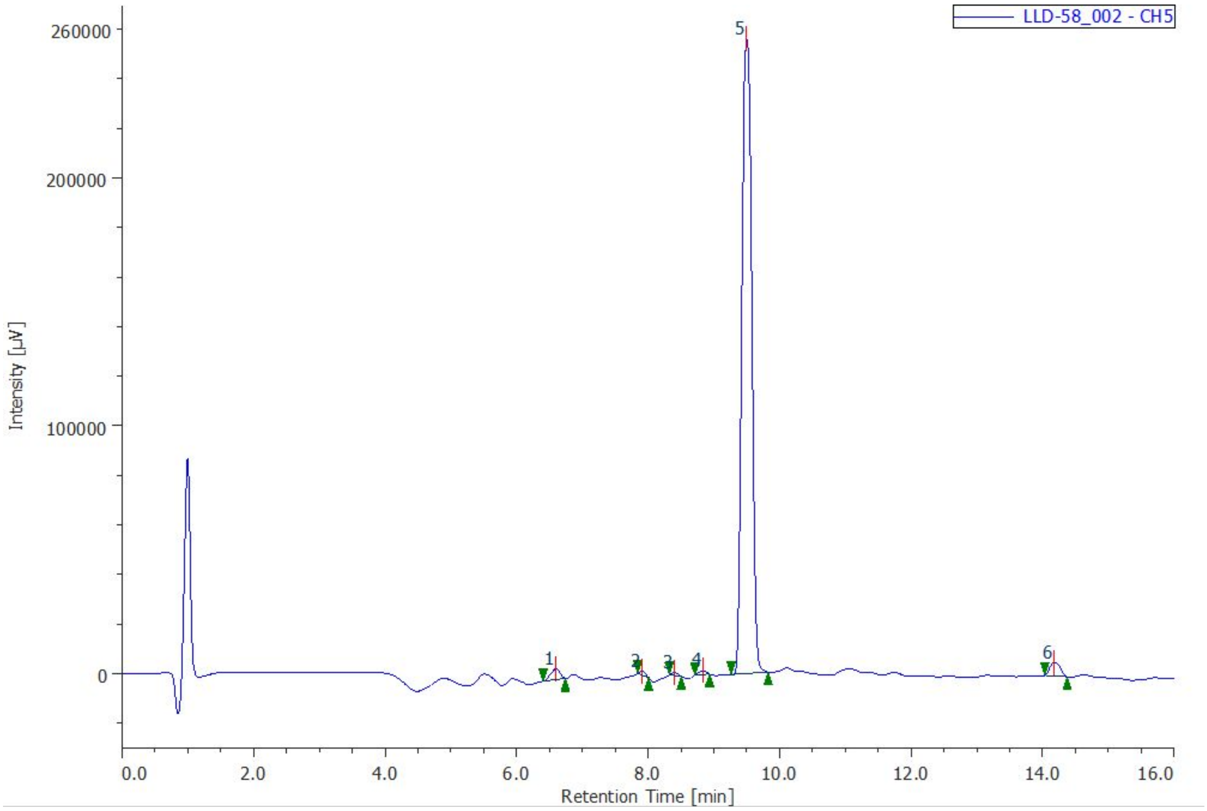

#	Peak Name	CH	tR	Area	Height	Area%	Height%	Quantity	NTP	Resolution	Symmetry Factor	Warning
1	Peak-001	5	6.597	41821	4288	1.518	1.590	N/A	9329	5.727	0.881	
2	Peak-002	5	7.903	9239	1439	0.335	0.533	N/A	29397	2.545	1.342	
3	Peak-003	5	8.393	7050	1028	0.256	0.381	N/A	27692	2.016	1.227	
4	Peak-004	5	8.833	12268	1518	0.445	0.563	N/A	22415	2.571	0.875	
5	Peak-005	5	9.497	2625565	256103	95.311	94.946	N/A	18223	15.861	1.068	
6	Peak-006	5	14.177	58786	5358	2.134	1.986	N/A	33395	N/A	1.144	
Total		5		2754729	269734			0.00000				

Figure S93. HPLC of compound **19**.

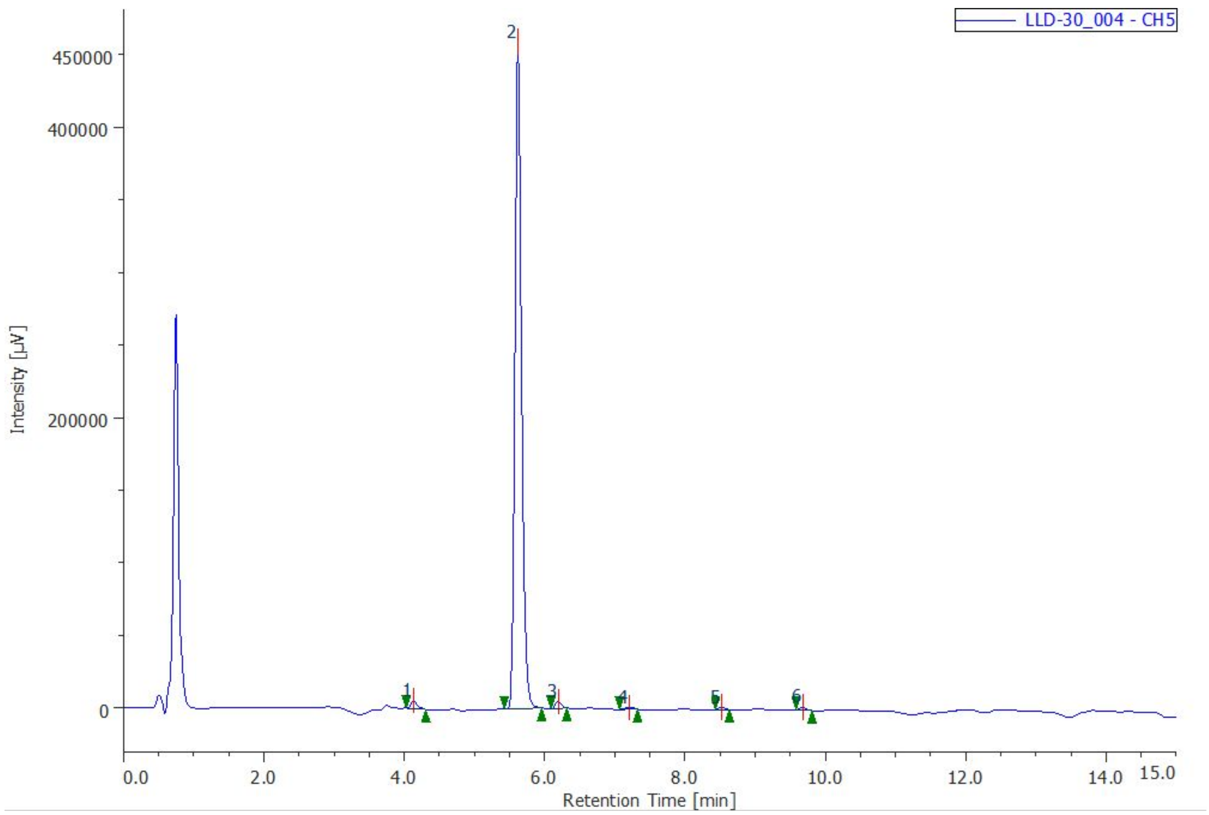

#	Peak Name	CH	tR	Area	Height	Area%	Height%	Quantity	NTP	Resolution	Symmetry Factor	Warning
1	Peak-001	5	4.130	35372	5472	1.147	1.155	N/A	10250	9.109	1.507	
2	Peak-002	5	5.617	2987210	458813	96.862	96.845	N/A	18740	3.467	1.238	
3	Peak-003	5	6.190	28370	4578	0.920	0.966	N/A	21860	4.949	1.189	
4	Peak-004	5	7.213	11719	1362	0.380	0.288	N/A	13624	6.307	0.879	
5	Peak-005	5	8.520	9238	1530	0.300	0.323	N/A	41026	7.048	1.142	
6	Peak-006	5	9.673	12063	2006	0.391	0.423	N/A	58551	N/A	1.206	
Total		5		3083972	473761			0.00000				

Figure S94. HPLC of compound **20**.

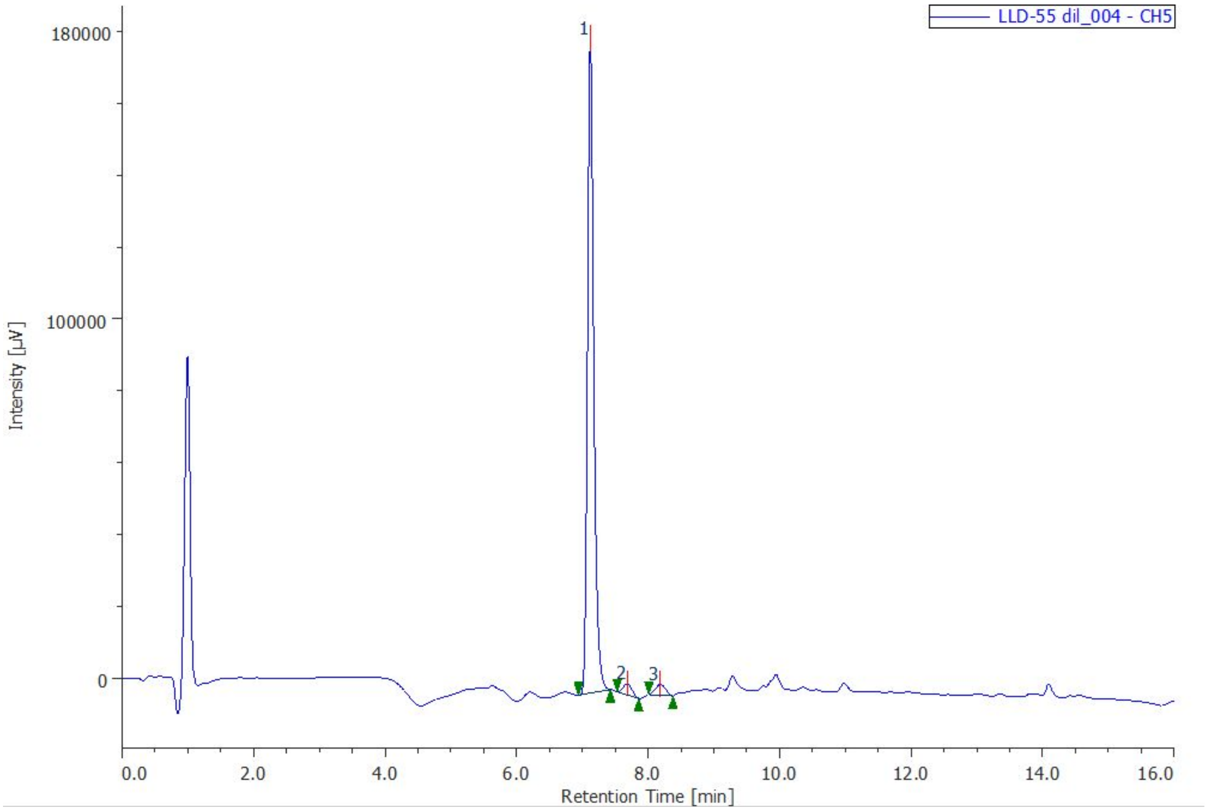

#	Peak Name	CH	tR	Area	Height	Area%	Height%	Quantity	NTP	Resolution	Symmetry Factor	Warning
1	Peak-001	5	7.120	1218850	181944	95.243	96.736	N/A	28804	2.642	1.280	
2	Peak-002	5	7.680	28792	3108	2.250	1.652	N/A	14262	1.835	1.062	
3	Peak-003	5	8.183	32086	3031	2.507	1.612	N/A	12487	N/A	1.006	
Total		5		1279728	188083			0.00000				

Figure S95. HPLC of compound **21**.

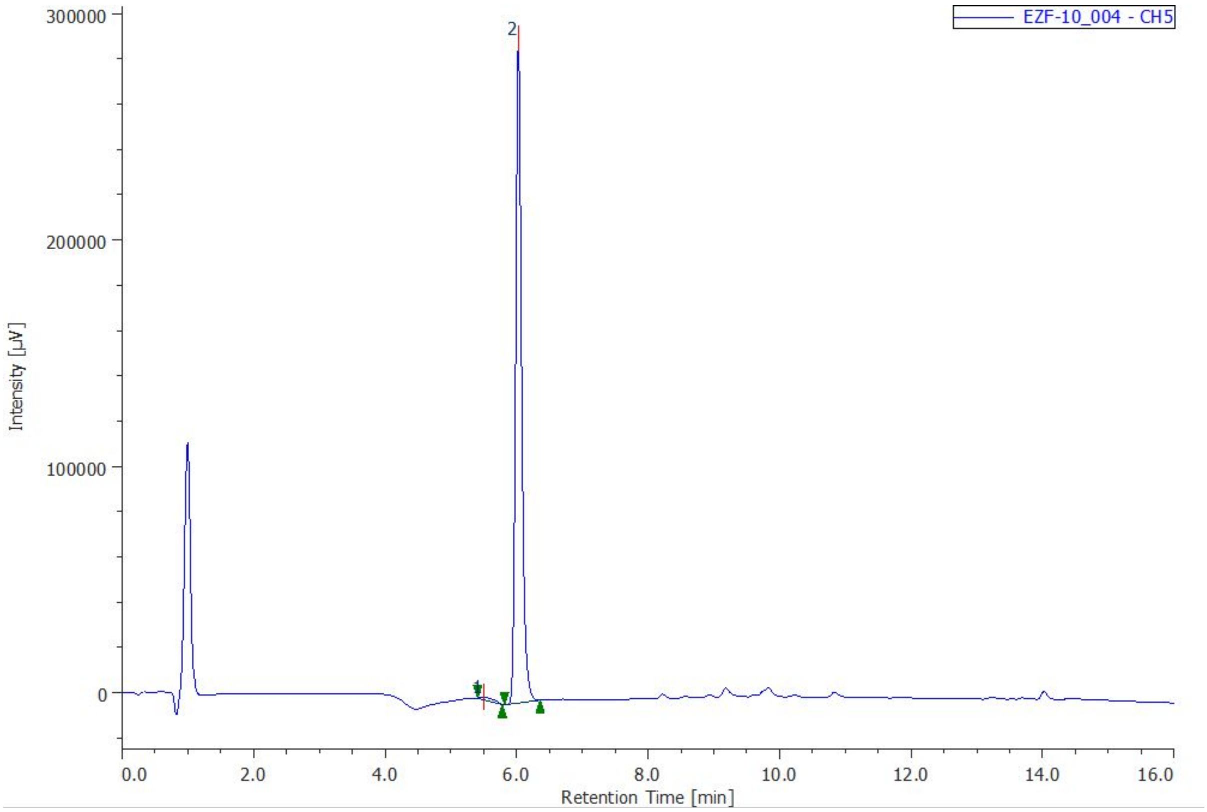

#	Peak Name	CH	tR	Area	Height	Area%	Height%	Quantity	NTP	Resolution	Symmetry Factor	Warning
1	Peak-001	5	5.500	19370	1141	1.032	0.388	N/A	2216	1.687	1.965	
2	Peak-002	5	6.027	1857727	292966	98.968	99.612	N/A	23122	N/A	1.217	
Total		5		1877097	294107			0.00000				

Figure S96. HPLC of compound **22**.

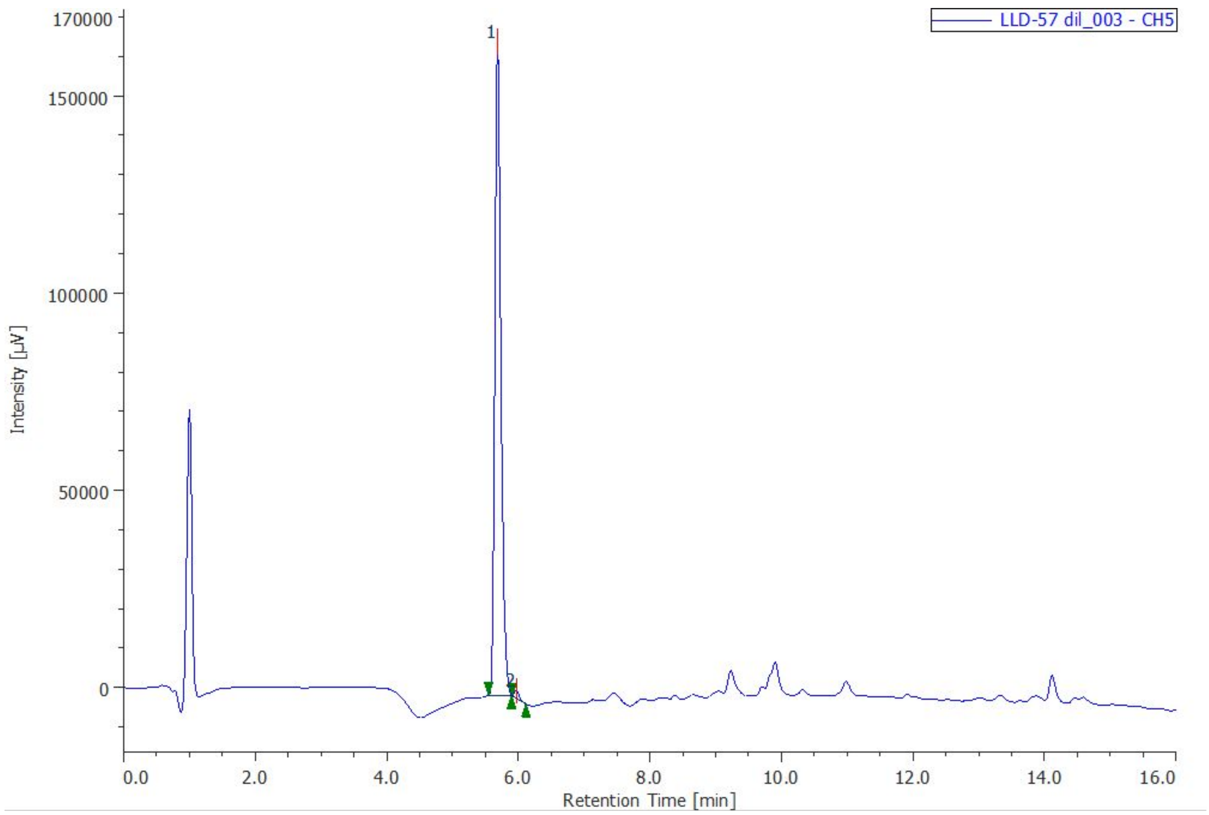

#	Peak Name	CH	tR	Area	Height	Area%	Height%	Quantity	NTP	Resolution	Symmetry Factor	Warning
1	Peak-001	5	5.683	1058620	165496	99.105	98.845	N/A	19268	1.903	1.273	
2	Peak-002	5	5.967	9560	1934	0.895	1.155	N/A	31369	N/A	1.366	
Total		5		1068180	167430			0.00000				

Figure S97. HPLC of compound **23**.

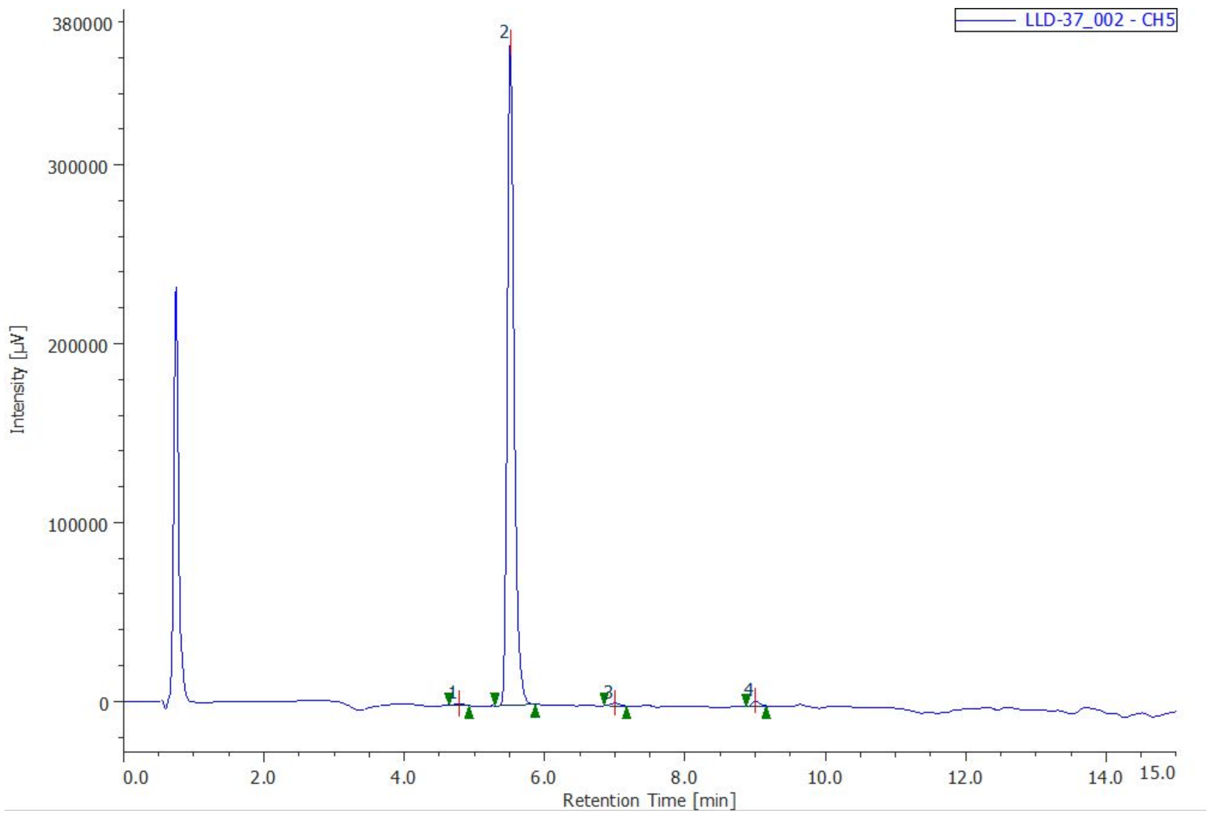

#	Peak Name	CH	tR	Area	Height	Area%	Height%	Quantity	NTP	Resolution	Symmetry Factor	Warning
1	Peak-001	5	4.780	10392	1141	0.405	0.303	N/A	5463	3.415	1.013	
2	Peak-002	5	5.510	2516651	370580	98.099	98.519	N/A	16805	6.572	1.303	
3	Peak-003	5	7.000	15362	1510	0.599	0.401	N/A	9676	8.029	1.046	
4	Peak-004	5	9.000	23016	2920	0.897	0.776	N/A	28069	N/A	1.183	
Total		5		2565421	376151			0.00000				

Figure S98. HPLC of compound **24**.

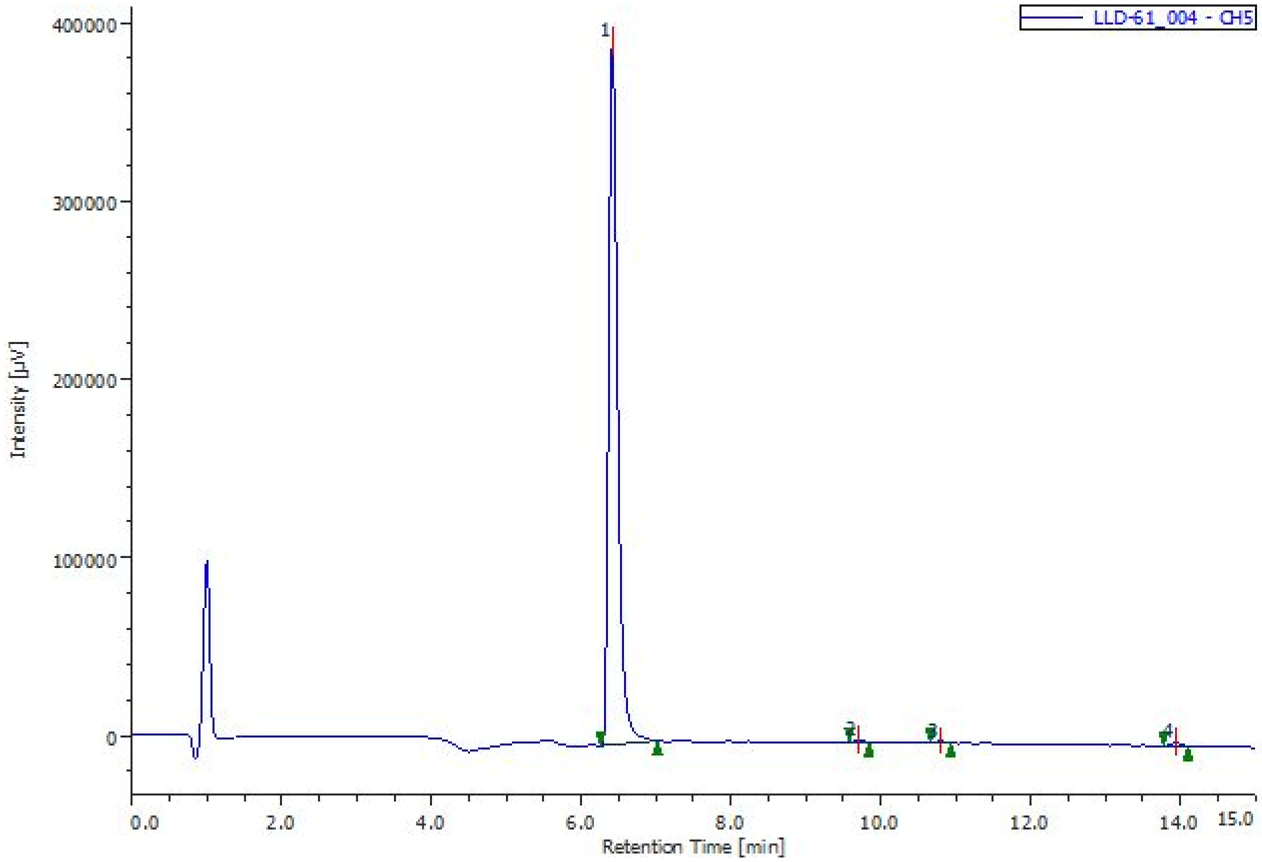

#	Peak Name	CH	tR	Area	Height	Area%	Height%	Quantity	NTP	Resolution	Symmetry Factor	Warning
1	Peak-001	5	6.413	3340152	394447	98.848	98.748	N/A	14667	15.482	1.440	
2	Peak-002	5	9.700	10859	1417	0.321	0.355	N/A	32911	4.915	1.191	
3	Peak-003	5	10.787	10668	1297	0.316	0.325	N/A	35356	14.534	1.078	
4	Peak-004	5	13.937	17388	2287	0.515	0.572	N/A	73833	N/A	1.110	
Total		5		3379067	399448			0.00000				

Figure S99. HPLC of compound **25**.

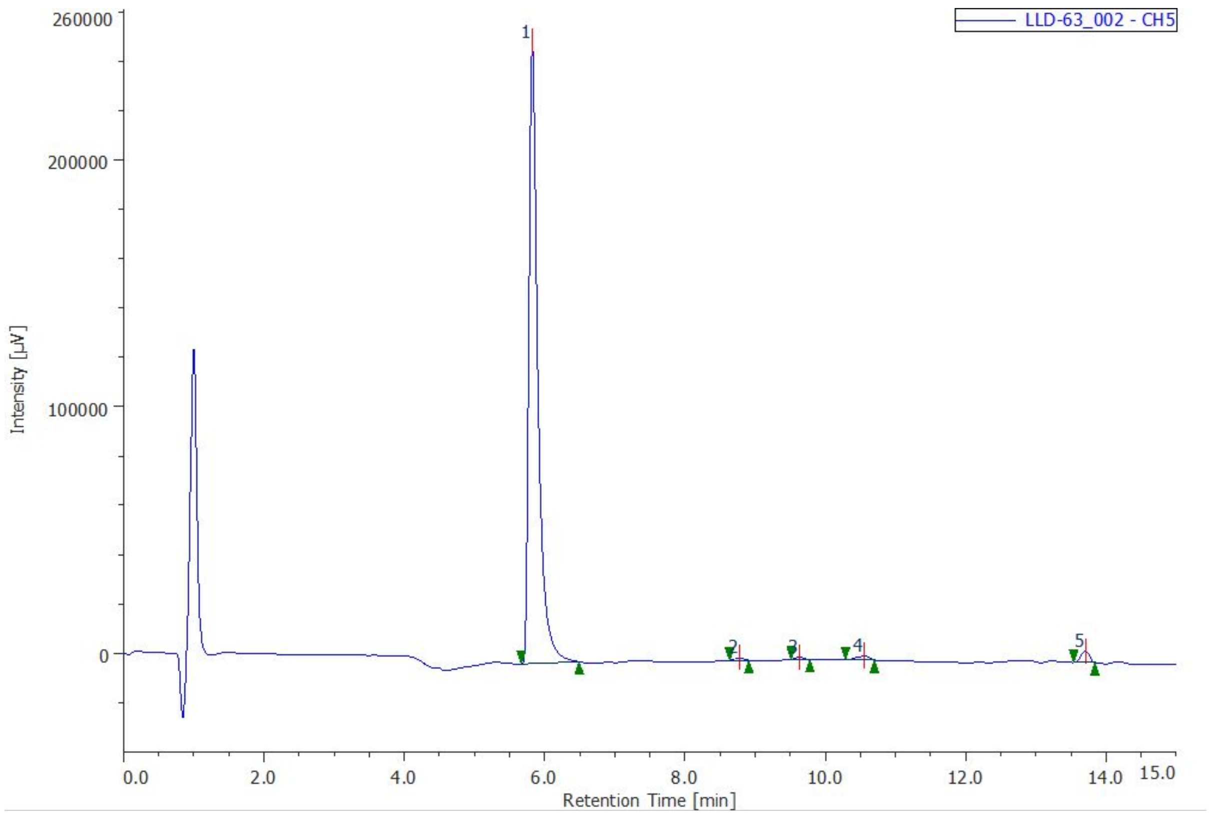

#	Peak Name	CH	tR	Area	Height	Area%	Height%	Quantity	NTP	Resolution	Symmetry Factor	Warning
1	Peak-002	5	5.823	2261893	251695	96.837	96.934	N/A	11797	13.150	1.734	
2	Peak-003	5	8.777	8567	1025	0.367	0.395	N/A	22146	3.776	0.917	
3	Peak-004	5	9.627	7173	916	0.307	0.353	N/A	31927	3.168	1.102	
4	Peak-005	5	10.553	19389	1543	0.830	0.594	N/A	12948	10.306	0.799	
5	Peak-006	5	13.703	38742	4475	1.659	1.724	N/A	51331	N/A	0.934	
Total		5		2335764	259654			0.00000				

Figure S100. HPLC of compound **26**.

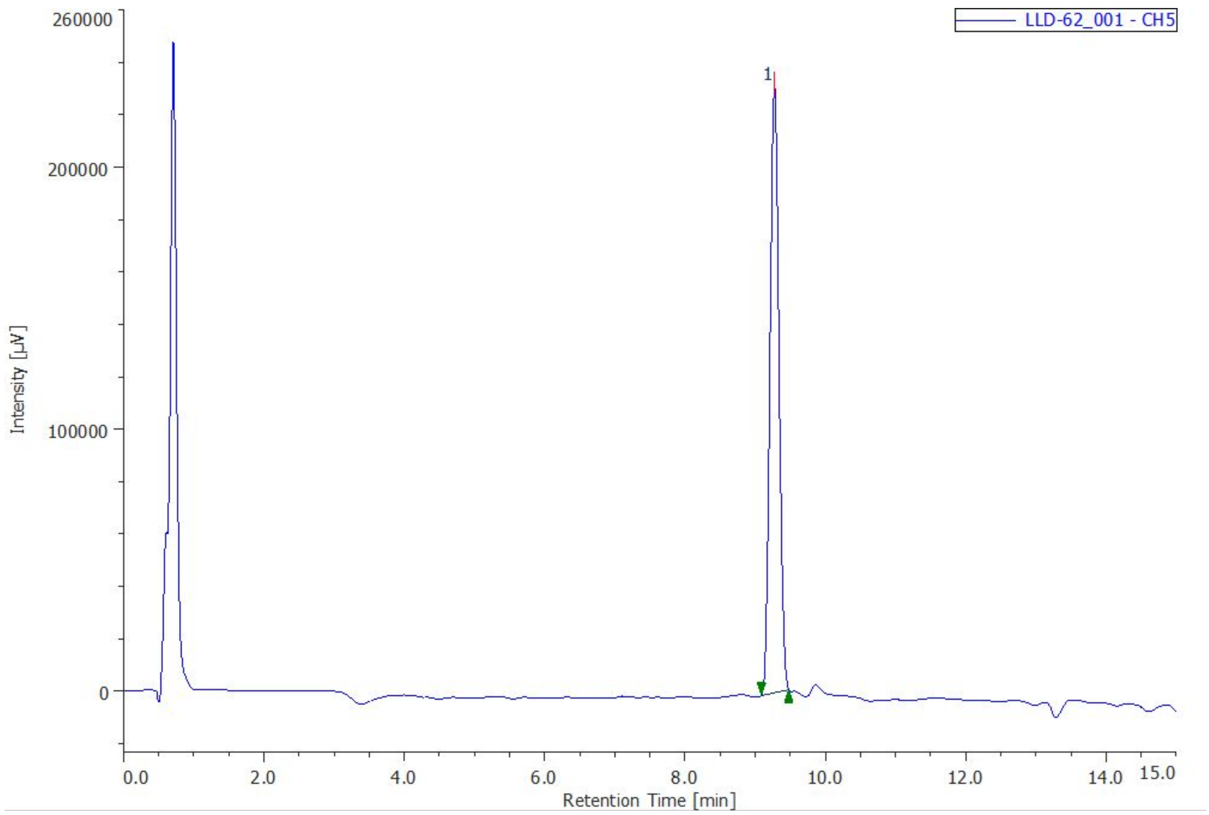

#	Peak Name	CH	tR	Area	Height	Area%	Height%	Quantity	NTP	Resolution	Symmetry Factor	Warning
1	Peak-001	5	9.273	2069810	232109	100.000	100.000	N/A	24221	N/A	1.054	
Total		5		2069810	232109			0.00000				

Figure S101. HPLC of compound **27**.

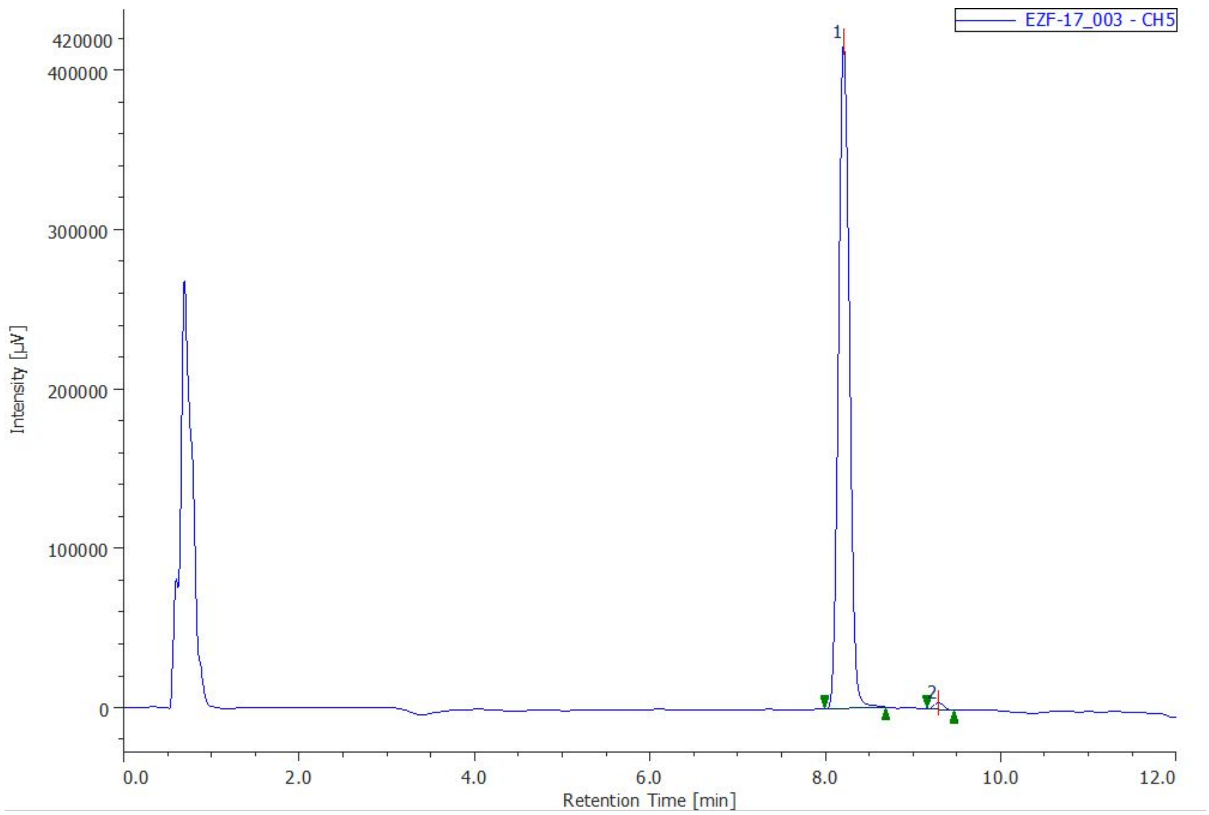

#	Peak Name	CH	tR	Area	Height	Area%	Height%	Quantity	NTP	Resolution	Symmetry Factor	Warning
1	Peak-001	5	8.207	3620924	417796	99.145	99.077	N/A	20085	4.775	1.088	
2	Peak-002	5	9.287	31239	3892	0.855	0.923	N/A	28022	N/A	1.123	
Total		5		3652163	421688			0.00000				

Figure S102. HPLC of compound **28**.

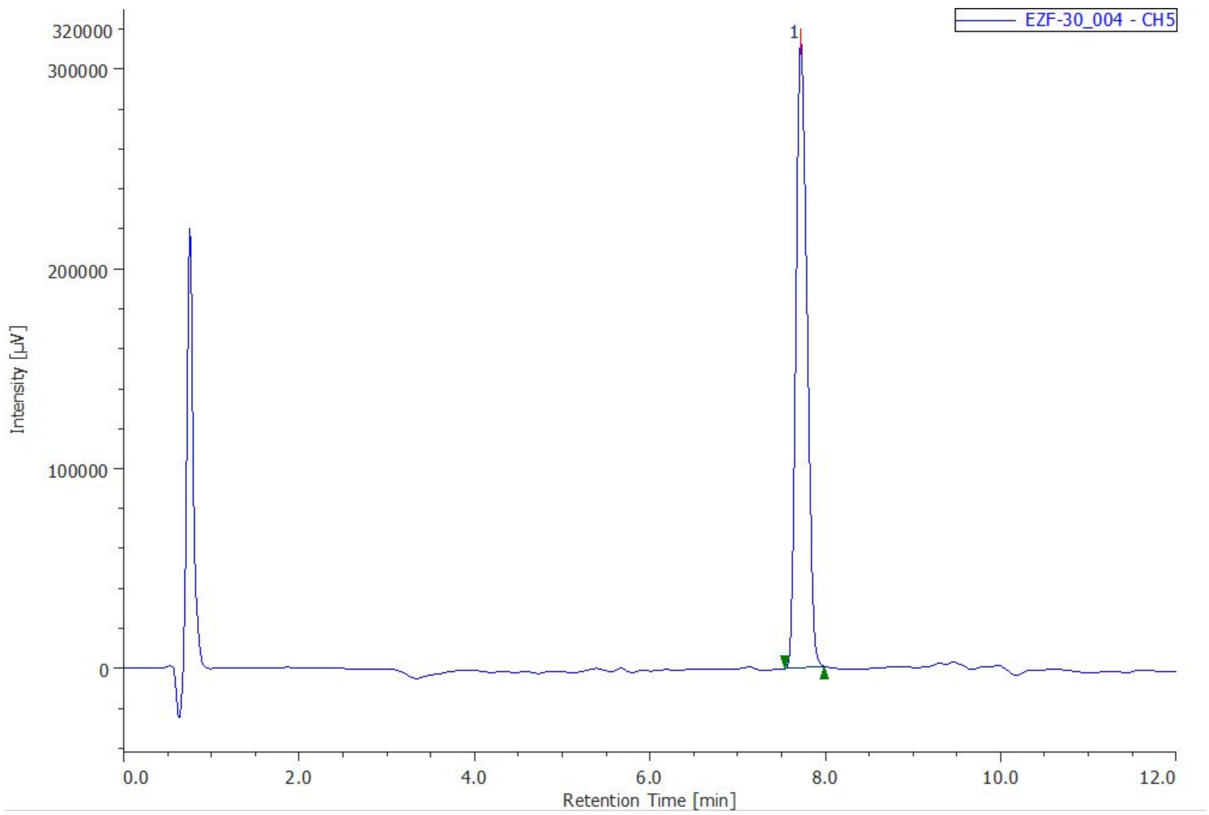

#	Peak Name	CH	tR	Area	Height	Area%	Height%	Quantity	NTP	Resolution	Symmetry Factor	Warning
1	Peak-001	5	7.717	2749917	312735	100.000	100.000	N/A	16546	N/A	1.193	
Total		5		2749917	312735			0.00000				

Figure S103. HPLC of compound **29**.

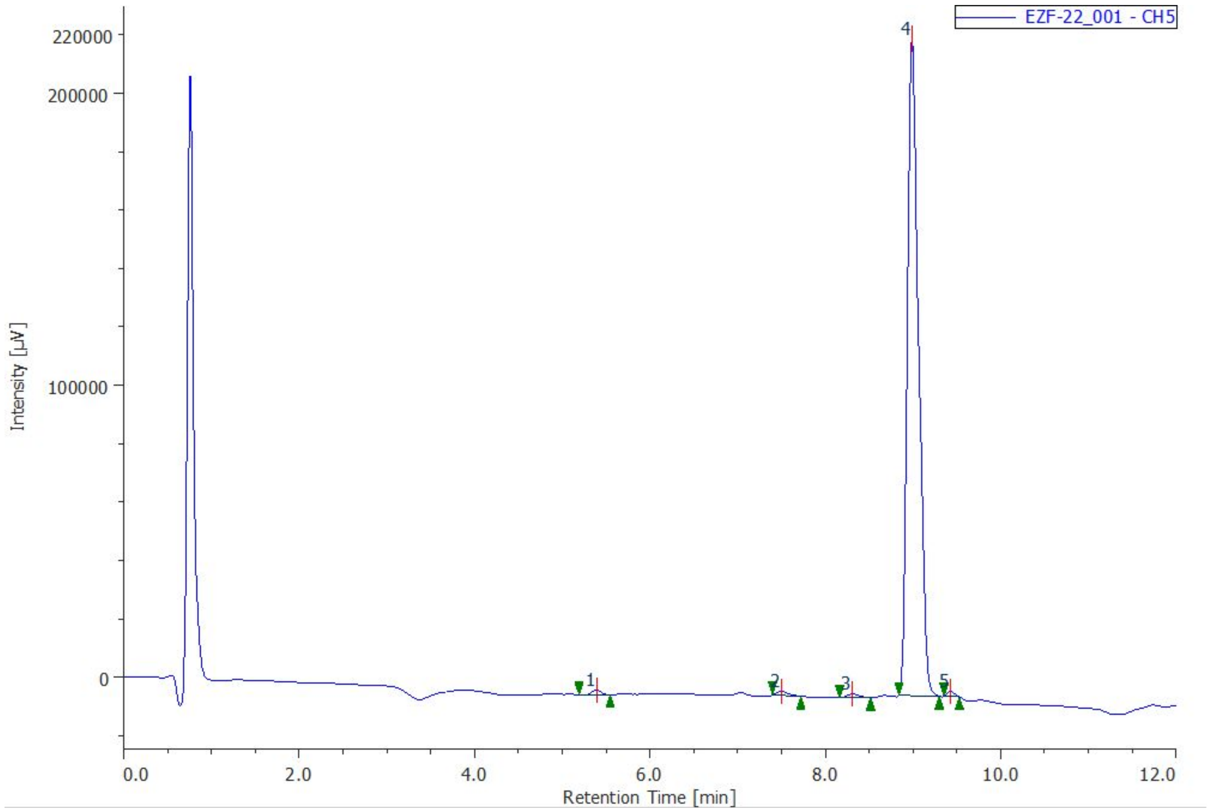

#	Peak Name	CH	tR	Area	Height	Area%	Height%	Quantity	NTP	Resolution	Symmetry Factor	Warning
1	Peak-001	5	5.390	12113	1682	0.592	0.729	N/A	12291	9.561	1.019	
2	Peak-002	5	7.497	12572	1371	0.615	0.594	N/A	14695	3.365	1.532	
3	Peak-003	5	8.307	12079	1349	0.591	0.585	N/A	19935	2.883	1.178	
4	Peak-004	5	8.987	1998601	224653	97.737	97.377	N/A	22889	2.204	1.280	
5	Peak-005	5	9.427	9509	1649	0.465	0.715	N/A	53711	N/A	1.176	
Total		5		2044874	230704			0.00000				

Figure S104. HPLC of compound **30**.

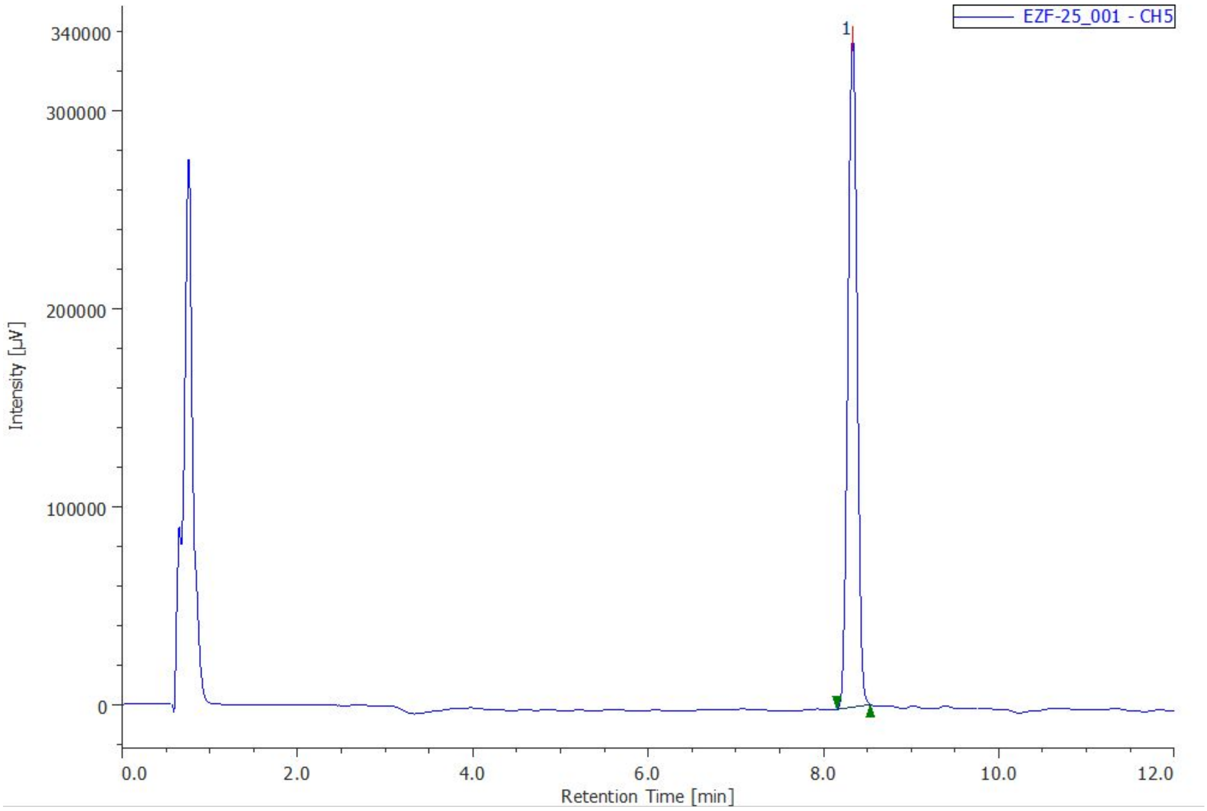

#	Peak Name	CH	tR	Area	Height	Area%	Height%	Quantity	NTP	Resolution	Symmetry Factor	Warning
1	Peak-001	5	8.330	2474759	337699	100.000	100.000	N/A	28887	N/A	1.006	
Total		5		2474759	337699			0.00000				

Figure S105. HPLC of compound **31**.

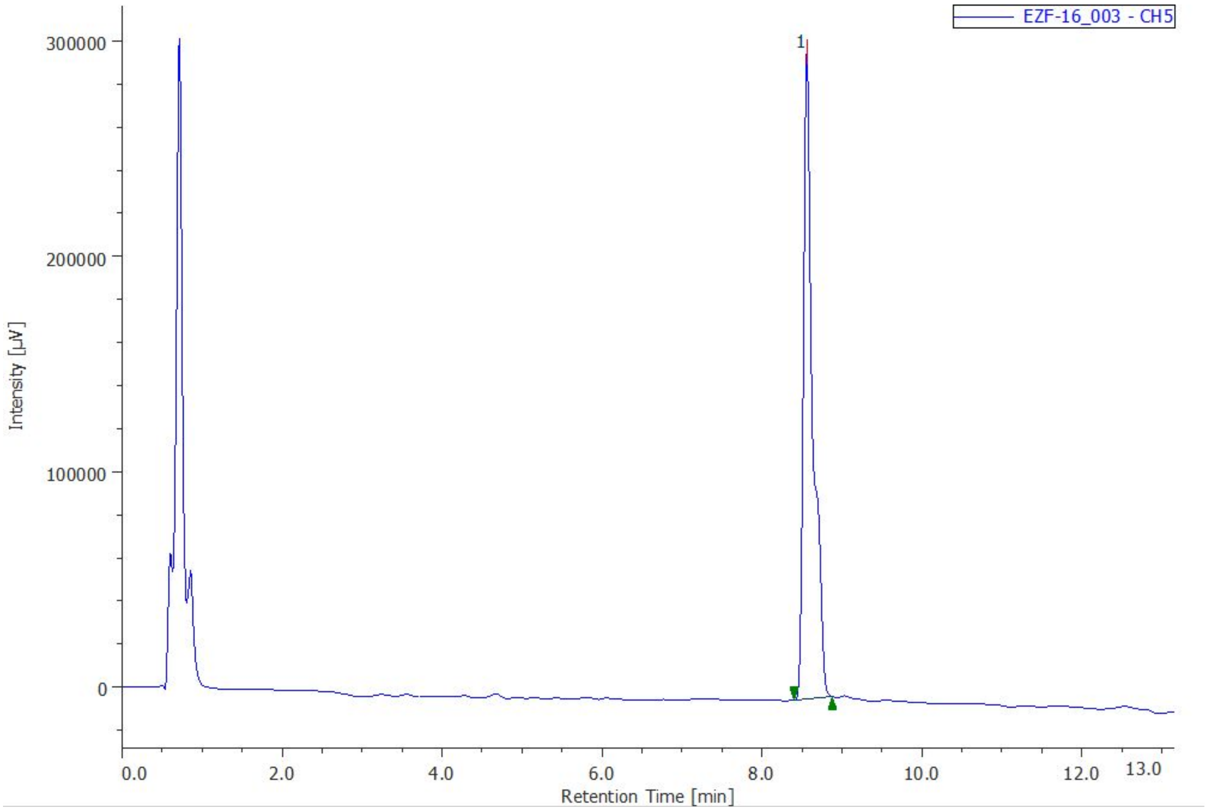

#	Peak Name	CH	tR	Area	Height	Area%	Height%	Quantity	NTP	Resolution	Symmetry Factor	Warning
1	Peak-001	5	8.560	2462865	300152	100.000	100.000	N/A	38634	N/A	1.625	
Total		5		2462865	300152			0.00000				

Figure S106. HPLC of compound **32**.

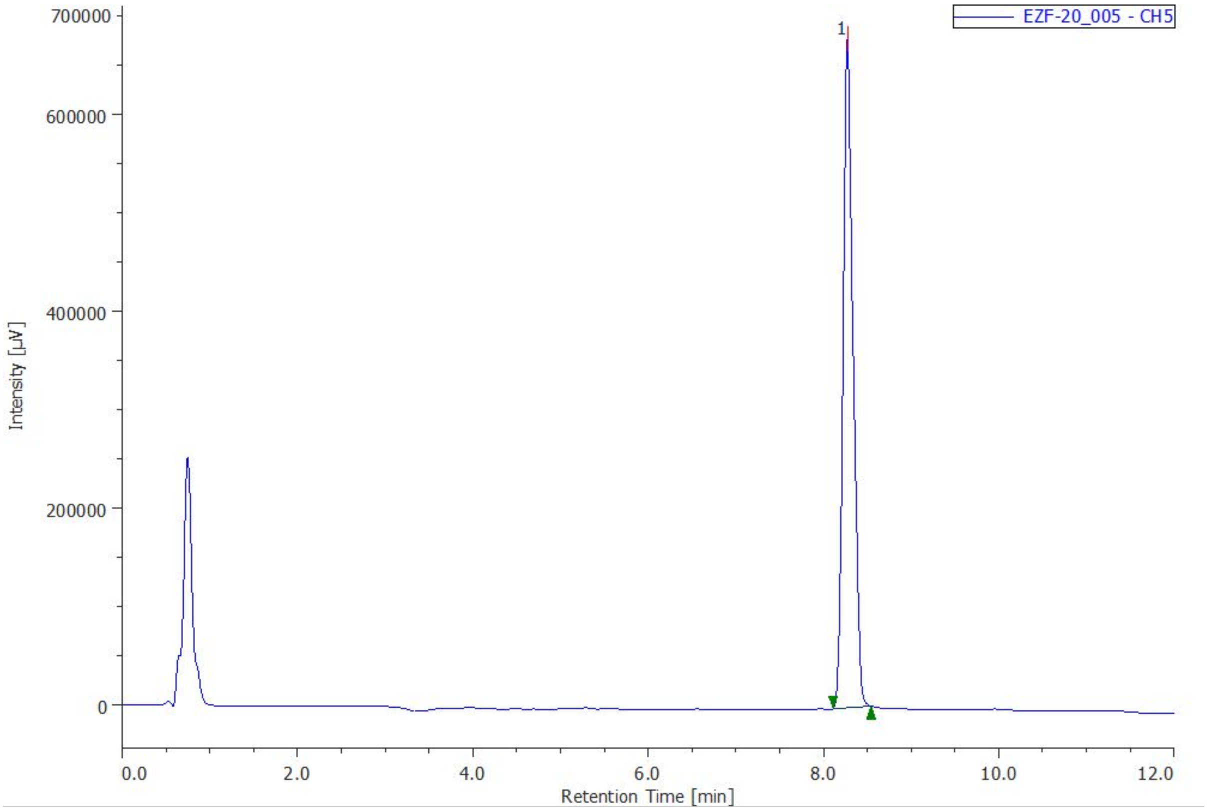

#	Peak Name	CH	tR	Area	Height	Area%	Height%	Quantity	NTP	Resolution	Symmetry Factor	Warning
1	Peak-001	5	8.270	5400624	679122	100.000	100.000	N/A	25098	N/A	1.199	
Total		5		5400624	679122			0.00000				

Figure S107. HPLC of compound **33**.

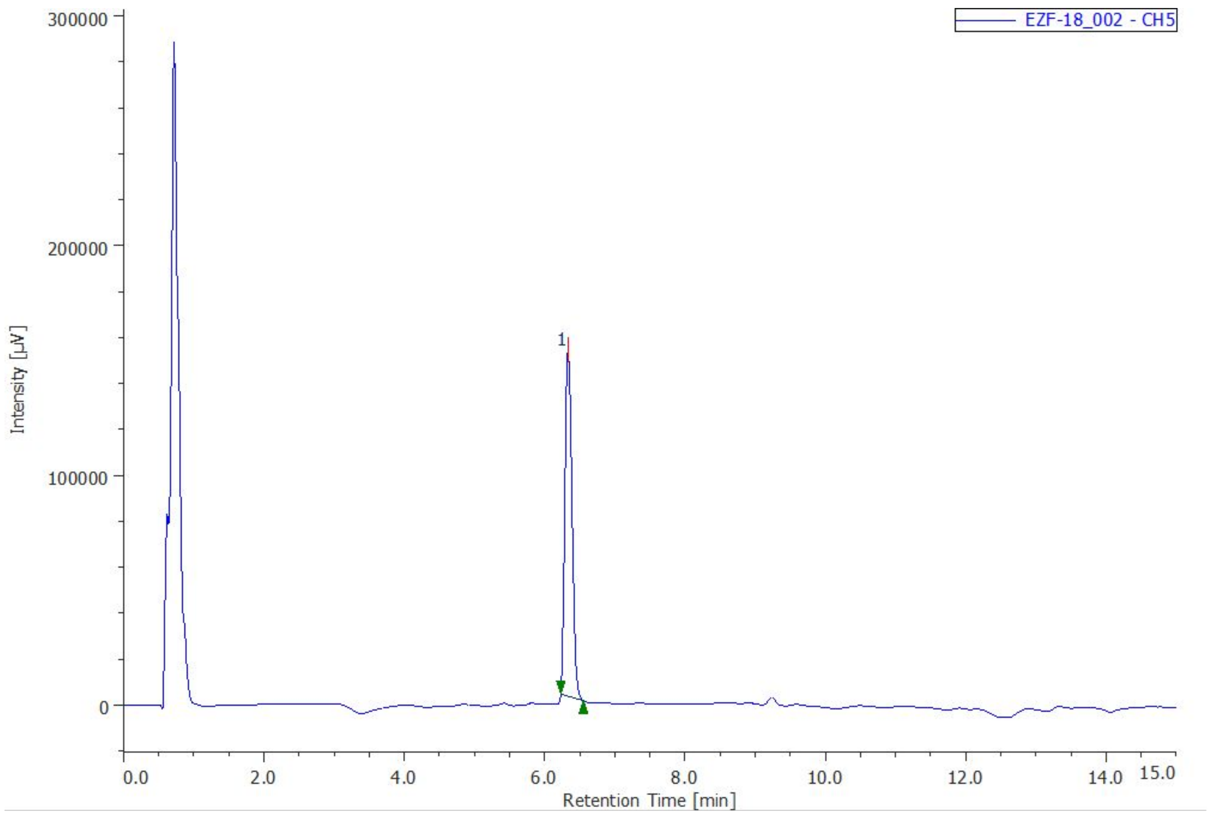

#	Peak Name	CH	tR	Area	Height	Area%	Height%	Quantity	NTP	Resolution	Symmetry Factor	Warning
1	Peak-001	5	6.333	1064753	150566	100.000	100.000	N/A	17520	N/A	1.204	
Total		5		1064753	150566			0.00000				

Figure S108. HPLC of compound **34**.

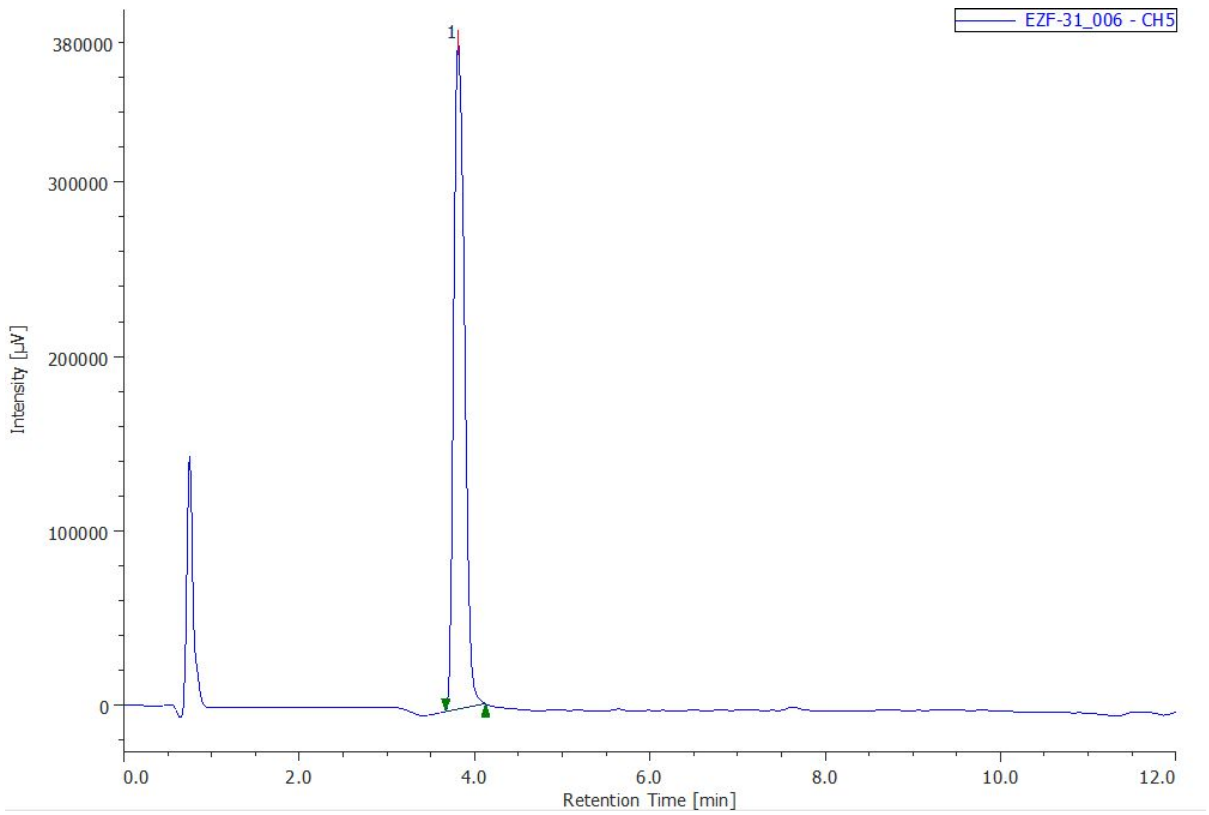

#	Peak Name	CH	tR	Area	Height	Area%	Height%	Quantity	NTP	Resolution	Symmetry Factor	Warning
1	Peak-001	5	3.813	3284653	381825	100.000	100.000	N/A	4260	N/A	1.270	
Total		5		3284653	381825			0.00000				

Figure S109. HPLC of compound **35**.

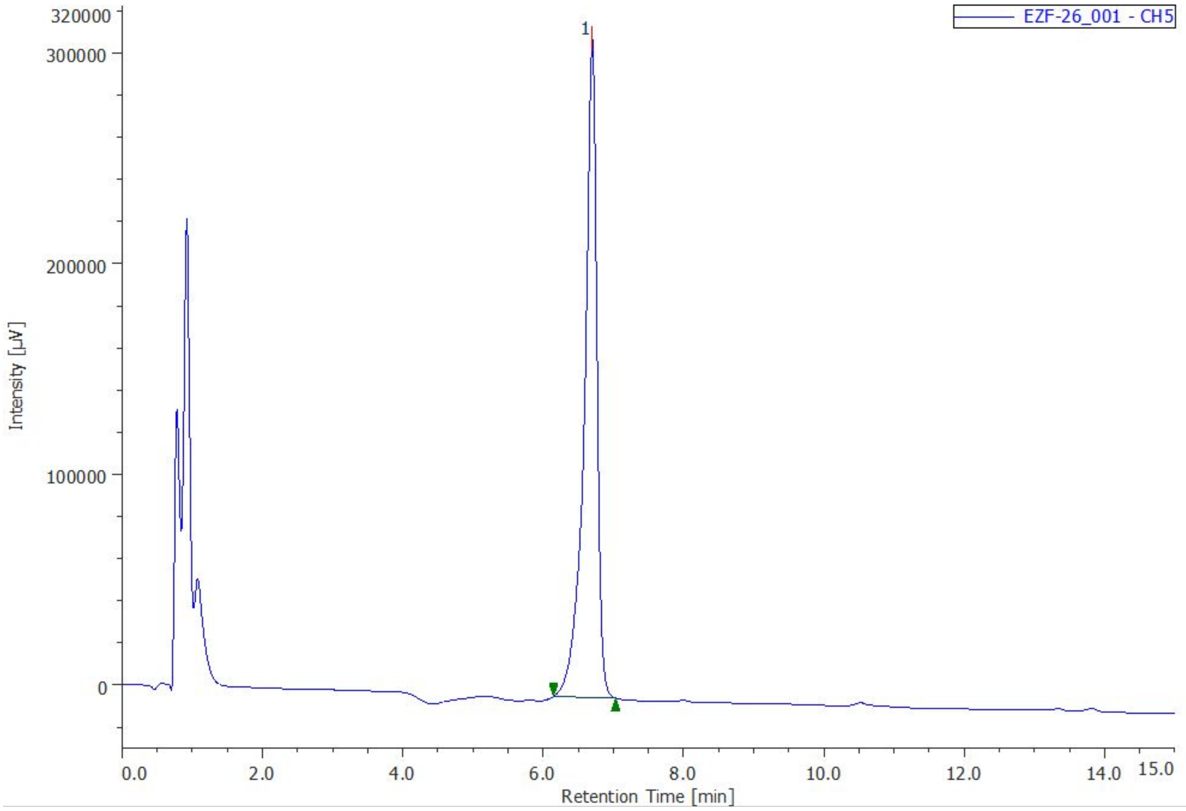

#	Peak Name	CH	tR	Area	Height	Area%	Height%	Quantity	NTP	Resolution	Symmetry Factor	Warning
1	Peak-001	5	6.700	3867127	312515	100.000	100.000	N/A	8813	N/A	0.739	
Total		5		3867127	312515			0.00000				

Figure S110. HPLC of compound **36**.

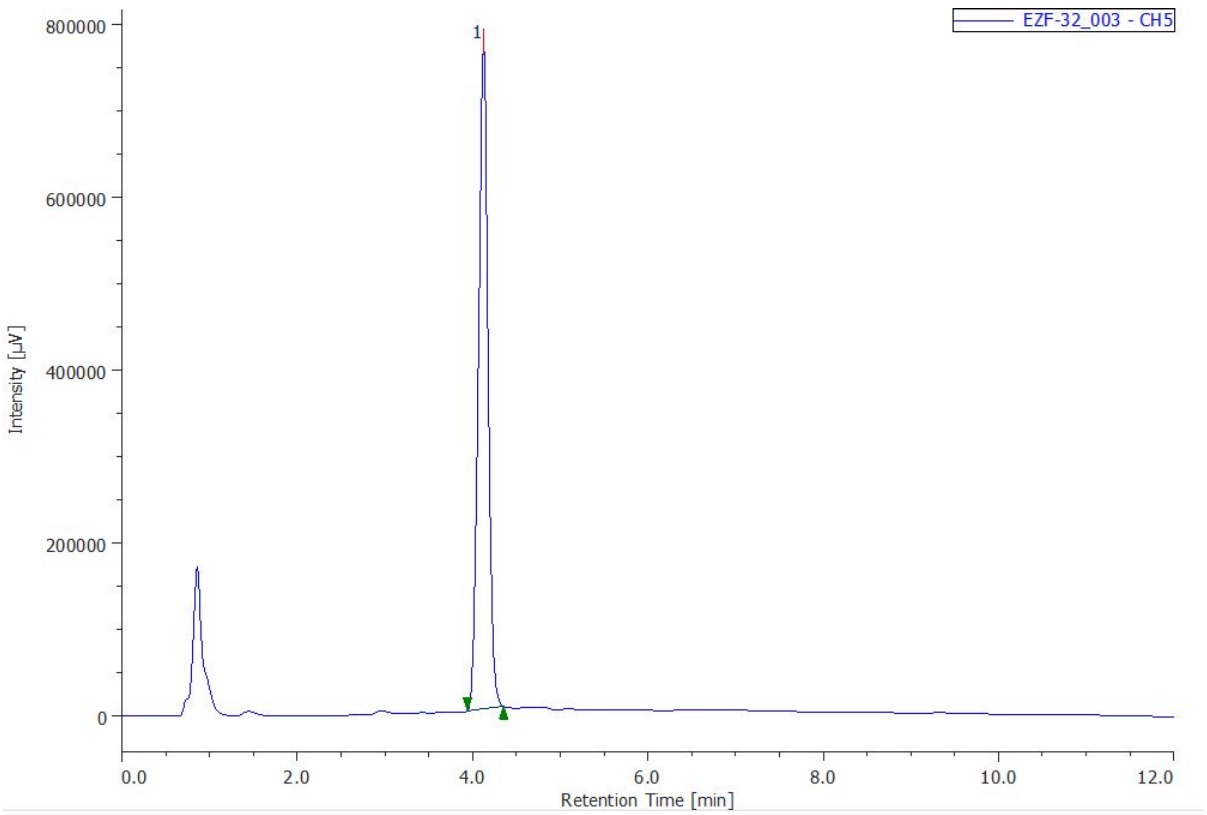

#	Peak Name	CH	tR	Area	Height	Area%	Height%	Quantity	NTP	Resolution	Symmetry Factor	Warning
1	Peak-001	5	4.127	5882911	770154	100.000	100.000	N/A	6964	N/A	0.975	
Total		5		5882911	770154			0.00000				

Figure S111. HPLC of compound **37**.

Supplementary Methods

Maintenance of human embryonic stem cell cultures

Human Embryonic Stem Cells (hESC) lines H9 (WA09) were cultured in feeder-free condition on hESC- qualified Matrigel (Corning) in complete mTSER+ media (STEMCELL Technologies). The colonies were manually passaged by mechanical scraping with 0.5mM EDTA (Invitrogen; pH=8) every 4-6 days and maintained at 37 °C with 5% CO_2.

Culture of human dorsal forebrain organoid

The dorsal forebrain organoid was generated using STEMdiff™ Dorsal Organoid Kit (STEMCELL Technologies) following the manufacture protocol with some modification.

To initiate the generation of human dorsal forebrain organoid, intact WA09 colonies were lifted from the plates using Accutase for 5 minutes and resuspended with complete mTSER plus media. Viable cells were count and calculated to obtained 2.1×10^6 total cells. Single-cell suspension was centrifuge at 300 × g for 5 minutes and resuspended with Forebrain Organoid Formation Medium containing 10 µM Y-27632 (STEMCELL Technologies) seeded 1mL in AggreWell™800 plate (STEMCELL Technologies) which was pre-treated with Anti-Adherence Rinsing Solution (STEMCELL Technologies). The plate was centrifuged at 100 ×g to capture cells into microwells and grown in the incubator at 37 °C with 5% CO_2.

From day 1-5, media was changed daily using partial medium changes using Forebrain Organoid Formation Medium. On day 6, neural aggregates were harvested using a wide-bore pipette tip and transfer individually house in the 96 low attachments well-plate supplemented with Forebrain organoid expansion media. From day 6-24, full media exchange was performed every 3 days using Forebrain Organoid Expansion Medium. On day 25, cortical organoids were harvested and placed in a 6 low attachment well-plate supplemented with Forebrain Organoid Differentiation Medium. From day 25-42, full media exchange was performed every 3 days using Forebrain Organoid Differentiation

Medium. On day 43 onwards, the organoids were cultured in Forebrain Organoid Maintenance Medium until the end of the experiment (~Day 90), with a partial media change every 3-4 days.

ZIKV infection in organoid

For infection, 3-4 single housed organoids were picked at random for dissociation and cell count to give an estimation of MOI infection. Briefly MOI 1 virus inoculation was done to the single housed organoid for 2 h in an orbital shaker in the incubator at 37 °C with 5% CO_2. After 2 h, the organoid was washed 2-3 times with the basal media to remove unbound virus and replaced with fresh culture media for further incubation. At indicated timepoints, the cultured supernatants were harvested and subjected to plaque quantification by standard BHK-21 plaque assay.

Histology and immunofluorescence

Organoids were fixed with 4% paraformaldehyde (Sigma) in 1× PB overnight at 4 °C. The next days, organoids were washed with 1× PBS and incubated in 30% sucrose solution overnight at 4 °C. Next, organoids were placed in tissue base molds and embedded within O.C.T. compound (Tissue-Tek) at -20 °C. Organoids blocks were then used for cryosectioning to obtain 20μm slices using cryostat machine (Leica). The cryosections were mounted on the charged glass slides (Thermo Fisher) and washed with 1 × PBS twice at room temperature. The sections were then permeabilized for 30 min and block for 1 h with PBST (1× PBS with 0.2% Triton X) and blocking buffer (5% donkey serum in PBST) respectively. The sections were then incubated with primary antibodies at 4 °C overnight with blocking buffer and incubate with secondary antibodies with blocking buffer for 1 h at room temperature. Nuclei were counterstained with DAPI for 10 min and cover slip was added on the glass slides with mounting media. Images were taken on Leica confocal microscopy and processed using ImageJ. The following primary antibodies were used for immunofluorescence: PAX6, (Merk; chicken), CTIP2 (Abcam; rat), SOX2 (Abcam; Rabbit), SATB2 (STEMCELL; Rabbit)
